# Enantiocontrol by assembled attractive interactions in copper-catalyzed asymmetric direct alkynylation of α-ketoesters with terminal alkynes: OH···O/sp^3^-CH···O two-point hydrogen bonding combined with dispersive attractions†
†Electronic supplementary information (ESI) available: Representative experimental procedures, spectroscopic data, and computational details. See DOI: 10.1039/c8sc00527c


**DOI:** 10.1039/c8sc00527c

**Published:** 2018-02-28

**Authors:** Martin C. Schwarzer, Akane Fujioka, Takaoki Ishii, Hirohisa Ohmiya, Seiji Mori, Masaya Sawamura

**Affiliations:** a Department of Chemistry , Faculty of Science , Hokkaido University , Sapporo 060-0810 , Japan . Email: sawamura@sci.hokudai.ac.jp; b Institute of Quantum Beam Science , Ibaraki University , Mito , Ibaraki 310-8512 , Japan . Email: seiji.mori.compchem@vc.ibaraki.ac.jp; c Division of Pharmaceutical Sciences , Graduate School of Medical Sciences , Kanazawa University , Kakuma-machi , Kanazawa 920-1192 , Japan

## Abstract

Copper-catalyzed asymmetric direct alkynylation of α-ketoesters with terminal alkynes with chiral prolinol–phosphine ligands, most preferably (α*R*,2*S*)-1-(2-dicyclohexylphosphinobenzyl)-α-neopentyl-2-pyrrolidinemethanol, afforded various enantioenriched chiral propargylic tertiary alcohols. Quantum-chemical calculations using the BP86 density functional including Grimme's empirical dispersion correction [DF-BP86-D3(BJ)-PCM(*t*BuOH)/TZVPP//DF-BP86-D3(BJ)/SVP] show the occurrence of OH···O/sp^3^-CH···O two-point hydrogen bonding between the chiral ligand and the carbonyl group of the ketoester in the stereo-determining transition states. Combined with the hydrogen-bonding interactions orienting the ketoester substrate, dispersive attractions between the chiral ligand (*P*-cyclohexyl groups) and the ketoester in the favored transition states, rather than steric repulsions in the disfavored transition state explain the enantioselectivity of the asymmetric copper catalysis.

## Abstract 2

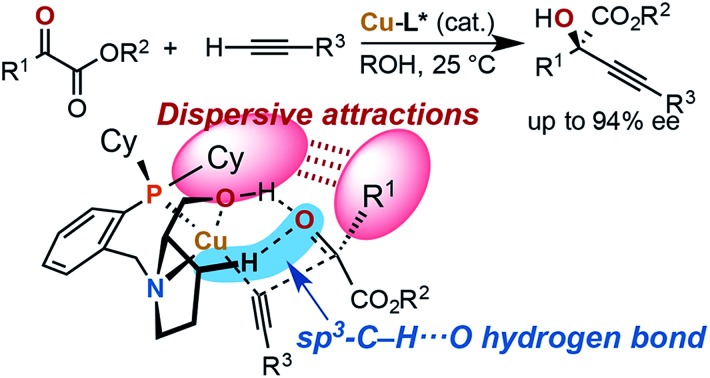
A chiral copper catalyst selects enantiofaces by assembled attractive interactions.

## Introduction

**Scheme 1:**
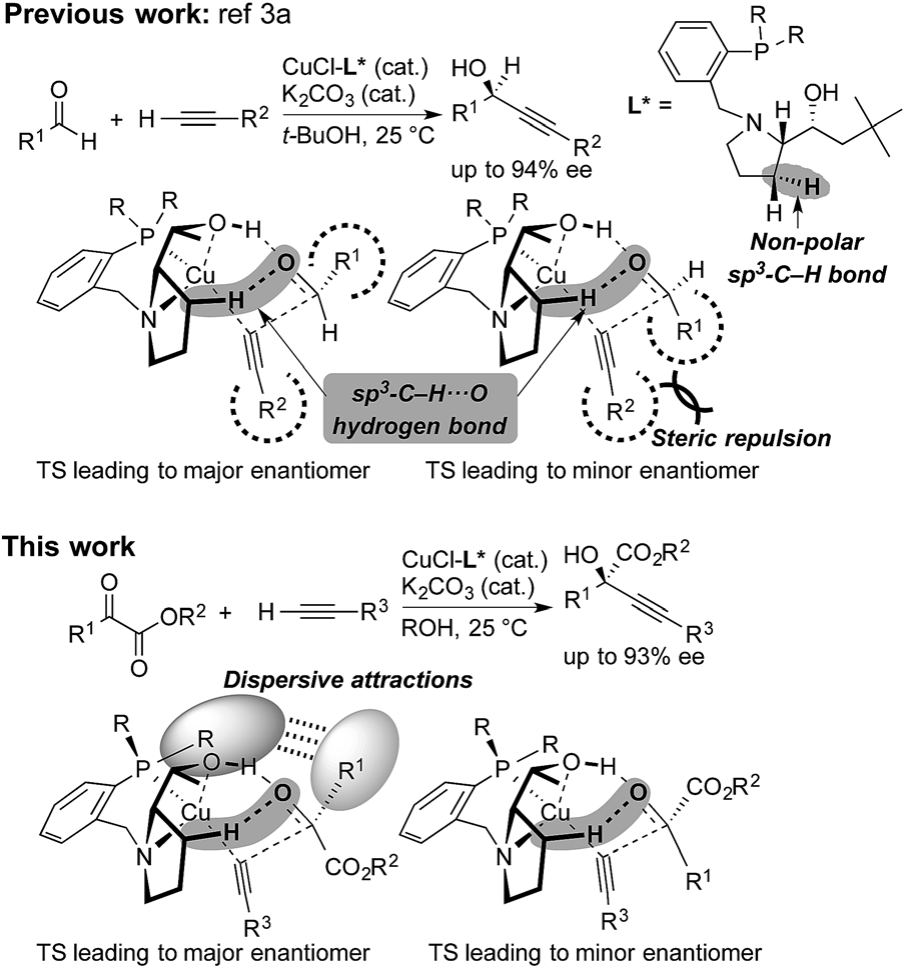
Copper-catalyzed enantioselective alkynylation of carbonyl compounds with chiral prolinol–phosphine ligands. Comparison between the reaction of aldehydes (ref. 3a) and α-ketoesters (this work). Non-classical hydrogen bonding with non-polar sp^3^-C–H bonds, steric repulsion, and dispersive attractions are highlighted.

Steric strain also called steric repulsion between catalysts and substrates plays an important role in enantioselective catalysis, while catalyst design utilizing catalyst–substrate secondary attractive interactions such as electrostatic interactions, hydrogen bondings, π/π stackings and C–H/π interactions may produce advanced concepts.^1^ In this regard, enantiocontrol without using catalyst–substrate steric strain has rarely been elucidated, but it should be more generally explored.^2^ Our previous study on the copper-catalyzed asymmetric direct alkynylation of aldehydes introduced a series of chiral prolinol–phosphine ligands (Scheme 1).^3^,^4^ Density functional theory (DFT) calculations indicated the occurrence of two-point hydrogen bonding comprising OH···O and non-classical sp^3^-CH···O hydrogen bonds,^5^–^7^ which orient the carbonyl group of the prochiral aldehyde. We deduced the enantioselectivity to be due to a steric repulsion between the substituents of the aldehyde (R^1^) and the alkyne (R^2^) in the reaction pathway leading to the minor enantiomer.

Herein, we report that the copper-catalyzed asymmetric direct alkynylation of α-ketoesters with chiral prolinol–phosphine ligands occurred with a high level of enantioselectivity through a discrimination of two ketoic carbonyl substituents, R^1^ and CO_2_R^2^, by the chiral catalyst. DFT calculations including Grimme's empirical dispersion correction^8^ indicated that steric repulsions between the catalyst and the substrates do not play a major role, but the enantioselectivity is determined by assembled attractive catalyst–substrate interactions. Namely, in addition to a two-point hydrogen bonding involving non-classical sp^3^-CH···O hydrogen bonding, dispersive attractions^9^ occur between the chiral ligand and the substrates to allow steric-strain-free enantioselection.

From the viewpoint of organic synthesis, catalytic enantioselective direct alkynylation of carbonyl compounds with terminal alkynes is a straightforward and atom-economical strategy for accessing enantioenriched propargylic alcohols, which are versatile building blocks for the asymmetric synthesis of more complex organic molecules.^10^ Substantial progress has been made in the alkynylation of aldehydes, affording chiral secondary propargylic alcohols through the invention of various efficient chiral catalyst systems with different metals such as Zn,^11^ In,^12^ Cu,^3^ and Ru.^13^ However, the synthesis of chiral tertiary propargylic alcohols through the corresponding reaction of ketones is still challenging. As for the reaction of activated ketones, nevertheless, there are limited examples that reported reasonable catalytic activities and high enantioselectivities.^14^–^17^ For instance, Jiang and co-workers achieved high enantioselectivities in the reaction of α-ketoesters through a modification of Carreira's Zn–β-aminoalcohol catalyst system.^14^ However, high enantioselectivities were achieved only with a stoichiometric amount of the chiral catalyst or under catalytic (5.5–20 mol%) conditions utilizing excess alkynes as solvents with a limited substrate scope. Oshima, Mashima, and co-workers introduced new Rh–Phebox catalysts to achieve high enantioselectivities for the reaction of trifluoropyruvates, and Song, Gong, and co-workers later introduced a similar Rh catalyst system.^15^ Shibasaki, Kanai, and co-worker reported moderate enantioselectivities in the Cu-catalyzed reaction between trifluoroacetophenone and phenylacetylene.^16^ Recently, Meggers and co-workers reported high enantioselectivities with a broader scope of trifluoromethyl aryl ketones in the studies on ruthenium complexes with metal-centered chirality.^17^ Thus, a chiral catalyst system allowing high enantioselectivity with a broad substrate scope is awaited, while excellent catalyst systems have been developed specifically for trifluoromethyl ketones.^15^,^17^

## Results and discussion

### Optimization

Initial experiments to find suitable reaction conditions were conducted for the reaction between methyl 2-phenylglyoxylate (**1a**, 0.2 mmol) and phenylacetylene (**2a**, 1.2 eq.) in the presence of CuCl (10 mol%), K_2_CO_3_ (30 mol%), and different phosphine ligands (**L1–8**) at 25 °C over 48 h (Table 1). The reaction with the prototype prolinol–phosphine chiral ligand **L1**, which consists of triphenylphosphine and a simple prolinol linked with each other by a methylene group, occurred cleanly to give the corresponding tertiary propargylic alcohol (**3aa**) in a high yield (92% yield after isolation) with a moderate enantioselectivity (56% ee) in favor of the *R* configuration (entry 1). The secondary (**L2**) or tertiary (**L3**) alcohol type ligands, which have one or two methyl groups at the position α to the hydroxyl group, gave slightly better enantioselectivities (58% and 67% ee), but the reaction occurred more slowly and the yield dropped to a moderate level (entries 2 and 3). Neopentyl-substituted ligand **L4**, which is the optimal ligand for the reaction of aliphatic aldehydes,3*a* was only comparable with the tertiary alcohol ligand (**L3**) concerning both product yield and enantioselectivity (71% yield and 67% ee) (entry 4). Thus, the ligand modification at the alcohol moiety was not fruitful. In contrast, the modification of the *P*-substituents had a significant impact. The introduction of electron-donating MeO groups (**L5**) at the *para*-position of the two *P*-phenyl groups caused a dramatic increase in the product yield (96%) with a slight improvement of the enantioselectivity (70% ee) as compared with the results with the parent Ph_2_P-type ligand (**L4**), while the substitutions with electron-withdrawing F atoms (**L6**) at the *para*-positions were unfavorable (entries 5 and 6). Finally, our ligand screening led to identification of the Cy_2_P-type ligand (**L7**) with a neopentyl substituent at the alcohol moiety as the most suitable. With **L7**, the reaction occurred quantitatively (97% yield) with enantioselectivity as high as 88% ee (entry 7). The corresponding experiment without using a glove box gave an essentially identical result concerning both the product yield and enantioselectivity (entry 8).

**Table 1:** Copper-catalyzed enantioselective alkynylation of **1a** with **2a** under various conditions

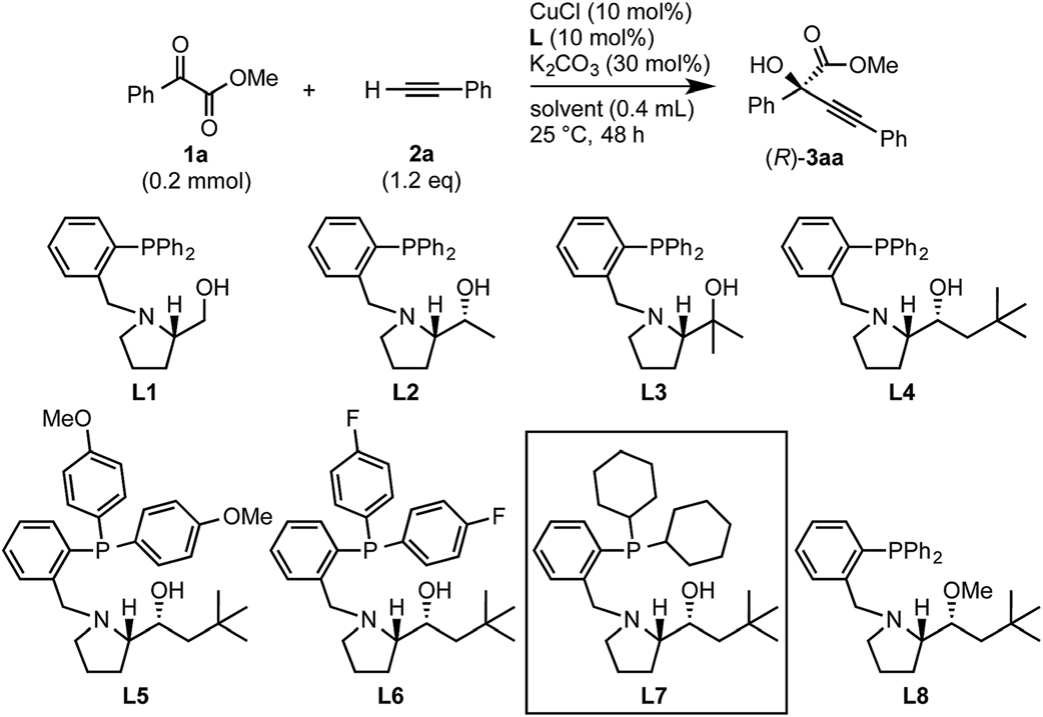				
Entry	Ligand	Solvent	Yield^*a*^, %	ee^*b*^, %
1	**L1**	*t*-BuOH	92	56
2	**L2**	*t*-BuOH	67	58
3	**L3**	*t*-BuOH	68	67
4	**L4**	*t*-BuOH	71	67
5	**L5**	*t*-BuOH	96	70
6	**L6**	*t*-BuOH	57	57
7	**L7**	*t*-BuOH	**97**	**88**
8^*c*^	**L7**	*t*-BuOH	98	88
9	**L7**	THF	26	74
10	**L7**	Dioxane	31	77
11	**L7**	MeCN	35	78
12	**L8**	*t*-BuOH	0	—

^*a*^Yield of the isolated product (silica gel chromatography).

^*b*^Determined by HPLC analysis.

^*c*^Experiment conducted without using a glove box.

The nature of the solvent had a strong impact on the yield and enantioselection (Table 1, entries 9–11). The use of aprotic solvents such as THF, dioxane or CH_3_CN in place of the protic solvent *t*-BuOH for the reaction with **L7** caused significant decreases in the product yields (26%, 31% and 35%) and enantioselectivities (74%, 77% and 78% ee). The protection of the hydroxy group in **L4** as a methyl ether (**L8**) inhibited the reaction completely (entry 12). Thus, favorable effects of the protic nature of the solvent and the critical role of the alcoholic site in the prolinol–phosphine ligand were confirmed like in our previous study on the asymmetric alkynylation of aldehydes.3*a*

### Scope of ketoesters

Various α-ketoester derivatives were subjected to the reaction with phenylacetylene (**2a**) with the Cu–**L7** catalyst system in *t*-BuOH or i-PrOH (Table 2). The isopropyl or *tert*-butyl 2-phenylglyoxylates (**1b** and **1c**) also served as substrates, and the comparison of the results with that with the methyl ester (**1a**) showed an increase in the enantioselectivity with the increase in the steric demands of the ester moieties (entries 1 and 2). 2-Hydroxyethyl ester (**1d**) was also a suitable substrate (entry 3).

**Table 2:** Copper-catalyzed enantioselective alkynylation of various α-ketoesters (**1**) with **2a**

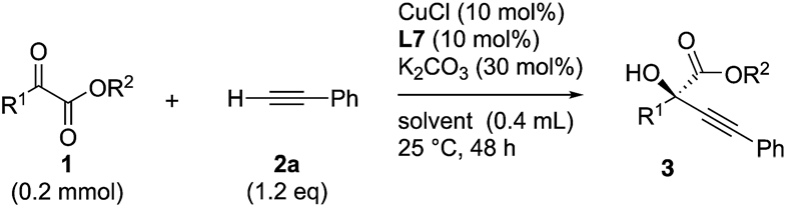					
Entry	Ketoester **1**	Propargylic alcohol **3**	Solvent	Yield^*a*^, %	ee^*b*^, %
1^*c*^	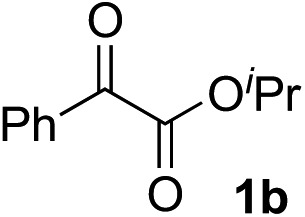	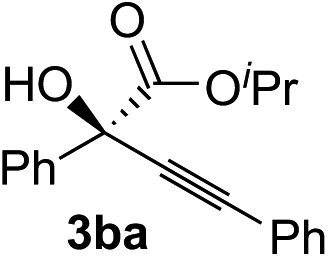	*t*-BuOH	99	90
2^*c*^	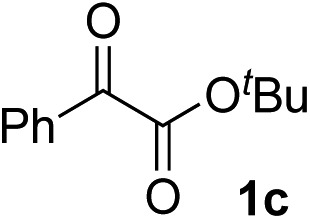	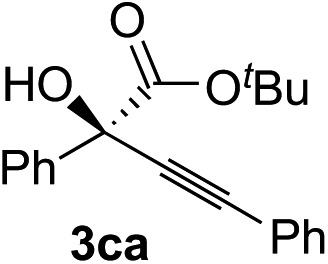	i-PrOH	80	92
3^*c*^	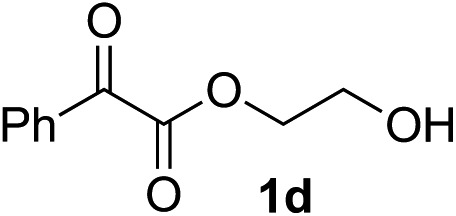	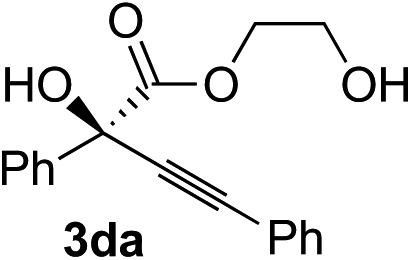	*t*-BuOH	48	90
4	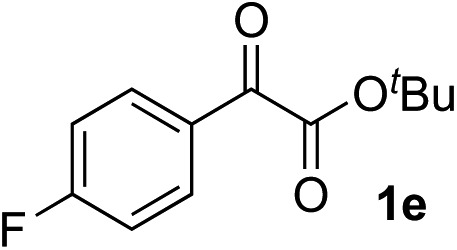	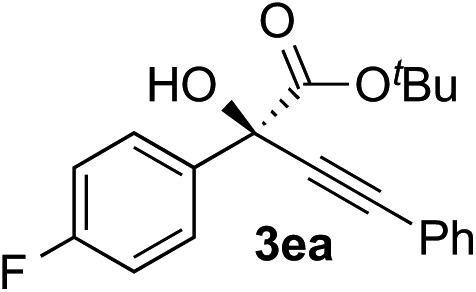	i-PrOH	98	89
5	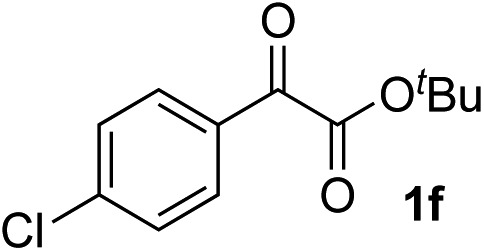	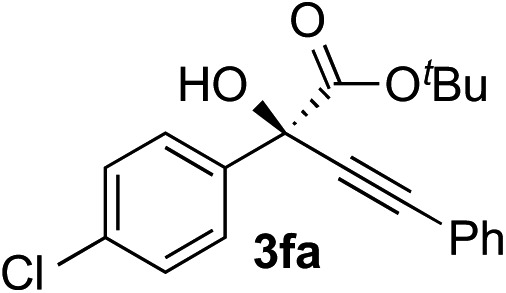	i-PrOH	97	92
6	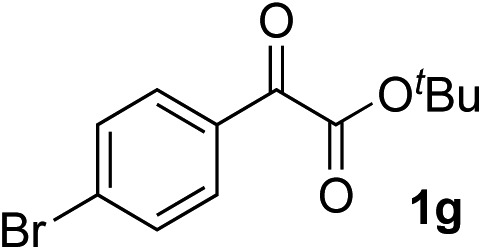	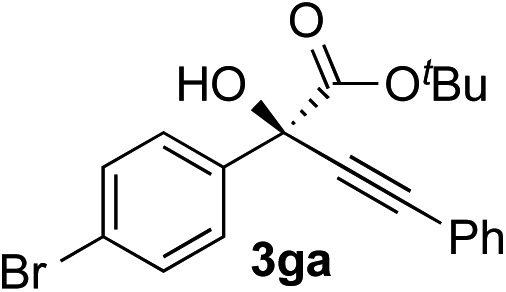	i-PrOH	97	92
7^*d*^	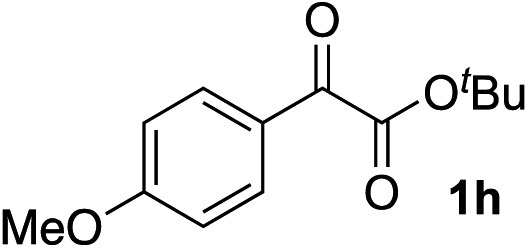	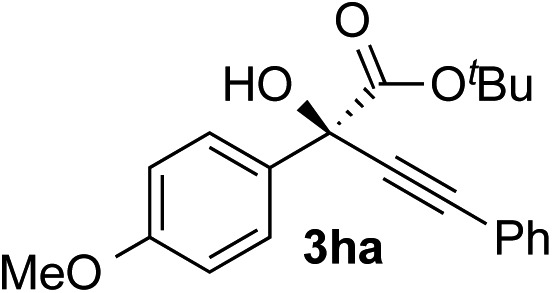	i-PrOH	30	91
8	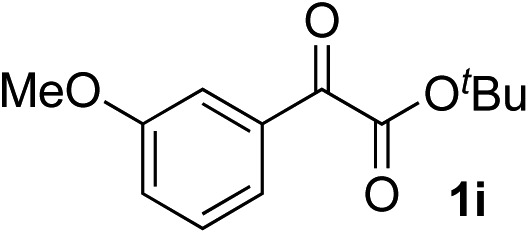	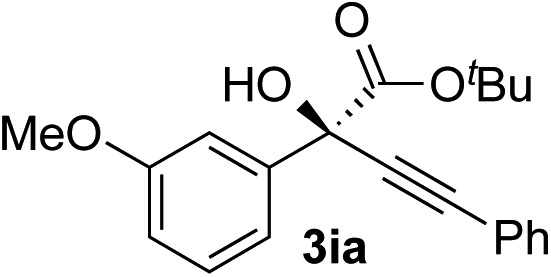	i-PrOH	97	93
9^*d*^	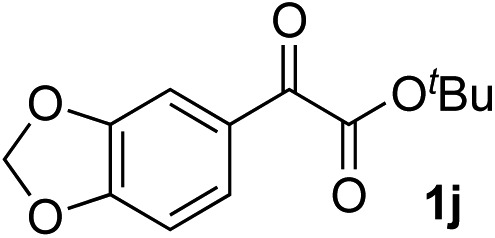	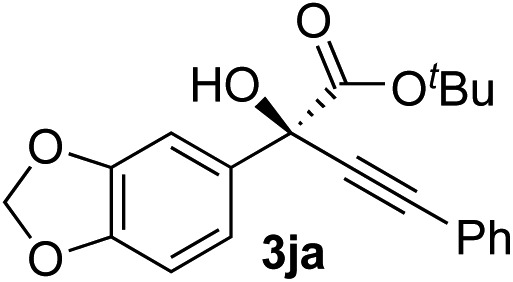	i-PrOH	84	93
10	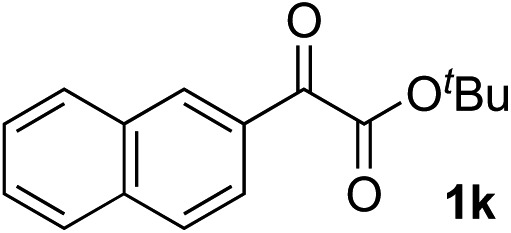	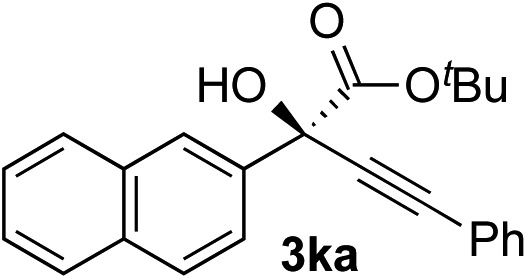	i-PrOH	92	94
11	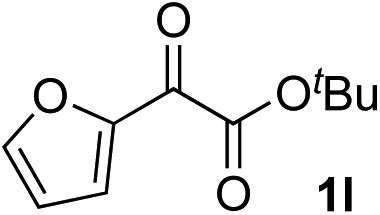	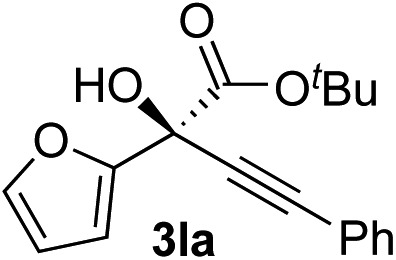	i-PrOH	64	66
12	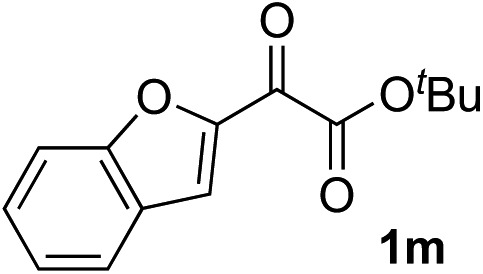	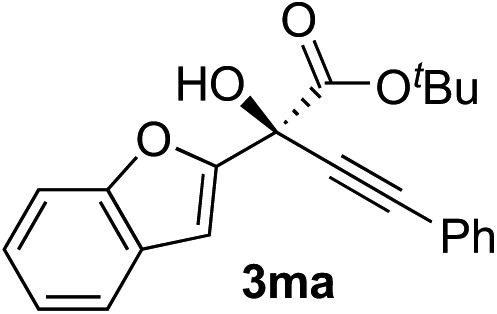	i-PrOH	93	69
13	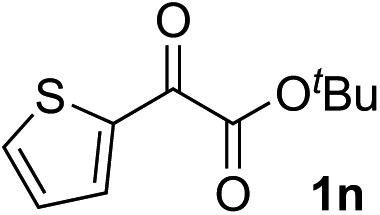	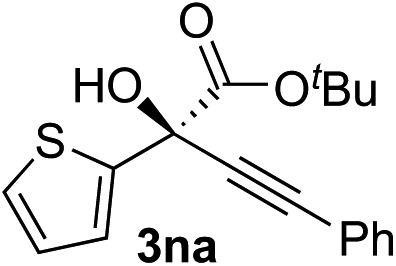	i-PrOH	97	84
14	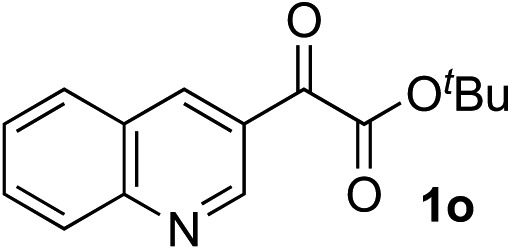	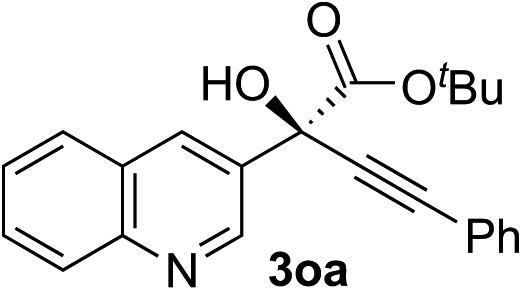	i-PrOH	91	93
15	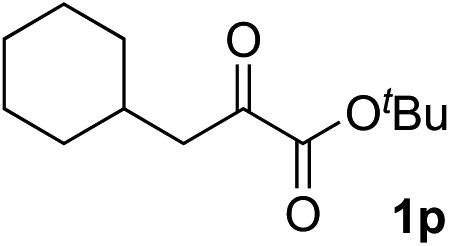	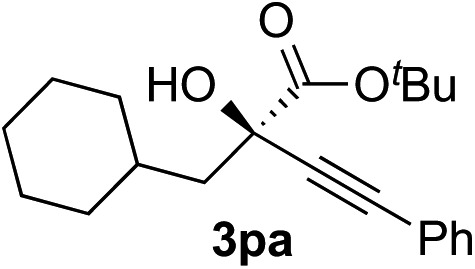	i-PrOH	85	90
16	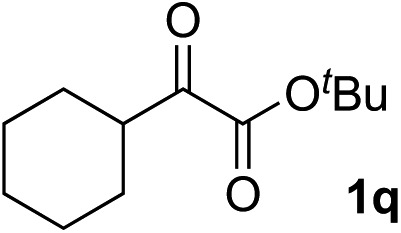	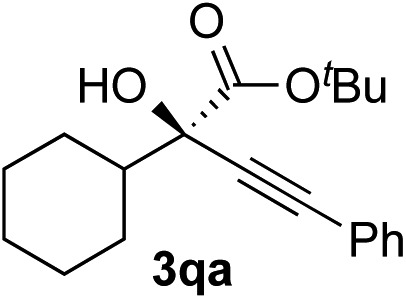	i-PrOH	82	86
17^*e*^	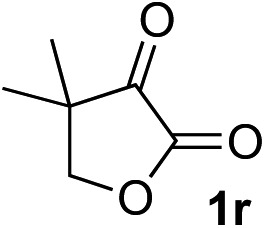	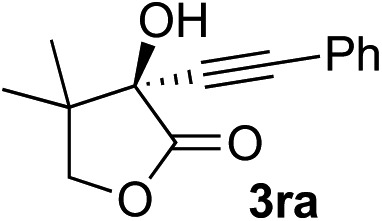	i-PrOH	99	90

^*a*^Yield of the isolated product (silica gel chromatography).

^*b*^Determined by HPLC analysis.

^*c*^The reaction was carried out in *t*-BuOH.

^*d*^The reaction was carried out for 72 h.

^*e*^The reaction was carried out at –20 °C.

Clean and highly enantioselective reactions occurred with 2-phenylglyoxylate derivatives with different halogen substituents (F: **1e**, Cl: **1f**, Br: **1g**) at the *para*-position of the aromatic ring (Table 3, entries 4–6). While a MeO substituent at the *para* position (**1h**) retarded the reaction, *m*-MeO (**1i**) and 3,4-methylenedioxy-substituted (**1j**) 2-phenylglyoxylates reacted more smoothly to give the corresponding products in high yields with high enantioselectivities (entries 7–9). Unfortunately, *tert*-butyl 2-(*o*-tolyl)glyoxylate is not reactive even at 40 °C. Steric effects of the substrate may have hampered the reaction. 2-Naphthylglyoxylate (**1k**) underwent a clean reaction with an enantioselectivity as high as 94% ee (entry 10). The reactions of five-membered heteroaromatic α-ketoesters with furan (**1l**), benzofuran (**1m**), or thiophene (**1n**) substituents were somewhat less enantioselective than those of the 2-phenylglyoxylate derivatives, but gave the corresponding tertiary heteroarylcarbinols with enantiomeric purities in a range of 66–84% ee (entries 11–13). The reaction of 3-quinolylglyoxylate (**1o**) occurred with a high yield (91%) and a high enantioselectivity (93% ee) (entry 14). The aliphatic α-ketoesters (**1p** and **1q**) with a branched alkyl group at the 2-position were also favorable substrates (entries 15 and 16). The reaction of the cyclic ketoester ketopantolactone (**1r**) occurred at –20 °C with an enantioselectivity of 90% ee (entry 17).

**Table 3:** Copper-catalyzed enantioselective alkynylation of α-ketoesters (**1**) with various alkynes (**2**)

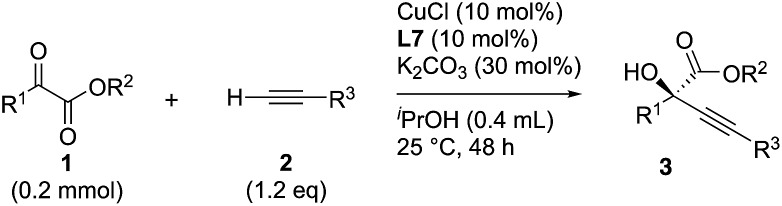					
Entry	Ketoester **1**	Alkyne **2**	Propargylic alcohol **3**	Yield^*a*^, %	ee^*b*^, %
1	**1c**	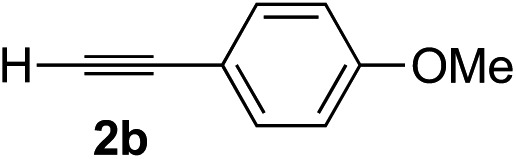	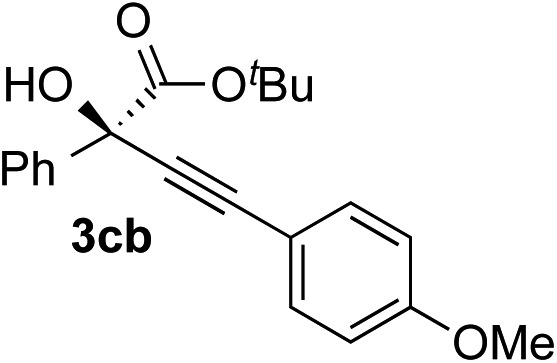	97	93
2	**1c**	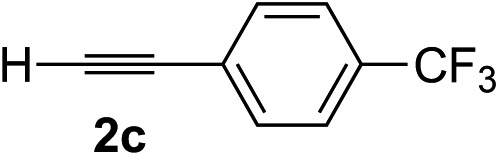	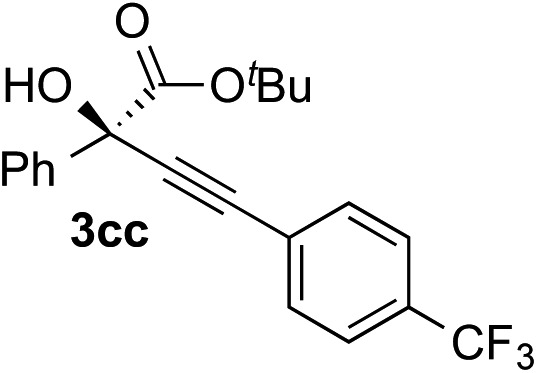	70	88
3	**1c**	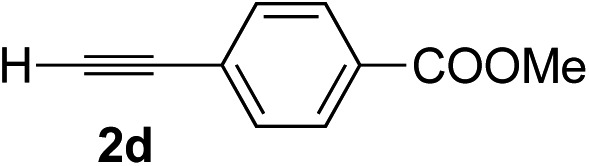	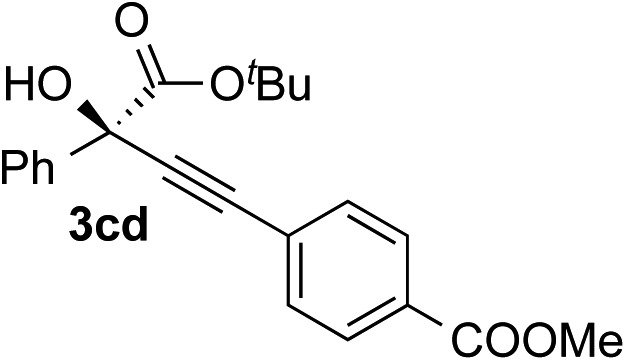	68	85
4	**1c**	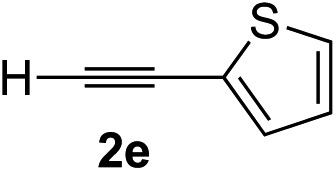	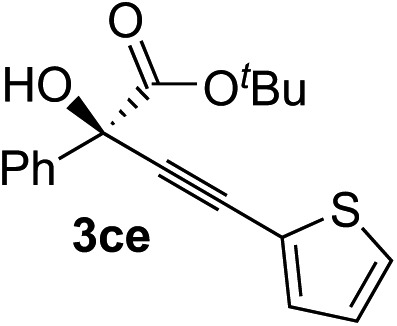	96	90
5	**1c**	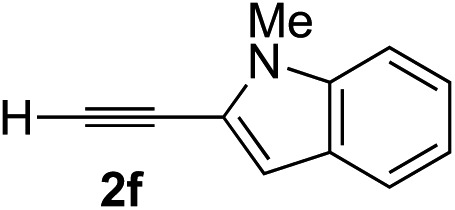	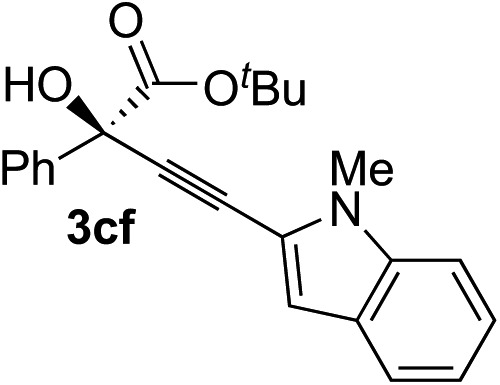	84	71
6	**1c**	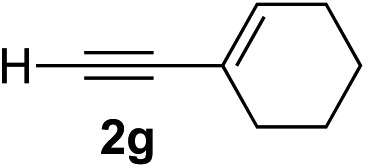	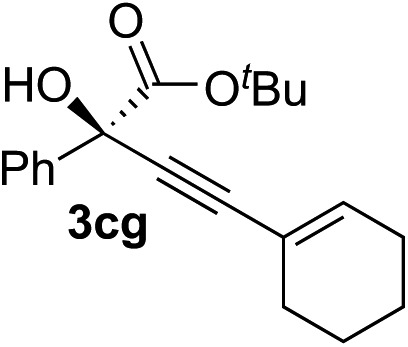	87	90
7	**1c**	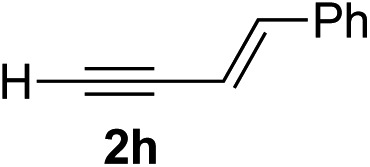	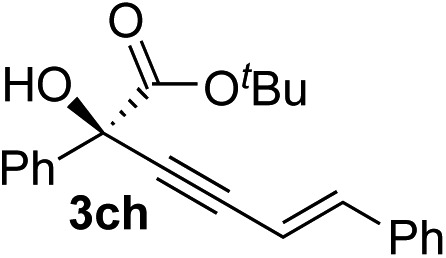	83	87
8	**1f**	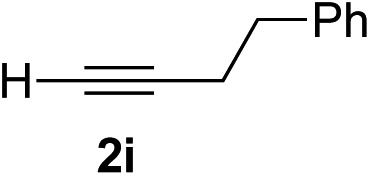	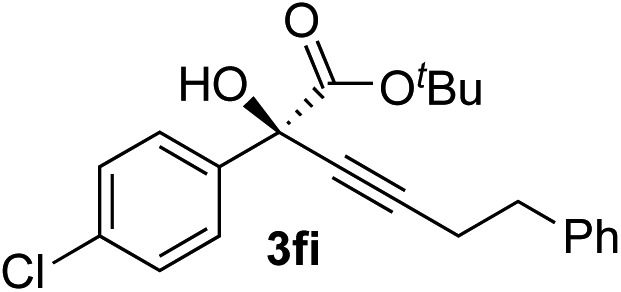	87	87
9	**1f**	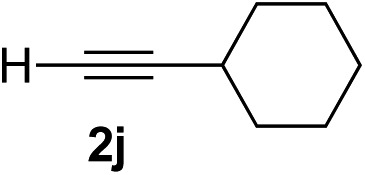	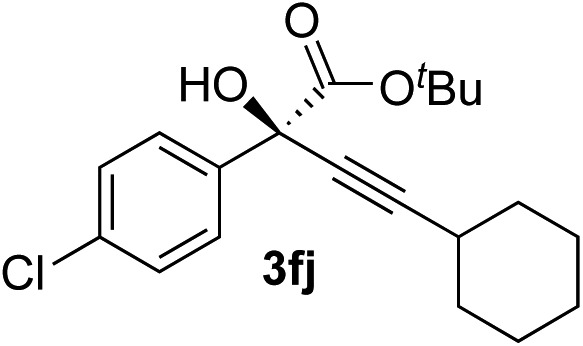	93	90
10	**1g**	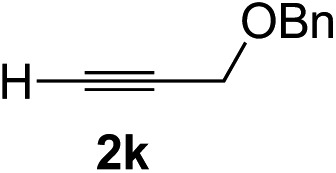	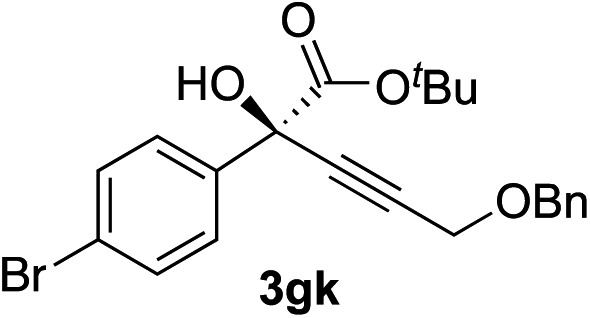	92	77
11	**1g**	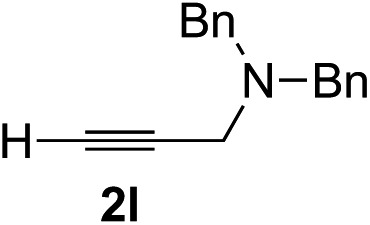	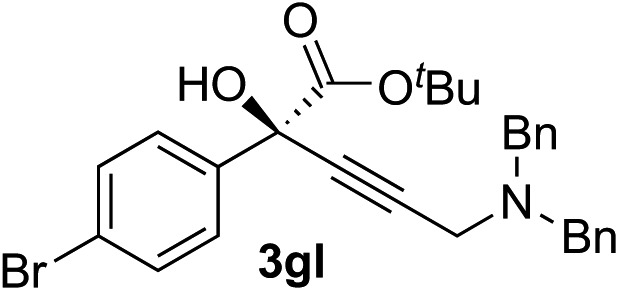	94	71
12^*c*^	**1r**	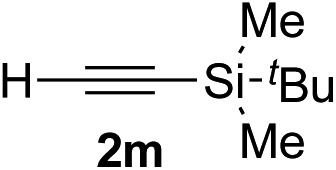	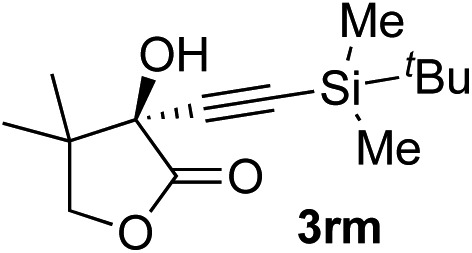	95	76

^*a*^Yield of the isolated product (silica gel chromatography).

^*b*^Determined by HPLC analysis.

^*c*^The reaction was carried out at 0 °C for 24 h.

Overall, the protocol with the Cu–**L7** catalyst system in *t*-BuOH or i-PrOH is applicable to a range of α-ketoesters including 2-(hetero)arylglyoxylates and 2-alkylglyoxylates. However, *tert*-butyl pyruvate did not react with phenylacetylene (**2a**) but gave a mixture of self-condensation products. The reaction between *tert*-butyl trifluoropyruvate and **2a** resulted in the decomposition of the ketoester without forming the desired alkynylation product.

### Scope of alkynes

Various enantioenriched tertiary propargylic alcohols with different substituents at the alkyne terminus were obtained (Table 3). The aromatic alkyne (**2b**) with an electron-donating methoxy substituent reacted with high product yield and enantioselectivity (entry 1). On the other hand, the substitution of the aromatic ring with the electron-withdrawing trifluoromethyl or methoxy carbonyl groups resulted in decreases in the yield and enantioselectivity (entries 2 and 3). The sulfur- or nitrogen-containing heteroaromatic groups were acceptable as substituents of the alkyne substrate (entries 4 and 5). The cyclic and acyclic 1,3-enyne derivatives (**2g** and **2h**) afforded the corresponding conjugated propargylic alcohols (**3cg** and **3ch**) (entries 6 and 7). Alkylacetylenes were also suitable substrates (entries 8–11). The reaction of linear alkylacetylene **2i** with **1f** proceeded with reasonably high enantioselectivity (entry 8). The α-branched aliphatic alkyne **2j** reacted with high yield and enantioselectivity (entry 9). Propargyl ether **2k** and propargylamine **2l** also participated in the reaction, albeit with moderate yields and enantioselectivities (entries 10 and 11). The *tert*-butyldimethylsilylacetylene **2m** underwent a clean reaction with a moderate enantioselectivity (entry 12). No reaction occurred with *tert*-butylacetylene and triisopropylsilylacetylene even at higher temperatures.

Overall, various terminal alkynes, such as phenylacetylene derivatives, conjugated enynes, linear or α-branched alkylacetylenes, protected propargyl alcohol, or amine derivatives, and *tert*-butyldimethylsilylacetylene, were acceptable substrates. However, the substituent of the alkynes had no small effect on reactivity and enantioselectivity. Furthermore, it should be noted that the reactivity and selectivity profile depending on the alkynes is significantly different between the alkynylation of aldehydes and that of ketoesters. Namely, the reaction of ketoesters is more sensitive to the steric and electronic effects in the alkyne. In particular, the nonreactiveness of *tert*-butylacetylene and triisopropylsilylacetylene is in sharp contrast to the results of the alkynylation of aldehydes.3*a* In the latter, bulky triisopropylsilylacetylene was the most favorable for enantiocontrol with a broad scope of the aldehyde.

### Quantum-chemical studies

The direct alkynylation of ketoesters with terminal alkynes exhibits similar behavior towards ligands and solvents to the previously studied reaction of aldehydes under comparable conditions.3*a* The similarity of the substrates and the reaction conditions suggest that the mechanisms are analogous. The hydroxyl group of the chiral prolinol–phosphine ligand is a key element in the catalytic activity and in the enantioselectivity by forming a highly directional hydrogen bond with the carbonyl oxygen. Additionally supporting this coordination is a non-classical sp^3^-CH···O hydrogen bond originating from the pyrrolidine moiety of the ligand, which has enough flexibility to bend inwards to the reactive center to allow this interaction.

**Scheme 2:**
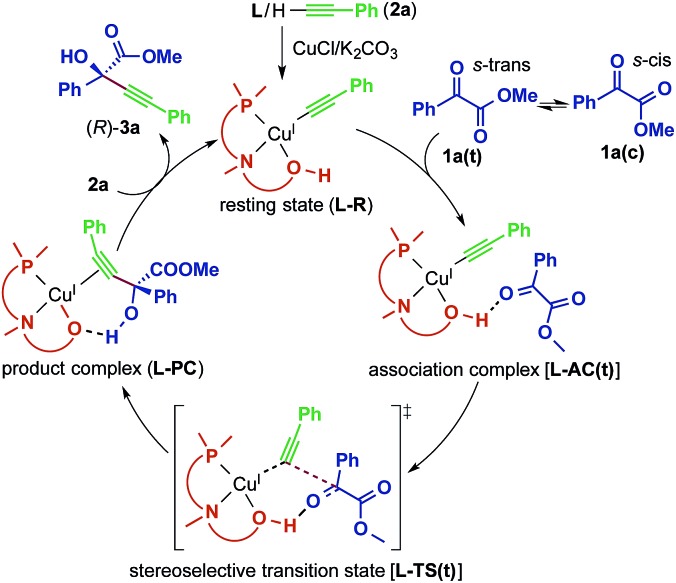
Proposed catalytic reaction pathway for the reaction between **1a** and **2a** catalyzed by the Cu–**L** system.

A proposed catalytic reaction pathway is shown in Scheme 2. The reaction starts with the formation of the (η^1^-alkynyl)copper(i) complex (**R**), which is also the resting state, through the association of the ligand, the metal center, and the deprotonated alkyne (**2**–H^+^). The ketoester (**1**) coordinates *via* the carbonyl oxygen to the hydroxy group of the ligand, bringing the reacting carbon atoms in proximity. This association complex (**AC**) is the precursor for the stereoselective carbon–carbon bond formation, which leads to the product complex (**PC**) in which the tertiary propargylic alcohol (**3**) is bound *via* the π-bonds to the copper center. Exchange with the substrate alkyne (**2**) regenerates the resting state (**R**) and therefore completes the catalytic cycle.

To further elucidate the origin of the enantioselectivity of the reaction, quantum-chemical calculations based on the transition states of the aldehyde reaction have been performed. Full geometry optimizations using the BP86 density functional^18^ including Grimme's empirical dispersion correction (DFTD3 with Becke–Johnson damping)^8^ in conjunction with the def2-SVP basis set^19^ have been carried out with the Gaussian 09 program suite.^20^ Density fitting has been employed to speed up the calculations.19c,^21^ This level of theory is denoted as DF-BP86-D3(BJ)/SVP. Normal coordinate analysis has been performed to confirm convergence towards stationary points and to estimate thermal corrections at 298.15 K and 1 atm. Calculations following the intrinsic reaction coordinates (IRCs) from first-order saddle points (transition states) to local minima (reactants and intermediates) have been used to describe the reaction pathways (see ESI† for details).^22^ To gain a better understanding of the energetics of this system, single-point calculations have been carried out on the converged geometries using the larger basis set def2-TZVPP,^19^ and estimates for the solvent (polarizable continuum model, PCM, with *tert*-butanol *ε* = 12.47).^23^ These energy values are discussed throughout the paper, and this level is denoted as DF-BP86-D3(BJ)-PCM(*t*BuOH)/TZVPP//DF-BP86-D3(BJ)/SVP. Relative Gibbs energies (electronic energies in the ESI†) in kcal mol^–1^ for the calculated reaction pathways of the model system (Table 1, entry 2) are summarized in Table 4.

**Table 4:** Relative Gibbs energies (electronic energies in the ESI) in kcal mol^–1^ for the calculated reaction pathways of the model system (Table 1, entry 2)^*a*^

	*R*-paths		*S*-paths	
	**1a(t)**	**1a(c)**	**1a(t)**	**1a(c)**
**R** + **1a**	9.7	12.0	9.7	12.0
**L2-AC**	3.3	0.0	3.7	2.6
**L2-TS**	9.3	8.7	9.6	12.0
**L2-PC**	–7.9	–9.0	–5.9	–7.4
Population (%)	24.5	62.0	13.2	0.2

^*a*^Gaussian 09, DF-BP86-D3(BJ)-PCM(*t*BuOH)/TZVPP//DF-BP86-D3(BJ)/SVP, 298.15 K, 1 atm.

For the initial computations, the system of **1a**, **2a** and **L2** has been chosen, since phenyl moieties have a small conformational space. To explain the selectivity of the reaction, it is sufficient to calculate the transition state of the C–C bond formation (**L2-TS**) and the connected intermediates (**L2-AC** and **L2-PC**). The α-ketoester has two different conformations (*s-cis* and *s-trans*) caused by rotation along the single bond connecting the two carbonyl groups (Scheme 2). The *s-cis* conformer [**1a(c)**] is about 2.3 kcal mol^–1^ higher in energy than the *s-trans* conformer [**1a(t)**] (Table 4, **R** + **1a**). Since the activation energies of all reaction pathways are larger than this, the rotation becomes unhindered and both rotamers have to be considered for the evaluation of the reaction mechanism. This is also reflected in the relative energies of the transition states leading to the *R* product (**L2-TS-R**), which are lower in energy than the corresponding *S* pathways (**L2-TS-S**). The *R*-stereochemistry of the product **3aa** is well reproduced by the calculations, and based on the four reaction pathways, the overall enantiomeric excess is estimated to be 73.1%.

**Fig. 1:**
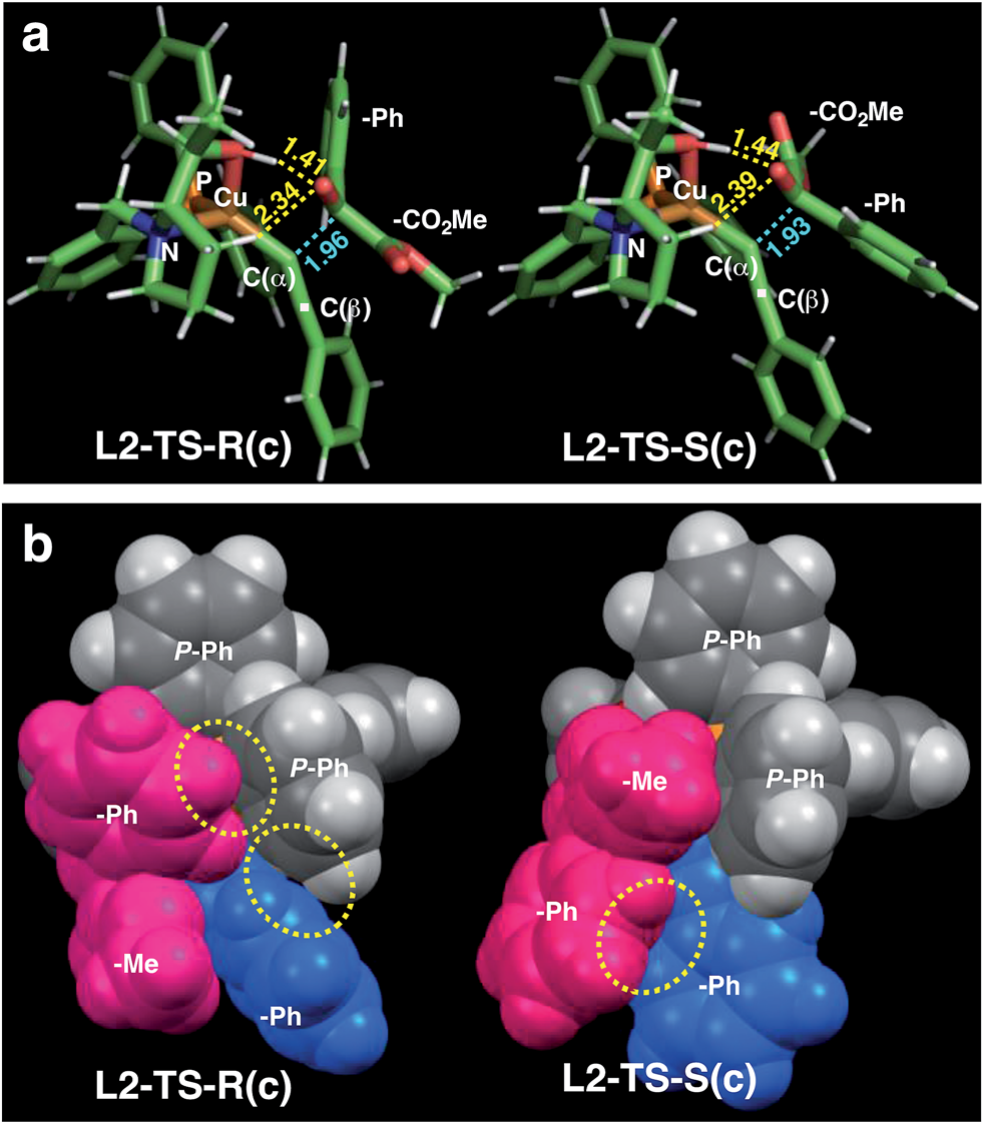
Comparison of the transition state structures leading to the respective *R* [left, **L2-TS-R(c)**] or *S* [right, **L2-TS-S(c)**] product complexes with **L2** and **1a** in the *s-cis* conformation (Table 4). (a) Stick models showing a developing C–C bond (blue dotted line). Atomic distances (in angstrom) of the OH···O/CH···O two-point hydrogen bonds are shown in yellow dotted lines. (b) Space-filling models highlighting dispersive substrate–ligand interactions (yellow dotted circles). Red: **1a**; blue: acetylide moiety.

**Fig. 2:**
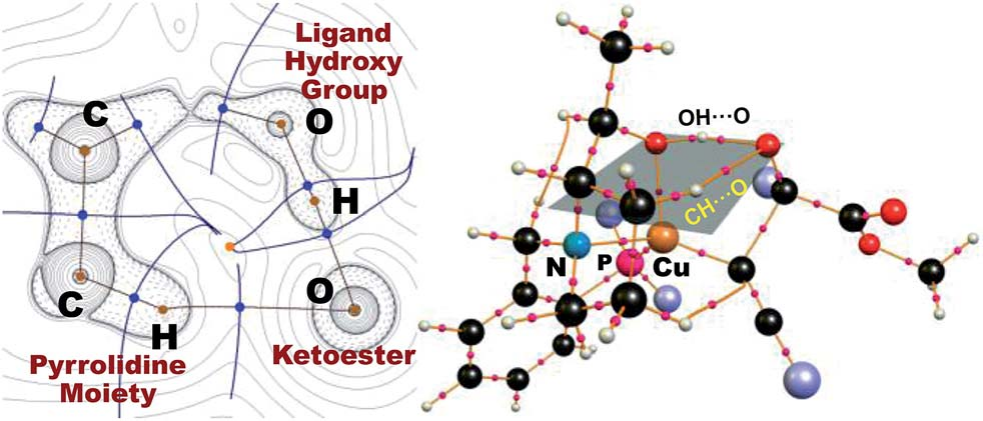
Laplacian of the electron density of the transition state (left) leading to the *R* product complex with **1a** in the *s-cis* conformation [**L2-TS-R(c)**] in the sp^3^-C–H···O···H–O plane, which is indicated on the right. Bond critical points (BCPs) are indicated with blue dots (left) and pink spheres (right), with the corresponding bond paths in light brown. Dotted lines mark areas of charge accumulation and solid lines represent areas of charge depletion. Solid blue lines correspond to zero-flux surfaces, and the orange dot represents a ring critical bond. The four terminal phenyl groups are shown as light-purple spheres for clarity (right).

As in the aldehyde system,3*a* a non-classical hydrogen bond between the sp^3^-C–H bond in the pyrrolidine ring and the carbonyl oxygen of the ketoester (**1a**) (sp^3^-CH···O) is preserved in all optimized transition states, in addition to a normal hydrogen bond donated by the copper-bound hydroxyl group, resulting in directional two-point hydrogen-bonding, which orients the ketoester (**1a**) in a well-defined manner (Fig. 1a). The quantum theory of atoms in molecules (QTAIM)^24^,^25^ allows one to qualitatively estimate the strengths of the classical OH···O bond as well as the non-classical sp^3^-CH···O interaction (Fig. 2). Since the strength of hydrogen bonds is not accessible experimentally, caution should be applied regarding the calculated absolute values (see ESI† for more information). The value of the potential energy density at the bond critical point is proportional to the strength of the hydrogen bond.25*c* This value indicates a rather strong classical OH···O bond motif, while the non-classical sp^3^-CH···O interaction is less than a tenth of that. This is in line with the previous analysis based on the distances of the respective interactions.3*a*

As visualized in the space-filling models in Fig. 1b, the stereo-discrimination by the catalyst (Cu–**L2**) is due to the dispersive attractions between the phenyl moiety of the ketoester (red-coloured) and the phenyl groups of the phosphine moiety (grey) through partial π-stacking in **L2-TS-R**,^8^ as opposed to **L2-TS-S** where these moieties are oriented away from each other. Additionally, in the *R* path the phenyl moiety of the alkyne (blue-coloured) can partially stack with one of the *P*-phenyl groups of **L2** (grey). In the *S*-path, this π-stacking is not possible. Instead, the phenyl moiety of the alkyne is in contact with the phenyl group of the ketoester. These non-covalent interactions can be further studied and visualised by analysing the electron density and its derivatives (see ESI† for details).^26^ These analyses also reveal the importance of dispersive effects for the sp^3^-CH···O interactions, which are similar to classical hydrogen bonds in most respects, but generally weaker. One difference is that the donating CH group is weakly polarized, which makes the isotropic effects more relevant, while the magnitude of the electrostatic component loses some significance.^5^ The classical OH···O bond motifs, on the other hand, are already too strong to register in the analyses within the chosen cut-off parameters. Overall, the dispersive attractions are stronger in **L2-TS-R** than in **L2-TS-S**.

**Fig. 3:**
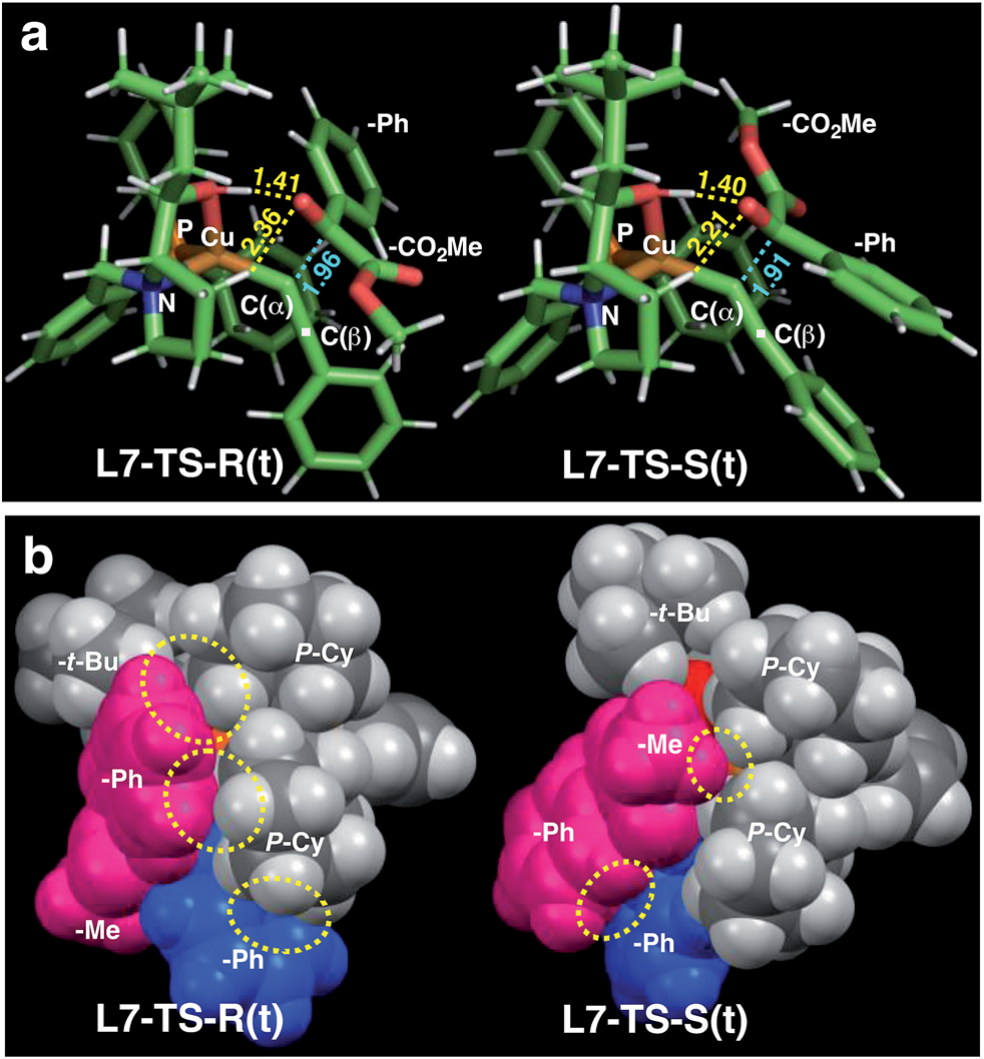
Comparison of the transition state structures leading to the respective *R* [left, **L7-TS-R(t)**] or *S* [right, **L7-TS-S(t)**] product complexes with **L7** and **1a** in the *s-trans* conformation (Table 5). (a) Stick models showing a developing C–C bond (blue dotted line). Atomic distances (in angstrom) of the OH···O/CH···O two-point hydrogen bonds are shown by yellow dotted lines. (b) Space-filling models highlighting dispersive substrate–ligand interactions (yellow dotted circles). Red: **1a**; blue: acetylide moiety.

Calculations for a more extended system with the **L7** chiral ligand yield similar conclusions (Table 5 and Fig. 3). The bulkier *P*-cyclohexyl moieties, as well as the inclusion of the neopentyl moiety in **L7** lead to a higher stereoselectivity, and thus the estimated enantiomeric excess is 99.6% (Table 5). The attractive dispersive interactions between the phenyl group of **1a** (red) and the *P*-cyclohexyl substituents (grey) in the *R* transition states are stronger than the analogous interactions of the methyl group of **1a** (red) in the *S* path (Fig. 3b). Even the partial π-stacking between the phenyl moieties of the alkyne (blue) and the ketoester (red) in **L7-TS-S** cannot counteract this trend.^26^ Non-covalent interactions (cyclohexyl···cyclohexyl) also play an important role for aliphatic substrates like **1q** (see ESI† for details).

**Table 5:** Relative Gibbs energies (electronic energies in the ESI) in kcal mol^–1^ for the calculated reaction pathways of the model system using **L7** as the ligand (Table 1, entry 7)^*a*^

	*R*-paths		*S*-paths	
	**1a(t)**	**1a(c)**	**1a(t)**	**1a(c)**
**R** + **1a**	10.0	12.3	10.0	12.3
**L7-AC**	0.0	2.6	6.1	5.1
**L7-TS**	9.0	10.2	12.5	15.8
**L7-PC**	–7.1	–7.3	–5.3	–5.4
Population (%)	89.0	10.8	0.2	0.0

^*a*^Gaussian 09, DF-BP86-D3(BJ)-PCM(*t*BuOH)/TZVPP//DF-BP86-D3(BJ)/SVP, 298.15 K, 1 atm.

The neopentyl moiety is too far from the reaction center to induce a change in the conformation of the transition state. Thus, these computations do not explain the decent role of this substituent for better enantioselectivity, while we postulate that it may influence the selectivity by blocking the coordination of the alcohol solvent to the ligand hydroxyl group.

The nearly co-planar arrangement of the PhCO moiety of **1a** in **L2-TS-R** and **L7-TS-R** (Fig. 1 and 2) towards the phosphine substituents implies that the above-mentioned inertness of the 2-(*o*-tolyl)glyoxylate may be due to Ar–CO twisting, which reduces the ligand–substrate dispersive attractions. This twist instead may also cause steric repulsions towards the acetylide moiety, as well as internal strain. Similarly, the relatively low enantioselectivity in the reactions of 2-(2-furyl)glyoxylate (**1l**) (Table 2, entry 11) may be because the furyl ring is too small to have sufficient contact with the ligand *P*-cyclohexyl groups.

For comparison with the previously reported aldehyde system,3*a* a model reaction between cyclohexanecarbaldehyde and trimethylsilylacetylene using ligand **L2** has been optimized to match the level of theory (see ESI† for details). Similar conclusions can be drawn from these calculations: the stereoselectivity is again due to the attractive dispersion interactions of the cyclohexyl moiety in the aldehyde and the *P*-phenyl groups of **L2**, which are present in the *R* paths, but absent in the corresponding *S* paths. In this regard the systems behave almost identically. However, when the alkyne has a substituent of an extreme steric demand as in the case of triisopropylsilylacetylene, which was the most preferable substrate in the reactions with aldehydes, transition states leading to a minor enantiomer will also be destabilized by steric repulsions between the substituent of the aldehyde and the bulky substituent of the alkyne.

## Conclusions

Copper-catalyzed asymmetric direct alkynylation of α-ketoesters with terminal alkynes to produce enantioenriched chiral propargylic tertiary alcohols has been developed by employing a chiral prolinol–phosphine ligand. Various α-ketoesters and terminal alkynes participated in the enantioselective reaction, but extreme steric demands in the alkyne as in *t*-butyl- or triisopropylsilylacetylenes inhibited the reaction. Quantum-chemical calculations show the occurrence of OH···O/sp^3^-CH···O two-point hydrogen bonding between the chiral ligand and the carbonyl group of the ketoester at the stereo-determining transition states. Combined with the hydrogen-bonding interactions orienting the ketoester substrate, dispersive attractions between the chiral ligand and the ketoester in the favored transition states, rather than steric repulsions in the disfavored transition states explain the enantioselectivity of the asymmetric copper catalysis.

## Conflicts of interest

There are no conflicts to declare.

## Supplementary Material

Supplementary information
