# Supplementary material for: Enantiocontrol by assembled attractive interactions in copper-catalyzed asymmetric direct alkynylation of α-ketoesters with terminal alkynes: OH···O/sp3-CH···O two-point hydrogen bonding combined with dispersive attractions
Source: Chem Sci. 2018 Feb 28;9(14):3484–93. doi: 10.1039/c8sc00527c (PMC5933290; doi:10.1039/c8sc00527c)
Supplement: Supplementary file 1 [file SC-009-C8SC00527C-s001.pdf]

Electronic Supplementary Material (ESI) for Chemical Science.
This journal is © The Royal Society of Chemistry 2018

Electronic Supplementary Information

Enantiocontrol by assembled attractive interactions in copper-catalyzed asymmetric direct alkynylation of α-ketoesters with terminal alkynes: OH···O/sp^3-CH···O two-point hydrogen-bonding combined with dispersive attractions

Martin C. Schwarzer,[b] Akane Fujioka,[a] Takaoki Ishii,[a] Hirohisa Ohmiya,[c] Seiji Mori,*[b] and Masaya Sawamura*[a]

a. *Department of Chemistry, Faculty of Science, Hokkaido University, Sapporo 060-0810, Japan*
E-mail: sawamura@sci.hokudai.ac.jp

b. *Institute of Quantum Beam Science, Ibaraki University, Mito, Ibaraki 310-8512, Japan*
E-mail: seiji.mori.compchem@vc.ibaraki.ac.jp

c. *Division of Pharmaceutical Sciences, Graduate School of Medical Sciences, Kanazawa University, Kakuma-machi, Kanazawa 920-1192, Japan*

Table of Contents Page S-

1 Instrumentation and Chemicals 2
2 Preparation of Chiral Ligands 2
3 Preparation of α-Ketoesters 4
4 Preparation of Alkynes 5
5 Procedures for Asymmetric Direct Alkynylation of α-Ketoesters 5
6 Characterization Data 6
7 Details on the Calculations of the Reaction Pathways and Supplemental Calculations on Different Density Functionals 15
8 Relative Electronic Energies of the Calculated States 17
9 Analyses in Terms of the Quantum Theory of Atoms in Molecules 17
10 Visualisation of Non-covalent Interactions 18
11 Re-optimization of the Aldehyde Model 20
12 Full citation of Gaussian 09 24
13 References 24
14 Absolute Electronic Energies and Coordinates of the Optimised structures 25
15 HPLC Charts 52
16 NMR Spectra 83

1 Instrumentation and Chemicals

NMR spectra were recorded on a Varian Gemini 2000 spectrometer, operating at 300 MHz for ^{1}H NMR and 75.4 MHz for ^{13}C NMR, and a JEOL ECX-400, operating at 400 MHz for ^{1}H NMR, 100.5 MHz for ^{13}C NMR and 161.8 MHz for ^{31}P NMR. Chemical shift values for ^{1}H and ^{13}C and ^{31}P NMR are referenced to Me_4Si, the residual solvent resonances and external aqueous 85% H_3PO_4, respectively. Chemical shifts are reported in δ ppm. Mass spectra were obtained with Thermo Fisher Scientific Exactive, JEOL JMS-T100LP or JEOL JMS-700TZ at the Instrumental Analysis Division, Equipment Management Center, Creative Research Institution, Hokkaido University. Melting points were measured on a Yanaco MP-500D apparatus. HPLC analyses were conducted on a HITACHI ELITE LaChrom system with a HITACHI L-2455 diode array detector. TLC analyses were performed on commercial glass plates bearing 0.25 mm layer of Merck Silica gel $60F_{254}$. Silica gel (Kanto Chemical Co., Silica gel 60 N, spherical, neutral) was used for column chromatography. All reactions were carried out under nitrogen or argon atmosphere. Unless otherwise noted, materials obtained from commercial suppliers were used without further purification. All solvents for catalytic reactions were degassed via three freeze-pump-thaw cycles before use. *t*-BuOH was purchased from Junsei Chem Co., Inc. and other solvents were purchased from Kanto Chem Co., Inc., and used without further purification.

2 Preparation of Chiral Ligands

L1 was prepared through reduction of the corresponding amide according to the literature.[1a] **L4-8** were prepared through reductive amination with the corresponding aminoalcohols and 2-(diorganophosphino)benzaldehydes according to the literature procedures.[1a, 1b]

2.1 [2-(Diphenylphosphanyl)phenyl][(*S*)-2-{(*R*)-1-[(triisopropylsilyl)oxy]ethyl}pyrrolidin-1-yl]methanone (S2)

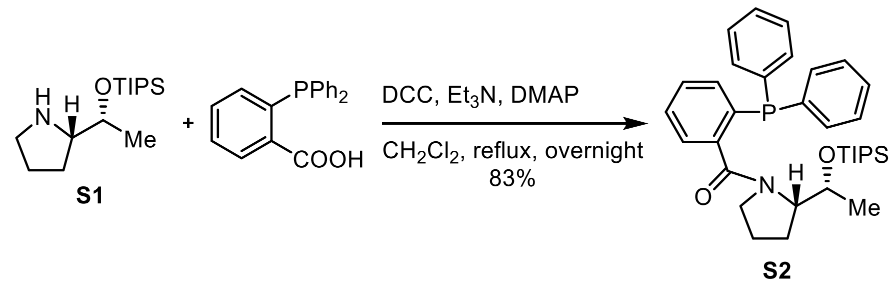

To a solution of **S1** (1.36 g, 5.0 mmol) in CH_2Cl_2 (40 mL) was added dropwise triethylamine (1.53 mL, 11 mmol) at room temperature. The resulting solution was stirred for 10 min. *o*-(Diphenylphosphino)benzoic acid (1.53 g, 5.0 mmol), DCC (1.13 mg, 5.5 mmol) and DMAP (183 mg, 1.5 mmol) were added, and the mixture was stirred overnight at 40 °C. The mixture filtered through a pad of celite. The filtrate was concentrated under reduced pressure. The crude product was purified by column chromatography on silica gel (EtOAc/*n*-hexane = 5 to 10%) to give **S2** (2.31 g, 4.13 mmol) in 83% yield.

2.2 (α*R*,2*S*)-(-)-1-(2-Diphenylphosphinobenzyl)-α-methyl-2-pyrrolidinemethanol (L2)

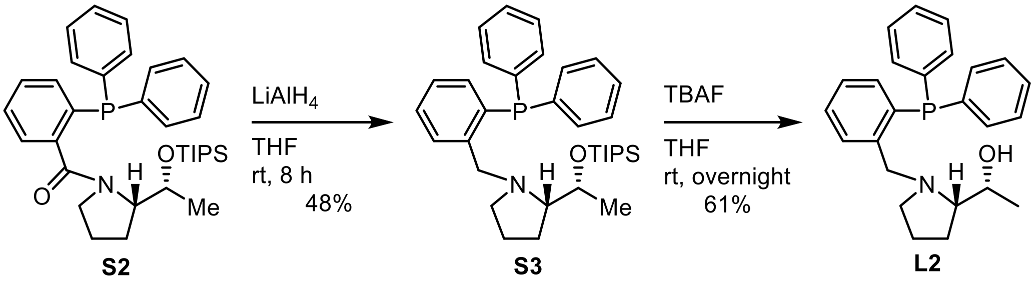

A solution of **S2** (2.31 g, 4.1 mmol) in THF (10 mL) was added dropwise to a suspension of $LiAlH_4$ (313 mg, 8.3 mmol) in THF (5 mL) at 0 ˚C. After being stirred at room temperature for 8 h, the mixture was cooled to 0 °C and quenched by H_2O (0.31 mL), 20% NaOH aq (0.31 mL) and H_2O (0.93 mL) in this order. The mixture was filtered through a pad of celite and the solvent was removed under reduced pressure. The crude product was purified with column chromatography on silica gel (AcOEt/*n*-hexane = 5 to 10%) to give **S3** (1.07 g, 2.0 mmol) in 48% yield.

To a solution of **S3** (1.07 g, 2.0 mmol) in THF (10 mL) was added TBAF (1 M in THF, 5.82 mL, 5.8 mmol) at room temperature. The mixture was stirred overnight at room temperature. The mixture was concentrated under reduced pressure. The crude product was purified by column chromatography on silica gel (EtOAc/*n*-hexane 5 to 10%) to give **L2** (483 mg, 1.2 mmol) in 61% yield. **L2**: Solid. **Mp** 99–100 °C. **^{1}H NMR** (400 Hz, $CDCl_3$) δ 0.62–0.75 (m, 1H), 1.11(d, *J* = 6.4 Hz, 3H), 1.24–1.36 (m, 1H), 1.45–1.57 (m, 1H), 1.60–1.71 (m, 1H), 1.94 (q, *J* = 7.2 Hz, 1H), 2.22 (t, *J* = 9.6 Hz, 1H), 2.39 (t, *J* = 7.6 Hz, 1H), 3.17 (d, *J* = 12.4 Hz, 1H), 3.57 (brs, 1H), 4.14–4.22 (m, 1H), 4.56 (dd, *J* = 12.4, 1.2 Hz, 1H), 6.96–7.03 (m, 1H), 7.14–7.22 (m, 3H), 7.22–7.38 (m, 10H). **^{13}C NMR** (100.5 Hz, $CDCl_3$) δ 18.51, 21.58, 22.62, 54.13, 58.01 (d, $J_{C–P}$ = 13.5 Hz), 63.71, 69.75, 127.37, 128.20, 128.23 (d, $J_{C–P}$ = 5.7 Hz), 128.41 (d, $J_{C–P}$ = 6.7 Hz), 128.89, 129.66 (d, $J_{C–P}$ = 6.7 Hz), 133.24 (d, $J_{C–P}$ = 19.1 Hz), 133.45 (d, $J_{C–P}$ = 19.2 Hz), 135.23, 135.46 (d, $J_{C–P}$ = 13.4 Hz), 136.24 (d, $J_{C–P}$ = 5.7 Hz), 138.17 (d, $J_{C–P}$ = 9.6 Hz), 144.34 (d, $J_{C–P}$ = 23.9 Hz). **^{31}P NMR** (161.8 Hz, $CDCl_3$) δ –17.68. **$[\alpha]_D^{24}$** –42.5 (*c* 1.07, $CHCl_3$). **HRMS–ESI** (*m/z*): $[M+H]^+$ calcd for $C_{25}H_{29}NOP$, 390.1981; found, 390.1979.

2.3 (*S*)-(-)-1-(2-Diphenylphosphinobenzyl)-α,α-dimethyl-2-pyrrolidinemethanol (L3)

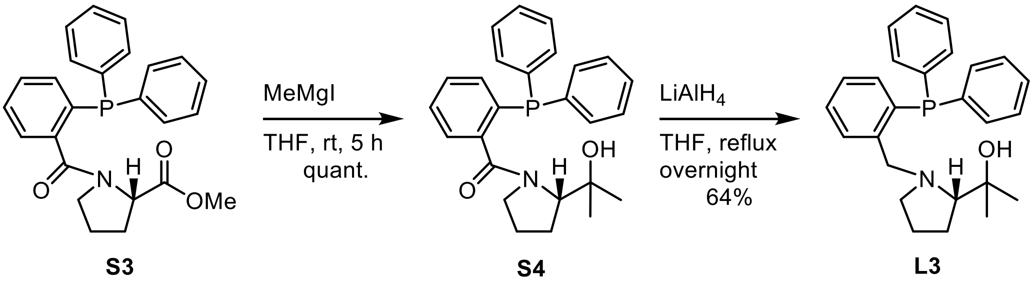

To a solution of **S3** (2.00 g, 5.0 mmol) in Et_2O (5.0 ml) was added Grignard reagent (1.0 M in Et_2O, 12.5 mL, 13 mmol) at 0 °C. After being stirred for 5 h, the mixture was quenched with a saturated NH_4Cl aq at 0 °C. The mixture was filtered through a pad of celite and the solvent was removed under reduced pressure. The obtained solid **S4** (2.20 g, 5.0 mmol) was used for next step without any purification.

A solution of **S4** (2.20 g, 5.0 mmol) in THF (10 mL) was added dropwise to a suspension of $LiAlH_4$ (380 mg, 10 mmol) in THF (10 mL) at 0 ˚C. After being refluxed overnight, the mixture was cooled to 0 °C and quenched by H_2O (0.38 mL), 20% NaOH aq (0.38 mL) and H_2O (1.14 mL) in this order. The mixture was filtered through a pad of celite and the solvent was removed under reduced pressure.

The crude product was purified with column chromatography on silica gel (hexane/CH_2Cl_2 = 50:50 to AcOEt = 100) to give **L3** (1.30 g, 3.2 mmol) in 64% yield. **L3**: Oil. **^{1}H NMR** (400 Hz, $CDCl_3$) δ 0.86 (s, 3H), 1.14 (s, 3H), 1.20–1.39 (m, 1H), 1.40–1.57 (m, 2H), 1.76–1.87 (m,1H), 2.25–2.34 (m, 1H), 2.46 (quin, *J* = 5.2 Hz, 1H), 2.70 (q, *J* = 4.8 Hz, 1H), 3.69 (d, *J* = 13.2 Hz, 1H), 3.93 (brs, 1H), 4.54 (d, *J* = 12.8 Hz, 1H), 6.98–7.03 (m, 1H), 7.10–7.35 (m, 12H), 7.42–7.49 (m, 1H). **^{13}C NMR** (100.5 Hz, $CDCl_3$) δ 23.85, 24.62, 27.54, 27.97, 54.25, 62.78 (d, $J_{C–P}$ = 15.3 Hz), 73.37, 73.42, 127.18, 128.06 (d, $J_{C–P}$ = 6.7 Hz), 128.14 (d, $J_{C–P}$ = 1.9 Hz), 128.23, 128.38, 128.87, 129.53 (d, $J_{C–P}$ = 6.7 Hz), 133.01 (d, $J_{C–P}$ = 18.2 Hz), 133.61 (d, $J_{C–P}$ = 19.2 Hz), 134.88, 135.01 (d, $J_{C–P}$ = 11.6 Hz), 136.10 (d, $J_{C–P}$ = 5.7 Hz), 137.50 (d, $J_{C–P}$ = 8.6 Hz), 145.18 (d, $J_{C–P}$ = 24.9 Hz). **^{31}P NMR** (161.8 Hz, $CDCl_3$) δ –16.47. **$[\alpha]_D^{26}$** –47.4 (*c* 0.96, $CHCl_3$). **HRMS–ESI** (*m/z*): $[M+H]^+$ calcd for $C_{26}H_{31}NOP$, 404.2143; found, 404.2147.

3 Preparation of α-Ketoesters

Ketoesters **1a** and **1r** were obtained from commercial suppliers and distilled before use. Ketoesters **1b** and **1c** were prepared according to the reported procedure.[2a] Ketoesters **1e**,[2b] **1f**,[2c] **1g**,[2b] **1h**,[2c] **1i**,[2d] **1j**,[2d] **1k**,[2c] **1l**,[2c] **1n**,[2b] **1p**,[2a] and **1q**[2c] were prepared according to the reported procedures.[2c]

3.1 2-Hydroxyethyl 2-Oxo-2-phenylacetate (1d)

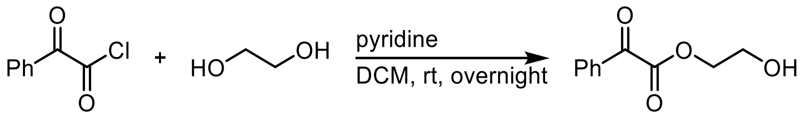

1d

A solution of benzeneacetyl chloride (843 mg, 5.0 mmol) in CH_2Cl_2 (5.0 mL) was added to a solution of pyridine (0.80 mL, 10 mmol) in ethylene glycol (10 mL) at room temperature. After being stirred overnight, the reaction mixture was quenched with saturated NH_4Cl aq. The aqueous layer was extracted with CH_2Cl_2, and the combined organic layer was washed with brine. The organic layer was dried over anhydrous $MgSO_4$, filtered, and evaporated under reduced pressure. The residue was purified by column chromatography on silica gel (EtOAc/*n*-hexane 20 to 50%) to give **1d** (575 mg, 3.0 mmol) in 59% yield. **1d**: Oil. **^{1}H NMR** (400 Hz, $CDCl_3$) δ 2.05 (brs, 1H), 3.98 (dd, *J* = 9.6, 6.0 Hz, 2H), 4.52 (dd, *J* = 6.0, 4.4 Hz, 2H), 7.53 (t, *J* = 8.0 Hz, 2H), 7.68 (t, *J* = 7.2 Hz, 1H), 8.04 (d, *J* = 8.4 Hz, 2H). **^{13}C NMR** (100.5 Hz, $CDCl_3$) δ 60.15, 67.39, 128.77, 130.05, 132.02, 135.02, 163.31, 186.09. **HRMS–ESI** (*m/z*): $[M+Na]^+$ calcd for $C_{10}H_{10}O_4Na$, 217.0471; found, 217.0472.

3.2 *tert*-Butyl 2-(Benzofuran-2-yl)-2-oxoacetate (1m)

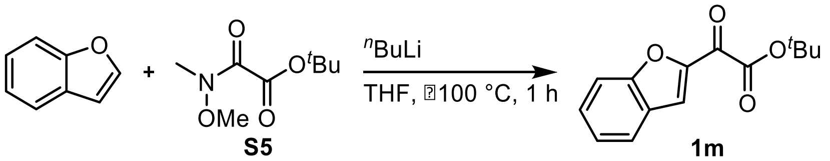

S5 **1m**

To a solution of 2,3-benzofuran (1.07 mL, 10 mmol) in THF (35 mL) was added *n*BuLi (1.64 M in hexane, 9.15 mL, 15 mmol) at -100 °C. After being stirred for 30 min, the mixture was added dropwise to a solution of **S5** (1.89 g, 10 mmol) in THF (15 mL). After being stirred at -100 °C for 1 h, the mixture was quenched with saturated NH_4Cl aq and allowed to warm to room temperature. The mixture was diluted with CH_2Cl_2 and washed 0.1 M HCl. The organic layer was dried over anhydrous $MgSO_4$, filtered, and evaporated under reduced pressure. The residue was purified by column

chromatography on silica gel (EtOAc/*n*-hexane 0 to 10%) to give **1m** (1.41 g, 5.7 mmol) in 57% yield. **1m**: Solid. **Mp** 50–51. **^{1}H NMR** (400 Hz, $CDCl_3$) δ 1.65 (s, 9H), 7.31–7.38 (m, 1H), 7.50–7.56 (m, 1H), 7.61 (d, *J* = 8.4 Hz, 1H), 7.76 (d, *J* = 8.4 Hz, 1H), 7.97 (s, 1H). **^{13}C NMR** (100.5 Hz, $CDCl_3$) δ 27.85, 84.98, 112.60, 119.96, 123.97, 124.25, 126.77, 129.65, 149.50, 156.40, 160.34, 174.43. **HRMS–ESI** (*m/z*): $[M+Na]^+$ calcd for $C_{14}H_{14}O_4Na$, 269.0784; found, 269.0785.

3.3 *tert*-Butyl 2-Oxo-2-(quinolin-3-yl)acetate (1o)

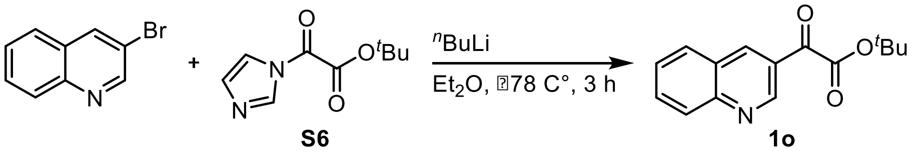

To a solution of 3-bromoqunoline (1.36 mL, 10 mmol) in Et_2O (35 mL) was added *n*BuLi (1.64 M in hexane, 7.30 mL, 12 mmol) at -78 °C. After being stirred for 3 h, the mixture was added dropwise to a solution of **S6** (2.35 g, 12 mmol) in Et_2O (15 mL). After being stirred at -78 °C for 3 h, the mixture was quenched with saturated NH_4Cl aq and allowed to warm to room temperature. The mixture was diluted with Et_2O and washed H_2O and brine. The organic layer was dried over anhydrous $MgSO_4$, filtered, and evaporated under reduced pressure. The residue was purified by column chromatography on silica gel (EtOAc/*n*-hexane = 10 to 30%) to give **1o** (706 mg, 2.7 mmol) in 27% yield. **1o**: Solid. **Mp** 84–86. **^{1}H NMR** (400 Hz, $CDCl_3$) δ 1.68 (s, 9H), 7.67 (t, *J* = 7.6 Hz, 1H), 7.90 (td, *J* = 8.4, 1.6 Hz, 1H), 7.98 (d, *J* = 8.4 Hz, 1H), 8.18 (d, *J* = 8.8 Hz, 1H), 8.87 (d, *J* = 2.0 Hz, 1H), 9.44 (d, *J* = 2.4 Hz, 1H). **^{13}C NMR** (100.5 Hz, $CDCl_3$) δ 28.04, 85.45, 125.35, 126.56, 127.86, 129.56, 129.73, 132.95, 140.08, 149.59, 150.16, 162.30, 185.12. **HRMS–ESI** (*m/z*): $[M+Na]^+$ calcd for $C_{15}H_{15}O_3NNa$, 280.0944; found, 280.0946.

4 Preparation of Alkynes

Alkynes **2a**, **2b**, **2c**, **2d, 2g**, **2i**, **2j**, and **2m** were obtained from commercial suppliers and distilled before use. Alkynes **2e**[3a], **2f**[3b], **2h**[3c], **2k**[3d] and **2l**[3e] were synthesized through simple derivatization of commercial available alkynes.

5 Procedures for Asymmetric Direct Alkynylation of α-Ketoesters

5.1 Using a Glove Box

The reaction in Table 1, entry 7 is representative. In a glove box, CuCl (2.0 mg, 0.02 mmol), K_2CO_3 (8.3 mg, 0.06 mmol) and **L7** (9.2 mg, 0.02 mmol) were placed in a vial containing a magnetic stirring bar. *t*-BuOH (0.4 mL) was added to the vial, and then the resulting mixture was stirred at room temperature for 5 min. Methyl 2-phenylglyoxylate **1a** (28.3 µL, 0.20 mmol) and phenylacetylene (**2a**) (26.4 µL, 0.24 mmol) were added to the vial. The vial was sealed with a screw cap, and removed from the glove box. After being stirred at 25 °C for 48 h, the reaction mixture was concentrated. Then, the residue was subjected to column chromatography on silica gel (EtOAc/*n*-hexane 0 to 5%) to give **3aa** (51.0 mg, 0.19 mmol) in 97% yield.

5.2 No Glove Box Procedure (Table 1, entry 8)

CuCl (2.0 mg, 0.02 mmol), K_2CO_3 (8.3 mg, 0.06 mmol) and **L7** (9.2 mg, 0.02 mmol) were placed in a vial containing a magnetic stirring bar. The vial was sealed with a Teflon®-coated silicon rubber septum. Then, the vial was evacuated and back-filled with argon. *t*-BuOH (0.4 mL) was added to the

vial, and then the resulting mixture was stirred at room temperature for 5 min. Methyl 2-phenylglyoxylate (**1a**) (32.8 mg, 0.20 mmol) and phenylacetylene (**2a**) (24.5 mg, 0.24 mmol) were added to the vial. After being stirred at 25 °C for 48 h, the reaction mixture was concentrated. Then, the residue was subjected to column chromatography on silica gel (EtOAc/n-hexane 0 to 5%) to give **3aa** (52.3 mg, 0.20 mmol) in 98% yield. The ee value (88% ee) was determined by chiral HPLC analysis: CHIRALCEL® IC column, 4.6 mm × 250 mm, Daicel Chemical Industries, hexane/2-propanol = 97:3, 0.5 mL/min, 40 ˚C, 250 nm UV detector, retention time = 42.3 min (minor), 45.7 min (major).

6 Characterization Data

6.1 (*R*)-(+)-Methyl 2-Hydroxy-2,4-diphenylbut-3-ynoate (3aa)

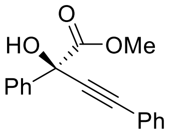

3aa was known compound. (lit.[4] **[α]$_D$^{24}** +19.6, *c* 4.04, $CHCl_3$, *R*). **[α]$_D$^{21}** +16.1 (*c* 0.98, $CHCl_3$). The ee value (88% ee) was determined by chiral HPLC analysis: CHIRALCEL® IC column, 4.6 mm × 250 mm, Daicel Chemical Industries, hexane/2-propanol = 97:3, 0.5 mL/min, 40 ˚C, 250 nm UV detector, retention time = 39.8 min (minor), 42.5 min (major).

6.2 (*R*)-(+)-*iso*-Propyl 2-Hydroxy-2,4-diphenylbut-3-ynoate (3ba)

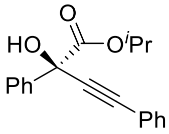

(*S*)-Compound was reported. (lit.[5] **[α]$_D$^{20}** –27.9, *c* 0.50, $CHCl_3$, *S*). **[α]$_D$^{22}** +29.9 (*c* 1.07, $CHCl_3$). The ee value (90% ee) was determined by chiral HPLC analysis: CHIRALCEL® IC column, 4.6 mm × 250 mm, Daicel Chemical Industries, hexane/2-propanol = 97:3, 0.5 mL/min, 40 ˚C, 250 nm UV detector, retention time = 28.1 min (major), 30.7 min (minor).

6.3 (*R*)-(+)-*tert*-Butyl 2-Hydroxy-2,4-diphenylbut-3-ynoate (3ca)

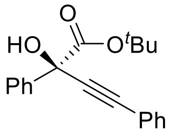

Oil. **^{1}H NMR** (300 MHz, $CDCl_3$) δ 1.43 (s, 9H), 4.39 (s, 1H), 7.30–7.42 (m, 6H), 7.48–7.54 (m, 2H), 7.69–7.75 (m, 2H). **^{13}C NMR** (75.4 MHz, $CDCl_3$) δ 27.55, 73.32, 84.32, 85.42, 87.69, 122.24, 126.16, 128.14, 128.29, 128.35, 128.73, 131.85, 139.85, 170.82. **HRMS–ESI** (*m/z*): $[M+Na]^+$ calcd for $C_{20}H_{20}O_3Na$, 331.1305; found, 331.1305. **[α]$_D$^{23}** +22.8 (*c* 1.30, $CHCl_3$). The ee value (92% ee) was determined by chiral HPLC analysis: CHIRALCEL® IC column, 4.6 mm × 250 mm, Daicel Chemical Industries, hexane/2-propanol = 97:3, 0.5 mL/min, 40 ˚C, 250 nm UV detector, retention time = 25.4 min (major), 29.7 min (minor).

6.4 (*R*)-(+)-2-Hydroxyethyl 2-Hydroxy-2,4-diphenylbut-3-ynoate (3da)

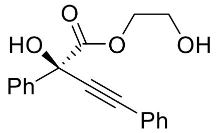

White Solid. **Mp** 94–95 °C. ^{1}**H NMR** (400 MHz, $CDCl_3$) δ 3.71–3.85 (m, 2H), 4.20–4.45 (m, 3H), 7.32–7.47 (m, 6H), 7.50–7.58 (m, 2H), 7.74–7.81 (m, 2H). ^{13}**C NMR** (100.5 MHz, $CDCl_3$) δ 60.60, 68.47, 73.43, 86.54, 86.77, 121.62, 126.16, 128.36, 128.50, 128.92, 129.07, 131.92, 139.19, 171.80. **HRMS–ESI** (*m/z*): $[M+Na]^+$ calcd for $C_{18}H_{16}O_4Na$, 319.0941; found, 319.0941. $[\alpha]_D^{26}$ +21.2 (*c* 1.19, $CHCl_3$). The ee value (90% ee) was determined by chiral HPLC analysis: CHIRALCEL® IC column, 4.6 mm × 250 mm, Daicel Chemical Industries, hexane/2-propanol = 90:10, 0.5 mL/min, 40 ˚C, 250 nm UV detector, retention time = 25.9 min (minor), 33.3 min (major).

6.5 (*R*)-(+)-*tert*-Butyl 2-(4-Fluorophenyl)-2-hydroxy-4-phenylbut-3-ynoate (3ea)

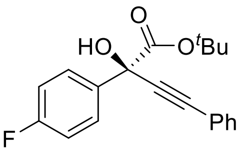

Oil. ^{1}**H NMR** (300 MHz, $CDCl_3$) δ 1.43 (s, 9H), 4.40 (s, 1H), 7.02–7.10 (m, 2H), 7.31–7.38 (m, 3H), 7.46–7.53 (m, 2H), 7.66–7.74 (m, 2H). ^{13}**C NMR** (75.4 MHz, $CDCl_3$) δ 27.55, 72.81, 84.53, 85.56, 87.49, 115.00 (d, $J_{C\text{-}F}$ = 21.8 Hz), 122.03, 128.11 (d, $J_{C\text{-}F}$ = 8.2 Hz), 128.33, 128.87, 131.85, 135.69, 162.77 (d, $J_{C\text{-}F}$ =246.8 Hz), 170.62. **HRMS–ESI** (*m/z*): $[M+Na]^+$ calcd for $C_{20}H_{19}O_3FNa$, 349.1210; found, 349.1209. $[\alpha]_D^{24}$ +26.3 (*c* 1.40, $CHCl_3$). The ee value (89% ee) was determined by chiral HPLC analysis: CHIRALCEL® AD-H column, 4.6 mm × 250 mm, Daicel Chemical Industries, hexane/2-propanol = 97:3, 0.5 mL/min, 40 ˚C, 250 nm UV detector, retention time = 15.7 min (major), 17.1 min (minor).

6.6 (*R*)-(+)-*tert*-Butyl 2-(4-Chlorophenyl)-2-hydroxy-4-phenylbut-3-ynoate (3fa)

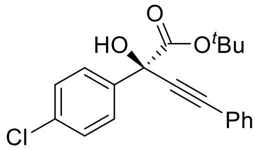

Oil. ^{1}**H NMR** (300 MHz, $CDCl_3$) δ 1.43 (s, 9H), 4.40 (s, 1H), 7.30–7.41 (m, 5H), 7.45–7.54 (m, 2H), 7.62–7.71 (m, 2H). ^{13}**C NMR** (75.4 MHz, $CDCl_3$) δ 27.53, 72.82, 84.67, 85.61, 87.30, 121.96, 127.72 128.29, 128.34, 128.90, 131.86, 134.34, 138.45, 170.41. **HRMS–ESI** (*m/z*): $[M+Na]^+$ calcd for $C_{20}H_{19}O_3ClNa$, 365.0915; found, 365.0915. $[\alpha]_D^{24}$ +27.9 (*c* 1.46, $CHCl_3$). The ee value (92% ee) was determined by chiral HPLC analysis: CHIRALCEL® IC column, 4.6 mm × 250 mm, Daicel Chemical Industries, hexane/2-propanol = 97:3, 0.5 mL/min, 40 ˚C, 250 nm UV detector, retention time = 15.6 min (major), 16.7 min (minor).

6.7 (*R*)-(+)-*tert*-Butyl 2-(4-Bromophenyl)-2-hydroxy-4-phenylbut-3-ynoate (3ga)

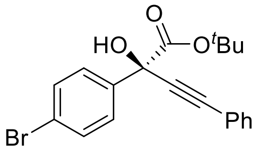

Oil. **^{1}H NMR** (400 MHz, $CDCl_3$) δ 1.43 (s, 9H), 4.43–4.45 (m, 1H), 7.31–7.38 (m, 3H), 7.46–7.52 (m, 4H), 7.61 (dd, *J* = 6.8, 2.0 Hz, 2H). **^{13}C NMR** (100.5 MHz, $CDCl_3$) δ 27.52, 72.84, 84.69, 85.59, 87.20, 121.91, 122.59, 128.03, 128.33, 128.90, 131.22, 131.83, 138.96, 170.31. **HRMS–ESI** (*m/z*): $[M+Na]^+$ calcd for $C_{20}H_{19}O_3BrNa$, 409.0410; found, 409.0415. **$[\alpha]_D^{26}$** +28.1 (*c* 0.55, $CHCl_3$). The ee value (92% ee) was determined by chiral HPLC analysis: CHIRALCEL® IC column, 4.6 mm × 250 mm, Daicel Chemical Industries, hexane/2-propanol = 97:3, 0.5 mL/min, 40 ˚C, 250 nm UV detector, retention time = 17.2 min (major), 19.1 min (minor).

6.8 (*R*)-(+)-*tert*-Butyl 2-Hydroxy-2-(4-methoxyphenyl)-4-phenylbut-3-ynoate (3ha)

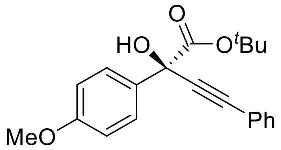

Oil. **^{1}H NMR** (400 MHz, $CDCl_3$) δ 1.44 (s, 9H), 3.82 (s, 3H), 4.35 (s, 1H), 6.87–6.92 (m, 2H), 7.31–7.37 (m, 3H), 7.48–7.53 (m, 2H), 7.62–7.65 (m, 2H). **^{13}C NMR** (100.5 MHz, $CDCl_3$) δ 27.57, 55.25, 72.92, 84.17, 85.27, 87.84, 113.44, 122.26, 127.44, 128.27, 128.69, 131.83, 132.03, 159.57, 171.00. **HRMS–ESI** (*m/z*): $[M+Na]^+$ calcd for $C_{21}H_{22}O_4Na$, 361.1410; found, 361.1413. **$[\alpha]_D^{26}$** +28.1 (*c* 0.55, $CHCl_3$). The ee value (91% ee) was determined by chiral HPLC analysis: CHIRALCEL® IC column, 4.6 mm × 250 mm, Daicel Chemical Industries, hexane/2-propanol = 98:2, 0.5 mL/min, 40 ˚C, 250 nm UV detector, retention time = 63.1 min (major), 80.4 min (minor).

6.9 (*R*)-(+)-*tert*-Butyl 2-Hydroxy-2-(3-methoxyphenyl)-4-phenylbut-3-ynoate (3ia)

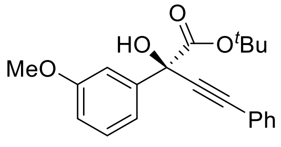

Oil. **^{1}H NMR** (300 MHz, $CDCl_3$) δ 1.44 (s, 9H), 3.83 (s, 3H), 4.37 (s, 1H), 6.85–6.91 (m, 1H), 7.27–7.37 (m, 6H), 7.47–7.53 (m, 2H). **^{13}C NMR** (75.4 MHz, $CDCl_3$) δ 27.58, 55.27, 73.23, 84.37, 85.37, 87.62, 111.77, 114.07, 118.66, 122.25, 128.30, 128.74, 129.16, 131.86, 141.41, 159.44, 170.72. **HRMS–ESI** (*m/z*): $[M+Na]^+$ calcd for $C_{21}H_{22}O_4Na$, 361.1410; found, 361.1412. **$[\alpha]_D^{24}$** +20.5 (*c* 1.48, $CHCl_3$). The ee value (93% ee) was determined by chiral HPLC analysis: CHIRALCEL® OD-3 column, 4.6 mm × 250 mm, Daicel Chemical Industries, hexane/2-propanol = 98:2, 0.5 mL/min, 40 ˚C, 250 nm UV detector, retention time = 16.9 min (major), 18.6 min (minor).

6.10 (*R*)-(+)-*tert*-Butyl 2-(Benzo[d][1,3]dioxol-5-yl)-2-hydroxy-4-phenylbut-3-ynoate (3ja)

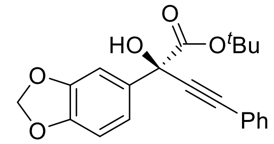

Oil. **^{1}H NMR** (400 MHz, $CDCl_3$) δ 1.45 (s, 9H), 4.35 (s, 1H), 5.99 (s, 2H), 6.80 (d, *J* = 8.0 Hz, 1H), 7.18–7.24 (m, 2H), 7.29–7.37 (m, 3H), 7.48–7.52 (m, 2H). **^{13}C NMR** (100.5 MHz, $CDCl_3$) δ 27.57, 73.02, 84.33, 85.38, 87.61, 101.22, 107.00, 107.76, 119.90, 122.11, 128.28, 128.75, 131.81, 133.81, 147.48, 147.63, 170.76. **HRMS–ESI** (*m/z*): $[M+Na]^+$ calcd for $C_{21}H_{20}O_5Na$, 375.1203; found, 375.1208. **$[\alpha]_D^{27}$** +27.0 (*c* 0.50, $CHCl_3$). The ee value (93% ee) was determined by chiral HPLC analysis: CHIRALCEL® IC column, 4.6 mm × 250 mm, Daicel Chemical Industries, hexane/2-propanol = 90:10, 0.5 mL/min, 40 ˚C, 250 nm UV detector, retention time = 27.2 min (minor), 31.7 min (major).

6.11 (*R*)-(+)-*tert*-Butyl 2-Hydroxy-2-(naphthalen-2-yl)-4-phenylbut-3-ynoate (3ka)

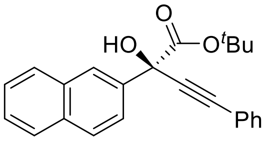

Oil. **^{1}H NMR** (300 MHz, $CDCl_3$) δ 1.43 (s, 9H), 4.50 (s, 1H), 7.33–7.41 (m, 3H), 7.47–7.58 (m, 4H), 7.67–7.80 (m, 1H), 7.81–7.92 (m, 3H), 8.20–8.25 (m, 1H). **^{13}C NMR** (75.4 MHz, $CDCl_3$) δ 27.58, 73.44, 84.49, 85.63, 87.71, 122.24, 124.12, 125.33, 126.16, 126.38, 127.54, 127.96, 128.32, 128.50, 128.80, 131.91, 132.90, 133.20, 137.16, 170.79. **Anal.** Calcd for $C_{24}H_{22}O_3$: C, 80.42; H, 6.19%. Found: C, 80.04; H, 6.18%. **$[\alpha]_D^{24}$** +40.2 (*c* 1.46, $CHCl_3$). The ee value (94% ee) was determined by chiral HPLC analysis: CHIRALCEL® AD-H column, 4.6 mm × 250 mm, Daicel Chemical Industries, hexane/2-propanol = 97:3, 0.5 mL/min, 40 ˚C, 250 nm UV detector, retention time = 32.1 min (major), 36.4 min (minor).

6.12 (*S*)-(-)-*tert*-Butyl 2-(Furan-2-yl)-2-hydroxy-4-phenylbut-3-ynoate (3la)

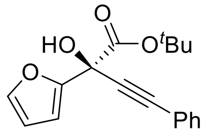

Oil. **^{1}H NMR** (400 MHz, $CDCl_3$) δ 1.50 (s, 9H), 4.34 (s, 1H), 6.38 (dd, *J* = 3.2, 2.0 Hz, 1H), 6.65 (dd, *J* = 3.2, 0.8 Hz, 1H), 7.31–7.37 (m, 3H), 7.41 (dd, *J* = 2.0, 0.8 Hz, 1H), 7.48–7.52 (m, 2H). **^{13}C NMR** (100.5 MHz, $CDCl_3$) δ 27.66, 68.71, 84.73, 84.83, 85.23, 109.11, 110.39, 121.85, 128.30, 128.94, 131.93, 143.13, 151.73, 168.70. **HRMS–ESI** (*m/z*): $[M+Na]^+$ calcd for $C_{18}H_{18}O_4Na$, 321.1097; found, 321.1098. **$[\alpha]_D^{29}$** –9.78 (*c* 0.94, $CHCl_3$). The ee value (66% ee) was determined by chiral HPLC analysis: CHIRALCEL® IC column, 4.6 mm × 250 mm, Daicel Chemical Industries, hexane/2-propanol = 97:3, 0.5 mL/min, 40 ˚C, 250 nm UV detector, retention time = 51.0 min (minor), 59.5 min (major).

6.13 (*S*)-(-)-*tert*-Butyl 2-(Benzofuran-2-yl)-2-hydroxy-4-phenylbut-3-ynoate (3ma)

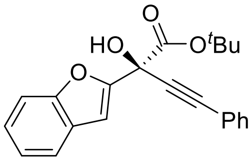

White solid. **Mp** 101–103 °C. **^{1}H NMR** (400 MHz, $CDCl_3$) δ 1.49 (s, 9H), 4.47 (s, 1H), 7.07 (s, 1H), 7.22–7.40 (m, 5H), 7.48 (d, *J* = 8.4 Hz, 1H), 7.52–7.56 (m, 2H), 7.59 (dd, *J* = 7.6, 0.8 Hz, 1H). **^{13}C NMR** (100.5 MHz, $CDCl_3$) δ 27.63, 69.10, 84.86, 85.24, 105.99, 111.51, 121.47, 121.69, 122.96, 124.85, 127.63, 128.33, 128.86, 129.07, 131.97, 154.25, 155.32, 168.40. **HRMS–ESI** (*m/z*): $[M+Na]^+$ calcd for $C_{22}H_{20}O_4Na$, 371.1254; found, 371.1252. **$[\alpha]_D^{28}$** –15.4 (*c* 0.54, $CHCl_3$). The ee value (69% ee) was determined by chiral HPLC analysis: CHIRALCEL® IC column, 4.6 mm × 250 mm, Daicel Chemical Industries, hexane/2-propanol = 90:10, 0.5 mL/min, 40 ˚C, 250 nm UV detector, retention time = 17.7 min (minor), 19.2 min (major).

6.14 (*R*)-(+)-*tert*-Butyl 2-Hydroxy-4-phenyl-2-(thiophen-2-yl)but-3-ynoate (3na)

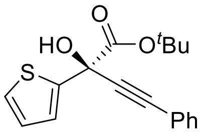

White solid. **Mp** 49–50 °C. **^{1}H NMR** (400 MHz, $CDCl_3$) δ 1.49 (s, 9H), 4.50 (s, 1H), 6.98 (dd, *J* = 5.2, 4.0 Hz, 1H), 7.27–7.38 (m, 5H), 7.45–7.52 (m, 2H). **^{13}C NMR** (100.5 MHz, $CDCl_3$) δ 27.55, 70.71, 84.82, 87.25, 121.95, 125.84, 125.95, 126.67, 128.27, 128.85, 131.85, 144.21, 169.71. **HRMS–ESI** (*m/z*): $[M+Na]^+$ calcd for $C_{18}H_{18}O_3NaS$, 337.0869; found, 337.0870. **$[\alpha]_D^{23}$** +49.4 (*c* 1.00, $CHCl_3$). The ee value (84% ee) was determined by chiral HPLC analysis: CHIRALCEL® IC column, 4.6 mm × 250 mm, Daicel Chemical Industries, hexane/2-propanol = 97:3, 0.5 mL/min, 40 ˚C, 250 nm UV detector, retention time = 28.9 min (major), 44.3 min (minor).

6.15 (*S*)-(+)-*tert*-Butyl 2-Hydroxy-4-phenyl-2-(quinolin-3-yl)but-3-ynoate (3oa)

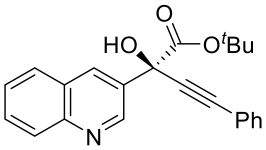

White solid. **Mp** 155–157 °C. **^{1}H NMR** (400 MHz, $CDCl_3$) δ 1.45 (s, 9H), 4.63 (m, 1H), 7.35–7.41 (m, 3H), 7.50–7.55 (m, 2H), 7.56–7.62 (m, 1H), 7.72–7.78 (m, 1H), 7.89 (d, *J* = 8.0 Hz, 1H), 8.14 (d, *J* = 8.8 Hz, 1H), 8.52 (d, *J* = 2.4 Hz, 1H), 9.27 (d, *J* = 2.4 Hz, 1H). **^{13}C NMR** (100.5 MHz, $CDCl_3$) δ 27.58, 71.86, 85.33, 86.16, 86.89, 121.71, 126.98, 127.24, 128.31, 128.39, 129.08, 129.14, 129.93, 131.92, 132.68, 133.06, 147.67, 149.48, 170.04. **HRMS–ESI** (*m/z*): $[M+Na]^+$ calcd for $C_{23}H_{21}O_3NNa$, 382.1414; found, 382.1415. **$[\alpha]_D^{26}$** +127.4 (*c* 1.00, $CHCl_3$). The ee value (93% ee) was determined by chiral HPLC analysis: CHIRALCEL® IC column, 4.6 mm × 250 mm, Daicel Chemical Industries, hexane/2-propanol = 50:50, 0.5 mL/min, 40 ˚C, 250 nm UV detector, retention time = 10.6 min (major), 31.7 min (minor).

6.16 (*R*)-(-)-*tert*-Butyl 2-(Cyclohexylmethyl)-2-hydroxy-4-phenylbut-3-ynoate (3pa)

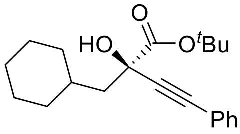

Oil. **^{1}H NMR** (300 MHz, $CDCl_3$) δ 0.93–2.02 (m, 13H), 1.55 (s, 9H), 3.64 (s, 1H), 7.26–7.35 (m, 3H), 7.38–7.44 (m, 2H). **^{13}C NMR** (75.4 MHz, $CDCl_3$) δ 26.26, 26.29, 27.71, 27.77, 34.04, 34.47, 46.39, 71.37, 83.60, 89.01, 122.49, 128.21, 128.43, 131.69, 172.13. **HRMS–ESI** (*m/z*): $[M+Na]^+$ calcd for $C_{21}H_{28}O_3Na$, 351.1931; found, 351.1930. **$[\alpha]_D^{25}$** –16.7 (*c* 0.92, $CHCl_3$). The ee value (90% ee) was determined by chiral HPLC analysis: CHIRALCEL® IC column, 4.6 mm × 250 mm, Daicel Chemical Industries, hexane/2-propanol = 99:1, 0.5 mL/min, 40 ˚C, 250 nm UV detector, retention time = 18.0 min (major), 20.0 min (minor).

6.17 (*R*)-(-)-*tert*-Butyl 2-Cyclohexyl-2-hydroxy-4-phenylbut-3-ynoate (3qa)

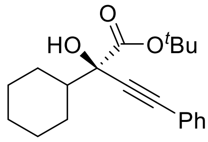

Oil. **^{1}H NMR** (300 MHz, $CDCl_3$) δ 1.08–1.98 (m, 10H), 1.54 (s, 9H), 2.10–2.20 (m, 1H), 3.59 (s, 1H), 7.25–7.36 (m, 3H), 7.40–7.45 (m, 2H). **^{13}C NMR** (75.4 MHz, $CDCl_3$) δ 26.12, 26.14, 26.31, 26.40, 26.72, 27.86, 46.25, 74.63, 83.63, 84.19, 88.12, 122.58, 128.19, 128.39, 131.76, 171.75. **HRMS–ESI** (*m/z*): $[M+Na]^+$ calcd for $C_{20}H_{26}O_3Na$, 337.1774; found, 337.1773. **$[\alpha]_D^{24}$** –16.7 (*c* 1.00, $CHCl_3$). The ee value (86% ee) was determined by chiral HPLC analysis: CHIRALCEL® IC column, 4.6 mm × 250 mm, Daicel Chemical Industries, hexane/2-propanol = 99.5:0.5, 0.5 mL/min, 40 ˚C, 250 nm UV detector, retention time = 27.9 min (major), 33.2 min (minor).

6.18 (*R*)-(+)-3-Hydroxy-4,4-dimethyl-3-(phenylethynyl)dihydrofuran-2(3H)-one (3ra)

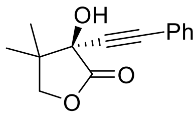

(*S*)-Compound was reported. (lit.[4] **$[\alpha]_D^{20}$** –17.5, *c* 0.45, $CHCl_3$) **$[\alpha]_D^{23}$** +1.3 (*c* 0.73, $CHCl_3$). The ee value (90% ee) was determined by chiral HPLC analysis: CHIRALCEL® IC column, 4.6 mm × 250 mm, Daicel Chemical Industries, hexane/2-propanol = 97:3, 0.5 mL/min, 40 ˚C, 250 nm UV detector, retention time = 43.3 min (minor), 53.8 min (major).

6.19 (*R*)-(+)-*tert*-Butyl 2-Hydroxy-4-(4-methoxyphenyl)-2-phenylbut-3-ynoate (3cb)

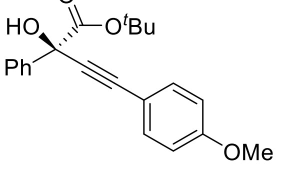

Oil. **^{1}H NMR** (300 MHz, $CDCl_3$) δ 1.43 (s, 9H), 3.82 (s, 3H), 4.37 (s, 1H), 6.82–6.89 (m, 2H), 7.28–7.52 (m, 5H), 7.68–7.77 (m, 2H). **^{13}C NMR** (75.4 MHz, $CDCl_3$) δ 27.56, 55.29, 73.37, 84.18, 85.46, 86.33, 113.91, 114.31, 126.20, 128.11, 128.29, 133.34, 140.02, 159.91, 170.95. **Anal.** Calcd for $C_{21}H_{22}O_4$: C,

74.54; H, 6.55%. Found: C, 74.32; H, 6.56%. **[α]$_D^{24}$** +33.1 (*c* 1.29, $CHCl_3$). The ee value (93% ee) was determined by chiral HPLC analysis: CHIRALCEL® OD-3 column, 4.6 mm × 250 mm, Daicel Chemical Industries, hexane/2-propanol = 97:3, 0.5 mL/min, 40 ˚C, 250 nm UV detector, retention time = 43.8 min (major), 51.1 min (minor).

6.20 (*R*)-(+)-*tert*-Butyl 2-Hydroxy-2-phenyl-4-[4-(trifluoromethyl)phenyl]but-3-ynoate (3cc)

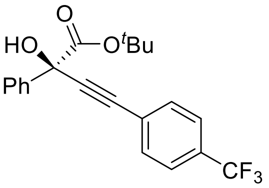

Oil. **^{1}H NMR** (300 MHz, $CDCl_3$) δ 1.44 (s, 9H), 4.42 (s, 1H), 7.30–7.44 (m, 3H), 7.57–7.66 (m, 4H), 7.66–7.74 (m, 2H). **^{13}C NMR** (75.4 MHz, $CDCl_3$) δ 27.53, 73.27, 83.88, 84.64, 90.19, 123.80 (q, $J_{C–F}$ = 272.5 Hz), 125.25 (q, $J_{C–F}$ = 4.0 Hz), 126.03, 128.25, 128.54, 130.49 (q, $J_{C–F}$ = 32.6 Hz), 132.12, 139.52, 170.48. **HRMS–ESI** (*m/z*): $[M+Na]^+$ calcd for $C_{21}H_{19}O_3F_3Na$, 399.1179; found, 399.1178. **[α]$_D^{23}$** +8.8 (*c* 2.00, $CHCl_3$). The ee value (88% ee) was determined by chiral HPLC analysis: CHIRALCEL® IC column, 4.6 mm × 250 mm, Daicel Chemical Industries, hexane/2-propanol = 97:3, 0.5 mL/min, 40 ˚C, 250 nm UV detector, retention time = 13.8 min (major), 16.0 min (minor).

6.21 (*R*)-(+)-Methyl 4-[4-(*tert*-Butoxy)-3-hydroxy-4-oxo-3-phenylbut-1-yn-1-yl]benzoate (3cd)

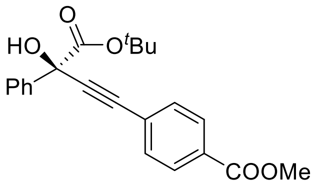

White solid. **Mp** 103–104 °C. **^{1}H NMR** (400 MHz, $CDCl_3$) δ 1.44 (s, 9H), 3.93 (s, 3H), 4.43 (s, 1H), 7.32–7.42 (m, 3H), 7.57 (dd, *J* = 6.4, 1.6 Hz, 2H), 7.69–7.73 (m, 2H), 8.01 (dd, *J* = 6.4, 1.6 Hz, 2H). **^{13}C NMR** (100.5 MHz, $CDCl_3$) δ 27.56, 52.31, 73.29, 84.49, 84.65, 90.58, 126.06, 126.85, 128.25, 128.52, 129.46, 130.01, 131.81, 139.53, 166.44, 170.53. **HRMS–ESI** (*m/z*): $[M+Na]^+$ calcd for $C_{22}H_{22}O_5Na$, 389.1359; found, 389.1362. **[α]$_D^{28}$** +32.6 (*c* 0.64, $CHCl_3$). The ee value (85% ee) was determined by chiral HPLC analysis: CHIRALCEL® OD-3 column, 4.6 mm × 250 mm, Daicel Chemical Industries, hexane/2-propanol = 97:3, 0.5 mL/min, 40 ˚C, 250 nm UV detector, retention time = 19.8 min (minor), 22.1 min (major).

6.22 (*R*)-(+)-*tert*-Butyl 2-Hydroxy-2-phenyl-4-(thiophen-2-yl)but-3-ynoate (3ce)

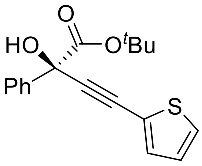

Oil. **^{1}H NMR** (400 MHz, $CDCl_3$) δ 1.43 (s, 9H), 4.39 (s, 1H), 7.00 (dd, *J* = 5.2, 3.6 Hz, 1H), 7.27–7.41 (m, 5H), 7.67–7.71 (m, 2H). **^{13}C NMR** (100.5 MHz, CD_2Cl_2) δ 26.94, 73.17, 78.30, 84.27, 91.26, 121.55, 125.76, 126.77, 127.56, 127.87, 128.12, 132.46, 139.44, 170.14. **HRMS–ESI** (*m/z*): $[M+Na]^+$ calcd for $C_{18}H_{18}O_3NaS$, 337.0869; found, 337.0870. **[α]$_D^{28}$** +32.2 (*c* 1.30, $CHCl_3$). The ee value (90% ee) was determined by chiral HPLC analysis: CHIRALCEL® IC column, 4.6 mm × 250 mm, Daicel Chemical

Industries, hexane/2-propanol = 90:10, 0.5 mL/min, 40 ˚C, 250 nm UV detector, retention time = 14.1 min (major), 15.2 min (minor).

6.23 (*R*)-(+)-*tert*-Butyl 2-Hydroxy-4-(1-methyl-1H-indol-2-yl)-2-phenylbut-3-ynoate (3cf)

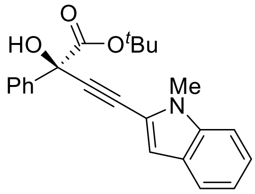

White solid. **Mp** 114–115 °C. **^{1}H NMR** (400 MHz, $CDCl_3$) δ 1.44 (s, 9H), 3.84 (s, 3H), 4.45 (s, 1H), 6.84 (s, 1H), 7.10–7.15 (m, 1H), 7.27–7.30 (m, 2H), 7.33–7.43 (m, 3H), 7.58–7.60 (m, 1H), 7.73–7.75 (m, 2H). **^{13}C NMR** (100.5 MHz, $CDCl_3$) δ 27.54, 30.61, 73.51, 84.62, 93.72, 108.02, 109.40, 120.13, 120.84, 121.04, 123.23, 126.03, 126.92, 128.24, 128.51, 137.18, 139.57, 170.49. **HRMS–ESI** (*m/z*): $[M+Na]^+$ calcd for $C_{23}H_{23}O_3NNa$, 384.1570; found, 384.1579. **$[\alpha]_D^{28}$** +34.3 (*c* 0.52, $CHCl_3$). The ee value (71% ee) was determined by chiral HPLC analysis: CHIRALCEL® IC column, 4.6 mm × 250 mm, Daicel Chemical Industries, hexane/2-propanol = 97:3, 1.0 mL/min, 40 ˚C, 250 nm UV detector, retention time = 16.8 min (major), 17.9 min (minor).

6.24 (*R*)-(+)-*tert*-Butyl 4-(Cyclohex-1-en-1-yl)-2-hydroxy-2-phenylbut-3-ynoate (3cg)

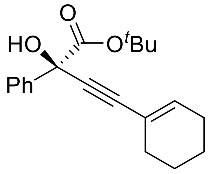

Oil. **^{1}H NMR** (300 MHz, $CDCl_3$) δ 1.40 (s, 9H), 1.55–1.71 (m, 4H), 2.07–2.21 (m, 4H), 4.28 (s, 1H), 6.18–6.22 (m, 1H), 7.25–7.38 (m, 3H), 7.60–7.68 (m, 2H). **^{13}C NMR** (75.4 MHz, $CDCl_3$) δ 21.41, 22.19, 25.62, 27.54, 28.93, 73.27, 83.98, 84.97, 87.39, 119.89, 126.20, 128.04, 128.19, 136.06, 140.11, 171.07. **HRMS–ESI** (*m/z*): $[M+Na]^+$ calcd for $C_{20}H_{24}O_3Na$, 335.1618; found, 335.1618. **$[\alpha]_D^{22}$** +23.2 (*c* 1.03, $CHCl_3$). The ee value (90% ee) was determined by chiral HPLC analysis: CHIRALCEL® OJ-3 column, 4.6 mm × 250 mm, Daicel Chemical Industries, hexane/2-propanol = 95:5, 0.5 mL/min, 40 ˚C, 250 nm UV detector, retention time = 18.9 min (major), 22.5 min (minor).

6.25 (*R,E*)-(+)-*tert*-Butyl 2-Hydroxy-2,6-diphenylhex-5-en-3-ynoate (3ch)

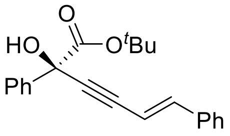

White solid. **Mp** 64 °C. **^{1}H NMR** (400 MHz, $CDCl_3$) δ 1.43 (s, 9H), 4.37 (s, 1H), 6.26 (d, *J* = 16.0 Hz, 1H), 7.05 (d, *J* = 16.0 Hz, 1H), 7.27–7.43 (m, 8H), 7.66–7.71 (m, 2H). **^{13}C NMR** (100.5 MHz, $CDCl_3$) δ 27.53, 73.39, 84.36, 84.64, 89.59, 107.11, 126.10, 126.32, 128.12, 128.33, 128.72, 128.85, 135.88, 139.86, 142.53, 170.73. **HRMS–ESI** (*m/z*): $[M+Na]^+$ calcd for $C_{22}H_{22}O_3Na$, 357.1461; found, 357.1468. **$[\alpha]_D^{27}$** +30.6 (*c* 0.51, $CHCl_3$). The ee value (87% ee) was determined by chiral HPLC analysis: CHIRALCEL® OD-3 column, 4.6 mm × 250 mm, Daicel Chemical Industries, hexane/2-propanol = 90:10, 0.5 mL/min, 40 ˚C, 220 nm UV detector, retention time = 15.0 min (minor), 18.0 min (major).

6.26 (*R*)-(+)-*tert*-Butyl 2-(4-Chlorophenyl)-2-hydroxy-6-phenylhex-3-ynoate (3fi)

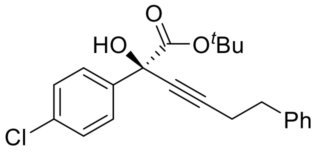

Oil. ^{1}**H NMR** (300 MHz, $CDCl_3$) δ 1.37 (s, 9H), 2.62 (t, *J* =7.1 Hz, 2H), 2.88 (t, *J* =7.1 Hz, 2H), 4.26 (s, 1H), 7.20–7.35 (m, 7H), 7.43–7.48 (m, 2H). ^{13}**C NMR** (75.4 MHz, $CDCl_3$) δ 20.75, 27.46, 34.55, 72.41, 79.32, 84.28, 85.88, 126.37, 127.70, 128.08, 128.41, 128.47, 134.06, 138.70, 140.28, 170.65. **HRMS–ESI** (*m/z*): $[M+Na]^+$ calcd for $C_{22}H_{23}O_3ClNa$, 393.1228; found, 393.1229. $[\alpha]_D^{24}$ +27.4 (*c* 1.12, $CHCl_3$). The ee value (87% ee) was determined by chiral HPLC analysis: CHIRALCEL® OD-3 column, 4.6 mm × 250 mm, Daicel Chemical Industries, hexane/2-propanol = 97:3, 0.5 mL/min, 40 ˚C, 250 nm UV detector, retention time = 21.5 min (minor), 23.6 min (major).

6.27 (*R*)-(+)-*tert*-Butyl 2-(4-Chlorophenyl)-4-cyclohexyl-2-hydroxybut-3-ynoate (3fj)

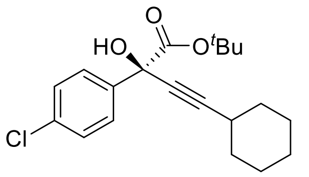

Oil. ^{1}**H NMR** (300 MHz, $CDCl_3$) δ 1.31–1.43 (m, 2H), 1.40 (s, 9H), 1.43–1.62 (m, 3H), 1.65–1.86 (m, 5H), 2.49–2.60 (m, 1H), 4.25 (s, 1H), 7.28–7.36 (m, 2H), 7.55–7.64 (m, 2H). ^{13}**C NMR** (75.4 MHz, $CDCl_3$) δ 24.48, 25.85, 27.49, 28.78, 32.17, 32.19, 72.41, 78.87, 84.09, 90.76, 127.73, 128.13, 134.06, 138.91, 170.92. **Anal.** Calcd for $C_{20}H_{25}O_4Cl$: C, 68.86; H, 7.22%. Found: C, 68.72; H, 7.28%. $[\alpha]_D^{24}$ +31.8 (*c* 1.70, $CHCl_3$). The ee value (90% ee) was determined by chiral HPLC analysis: CHIRALCEL® OD-3 column, 4.6 mm × 250 mm, Daicel Chemical Industries, hexane/2-propanol = 99:1, 0.5 mL/min, 40 ˚C, 220 nm UV detector, retention time = 24.5 min (major), 26.1 min (minor).

6.28 (*R*)-(+)-*tert*-Butyl 5-(Benzyloxy)-2-(4-bromophenyl)-2-hydroxypent-3-ynoate (3gk)

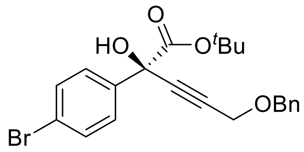

Oil. ^{1}**H NMR** (400 MHz, $CDCl_3$) δ 1.41 (s, 9H), 4.30 (s, 2H), 4.35 (s, 1H), 4.64 (s, 2H), 7.29–7.38 (m, 5H), 7.46–7.57 (m, 4H). ^{13}**C NMR** (100.5 MHz, $CDCl_3$) δ 27.46, 57.12, 71.38, 72.41, 81.70, 84.76, 122.59, 127.87, 127.92, 128.06, 128.42, 131.20, 137.07, 138.72, 170.05. **HRMS–APCI** (*m/z*): $[M-H]^+$ calcd for $C_{22}H_{22}O_4Br$, 429.0707; found, 429.0711. $[\alpha]_D^{26}$ +14.1 (*c* 2.02, $CHCl_3$). The ee value (77% ee) was determined by chiral HPLC analysis: CHIRALCEL® IC column, 4.6 mm × 250 mm, Daicel Chemical Industries, hexane/2-propanol = 95:5, 0.5 mL/min, 40 ˚C, 220 nm UV detector, retention time = 29.2 min (minor), 31.6 min (major).

6.29 (*R*)-(+)-*tert*-Butyl 2-(4-Bromophenyl)-5-(dibenzylamino)-2-hydroxypent-3-ynoate (3gl)

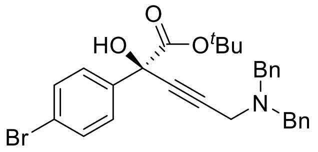

Oil. ^{1}**H NMR** (400 MHz, $CDCl_3$) δ 1.45 (s, 9H), 3.37 (s, 2H), 3.73 (s, 4H), 4.40 (brs, 1H), 7.27 (d, *J* = 7.2 Hz, 2H), 7.33 (t, *J* = 7.2 Hz, 4H), 7.40 (d, *J* = 7.2 Hz, 4H), 7.52 (d, *J* = 8.8 Hz, 2H), 7.61 (d, *J* = 8.8 Hz, 2H). ^{13}**C NMR** (100.5 MHz, $CDCl_3$) δ 27.59, 41.26, 57.66, 72.52, 81.06, 84.03, 84.67, 122.59, 127.22, 127.98, 128.33, 128.96, 131.22, 138.54, 139.18, 170.56. **HRMS–ESI** (*m/z*): $[M+H]^+$ calcd for $C_{29}H_{31}O_3NBr$, 520.1482; found, 520.1484. $[\alpha]_D^{26}$ +24.7 (*c* 1.00, $CHCl_3$). The ee value (71% ee) was determined by chiral HPLC analysis: CHIRALCEL® IC column, 4.6 mm × 250 mm, Daicel Chemical Industries, hexane/2-propanol = 95:5, 0.5 mL/min, 40 ˚C, 220 nm UV detector, retention time = 14.2 min (minor), 18.3 min (major).

6.30 (*S*)-(-)-3-[(*tert*-Butyldimethylsilyl)ethynyl]-3-hydroxy-4,4-dimethyldihydrofuran-2(3H)-one (3rm)

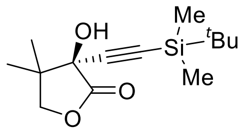

White solid. **Mp** 102–104 °C. ^{1}**H NMR** (400 MHz, $CDCl_3$) δ 0.12 (s, 3H), 0.13 (s, 3H), 0.93 (s, 9H), 1.12 (s, 3H), 1.26 (s, 3H), 2.89 (s, 1H), 4.01 (d, *J* =8.0, 1H), 4.08 (d, *J* = 8.0 Hz, 1H). ^{13}**C NMR** (100.5 MHz, $CDCl_3$) δ –4.97, –4.95, 16.50, 19.56, 20.24, 25.95, 44.26, 74.56, 76.62, 93.94, 99.27, 174.38. **Anal.** Calcd for $C_{14}H_{24}O_3Si$: C, 62.64; H, 9.01%. Found: C, 62.44; H, 8.76%. $[\alpha]_D^{25}$ –2.7 (*c* 1.02, $CHCl_3$). The ee value (76% ee) was determined by chiral HPLC analysis: CHIRALCEL® IC column, 4.6 mm × 250 mm, Daicel Chemical Industries, hexane/2-propanol = 97:3, 0.5 mL/min, 40 ˚C, 220 nm UV detector, retention time = 14.0 min (major), 16.2 min (minor).

7 Details on the Calculations of the Reaction Pathways and Supplemental Calculations on Different Density Functionals

We have initially performed the geometry optimisations without the dispersion correction using the BP86 functional in conjunction with the def2-SVP basis set including density fitting. This level is denoted as DF-BP86/SVP. On this level normal coordinate analysis has been performed and the resulting structures have been confirmed as local minima or transition states. The imaginary modes of the transition states have been followed with intrinsic reaction coordinates (IRC) to describe the reaction pathway.

Single point calculations on the obtained geometries including the dispersion correction, solvent correction (tBuOH) and the larger def2-TZVPP basis set have been performed for a better description of the energies. This level is denoted as DF-BP86-D3(BJ)-PCM/TZVPP//DF-BP86/SVP.

To cross validate these results additional calculations on the M06L,[6] B3LYP,[7] and PBE0[8] density functionals have been conducted. These results reproduce the trend observed with BP86, although the activation energies are in every case higher. The resulting enantiomeric excess is comparable in all cases.

The pure density functional M06L overestimates the relative energies the most, and predicts that the product complex (**PC**) is higher in energy than the association complex (**AC**). This may well be a

result of using zero-damping for the DFT-D3 correction, since Becke-Johnson damping is currently not available.

Table S1. Relative Gibbs energies for different density functionals in kcal mol^{-1} for the calculated reaction pathways of the model system using **L7** as ligand (entry 7, Table 1) and their relative populations in percent in parentheses.

**State**	**R-Paths**				**S-Paths**			
**2a +**	1a (s-trans)		1a (s-cis)		1a (s-trans)		1a (s-cis)	
**BP86**								
**R+1a**	2.3		3.1		2.3		3.1	
**L7-AC**	0.6		1.8		0.0		1.2	
**L7-TS**	4.2	(96.7)	6.3	(2.8)	7.3	(0.5)	9.7	(0.0)
**L7-PC**	-7.6		-7.6		-7.2		-7.3	
**M06L**								
**L7-AC**	0.9		2.4		0.0		1.1	
**L7-TS**	13.7	(98.6)	16.4	(1.1)	17.2	(0.3)	18.1	(0.1)
**L7-PC**	2.5		2.6		2.9		2.8	
**B3LYP**								
**L7-AC**	0.3		1.5		0.0		1.1	
**L7-TS**	7.6	(97.2)	9.8	(2.4)	11.0	(0.3)	13.1	(0.0)
**L7-PC**	-8.4		-8.3		-8.2		-8.3	
**PBE0**								
**L7-AC**	0.4		1.4		0.0		1.1	
**L7-TS**	9.9	(97.1)	12.1	(2.3)	12.9	(0.6)	14.6	(0.0)
**L7-PC**	-2.2		-2.2		-2.3		-2.3	

R - resting state; **AC** - association complex; **TS** - transition state; **PC** - product complex; Gaussian 09; Based on optimized geometries on DF-BP86/SVP; 298.15 K, 1 atm. The single point calculations used the def2-TZVPP basis set and include dispersion corrections and solvent effects.

In order to confirm these results, selected states have been re-optimised including the dispersion correction. We have noticed significant changes in energies and molecular structure. Therefore we have reoptimized all important structures on DF-BP86-D3(BJ)/SVP, which is the level of theory used in the main text. Normal coordinate analysis has been performed to confirm these structures. We have opted to not repeat the IRC calculations, because it is very unlikely, that the overall shape of the potential energy surface changes.

8 Relative Electronic Energies of the Calculated States

Table S2. Relative Gibbs energies (electronic energies in parenthesis) in kcal mol^{-1} for the calculated reaction pathways of the model system using **L2** as ligand (entry 2, Table 1 main text).[a]

	R-Paths				*S*-Paths			
	**1a** (trans)		**1a** (cis)		**1a** (trans)		**1a** (cis)	
**R + 1a**	9.7	(22.9)	12.0	(24.3)	9.7	(22.9)	12.0	(24.3)
**L2-AC**	3.3	(1.4)	0.0	(0.0)	3.7	(0.8)	2.6	(2.8)
**L2-TS**	9.3	(7.7)	8.7	(7.6)	9.6	(10.3)	12.0	(12.3)
**L2-PC**	-7.9	(-10.5)	-9.0	(-11.2)	-5.9	(-7.3)	-7.4	(-9.6)
Pop. (%)	24.5		62.0		13.2		0.2	

[a] Gaussian 09, DF-BP86-D3(BJ)-PCM(tBuOH)/TZVPP//DF-BP86-D3(BJ)/SVP, 298.15 K, 1 atm.

Table S3. Relative Gibbs energies (electronic energies in parenthesis) in kcal mol^{-1} for the calculated reaction pathways of the model system using **L7** as ligand (entry 7, Table 1 main text).[a]

	R-Paths				*S*-Paths			
	**1a** (trans)		**1a** (cis)		**1a** (trans)		**1a** (cis)	
**R + 1a**	10.0	(21.7)	12.3	(23.1)	10.0	(21.7)	12.3	(23.1)
**L7-AC**	0.0	(0.0)	2.6	(0.1)	6.1	(0.3)	5.1	(1.7)
**L7-TS**	9.0	(5.2)	10.2	(6.2)	12.5	(9.5)	15.8	(11.2)
**L7-PC**	-7.1	(-12.3)	-7.3	(-12.3)	-5.3	(-8.7)	-5.4	(-8.7)
Pop. (%)	89.0		10.8		0.2		0.0	

[a] Gaussian 09, DF-BP86-D3(BJ)-PCM(tBuOH)/TZVPP//DF-BP86-D3(BJ)/SVP, 298.15 K, 1 atm.

9 Analyses in Terms of the Quantum Theory of Atoms in Molecules

The quantum theory of atoms in molecules (QTAIM) allows to approximately estimate the strengths of the classical OH···O bond, as well as the non-classical sp^3-CH···O interaction. Since the strength of hydrogen bonds cannot be measured directly, caution should be applied regarding the absolute values. However, a qualitative, relative assessment of these bonds can aid in understanding the importance of these interactions. According to Espinoza *et. al.*,[9] the value of the potential energy density at the bond critical point is proportional to the strength of the hydrogen bond: $E_{\text{H-Bond}} = \frac{1}{2}V(\vec{r}_{\text{BCP}})$.

These values indicate a rather strong classical OH···O bond motive, while the non-classical sp^3-CH···O interaction is less than a tenth of that. This is in line with the previous analysis based on the distances of the respective interactions.

Table S4. Hydrogen bond strengths in the transition states estimated at the bond critical points with QTAIM at the DF-BP86-D3(BJ)-PCM/TZVPP//DF-BP86-D3(BJ)/SVP level of theory in kcal mol^{-1}. Corresponding distances of the interactions given in angstrom.

Transition state	E(OH···O)	d(OH···O)	E(CH···O)	d(CH···O)
**L2-TS-R**(t)	36.4	1.42	2.2	2.36
**L2-TS-R**(c)	38.0	1.41	2.3	2.34
**L2-TS-S**(t)	24.2	1.53	3.4	2.20
**L2-TS-S**(c)	34.1	1.44	2.1	2.39
**L7-TS-R**(t)	37.3	1.41	2.3	2.36
**L7-TS-R**(c)	41.0	1.38	2.5	2.32
**L7-TS-S**(t)	38.8	1.40	3.7	2.21
**L7-TS-S**(c)	34.3	1.43	2.9	2.26

10 Visualisation of Non-covalent Interactions

Non-covalent interactions, like dispersion effects, can be revealed with a method introduced by Erin R. Johnson *et. al.*[10] We have used the program NCIPLOT and VMD to visualise these areas, displayed in green. The analysis is based on the wave function obtained by the DF-BP86-D3(BJ)-PCM/TZVPP//DF-BP86-D3(BJ)/SVP level of theory, and the cut-off values used are s = 0.5 a.u. for the reduced density gradient and ρ < 0.05 a.u. for the electron density.

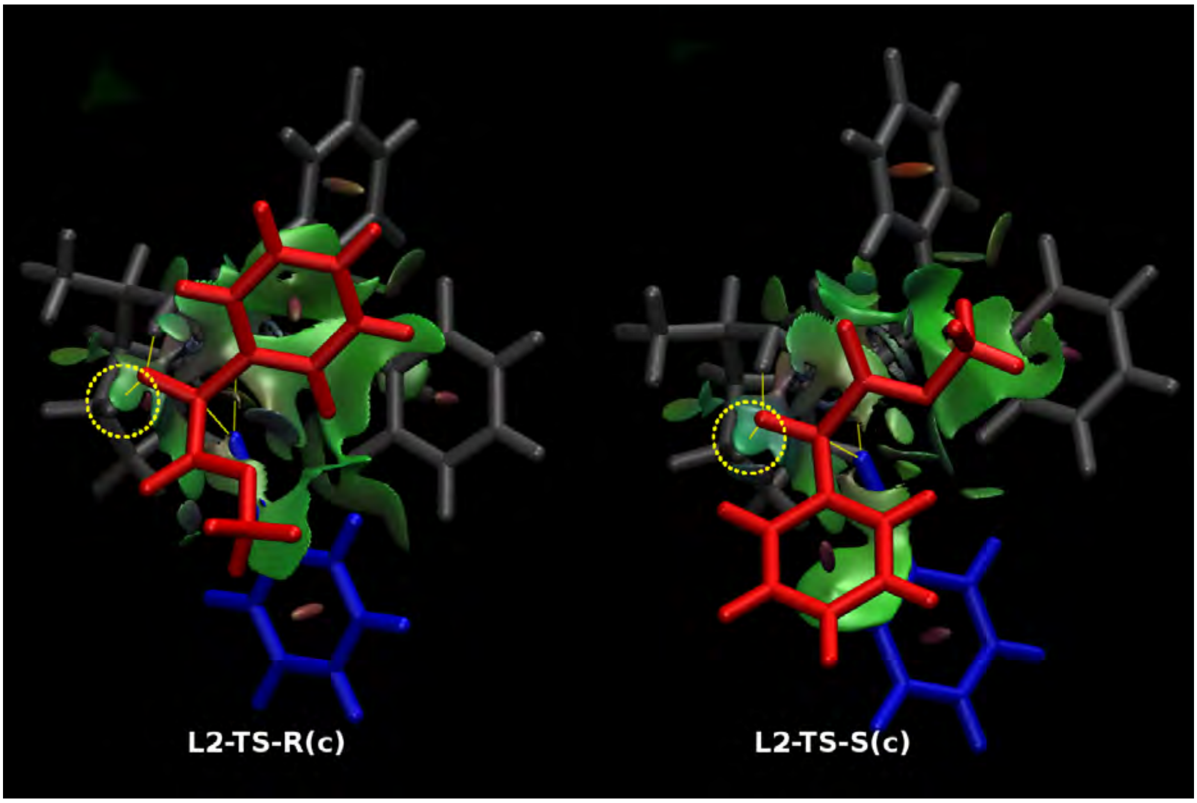

Figure S1. Plot of non-covalent interactions of the transition states structures leading to the respective *R* [left, **L2-TS-R(t)**] or *S* [right, **L2-TS-R(t)**] product complexes of the model system using **L2** as ligand (entry 2, Table 1 main text) and **1a** in *s*-trans conformation (red). The acetylide is highlighted in blue. The non-classical sp^3-CH···O hydrogen bonds are highlighted in the yellow dotted circles.

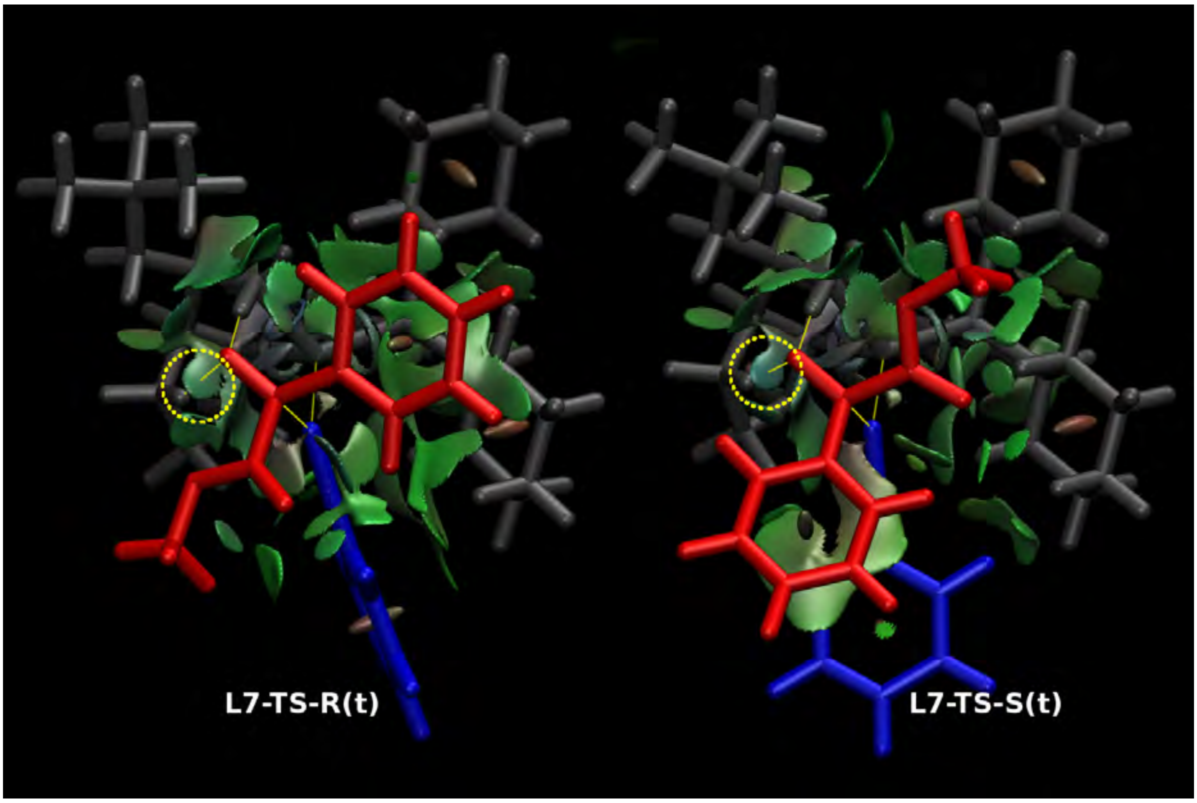

Figure S2. Plot of non-covalent interactions of the transition states structures leading to the respective *R* [left, **L7-TS-R(t)**] or *S* [right, **L7-TS-R(t)**] product complexes of the model system using **L7** as ligand (entry 7, Table 1 main text) and **1a** in *s*-trans conformation (red). The acetylide is highlighted in blue. The non-classical sp^3-CH···O hydrogen bonds are highlighted in the yellow dotted circles.

Non-covalent interactions are also important for aliphatic substrates, to address this point we have exemplarily optimized the transition states for the ketoester **1q** (*s*-trans) leading to the *R* and *S* products. It has been shown that even approximate electron densities (ref. 26 main text) yield excellent results for non-covalent interactions. Based on this calculation we have visualized these interactions, and as can be seen in Figure S3, there are stabilizing interactions between the phosphine moiety and the cyclohexyl moiety of **1q**. The energy difference between those two states is 2.5 kcal mol^{-1} in favor of the *R* transition state at the DF-BP86-D3(BJ)/SVP level of theory.

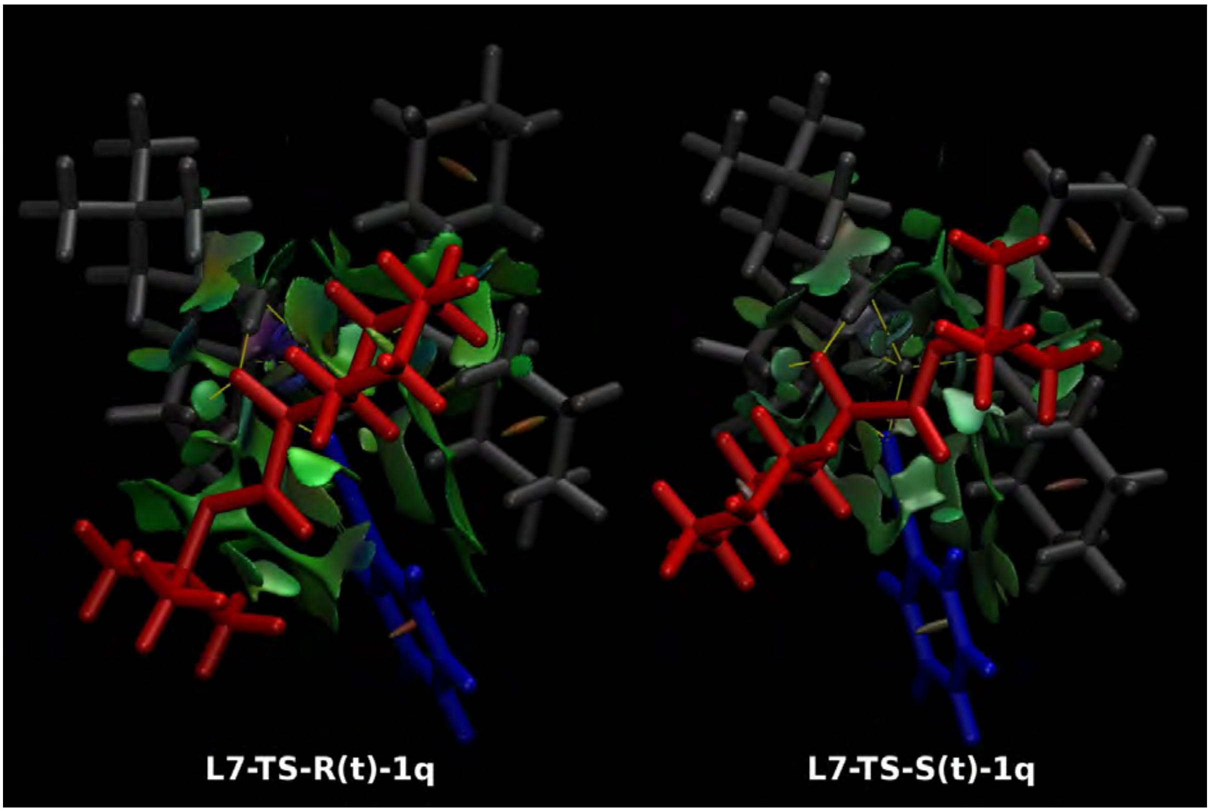

Figure S3. Plot of non-covalent interactions of the transition state structures leading to the *R* [left, **L7-TS-R(t)-1q**] and *S* product complex [right, **L7-TS-S(t)-1q**] of the model system using **L7** as ligand and **1q** in *s*-trans conformation (red). The acetylide is highlighted in blue.

11 Re-optimization of the Aldehyde Model

To allow a meaningful comparison between the different reactions, we have optimized the most stable transition state structures for the following model reaction:

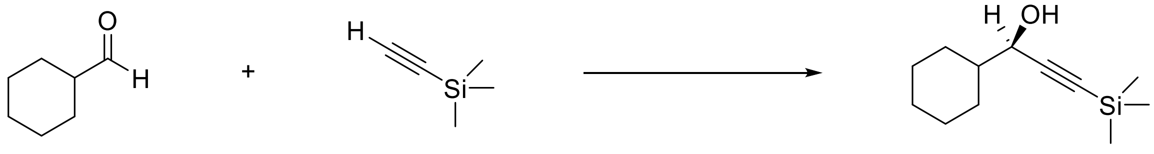

For this purpose, we have adopted model **1a** from reference 3a (main text) and substituted the used ligand (**L1**) with the slightly larger **L2**. The level of theory is analogous to the main text DF-BP86-D3(BJ)-PCM(tBuOH)/TZVPP//DF-BP86-D3(BJ)/SVP.

Table S5. Relative Gibbs energies (electronic energies in parenthesis) in kcal mol^{-1} for the calculated reaction pathways of the aldehyde model system using **L2** as ligand (modified from model 1a ref. 3a, main text).[a]

	R-Paths		*S*-Paths	
**Ald-L2-AC**	0.0	(0.0)	4.7	(4.5)
**Ald-L2-TS**	7.7	(6.6)	12.2	(12.6)
**Ald-L2-PC**	-12.6	(-17.5)	-8.0	(-12.7)
Pop. (%)	99.9		0.1	

[a] Gaussian 09, DF-BP86-D3(BJ)-PCM(tBuOH)/TZVPP//DF-BP86-D3(BJ)/SVP, 298.15 K, 1 atm.

Table S6: Hydrogen bond strengths in the transition states estimated at the bond critical points with QTAIM at the DF-BP86-D3(BJ)-PCM(tBuOH)/TZVPP//DF-BP86-D3(BJ)/SVP level of theory in kcal mol^{-1}. Corresponding distances of the interactions given in angstrom.

Transition state	E(OH···O)	d(OH···O)	E(CH···O)	d(CH···O)
**Ald-L2-TS-R**	29.6	1.47	3.1	2.24
**Ald-L2-TS-S**	29.3	1.48	4.2	2.12

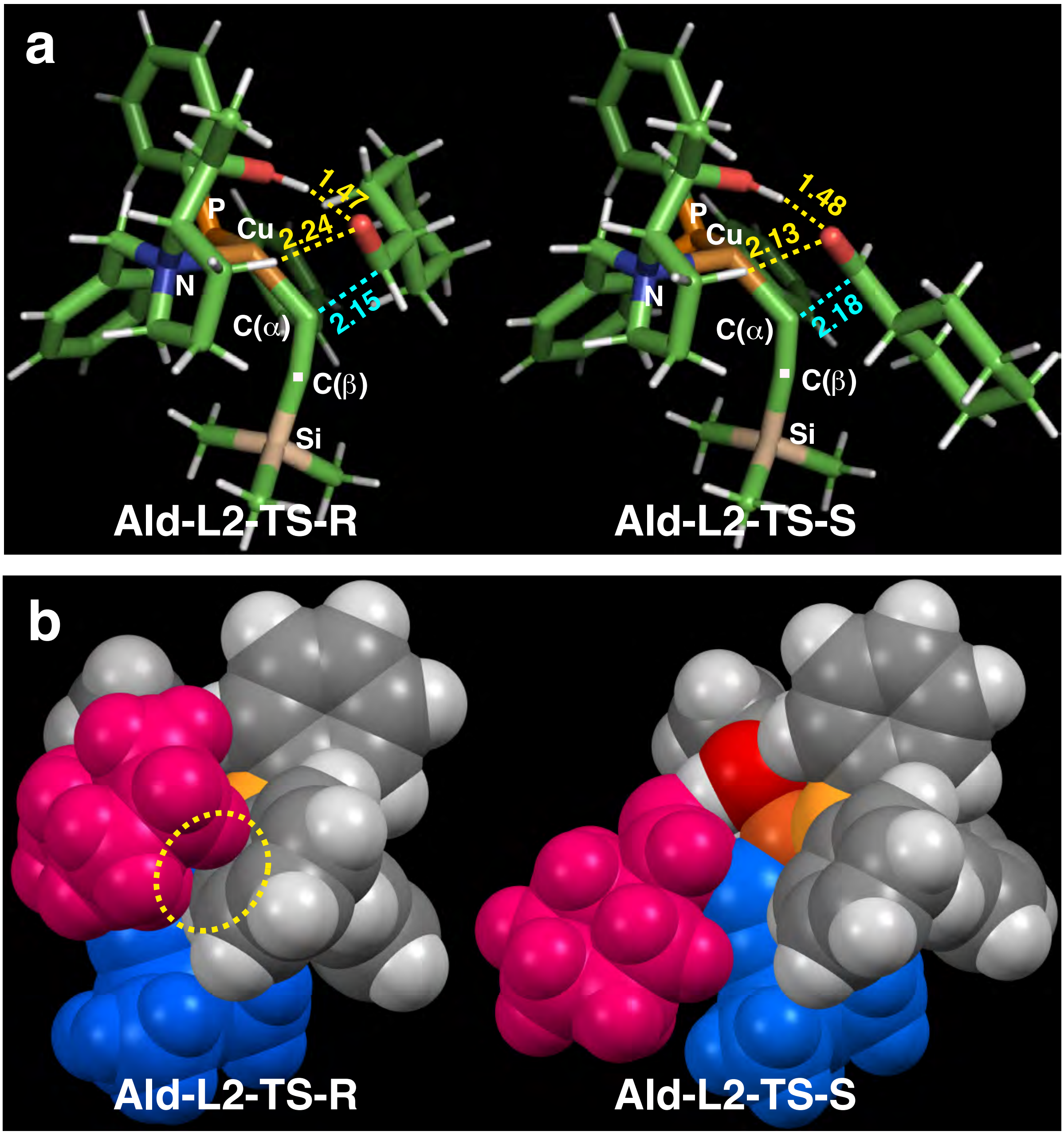

Figure S4. Comparison of the transition state structures leading to the respective *R* [left, **Ald-L2-TS-R**] or *S* [right, **Ald-L2-TS-S**] product complexes (Tables S5, S6). (a) Stick models showing a developing C–C bond (blue dotted line). Atomic distances (in angstrom) of the O–H···O/C–H···O two-point hydrogen-bonds are shown in yellow dotted lines. (b) Space-filling models highlighting dispersive substrate-ligand interactions (yellow dotted circles). Red: aldehyde; blue: acetylide moiety.

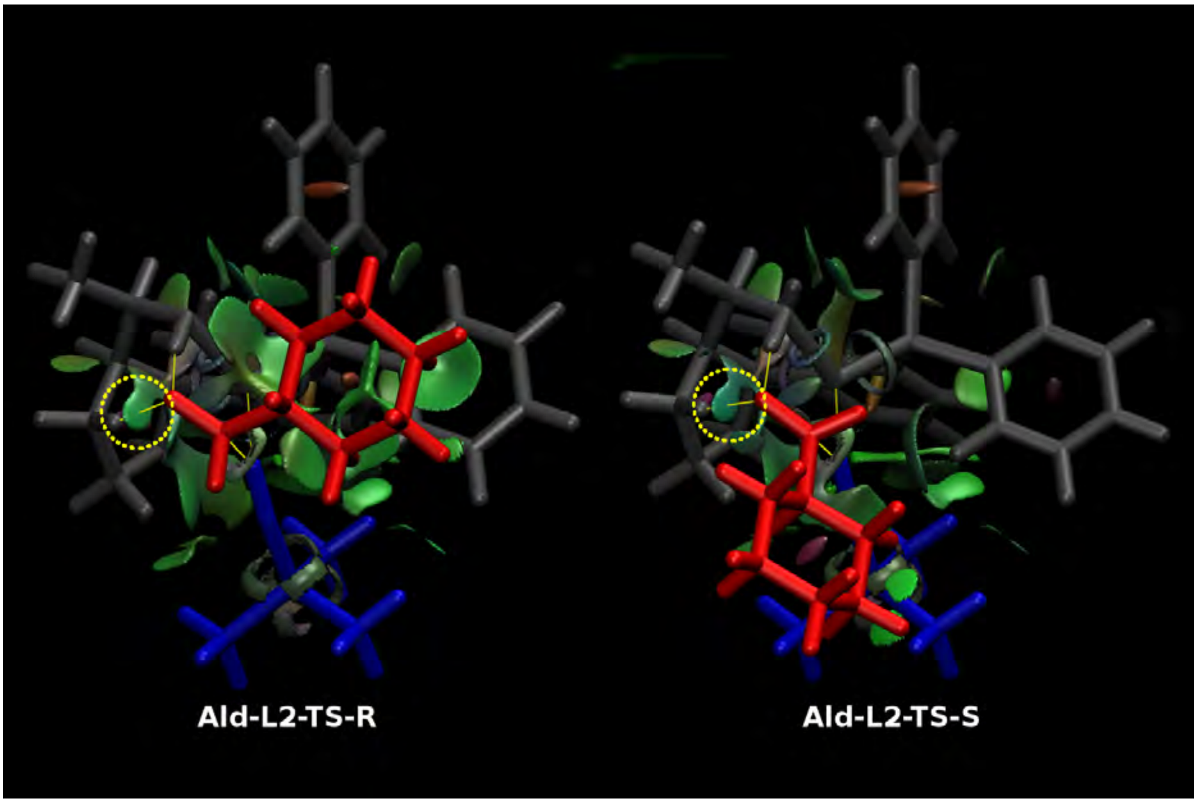

Figure S5. Plot of non-covalent interactions of the transition state structures leading to the respective *R* [left, **Ald-L2-TS-R**] or *S* [right, **Ald-L2-TS-S**] product complexes (Tables S5, S6) using **L2** as ligand. The aldehyde is highlighted in red and the acetylide in blue. The analysis is based on the wave function obtained by the DF-BP86-D3(BJ)-PCM/TZVPP//DF-BP86-D3(BJ)/SVP level of theory, and the cut-off values used are s = 0.5 a.u. for the reduced density gradient and $\rho < 0.05$ a.u. for the electron density. The non-classical sp^3-CH···O hydrogen bonds are highlighted in the yellow dotted circles.

12 Full citation of Gaussian 09

Gaussian 09, Revision D.01, M. J. Frisch, G. W. Trucks, H. B. Schlegel, G. E. Scuseria, M. A. Robb, J. R. Cheeseman, G. Scalmani, V. Barone, B. Mennucci, G. A. Petersson, H. Nakatsuji, M. Caricato, X. Li, H. P. Hratchian, A. F. Izmaylov, J. Bloino, G. Zheng, J. L. Sonnenberg, M. Hada, M. Ehara, K. Toyota, R. Fukuda, J. Hasegawa, M. Ishida, T. Nakajima, Y. Honda, O. Kitao, H. Nakai, T. Vreven, J. A. Montgomery, Jr., J. E. Peralta, F. Ogliaro, M. Bearpark, J. J. Heyd, E. Brothers, K. N. Kudin, V. N. Staroverov, T. Keith, R. Kobayashi, J. Normand, K. Raghavachari, A. Rendell, J. C. Burant, S. S. Iyengar, J. Tomasi, M. Cossi, N. Rega, J. M. Millam, M. Klene, J. E. Knox, J. B. Cross, V. Bakken, C. Adamo, J. Jaramillo, R. Gomperts, R. E. Stratmann, O. Yazyev, A. J. Austin, R. Cammi, C. Pomelli, J. W. Ochterski, R. L. Martin, K. Morokuma, V. G. Zakrzewski, G. A. Voth, P. Salvador, J. J. Dannenberg, S. Dapprich, A. D. Daniels, O. Farkas, J. B. Foresman, J. V. Ortiz, J. Cioslowski, and D. J. Fox, Gaussian, Inc., Wallingford CT, 2013.

13 References

1. (a) T. Ishii, R. Watanabe, T. Moriya, H. Ohmiya, S. Mori and M. Sawamura, *Chem. Eur. J.* 2013, **19**, 13547–13553. (b) Y. Takayama, T. Ishii, H. Ohmiya, T. Iwai, M. C. Schwarzer, S. Mori, T. Taniguchi, K. Monde and M. Sawamura, *Chem. Eur. J.* 2017, **23**, 8400-8404.
2. (a) A. Nakamura, S. Lectard, D. Hashizume, Y. Hamashita and M. Sodeoka, *J. Am. Chem. Soc*. 2010, **132**, 4036–4037. (b) D. Enders, A. Rembiak, B. Anne, *Adv. Synth. Catal.* 2013, **355**, 1937–1942. (c) K. Thai, S. M. Langdon, F. Bilodeau, M. Gravel, *Org. Lett.* 2013, **15**, 2214–2217. (d) S. Khan and Q. N. Ahmed, *Eur. J. Org. Chem.* 2016, 5377–5385.
3. (a) S. Uttiya, L. Miozzo, E. M. Fumagalli, S. Bergantin, R. Ruffo, M. Parravicini, A. Papagni, M. Moret and A. Sassella, *J. Mater. Chem. C* 2014, **2**, 4147–4155. (b) P. P. Tian, S. H. Cai, Q. J. Liang, X. Y. Zhou, Y. H. Xu and T. P. Loh, *Org. Lett.* 2015, **17**, 1636–1639. (c) A. Torrado, S. López, R. Alvarez and A. R. Lera, *Synthesis*, 1995, **3**, 285–293. (d) I. Bernar, B. Fiser, D. B. Ania, E. G. Bengoa and F. P. J. T. Rutjes, *Org. Lett*. 2017, **19**, 4211−4214. (e) J. Deschamps, M. Balog, B. Boury, M. B. Yahia, J. S. Filhol, A. Lee, A. A. Choueiry, T. Barisien, L. Legrand, M. Schott and S. G. Dutremez, *Chem. Mater*. 2010, **22**, 3961–3982.
4. B. Jiang, Z. Chen and X. Tang, *Org. Lett*. 2002, **4**, 3451–3453.
5. R. Infante, A. Gago, J. Nieto and C. Andrés, *Adv. Synth. Catal.* 2012, **354**, 2797–2804.
6. Y. Zhao, N. E. Schultz, and D. G. Truhlar, *Theor. Chem. Acc.* 2008, **120**, 215–241.
7. (a) P. J. Stephens, F. J. Devlin, C. F. Chabalowski, and M. J. Frisch, *J. Phys. Chem.* 1994, **98**, 11623–11627. (b) A. D. Becke, *J. Chem. Phys.* 1993, **93**, 5648. (c) C. Lee, W. Yang, R. G. Parr, *Phys. Rev. B* 1988, **37**, 785–789. (d) S. H. Vosko, L. Wilk, and M. Nusair, *Can. J. Phys.* 1980, **58**, 1200–1211.
8. (a) C. Adamo and V. Barone, *J. Chem. Phys.* 1999, **110**, 6158. (b) K. Burke, M. Ernzerhof, and J. P. Perdew, *Chem. Phys. Lett.* 1997, **265**, 115–120.
9. E. Espinosa, E. Molins and C. Lecomte, *Chem. Phys. Lett.,* 1998, **285**, 170–173.
10. E. R. Johnson, S. Keinan, P. Mori-Sanchez, J. Contreras-Garcia, A. J. Cohen and W. Yang, *J. Am. Chem. Soc.*, 2010, **132**, 6498-6506.

14 Absolute Electronic Energies and Coordinates of the Optimised structures

Listing S1: Absolute electronic energies of the calculated structures at the DF-BP86-D3(BJ)-PCM/TZVPP//DF-BP86-D3(BJ)/SVP level of theory in hartree (a.u.).

```
1a(s-cis)              -573.704287305
1a(s-trans)            -573.706479453
L2-R                   -3390.23492966
L2-AC-R(s-trans)       -3963.97579681
L2-TS-R(s-trans)       -3963.96561853
L2-PC-R(s-trans)       -3963.99467272
L2-AC-R(s-cis)         -3963.97795675
L2-TS-R(s-cis)         -3963.96587452
L2-PC-R(s-cis)         -3963.99576293
L2-AC-S(s-trans)       -3963.98917391
L2-TS-S(s-trans)       -3963.96147609
L2-PC-S(s-trans)       -3963.97668977
L2-AC-S(s-cis)         -3963.99318992
L2-TS-S(s-cis)         -3963.95840522
L2-PC-S(s-cis)         -3963.97350579
L7-R                   -3554.81677081
L7-AC-R(s-trans)       -4128.55783387
L7-TS-R(s-trans)       -4128.54951111
L7-PC-R(s-trans)       -4128.57750878
L7-AC-R(s-cis)         -4128.55762380
L7-TS-R(s-cis)         -4128.54794453
L7-PC-R(s-cis)         -4128.57750954
L7-AC-S(s-trans)       -4128.55729900
L7-TS-S(s-trans)       -4128.54274797
L7-PC-S(s-trans)       -4128.57169613
L7-AC-S(s-cis)         -4128.55507890
L7-TS-S(s-cis)         -4128.53991424
L7-PC-S(s-cis)         -4128.57165486
```

Listing S2: Cartesian coordinates of the calculated structures optimized at the DF-BP86-D3(BJ)/SVP level of theory in angstrom.

```
1a(s-cis)
C    -0.15986    0.00007    0.09620
C    -0.13831    0.00004    1.59794
O     0.87783   -0.00003   -0.55185
C    -1.46302   -0.00001   -0.77245
O    -2.62286    0.00001   -0.06884
O    -1.41451   -0.00001   -1.98295
C    -3.82237   -0.00001   -0.86766
H    -4.66390    0.00001   -0.15216
H    -3.85762    0.89938   -1.51477
H    -3.85762   -0.89944   -1.51472
C    -1.27085    0.00003    2.44770
C    -1.10778    0.00000    3.84195
C     0.17731   -0.00002    4.40932
C     1.31091   -0.00001    3.57404
C     1.15337    0.00001    2.18475
H    -2.27660    0.00005    2.01303
H    -1.99673   -0.00000    4.49196
H     0.29763   -0.00004    5.50429
H     2.32100   -0.00003    4.01274
H     2.02198    0.00002    1.50948

1a(s-trans)
C    -0.14783    0.00002    0.05372
C    -0.12326    0.00001    1.54956
O     0.87933    0.00002   -0.61341
C    -1.51335    0.00004   -0.69991
O    -1.29601    0.00001   -2.02707
O    -2.62531    0.00001   -0.19612
C    -2.47975   -0.00001   -2.84118
H    -2.12778   -0.00003   -3.88797
H    -3.09615   -0.89906   -2.63573
H    -3.09616    0.89903   -2.63576
C    -1.27131    0.00001    2.37962
C    -1.12557   -0.00000    3.77564
C     0.15311   -0.00001    4.35844
C     1.29959   -0.00001    3.53989
C     1.16195   -0.00000    2.14857
H    -2.26710    0.00002    1.91888
H    -2.02218    0.00000    4.41496
H     0.25918   -0.00002    5.45496
H     2.30276   -0.00002    3.99417
H     2.03883   -0.00000    1.48374

L2-R
Cu    0.50418    0.32445    0.88816
C    -1.18560    0.97451    0.36016
C    -2.32123    1.23352   -0.09342
H    -0.64460   -0.85932   -0.71903
O     0.21775   -1.33273   -0.85123
C     0.21944   -2.60060   -0.19565
H     1.28975   -2.90672   -0.20541
C    -0.23988   -2.52690    1.27666
H    -0.05193   -3.54166    1.71686
```

N	0.51794	-1.52910	2.08202
P	2.64182	0.73202	1.10529
C	-1.72586	-2.15251	1.49747
H	-2.12165	-1.56317	0.64561
H	-2.34210	-3.06819	1.58949
C	-1.74699	-1.27812	2.77696
H	-2.46053	-1.64673	3.53969
H	-2.02515	-0.24104	2.50592
C	-0.30526	-1.31917	3.29594
H	0.01372	-0.39614	3.81927
H	-0.15365	-2.17800	3.99995
C	1.88692	-1.98257	2.40548
H	2.43339	-2.13573	1.45213
H	1.83699	-2.97694	2.91771
C	3.07194	0.26162	2.83429
C	3.73795	1.13813	3.71280
C	2.64810	-1.01729	3.29600
C	4.00776	0.75667	5.03831
H	4.05065	2.12961	3.35071
C	2.93784	-1.38351	4.62321
C	3.61317	-0.51041	5.49368
H	4.53093	1.45261	5.71271
H	2.61703	-2.37517	4.98222
H	3.82327	-0.82057	6.52905
C	3.26335	2.44753	0.92309
C	4.61688	2.77342	0.69570
C	2.29929	3.47381	1.02188
C	5.00340	4.11933	0.58760
H	5.36694	1.97374	0.59394
C	2.69289	4.81798	0.92322
H	1.23938	3.19703	1.15608
C	4.04417	5.14157	0.70704
H	6.05979	4.37227	0.40486
H	1.93766	5.61575	1.00038
H	4.35091	6.19579	0.61898
C	3.79890	-0.31523	0.12864
C	5.06030	-0.71534	0.62139
C	3.37152	-0.75363	-1.14398
C	5.89581	-1.52765	-0.16261
H	5.37924	-0.40274	1.62852
C	4.21371	-1.56370	-1.92390
H	2.36173	-0.49050	-1.49680
C	5.47566	-1.94846	-1.43743
H	6.87899	-1.83785	0.22542
H	3.87548	-1.90589	-2.91466
H	6.13121	-2.58821	-2.04914
C	-3.62436	1.53601	-0.59000
C	-4.54839	2.29141	0.18556
C	-4.04515	1.08544	-1.87290
C	-5.83018	2.57866	-0.30222
C	-5.32947	1.37580	-2.35216
C	-6.23091	2.12332	-1.57202
H	-4.23556	2.64739	1.17901
H	-3.33929	0.50403	-2.48603
H	-6.52773	3.16602	0.31667
H	-5.63166	1.01596	-3.34903
H	-7.23937	2.35063	-1.95182
C	-0.58771	-3.63523	-0.98477
H	-0.20019	-3.70398	-2.02012
H	-0.52606	-4.64069	-0.51817
H	-1.65748	-3.34504	-1.03750

L2-AC-R(s-trans)

C	-1.88679	-1.37882	-2.31649
O	-1.72707	-2.61998	-2.32754
Cu	-0.20894	-0.90121	0.03594
C	-1.75035	0.12386	0.37046
C	-2.57297	1.00385	0.69868
H	-0.32211	-2.84871	-1.62384
O	0.46614	-2.62657	-1.02519
C	0.78931	-3.72599	-0.16445
H	1.87718	-3.61955	0.04729
C	0.02303	-3.59279	1.16478
H	0.40879	-4.38106	1.86480
N	0.23752	-2.25471	1.76367
P	1.64712	0.35349	0.27561
C	-1.51448	-3.69258	1.04844
H	-1.85100	-3.37222	0.04152
H	-1.85153	-4.73774	1.19457
C	-2.07125	-2.71948	2.12176
H	-2.70277	-3.22976	2.87556
H	-2.67290	-1.92699	1.63723
C	-0.82771	-2.09510	2.77526
H	-0.95703	-1.02270	3.02102
H	-0.54099	-2.64081	3.71088
C	1.59191	-2.05001	2.30173
H	2.32160	-2.20967	1.48050
H	1.81044	-2.82019	3.08533
C	1.85145	0.50434	2.10735
C	2.02048	1.75645	2.73224
C	1.79193	-0.67278	2.90548
C	2.13405	1.85767	4.12874
H	2.06256	2.66571	2.11425
C	1.91506	-0.55139	4.30221
C	2.08338	0.69956	4.91863
H	2.26572	2.84619	4.59604
H	1.86959	-1.46425	4.91846
H	2.17090	0.76642	6.01420
C	1.59348	2.09275	-0.29265
C	2.62427	2.68935	-1.04573
C	0.41195	2.82129	-0.01782
C	2.47825	4.00672	-1.51492
H	3.53809	2.11974	-1.27345
C	0.27957	4.13957	-0.47693
H	-0.41372	2.33535	0.52730
C	1.31065	4.73558	-1.22878
H	3.28524	4.46669	-2.10717
H	-0.64481	4.69710	-0.25909
H	1.20061	5.76795	-1.59656
C	3.29406	-0.26623	-0.25865
C	4.49053	0.06405	0.41447
C	3.33401	-1.12718	-1.37730
C	5.71613	-0.45206	-0.03657
H	4.45632	0.72487	1.29515
C	4.56416	-1.63454	-1.82821
H	2.39203	-1.40738	-1.87337
C	5.75481	-1.29864	-1.16002
H	6.64759	-0.19276	0.49136
H	4.59133	-2.30449	-2.70225
H	6.71750	-1.70307	-1.51082
C	-3.48823	2.05667	1.00051
C	-4.42227	2.50649	0.02388
C	-3.47673	2.70173	2.26862
C	-5.30340	3.55751	0.31324
C	-4.36239	3.75205	2.54598
C	-5.28084	4.18659	1.57216
H	-4.43689	2.00772	-0.95757
H	-2.75506	2.36146	3.02738
H	-6.01850	3.89185	-0.45583
H	-4.33675	4.23907	3.53429
H	-5.97566	5.01198	1.79374
C	-0.87052	-0.45284	-2.88163
C	0.17175	-1.01870	-3.65658
C	-0.88859	0.94808	-2.66672
C	1.19146	-0.21144	-4.17565
C	0.12544	1.74979	-3.20028

C	1.17243	1.17554	-3.94414
H	0.16436	-2.10331	-3.83870
H	-1.67695	1.38751	-2.04400
H	2.00281	-0.66273	-4.76783
H	0.11953	2.83240	-3.00662
H	1.97763	1.81386	-4.33989
C	-3.25003	-0.82835	-1.88258
O	-3.75312	0.18572	-2.33997
O	-3.85207	-1.67683	-1.03006
C	-5.09420	-1.21680	-0.47793
H	-4.89600	-0.38057	0.22419
H	-5.77805	-0.86786	-1.27712
H	-5.52576	-2.07843	0.06227
C	0.55159	-5.06301	-0.85986
H	1.16247	-5.13291	-1.78183
H	0.82693	-5.90750	-0.19544
H	-0.51568	-5.17752	-1.14050

L2-TS-R(s-trans)

C	0.97682	0.20877	-4.88620
C	0.62881	-1.14982	-4.95533
C	-0.43265	-1.64456	-4.17867
C	-1.15669	-0.79139	-3.32187
C	-0.80231	0.57329	-3.25929
C	0.25550	1.06643	-4.03553
C	-2.31137	-1.41740	-2.53605
C	-3.68556	-0.78755	-2.85632
O	-4.68468	-1.50191	-2.30053
C	-5.99126	-0.93402	-2.46123
O	-2.30299	-2.69722	-2.37953
O	-3.85621	0.21206	-3.53601
C	-2.15789	-0.44668	-0.84394
C	-2.90385	0.49214	-0.46267
C	-3.65703	1.65125	-0.14447
C	-3.98771	1.98533	1.19738
C	-4.65348	3.18447	1.48492
C	-5.02538	4.05973	0.44682
C	-4.72513	3.72488	-0.88763
C	-4.04087	2.54089	-1.18896
Cu	-0.42970	-1.17192	-0.35131
P	1.17145	0.28848	-0.06575
C	2.75056	-0.01541	-0.94464
C	3.98487	0.53607	-0.53887
C	5.14572	0.27148	-1.28239
C	5.07801	-0.54163	-2.43007
C	3.85059	-1.09735	-2.82939
C	2.68672	-0.84331	-2.08545
O	-0.10582	-3.02083	-1.29186
C	-0.01250	-4.07105	-0.33671
C	-0.57062	-5.38227	-0.89143
N	-0.24102	-2.32510	1.45086
C	-0.68801	-3.66832	0.99441
C	-2.22195	-3.51500	0.93092
C	-2.56593	-2.41452	1.96952
C	-1.20359	-1.92927	2.49925
C	1.17257	-2.25841	1.87004
C	1.57203	-0.91914	2.47206
C	1.62794	0.29242	1.72472
C	1.99266	1.49257	2.37059
C	2.31654	1.51173	3.73658
C	2.27329	0.31980	4.47546
C	1.90167	-0.87698	3.84143
C	0.73429	2.02918	-0.41075
C	1.42592	2.82835	-1.34266
C	0.93405	4.10414	-1.67097
C	-0.24568	4.58521	-1.07677
C	-0.93357	3.79315	-0.13832
C	-0.44771	2.52042	0.18959
H	-1.04300	-3.02722	-1.81742
H	1.07560	-4.20981	-0.13391
H	-0.40628	-4.42935	1.76970
H	-2.52802	-3.19530	-0.08849
H	-2.72418	-4.47906	1.14264
H	-3.19748	-2.79191	2.79782
H	-3.10664	-1.58432	1.47609
H	-1.15180	-0.83509	2.66841
H	-0.94041	-2.43359	3.46385
H	1.80256	-2.48215	0.98334
H	1.37890	-3.05589	2.62787
H	2.01172	2.42758	1.78924
H	2.60121	2.45912	4.22054
H	1.85812	-1.81165	4.42413
H	2.52190	0.31886	5.54814
H	2.33935	2.44440	-1.82172
H	-0.99850	1.89087	0.90452
H	1.47521	4.72387	-2.40352
H	-1.86506	4.15483	0.32399
H	-0.63421	5.57932	-1.34781
H	4.03489	1.16923	0.36122
H	1.72375	-1.28925	-2.38117
H	6.10969	0.70053	-0.96600
H	3.79259	-1.73576	-3.72455
H	5.99108	-0.74620	-3.01146
H	-3.69568	1.29753	2.00570
H	-3.79979	2.27022	-2.22775
H	-4.88972	3.43866	2.53046
H	-5.01872	4.40265	-1.70489
H	-5.55137	4.99920	0.67781
H	-0.71786	-2.70588	-4.22202
H	-1.34136	1.24418	-2.57717
H	1.18576	-1.83210	-5.61744
H	0.52179	2.13161	-3.96586
H	1.81359	0.59940	-5.48647
H	-6.04931	0.05380	-1.95783
H	-6.23726	-0.79578	-3.53384
H	-6.69350	-1.64584	-1.99044
H	-0.01038	-5.67744	-1.80062
H	-0.48674	-6.20145	-0.14728
H	-1.63805	-5.26755	-1.16887

L2-PC-R(s-trans)

C	-2.27486	-1.12733	-1.89272
O	-2.13954	-2.51656	-1.82646
Cu	-0.37527	-0.81366	0.33273
C	-2.17586	-0.48772	-0.55162
C	-2.17157	0.24722	0.46868
H	-1.21043	-2.65976	-1.37330
O	0.11868	-2.46103	-0.67203
C	0.67270	-3.39700	0.20411
H	1.76858	-3.18070	0.34540
C	0.01735	-3.29726	1.60424
H	0.53404	-3.99886	2.30913
N	0.13749	-1.90818	2.15469
P	1.39651	0.59543	0.38744
C	-1.50773	-3.55198	1.61511
H	-1.92312	-3.36272	0.60456
H	-1.72599	-4.60522	1.87869
C	-2.09620	-2.54712	2.64182
H	-2.69893	-3.03661	3.43195
H	-2.74954	-1.80936	2.13559
C	-0.86735	-1.84769	3.23680
H	-1.05043	-0.80163	3.54874
H	-0.48358	-2.40662	4.12918
C	1.50223	-1.58500	2.61552
H	2.19898	-1.79243	1.77796
H	1.78533	-2.27224	3.45185

C	1.71439	0.94269	2.16936
C	1.85728	2.25649	2.66038
C	1.66249	-0.15005	3.08381
C	1.93652	2.50402	4.04095
H	1.89207	3.09585	1.94944
C	1.75195	0.12017	4.46196
C	1.88037	1.43341	4.94667
H	2.04511	3.53744	4.40591
H	1.71246	-0.72376	5.17004
H	1.93845	1.61640	6.03085
C	1.35449	2.24784	-0.40284
C	2.39785	2.72886	-1.22018
C	0.18075	3.02104	-0.24792
C	2.27391	3.97219	-1.86294
H	3.30497	2.12280	-1.36264
C	0.06591	4.26563	-0.88378
H	-0.64795	2.64354	0.36960
C	1.11125	4.74419	-1.69582
H	3.09247	4.33785	-2.50284
H	-0.85256	4.85906	-0.75368
H	1.01556	5.71678	-2.20358
C	2.95497	-0.15984	-0.21269
C	4.21973	0.17855	0.31546
C	2.84831	-1.11086	-1.25189
C	5.37739	-0.42113	-0.20594
H	4.29252	0.91058	1.13578
C	4.01373	-1.69852	-1.77181
H	1.84769	-1.41908	-1.60326
C	5.27500	-1.35495	-1.25423
H	6.36475	-0.15915	0.20641
H	3.93146	-2.44193	-2.58031
H	6.18488	-1.82387	-1.66154
C	-2.60111	1.22556	1.42716
C	-3.86865	1.84356	1.25770
C	-1.77495	1.62163	2.50800
C	-4.28877	2.83103	2.15806
C	-2.20561	2.61164	3.40198
C	-3.46327	3.21731	3.23268
H	-4.49093	1.53813	0.40273
H	-0.78531	1.15822	2.62088
H	-5.27124	3.30957	2.01966
H	-1.54795	2.91194	4.23247
H	-3.80147	3.99479	3.93556
C	-1.17656	-0.45212	-2.77025
C	-0.31890	-1.25871	-3.53541
C	-1.00039	0.94425	-2.75649
C	0.74033	-0.67174	-4.24748
C	0.05308	1.52931	-3.47458
C	0.93524	0.72011	-4.21133
H	-0.47820	-2.34560	-3.53202
H	-1.67469	1.57219	-2.15445
H	1.42631	-1.30917	-4.82798
H	0.19948	2.61930	-3.43516
H	1.77758	1.17581	-4.75523
C	-3.64764	-0.75033	-2.51117
O	-4.34820	0.17417	-2.13560
O	-3.93226	-1.54308	-3.55862
C	-5.15241	-1.23130	-4.24906
H	-6.02421	-1.33020	-3.57058
H	-5.12826	-0.19324	-4.63948
H	-5.22503	-1.95667	-5.07920
C	0.56673	-4.81824	-0.36344
H	1.07100	-4.85644	-1.34967
H	1.04219	-5.56933	0.30284
H	-0.49509	-5.10265	-0.51632

L2-AC-R(s-cis)

C	-2.19615	-1.12960	-2.00715
O	-2.06249	-2.32913	-1.71038
Cu	-0.05139	-0.73194	0.51563
C	-1.72106	0.12674	0.51229
C	-2.75489	0.83290	0.52048
H	-0.60421	-2.65271	-1.09107
O	0.29888	-2.57428	-0.64118
C	0.55664	-3.67830	0.22806
H	1.66653	-3.72877	0.31012
C	-0.01181	-3.40649	1.63494
H	0.36145	-4.21759	2.31549
N	0.43424	-2.09036	2.15882
P	1.87368	0.34722	0.47028
C	-1.55090	-3.31715	1.71531
H	-1.96320	-2.94112	0.75639
H	-1.99469	-4.31439	1.90379
C	-1.84253	-2.29820	2.84859
H	-2.40063	-2.74908	3.69277
H	-2.43247	-1.45052	2.45061
C	-0.45728	-1.80983	3.30612
H	-0.42606	-0.73107	3.55533
H	-0.10170	-2.38125	4.20212
C	1.86533	-2.03383	2.50899
H	2.45372	-2.27182	1.59857
H	2.10167	-2.82251	3.26834
C	2.34633	0.48525	2.25274
C	2.71572	1.71542	2.83289
C	2.29054	-0.68484	3.06251
C	3.04192	1.80057	4.19678
H	2.74584	2.61853	2.20417
C	2.62829	-0.57982	4.42471
C	3.00269	0.64815	4.99601
H	3.32943	2.77076	4.63168
H	2.58664	-1.48544	5.05196
H	3.25674	0.70237	6.06603
C	1.81058	2.09097	-0.09094
C	2.80143	2.70397	-0.88312
C	0.63644	2.80700	0.24111
C	2.62538	4.02507	-1.33167
H	3.70644	2.14285	-1.16175
C	0.47706	4.13141	-0.19103
H	-0.16496	2.30001	0.80471
C	1.46848	4.74341	-0.98242
H	3.39968	4.49645	-1.95765
H	-0.43705	4.68475	0.07703
H	1.33507	5.77905	-1.33282
C	3.40557	-0.35666	-0.26787
C	4.70125	0.02758	0.14145
C	3.24924	-1.35670	-1.25200
C	5.82962	-0.56309	-0.44989
H	4.82284	0.78505	0.93224
C	4.38232	-1.94414	-1.84048
H	2.23321	-1.68064	-1.52704
C	5.67112	-1.54569	-1.44505
H	6.83909	-0.25973	-0.12969
H	4.25699	-2.72504	-2.60746
H	6.55794	-2.00982	-1.90509
C	-3.92553	1.63841	0.46003
C	-5.19263	1.12755	0.85923
C	-3.86874	2.96662	-0.05107
C	-6.34788	1.91277	0.74555
C	-5.02887	3.74340	-0.16441
C	-6.27520	3.22196	0.23230
H	-5.24820	0.09565	1.23453
H	-2.89270	3.36783	-0.36529
H	-7.32038	1.49664	1.05360
H	-4.96365	4.76682	-0.56759
H	-7.18644	3.83383	0.14209
C	-1.06602	-0.33714	-2.57189

C	-0.04765	-1.05101	-3.24586
C	-0.94752	1.06625	-2.43524
C	1.07704	-0.38383	-3.74902
C	0.18043	1.72709	-2.93231
C	1.19894	1.00593	-3.58141
H	-0.14929	-2.13988	-3.36136
H	-1.72100	1.61816	-1.88949
H	1.86609	-0.94973	-4.26781
H	0.28500	2.81219	-2.78439
H	2.09241	1.53164	-3.95246
C	-3.64017	-0.58897	-1.95489
O	-4.55517	-1.19084	-1.43140
O	-3.77287	0.55553	-2.66479
C	-5.11533	1.06418	-2.77541
H	-5.75276	0.33920	-3.32221
H	-5.55189	1.24366	-1.77492
H	-5.03274	2.01101	-3.33730
C	0.04899	-4.99004	-0.36712
H	0.53133	-5.17926	-1.34674
H	0.27420	-5.84525	0.30280
H	-1.04869	-4.95317	-0.52359

L2-TS-R(s-cis)

C	-2.38912	-1.29753	-2.45598
O	-2.33786	-2.57637	-2.29882
Cu	-0.45836	-1.03474	-0.30827
C	-2.21157	-0.34335	-0.75659
C	-3.01558	0.54028	-0.36078
H	-1.06180	-2.87841	-1.78982
O	-0.10445	-2.86208	-1.29449
C	0.04429	-3.92587	-0.36342
H	1.14107	-4.03731	-0.19166
C	-0.60659	-3.56720	0.99234
H	-0.28547	-4.33511	1.74509
N	-0.17943	-2.22223	1.46475
P	1.22497	0.34934	-0.11366
C	-2.14509	-3.45159	0.97259
H	-2.48777	-3.12665	-0.03343
H	-2.61719	-4.43072	1.18423
C	-2.48758	-2.37426	2.03589
H	-3.09654	-2.77508	2.87005
H	-3.05086	-1.54566	1.56567
C	-1.12420	-1.87212	2.54567
H	-1.09165	-0.78182	2.74128
H	-0.82668	-2.39614	3.48984
C	1.24101	-2.14067	1.85764
H	1.85674	-2.36420	0.96106
H	1.46740	-2.93254	2.61567
C	1.69630	0.40251	1.67101
C	2.06355	1.61424	2.29224
C	1.63967	-0.79455	2.44188
C	2.38539	1.65856	3.65860
H	2.09234	2.53686	1.69224
C	1.96982	-0.72752	3.80939
C	2.34067	0.48167	4.42078
H	2.67190	2.61466	4.12405
H	1.92749	-1.65160	4.40887
H	2.58906	0.50162	5.49331
C	0.97985	2.10911	-0.55202
C	1.87902	2.84254	-1.35158
C	-0.22515	2.71070	-0.12125
C	1.57963	4.16907	-1.70855
H	2.80821	2.36980	-1.70444
C	-0.51290	4.03814	-0.46990
H	-0.94571	2.12344	0.46979
C	0.38725	4.76917	-1.26832
H	2.28381	4.73628	-2.33769
H	-1.45347	4.49669	-0.12750
H	0.15545	5.80801	-1.55132
C	2.76529	-0.16141	-0.96675
C	4.05335	0.21634	-0.52969
C	2.61630	-0.97397	-2.11147
C	5.18521	-0.20112	-1.24825
H	4.16638	0.83381	0.37577
C	3.75250	-1.37951	-2.83007
H	1.61152	-1.29879	-2.42366
C	5.03470	-0.99411	-2.40167
H	6.19072	0.09225	-0.90712
H	3.63022	-2.00668	-3.72682
H	5.92475	-1.31947	-2.96337
C	-3.88338	1.60713	-0.02069
C	-4.81123	1.50900	1.05386
C	-3.84953	2.81169	-0.78268
C	-5.66081	2.57967	1.35851
C	-4.71206	3.87069	-0.47649
C	-5.61729	3.76301	0.59690
H	-4.84895	0.57693	1.63739
H	-3.14229	2.88338	-1.62168
H	-6.37181	2.49041	2.19496
H	-4.67963	4.79203	-1.07962
H	-6.29150	4.59977	0.83746
C	-1.26021	-0.65608	-3.26969
C	-0.58921	-1.49106	-4.18699
C	-0.85439	0.69052	-3.15725
C	0.46707	-0.99506	-4.97031
C	0.20184	1.18501	-3.93448
C	0.86934	0.34449	-4.84452
H	-0.90561	-2.54159	-4.26536
H	-1.35275	1.34163	-2.42785
H	0.98190	-1.66386	-5.67867
H	0.51442	2.23332	-3.81427
H	1.70688	0.73223	-5.44556
C	-3.82807	-0.78833	-2.73094
O	-4.82717	-1.43793	-2.50894
O	-3.83816	0.45629	-3.28034
C	-5.14533	0.99773	-3.51734
H	-5.72879	0.33800	-4.19155
H	-5.70103	1.10900	-2.56386
H	-4.98653	1.98705	-3.98358
C	-0.49111	-5.24271	-0.92769
H	0.05049	-5.50259	-1.85871
H	-0.36066	-6.07344	-0.20325
H	-1.56927	-5.15535	-1.17167

L2-PC-R(s-cis)

C	-2.17961	-1.09152	-2.14562
O	-2.09309	-2.47915	-2.04650
Cu	-0.42581	-0.81961	0.20322
C	-2.17368	-0.44102	-0.80518
C	-2.22014	0.24517	0.24851
H	-1.18852	-2.64061	-1.55087
O	0.10234	-2.46331	-0.78706
C	0.59467	-3.41864	0.10397
H	1.68313	-3.21822	0.31087
C	-0.13912	-3.33020	1.46535
H	0.32859	-4.04679	2.18904
N	-0.03256	-1.94974	2.04043
P	1.36590	0.55156	0.38173
C	-1.66518	-3.56652	1.38787
H	-2.02225	-3.36682	0.35741
H	-1.91000	-4.61848	1.63233
C	-2.29882	-2.56128	2.38713
H	-2.95738	-3.04718	3.13370
H	-2.90755	-1.80782	1.84925
C	-1.09777	-1.88761	3.06281
H	-1.28415	-0.84275	3.37748

H	-0.77531	-2.46363	3.96850
C	1.30862	-1.65896	2.58419
H	2.04857	-1.87106	1.78578
H	1.52878	-2.36210	3.42618
C	1.59905	0.86722	2.18277
C	1.74357	2.17184	2.69798
C	1.47202	-0.23403	3.07936
C	1.75124	2.40175	4.08388
H	1.83724	3.01823	2.00086
C	1.48992	0.01823	4.46362
C	1.62046	1.32246	4.97148
H	1.86307	3.42810	4.46738
H	1.39202	-0.83276	5.15744
H	1.62213	1.49165	6.05946
C	1.40670	2.21996	-0.37567
C	2.52271	2.71045	-1.08394
C	0.22891	2.99914	-0.30854
C	2.46438	3.96784	-1.70850
H	3.43498	2.09975	-1.15738
C	0.17806	4.25685	-0.92685
H	-0.65363	2.61302	0.22379
C	1.29476	4.74371	-1.63205
H	3.33931	4.34103	-2.26385
H	-0.74459	4.85519	-0.86772
H	1.25004	5.72658	-2.12687
C	2.93243	-0.23212	-0.15553
C	4.17412	0.03844	0.45943
C	2.85585	-1.13458	-1.23974
C	5.33995	-0.58050	-0.01981
H	4.22266	0.73224	1.31408
C	4.02967	-1.74075	-1.71704
H	1.86936	-1.39521	-1.66043
C	5.26878	-1.46488	-1.11258
H	6.30924	-0.37204	0.46035
H	3.97000	-2.44570	-2.56108
H	6.18501	-1.94878	-1.48675
C	-2.69721	1.16841	1.24074
C	-1.88389	1.56179	2.33287
C	-3.99592	1.72794	1.11339
C	-2.35365	2.49149	3.27061
C	-4.45835	2.65377	2.05744
C	-3.64195	3.03893	3.13898
H	-0.87411	1.13948	2.42334
H	-4.61974	1.42519	0.25937
H	-1.70269	2.78784	4.10775
H	-5.46709	3.08299	1.94975
H	-4.01224	3.76734	3.87721
C	-0.99381	-0.47065	-2.94738
C	-0.17960	-1.31938	-3.71551
C	-0.70040	0.90341	-2.87356
C	0.94769	-0.79901	-4.37264
C	0.42439	1.42214	-3.53249
C	1.25869	0.56873	-4.27505
H	-0.42913	-2.38854	-3.75871
H	-1.34263	1.56519	-2.27419
H	1.59496	-1.47087	-4.95867
H	0.65881	2.49391	-3.44545
H	2.15361	0.97028	-4.77593
C	-3.47496	-0.74438	-2.93182
O	-4.00497	-1.46770	-3.74307
O	-3.90021	0.51365	-2.63292
C	-5.01296	0.97987	-3.41202
H	-4.75171	1.00614	-4.48977
H	-5.89166	0.31632	-3.28084
H	-5.23692	1.99806	-3.04554
C	0.50495	-4.82977	-0.49089
H	1.06471	-4.85887	-1.44697
H	0.93265	-5.59663	0.18967
H	-0.54934	-5.09888	-0.70941

L2-AC-S(s-trans)

C	-2.24344	-0.80812	-2.24825
O	-1.75146	-1.91632	-1.95549
Cu	0.32590	-0.43981	0.41099
C	-1.27419	0.51387	0.19279
C	-2.33787	1.15785	0.03743
H	-0.24033	-2.12166	-1.39262
O	0.61843	-2.18600	-0.86167
C	0.68068	-3.38161	-0.08431
H	1.76647	-3.54449	0.10693
C	-0.01306	-3.19493	1.28332
H	0.22662	-4.09619	1.90792
N	0.47296	-1.97871	1.98539
P	2.36719	0.35320	0.69216
C	-1.54266	-2.99009	1.22074
H	-1.81879	-2.54076	0.24602
H	-2.07092	-3.96010	1.30702
C	-1.88048	-2.00842	2.37271
H	-2.59792	-2.43372	3.10208
H	-2.31054	-1.07630	1.95793
C	-0.52914	-1.71048	3.03992
H	-0.43045	-0.66851	3.40263
H	-0.34812	-2.39547	3.90872
C	1.84157	-2.10941	2.51967
H	2.51938	-2.35729	1.67675
H	1.88574	-2.96756	3.23807
C	2.62063	0.34335	2.51845
C	3.03237	1.49008	3.22576
C	2.32884	-0.85706	3.22690
C	3.17352	1.45929	4.62355
H	3.24485	2.41703	2.67106
C	2.48556	-0.86940	4.62499
C	2.90470	0.27468	5.32589
H	3.49749	2.36413	5.16141
H	2.26354	-1.79851	5.17528
H	3.01387	0.23921	6.42099
C	2.65434	2.09962	0.20766
C	3.89234	2.60575	-0.23856
C	1.52334	2.94860	0.25136
C	4.00153	3.95311	-0.62500
H	4.77035	1.94357	-0.29120
C	1.64372	4.29614	-0.12060
H	0.54645	2.52981	0.55030
C	2.88166	4.80049	-0.56131
H	4.96916	4.34253	-0.97944
H	0.75956	4.95174	-0.08326
H	2.97092	5.85523	-0.86590
C	3.85051	-0.56169	0.09769
C	5.10027	-0.50054	0.75244
C	3.69663	-1.38397	-1.04117
C	6.19129	-1.23298	0.25688
H	5.21225	0.11593	1.65866
C	4.79352	-2.11114	-1.53441
H	2.70017	-1.48161	-1.50324
C	6.04082	-2.03394	-0.89016
H	7.16463	-1.18159	0.77028
H	4.66886	-2.75261	-2.42122
H	6.89758	-2.60907	-1.27554
C	-3.55001	1.87593	-0.15752
C	-4.75356	1.46441	0.47852
C	-3.59822	3.00105	-1.02846
C	-5.95232	2.14945	0.25044
C	-4.80281	3.68207	-1.24816
C	-5.98490	3.26175	-0.61192
H	-4.72654	0.58624	1.14047
H	-2.67470	3.30652	-1.54104

H	-6.87634	1.80988	0.74495
H	-4.82182	4.54842	-1.92876
H	-6.93067	3.79808	-0.78839
C	0.13312	-4.58184	-0.85512
H	0.70965	-4.73115	-1.78959
H	0.20102	-5.50975	-0.25057
H	-0.92881	-4.41901	-1.12976
C	-3.71018	-0.60593	-2.21578
C	-4.37950	0.47471	-2.84253
C	-4.47275	-1.57272	-1.51279
C	-5.77682	0.56953	-2.77210
C	-5.86360	-1.46167	-1.43314
C	-6.52113	-0.38988	-2.06861
H	-3.79153	1.22784	-3.37910
H	-3.93928	-2.40148	-1.02528
H	-6.28560	1.41632	-3.25647
H	-6.44366	-2.21089	-0.87174
H	-7.61694	-0.29910	-2.00396
C	-1.31241	0.32462	-2.72267
O	-1.65640	1.40656	-3.17399
O	-0.02648	-0.06969	-2.62711
C	0.94672	0.96091	-2.84757
H	0.85776	1.37523	-3.87205
H	0.80466	1.77631	-2.11046
H	1.93278	0.48669	-2.69837

L2-TS-S(s-trans)

C	-2.45360	-1.47458	-3.24776
O	-2.02561	-2.65276	-3.07155
Cu	-0.37144	-0.89930	-0.84240
C	-2.07913	-0.25240	-1.46931
C	-2.58149	0.64418	-0.74651
H	-0.67896	-2.83213	-2.36371
O	0.13039	-2.77017	-1.72323
C	0.14316	-3.78592	-0.72173
H	1.18790	-3.79377	-0.33196
C	-0.79603	-3.43984	0.45880
H	-0.59787	-4.18234	1.27618
N	-0.55690	-2.06732	0.98377
P	1.42400	0.25826	-0.37155
C	-2.29504	-3.42859	0.10063
H	-2.41659	-3.13267	-0.96149
H	-2.73693	-4.43725	0.21866
C	-2.94105	-2.37165	1.02952
H	-3.65579	-2.81981	1.74765
H	-3.48583	-1.61672	0.43304
C	-1.76185	-1.72543	1.77706
H	-1.85198	-0.62644	1.87956
H	-1.65810	-2.15490	2.80549
C	0.69003	-1.92049	1.76237
H	1.54139	-2.22016	1.11678
H	0.67606	-2.62337	2.63380
C	1.25885	0.57850	1.43632
C	1.38144	1.87674	1.97175
C	0.89533	-0.50677	2.28582
C	1.15408	2.11584	3.33751
H	1.64818	2.71073	1.30456
C	0.68730	-0.24593	3.65290
C	0.80918	1.05026	4.18248
H	1.25047	3.13705	3.73830
H	0.40845	-1.08204	4.31479
H	0.62832	1.22482	5.25437
C	1.65082	1.91766	-1.11259
C	2.90678	2.50850	-1.36060
C	0.46744	2.59916	-1.48123
C	2.97692	3.77910	-1.95752
H	3.83021	1.97082	-1.09558
C	0.54494	3.87360	-2.06252
H	-0.50927	2.10968	-1.33333
C	1.79892	4.46510	-2.30244
H	3.95970	4.23530	-2.15602
H	-0.38102	4.39764	-2.34555
H	1.85861	5.46026	-2.77056
C	3.05093	-0.58515	-0.49647
C	4.14312	-0.27084	0.34115
C	3.17028	-1.62117	-1.45000
C	5.35502	-0.96738	0.20493
H	4.03630	0.51264	1.10846
C	4.38735	-2.31095	-1.58321
H	2.28989	-1.90597	-2.05101
C	5.47987	-1.98284	-0.76109
H	6.20617	-0.72031	0.85909
H	4.47795	-3.11924	-2.32593
H	6.43076	-2.52915	-0.86390
C	-3.09032	1.64638	0.12587
C	-4.41685	2.13848	-0.00926
C	-2.26738	2.17290	1.16144
C	-4.89534	3.12567	0.86289
C	-2.75970	3.15706	2.02732
C	-4.07381	3.63993	1.88384
H	-5.05632	1.72841	-0.80576
H	-1.24008	1.79579	1.27304
H	-5.92572	3.49809	0.74688
H	-2.10750	3.54918	2.82394
H	-4.45822	4.41365	2.56668
C	-0.16712	-5.15748	-1.32002
H	0.57742	-5.40868	-2.10110
H	-0.14265	-5.95011	-0.54376
H	-1.17121	-5.15864	-1.79071
C	-3.94359	-1.24487	-3.25665
C	-4.55100	0.01048	-3.49067
C	-4.76132	-2.36163	-2.98225
C	-5.94584	0.13653	-3.43806
C	-6.15714	-2.23023	-2.92364
C	-6.75532	-0.97900	-3.14868
H	-3.91701	0.88008	-3.70417
H	-4.27006	-3.33139	-2.81534
H	-6.40905	1.11910	-3.62269
H	-6.78210	-3.11023	-2.70265
H	-7.85077	-0.87130	-3.10207
C	-1.53152	-0.45707	-3.94666
O	-1.87118	0.53899	-4.56273
O	-0.23811	-0.84316	-3.81387
C	0.73277	0.10654	-4.26904
H	0.68883	0.22271	-5.37136
H	0.55941	1.09582	-3.79930
H	1.71668	-0.29189	-3.96135

L2-PC-S(s-trans)

C	-1.90247	-0.93043	-2.31762
O	-1.54586	-2.28402	-2.35576
Cu	-0.08019	-0.70343	0.08126
C	-1.74660	-0.31257	-0.96731
C	-1.81109	0.45813	0.03000
H	-0.69617	-2.41980	-1.75901
O	0.46179	-2.39651	-0.81165
C	0.71009	-3.38388	0.14806
H	1.77923	-3.30714	0.49385
C	-0.17233	-3.18784	1.40796
H	0.12774	-3.93666	2.18663
N	-0.00658	-1.81458	1.98175
P	1.80352	0.47573	0.45402
C	-1.69044	-3.27401	1.13737
H	-1.88630	-3.01428	0.07663
H	-2.06155	-4.30391	1.30390
C	-2.34957	-2.24003	2.08830

H	-3.08140	-2.69950	2.78149
H	-2.88708	-1.46270	1.51147
C	-1.17587	-1.62209	2.86439
H	-1.31174	-0.54976	3.10594
H	-1.00575	-2.16839	3.82811
C	1.27764	-1.61698	2.68067
H	2.08974	-1.92091	1.98905
H	1.33214	-2.30103	3.56491
C	1.83711	0.86240	2.24932
C	2.02145	2.17366	2.73165
C	1.50390	-0.18944	3.15306
C	1.87447	2.46054	4.09946
H	2.26562	2.97852	2.02092
C	1.37062	0.12024	4.51873
C	1.54625	1.43147	4.99572
H	2.01868	3.49032	4.46230
H	1.11360	-0.68831	5.22250
H	1.42540	1.64589	6.06889
C	2.00935	2.07971	-0.40914
C	3.25541	2.57125	-0.85257
C	0.82927	2.79329	-0.72051
C	3.31955	3.76795	-1.58624
H	4.17564	2.00762	-0.63509
C	0.89993	3.99439	-1.44174
H	-0.14700	2.38901	-0.41342
C	2.14482	4.48288	-1.87906
H	4.29510	4.14262	-1.93437
H	-0.02524	4.54270	-1.67838
H	2.19825	5.41837	-2.45778
C	3.37568	-0.42104	0.16200
C	4.55720	-0.14646	0.88458
C	3.36519	-1.42714	-0.83072
C	5.73344	-0.85737	0.59597
H	4.55008	0.61898	1.67728
C	4.54956	-2.12868	-1.11441
H	2.40475	-1.69096	-1.31510
C	5.73181	-1.84270	-0.40896
H	6.65581	-0.64426	1.15927
H	4.54270	-2.91809	-1.88262
H	6.65560	-2.39980	-0.63207
C	-2.34361	1.39873	0.97576
C	-3.69106	1.83010	0.84617
C	-1.55564	1.90971	2.03697
C	-4.22283	2.75206	1.75689
C	-2.09685	2.83283	2.94158
C	-3.43092	3.25662	2.80692
H	-4.30262	1.42828	0.02369
H	-0.51539	1.57435	2.13875
H	-5.26860	3.08059	1.64901
H	-1.46750	3.21881	3.75856
H	-3.85691	3.97917	3.52052
C	0.53020	-4.78818	-0.44300
H	1.21076	-4.91244	-1.30885
H	0.75701	-5.58394	0.29827
H	-0.50776	-4.92903	-0.80903
C	-3.36442	-0.73795	-2.73313
C	-3.91163	0.55640	-2.83497
C	-4.17831	-1.85910	-2.95772
C	-5.26567	0.72617	-3.15846
C	-5.53554	-1.68767	-3.28344
C	-6.08318	-0.39798	-3.38187
H	-3.27136	1.43557	-2.66089
H	-3.72226	-2.85548	-2.87354
H	-5.68618	1.74097	-3.24212
H	-6.17003	-2.57069	-3.46115
H	-7.14675	-0.26526	-3.63615
C	-0.95163	-0.14504	-3.29406
O	-1.23860	0.24813	-4.40553
O	0.27994	-0.02715	-2.73951
C	1.29187	0.59371	-3.54510
H	1.33838	0.12246	-4.54704
H	1.08146	1.67656	-3.66123
H	2.24089	0.45281	-2.99734

L2-AC-S(s-cis)

C	-1.94710	-1.05421	-2.13640
O	-1.95048	-2.43639	-1.95729
Cu	-0.22196	-0.77789	0.25799
C	-1.87289	-0.33794	-0.82972
C	-1.99806	0.35079	0.21547
H	-1.03987	-2.63700	-1.49622
O	0.25473	-2.45949	-0.69740
C	0.68395	-3.40573	0.23755
H	1.77474	-3.24516	0.46756
C	-0.07731	-3.24570	1.57855
H	0.34179	-3.96105	2.33223
N	0.07282	-1.85472	2.12060
P	1.62032	0.51986	0.42654
C	-1.60965	-3.41872	1.46643
H	-1.93050	-3.22485	0.42299
H	-1.90528	-4.45410	1.72515
C	-2.22647	-2.36655	2.42705
H	-2.91522	-2.81023	3.17266
H	-2.79956	-1.60734	1.85919
C	-1.01668	-1.71710	3.11089
H	-1.16895	-0.65519	3.38463
H	-0.73963	-2.27343	4.04325
C	1.40875	-1.59852	2.69643
H	2.16365	-1.86723	1.92960
H	1.57472	-2.27933	3.56831
C	1.79942	0.90116	2.21858
C	1.95964	2.22061	2.68865
C	1.60922	-0.16300	3.14831
C	1.92831	2.50125	4.06498
H	2.09527	3.03757	1.96343
C	1.59022	0.13995	4.52207
C	1.74149	1.45859	4.98581
H	2.05286	3.53797	4.41516
H	1.44401	-0.68093	5.24311
H	1.71285	1.66797	6.06638
C	1.70401	2.15238	-0.40172
C	2.87483	2.65629	-1.00604
C	0.50056	2.88855	-0.49508
C	2.84417	3.88671	-1.68429
H	3.80982	2.07777	-0.95492
C	0.47851	4.12309	-1.16037
H	-0.42383	2.48043	-0.06120
C	1.64923	4.62405	-1.75981
H	3.76113	4.27096	-2.15839
H	-0.46398	4.68876	-1.22582
H	1.62783	5.58720	-2.29350
C	3.18858	-0.31684	-0.02061
C	4.42195	-0.01139	0.59523
C	3.12085	-1.31063	-1.02371
C	5.58897	-0.67991	0.19146
H	4.46265	0.74644	1.39420
C	4.29635	-1.96816	-1.42521
H	2.13500	-1.60437	-1.43164
C	5.52754	-1.65251	-0.82410
H	6.55147	-0.44292	0.67205
H	4.24353	-2.74541	-2.20367
H	6.44459	-2.17578	-1.13830
C	-2.50041	1.26570	1.20079
C	-3.83032	1.75352	1.09686
C	-1.68621	1.71396	2.27184
C	-4.32082	2.66415	2.04184

C	-2.18521	2.62734	3.21001
C	-3.50363	3.10399	3.10133
H	-4.46137	1.40613	0.26477
H	-0.65488	1.34411	2.34717
H	-5.35373	3.03607	1.95240
H	-1.53406	2.96570	4.03100
H	-3.89688	3.81875	3.84103
C	0.54874	-4.83038	-0.31473
H	1.12404	-4.91236	-1.25820
H	0.93073	-5.59117	0.39900
H	-0.51123	-5.06335	-0.54658
C	-3.20102	-0.60035	-2.90088
C	-3.54223	0.76444	-2.98043
C	-3.99641	-1.56336	-3.54435
C	-4.68196	1.16157	-3.69696
C	-5.13694	-1.16100	-4.26039
C	-5.48335	0.19891	-4.33808
H	-2.90580	1.51058	-2.47988
H	-3.70231	-2.61904	-3.46244
H	-4.94727	2.22925	-3.75540
H	-5.76119	-1.91787	-4.76170
H	-6.37863	0.51079	-4.89903
C	-0.70289	-0.64484	-3.00972
O	-0.15443	-1.38366	-3.79504
O	-0.34333	0.64444	-2.78690
C	0.79786	1.10423	-3.53096
H	1.70686	0.55490	-3.21199
H	0.64581	0.94538	-4.61738
H	0.90259	2.17776	-3.29546

L2-TS-S(s-cis)

C	-2.22785	-1.21349	-2.64762
O	-2.17632	-2.48865	-2.46567
Cu	-0.33746	-0.99929	-0.39759
C	-2.04896	-0.29675	-0.96441
C	-2.89359	0.50445	-0.48863
H	-0.93423	-2.86691	-1.85179
O	-0.04927	-2.89655	-1.25342
C	-0.06349	-3.91363	-0.26332
H	1.00480	-4.11039	-0.00714
C	-0.76696	-3.42756	1.02643
H	-0.54957	-4.16787	1.84113
N	-0.27797	-2.08562	1.44963
P	1.47338	0.21155	-0.22548
C	-2.28522	-3.20736	0.89749
H	-2.51720	-2.86756	-0.13518
H	-2.84241	-4.14764	1.07560
C	-2.62545	-2.09796	1.92436
H	-3.24642	-2.47399	2.76132
H	-3.17962	-1.27569	1.43409
C	-1.26097	-1.60296	2.44540
H	-1.19127	-0.50136	2.54168
H	-1.02753	-2.04636	3.44634
C	1.11636	-2.07135	1.93731
H	1.77234	-2.40917	1.10780
H	1.23153	-2.81127	2.76910
C	1.77952	0.40637	1.58352
C	2.16210	1.64758	2.13180
C	1.56567	-0.70946	2.44406
C	2.35223	1.79808	3.51547
H	2.31139	2.50758	1.46081
C	1.76965	-0.53752	3.82655
C	2.15982	0.69961	4.36648
H	2.65296	2.77523	3.92486
H	1.60856	-1.39862	4.49561
H	2.30613	0.80328	5.45286
C	1.44461	1.92812	-0.86646
C	2.57716	2.58836	-1.38565
C	0.18747	2.57269	-0.87636
C	2.45279	3.89092	-1.89923
H	3.55244	2.07673	-1.39809
C	0.07248	3.87793	-1.37700
H	-0.70145	2.02092	-0.53085
C	1.20422	4.53881	-1.89051
H	3.33714	4.40293	-2.31046
H	-0.91212	4.37149	-1.38674
H	1.11107	5.55880	-2.29571
C	3.02644	-0.52586	-0.86248
C	4.27225	-0.37120	-0.21698
C	2.93003	-1.30172	-2.04054
C	5.42051	-0.97325	-0.75761
H	4.33827	0.21587	0.71292
C	4.08591	-1.89078	-2.57883
H	1.95112	-1.45471	-2.52543
C	5.32875	-1.72885	-1.94067
H	6.39190	-0.85346	-0.25202
H	4.00881	-2.49295	-3.49760
H	6.23026	-2.20163	-2.36163
C	-3.92387	1.37825	-0.05490
C	-5.27791	1.06768	-0.36626
C	-3.64045	2.58161	0.64797
C	-6.30436	1.94568	0.00265
C	-4.67896	3.44132	1.02797
C	-6.01311	3.13095	0.70354
H	-5.49778	0.14240	-0.91922
H	-2.59501	2.82255	0.89456
H	-7.34593	1.70052	-0.25817
H	-4.44678	4.36674	1.57860
H	-6.82582	3.81337	0.99794
C	-0.67543	-5.20945	-0.79727
H	-0.09329	-5.56991	-1.66817
H	-0.67763	-6.00401	-0.02219
H	-1.71891	-5.03987	-1.13165
C	-3.58104	-0.67574	-3.11979
C	-3.82910	0.66791	-3.47488
C	-4.64294	-1.60132	-3.17095
C	-5.11380	1.07039	-3.87008
C	-5.92998	-1.19455	-3.55991
C	-6.17132	0.14407	-3.91321
H	-3.01432	1.40052	-3.41453
H	-4.42431	-2.64309	-2.89553
H	-5.29353	2.12433	-4.13525
H	-6.74920	-1.93096	-3.59138
H	-7.17955	0.46642	-4.21832
C	-0.94799	-0.62930	-3.32853
O	-0.06201	-1.34381	-3.76185
O	-0.90470	0.72319	-3.40542
C	0.27482	1.26183	-4.02551
H	1.18498	0.90375	-3.50593
H	0.32985	0.95880	-5.09099
H	0.19382	2.35896	-3.92954

L2-PC-S(s-cis)

C	-1.99700	-0.95455	-2.12923
O	-1.72607	-2.11859	-1.78195
Cu	0.22212	-0.69257	0.61714
C	-1.38739	0.23545	0.39214
C	-2.40009	0.95266	0.22136
H	-0.21355	-2.43861	-1.15380
O	0.61055	-2.46940	-0.57401
C	0.65897	-3.64592	0.22922
H	1.74281	-3.85744	0.37882
C	0.03856	-3.38412	1.61870
H	0.30993	-4.24825	2.28231
N	0.55756	-2.12843	2.22149
P	2.18640	0.30560	0.73337

C	-1.48909	-3.16852	1.63169
H	-1.80617	-2.71559	0.66982
H	-2.02370	-4.13155	1.75025
C	-1.75335	-2.17626	2.79565
H	-2.39695	-2.60812	3.58751
H	-2.24051	-1.26106	2.40683
C	-0.35639	-1.83841	3.34620
H	-0.24448	-0.78366	3.66536
H	-0.09642	-2.49144	4.21937
C	1.97765	-2.19075	2.61342
H	2.57327	-2.41985	1.70543
H	2.13550	-3.03516	3.33197
C	2.65653	0.29585	2.51494
C	3.11130	1.45738	3.17125
C	2.48542	-0.90996	3.25443
C	3.40871	1.43988	4.54415
H	3.23213	2.38695	2.59365
C	2.79610	-0.90808	4.62711
C	3.25395	0.25201	5.27499
H	3.76460	2.35667	5.04000
H	2.66670	-1.84053	5.20087
H	3.48407	0.22591	6.35150
C	2.18724	2.07920	0.26948
C	3.23278	2.69593	-0.44883
C	1.01649	2.81406	0.57472
C	3.10939	4.03632	-0.85558
H	4.13752	2.11989	-0.69861
C	0.90949	4.15670	0.18390
H	0.17516	2.30721	1.07586
C	1.95252	4.76947	-0.53677
H	3.92465	4.51137	-1.42421
H	-0.00534	4.72118	0.42444
H	1.86015	5.81939	-0.85706
C	3.65020	-0.40702	-0.10978
C	4.92853	-0.48087	0.48410
C	3.43969	-0.94088	-1.40255
C	5.99343	-1.07507	-0.21382
H	5.08581	-0.07346	1.49541
C	4.51335	-1.52029	-2.09738
H	2.43293	-0.90876	-1.84929
C	5.78804	-1.59143	-1.50583
H	6.98976	-1.13313	0.25285
H	4.34827	-1.93309	-3.10519
H	6.62419	-2.05733	-2.05119
C	-3.51855	1.78128	-0.06212
C	-4.83652	1.40679	0.31817
C	-3.34960	2.99670	-0.78614
C	-5.93368	2.20634	-0.02263
C	-4.45239	3.79266	-1.11997
C	-5.75071	3.40084	-0.74435
H	-4.97765	0.46563	0.86956
H	-2.33160	3.28820	-1.08357
H	-6.94805	1.89266	0.27133
H	-4.30088	4.72785	-1.68295
H	-6.61768	4.02565	-1.01096
C	0.02526	-4.84282	-0.47746
H	0.55266	-5.04561	-1.43066
H	0.08024	-5.75404	0.15335
H	-1.04021	-4.64218	-0.71062
C	-3.40700	-0.53505	-2.32279
C	-3.81221	0.63995	-3.00148
C	-4.40274	-1.40812	-1.81640
C	-5.17515	0.92077	-3.17502
C	-5.75946	-1.11712	-1.98268
C	-6.15097	0.04931	-2.66828
H	-3.05635	1.33108	-3.39008
H	-4.07387	-2.31401	-1.28713
H	-5.47540	1.84252	-3.69546
H	-6.52137	-1.79916	-1.57365
H	-7.21944	0.28376	-2.79534
C	-0.77564	-0.07144	-2.48762
O	0.32570	-0.56757	-2.65185
O	-1.02872	1.24211	-2.63701
C	0.13646	2.06237	-2.84702
H	0.82808	1.95881	-1.98878
H	0.66202	1.76632	-3.77680
H	-0.23300	3.10039	-2.91608

L7-R

Cu	0.57913	0.48614	1.15392
C	-0.90315	0.85404	0.04581
C	-1.80029	0.79211	-0.82414
H	-0.11797	-0.90241	-0.51958
O	0.62493	-1.55110	-0.35857
C	0.14062	-2.66208	0.38798
H	1.03619	-3.30556	0.53004
C	-0.36347	-2.28044	1.80720
H	-0.30546	-3.22545	2.41256
N	0.48470	-1.26033	2.47816
P	2.59428	1.12719	1.72973
C	-1.79360	-1.70678	1.93971
H	-2.08613	-1.16087	1.02200
H	-2.52188	-2.52493	2.10330
C	-1.71851	-0.71912	3.13496
H	-2.47397	-0.92551	3.91842
H	-1.87250	0.31661	2.77165
C	-0.28964	-0.87644	3.67606
H	0.13000	0.04456	4.12774
H	-0.23378	-1.68878	4.44575
C	1.84469	-1.75352	2.76308
H	2.36275	-1.86775	1.78765
H	1.78078	-2.77518	3.21665
C	3.09047	0.43741	3.36609
C	3.86242	1.16884	4.29289
C	2.65993	-0.87858	3.70328
C	4.20941	0.62696	5.54128
H	4.20115	2.18420	4.03636
C	3.02064	-1.40592	4.95919
C	3.78581	-0.66759	5.87668
H	4.81256	1.21768	6.24843
H	2.68673	-2.42312	5.22166
H	4.04789	-1.10519	6.85244
C	-2.82702	0.67835	-1.80892
C	-4.19015	0.92739	-1.48640
C	-2.51706	0.28609	-3.14186
C	-5.19398	0.78406	-2.45384
C	-3.52809	0.14013	-4.10117
C	-4.87205	0.38708	-3.76493
H	-4.44235	1.23325	-0.45959
H	-1.46509	0.09963	-3.40573
H	-6.24324	0.98222	-2.18143
H	-3.26419	-0.16806	-5.12568
H	-5.66427	0.27266	-4.52132
C	-0.92775	-3.47686	-0.35574
H	-1.24892	-4.29569	0.32842
H	-1.82384	-2.83546	-0.50704
C	2.74252	2.97577	1.90040
C	1.66399	3.44333	2.90286
C	1.58557	4.97470	2.99293
C	1.34835	5.60330	1.61169
C	2.42651	5.15668	0.61342
C	2.50912	3.62490	0.51878
H	3.75436	3.25734	2.27089
H	1.84666	3.00316	3.90575
H	0.68599	3.03635	2.55355
H	0.78031	5.26751	3.70027

H	2.53696	5.36818	3.42026
H	1.32146	6.71153	1.68653
H	0.34994	5.28564	1.23464
H	2.22673	5.58164	-0.39336
H	3.41502	5.55979	0.93428
H	3.31383	3.33190	-0.18728
H	1.55960	3.21536	0.10385
C	3.54399	0.39507	-0.83471
C	4.65697	-0.22789	-1.69239
C	5.98580	0.53036	-1.54057
C	6.41765	0.61350	-0.06726
C	5.32011	1.24866	0.80516
C	3.98644	0.50262	0.63835
H	2.61160	-0.20426	-0.89324
H	3.29566	1.40620	-1.22562
H	4.34233	-0.25808	-2.75734
H	4.80346	-1.28792	-1.38057
H	6.78134	0.04932	-2.14922
H	5.86511	1.56267	-1.94322
H	7.36355	1.18830	0.03276
H	6.63243	-0.41246	0.31072
H	5.62668	1.25599	1.87333
H	5.18787	2.31140	0.50227
H	4.13106	-0.53718	1.01602
C	-0.53901	-4.10637	-1.72351
C	-1.65085	-5.10959	-2.09928
H	-1.45691	-5.56338	-3.09415
H	-1.72209	-5.93372	-1.35731
H	-2.64070	-4.60791	-2.14497
C	0.80675	-4.85207	-1.62466
H	0.79771	-5.60310	-0.80498
H	1.02426	-5.39156	-2.57043
H	1.64774	-4.15146	-1.44311
C	-0.45397	-3.02819	-2.82580
H	-0.24619	-3.49596	-3.81191
H	-1.41096	-2.47130	-2.91002
H	0.34538	-2.29567	-2.60529

L7-AC-R(s-trans)

C	-1.81209	-0.83676	-2.29056
O	-1.54506	-2.00450	-1.94487
Cu	0.07182	-0.26961	0.52905
C	-1.49620	0.63137	0.00947
C	-2.48797	1.39368	-0.08371
H	-0.24138	-2.21946	-1.06575
O	0.52032	-2.18782	-0.40189
C	0.39950	-3.27887	0.51658
H	1.43036	-3.45666	0.89628
C	-0.47754	-2.87719	1.72107
H	-0.36608	-3.68324	2.49546
N	-0.03797	-1.58595	2.30309
P	1.92997	0.78475	1.10856
C	-1.97262	-2.64470	1.41982
H	-2.11095	-2.29413	0.37774
H	-2.54515	-3.58624	1.53309
C	-2.41909	-1.53924	2.41375
H	-3.21131	-1.88329	3.10789
H	-2.79863	-0.66541	1.85036
C	-1.14247	-1.15792	3.18557
H	-1.05828	-0.07368	3.39949
H	-1.08017	-1.70679	4.16082
C	1.26263	-1.67878	2.98378
H	2.01345	-1.99518	2.22990
H	1.22331	-2.49070	3.75524
C	2.12632	0.75910	2.94497
C	2.61876	1.87288	3.65822
C	1.73532	-0.40535	3.66615
C	2.71368	1.86028	5.05927
H	2.93311	2.77262	3.10796
C	1.83502	-0.39972	5.07164
C	2.31354	0.71974	5.77136
H	3.09994	2.74364	5.59167
H	1.52845	-1.30110	5.62739
H	2.37522	0.69742	6.87051
C	-3.64168	2.22113	-0.15349
C	-4.68192	2.09685	0.81296
C	-3.81287	3.17499	-1.19539
C	-5.83824	2.88205	0.72992
C	-4.97456	3.95370	-1.27438
C	-5.99464	3.81219	-0.31626
H	-4.55804	1.36497	1.62587
H	-3.01415	3.29134	-1.94186
H	-6.62957	2.76906	1.48841
H	-5.08666	4.68053	-2.09457
H	-6.90668	4.42590	-0.38142
C	-0.79943	0.04211	-2.93961
C	0.48097	-0.50419	-3.19158
C	-1.06285	1.37775	-3.32690
C	1.47574	0.26415	-3.80882
C	-0.06756	2.13938	-3.95113
C	1.20524	1.58979	-4.19143
H	0.68657	-1.54239	-2.89919
H	-2.05858	1.79779	-3.14567
H	2.46596	-0.17689	-4.00077
H	-0.28685	3.17545	-4.25201
H	1.98622	2.19552	-4.67757
C	-3.28724	-0.40835	-2.24432
O	-3.79874	0.42564	-2.97603
O	-3.97231	-1.16266	-1.36368
C	-5.37251	-0.85558	-1.26662
H	-5.51895	0.20610	-0.98516
H	-5.88190	-1.04279	-2.23416
H	-5.77273	-1.52308	-0.48259
C	-0.11093	-4.55644	-0.15691
H	-0.39015	-5.27333	0.64767
H	-1.05521	-4.31110	-0.69097
C	1.82309	2.59526	0.66067
C	0.56134	3.18832	1.32879
C	0.24232	4.60521	0.82909
C	0.12287	4.64102	-0.70113
C	1.41003	4.11689	-1.35029
C	1.72317	2.69099	-0.87761
H	2.73177	3.14164	1.00364
H	0.66922	3.18439	2.43316
H	-0.29442	2.51901	1.09076
H	-0.69900	4.95796	1.30196
H	1.04336	5.31127	1.15145
H	-0.10596	5.66984	-1.05350
H	-0.72895	3.99433	-1.01109
H	1.32650	4.12684	-2.45738
H	2.25821	4.79267	-1.09098
H	2.65902	2.32863	-1.35273
H	0.91277	2.00555	-1.21219
C	3.49866	-0.48106	-0.86159
C	4.82815	-1.12373	-1.28410
C	5.99362	-0.12565	-1.18556
C	6.09541	0.47888	0.22475
C	4.77013	1.13359	0.65489
C	3.60320	0.14001	0.54513
H	2.66639	-1.21526	-0.88128
H	3.22209	0.31037	-1.59155
H	4.74568	-1.52861	-2.31612
H	5.03727	-1.99714	-0.62356
H	6.95145	-0.61357	-1.46740
H	5.83159	0.69540	-1.92180
H	6.92159	1.22080	0.27285

H	6.35449	-0.32607	0.95047
H	4.84757	1.52484	1.69196
H	4.56816	2.00832	-0.00324
H	3.79399	-0.69151	1.26481
C	0.85165	-5.28670	-1.13436
C	0.11279	-6.54015	-1.64958
H	0.74771	-7.11452	-2.35661
H	-0.16389	-7.21811	-0.81420
H	-0.82071	-6.26087	-2.18211
C	2.13690	-5.72411	-0.40220
H	1.90475	-6.35528	0.48231
H	2.79064	-6.31594	-1.07660
H	2.73072	-4.85295	-0.05424
C	1.22533	-4.39716	-2.33996
H	1.82045	-4.97654	-3.07738
H	0.31700	-4.02047	-2.85477
H	1.82387	-3.51960	-2.02609

L7-TS-R(s-trans)

C	-1.92601	-1.44518	-2.97065
O	-1.67028	-2.69474	-2.79482
Cu	-0.28036	-1.05752	-0.37249
C	-1.85028	-0.40952	-1.30974
C	-2.65400	0.50865	-0.99354
H	-0.56046	-2.91372	-1.95893
O	0.20009	-2.93206	-1.21288
C	-0.02100	-4.00630	-0.30274
H	0.96983	-4.22704	0.15746
C	-0.97160	-3.57358	0.83864
H	-0.90618	-4.35206	1.64470
N	-0.57508	-2.25959	1.40947
P	1.38290	0.21311	0.31365
C	-2.44442	-3.35073	0.44026
H	-2.51105	-3.02248	-0.61773
H	-3.02047	-4.29237	0.52851
C	-2.95982	-2.23795	1.39134
H	-3.77543	-2.58731	2.05483
H	-3.34337	-1.38422	0.80088
C	-1.72897	-1.81848	2.21849
H	-1.66154	-0.72808	2.40929
H	-1.71452	-2.33646	3.21147
C	0.69733	-2.30675	2.15007
H	1.49082	-2.60014	1.43089
H	0.64915	-3.11726	2.92109
C	1.46725	0.17071	2.15835
C	1.87062	1.30088	2.90141
C	1.08775	-1.01495	2.85236
C	1.89043	1.28513	4.30531
H	2.17382	2.21668	2.37178
C	1.11306	-1.01106	4.26160
C	1.50421	0.12359	4.99029
H	2.20817	2.18160	4.86047
H	0.81699	-1.92766	4.79766
H	1.50874	0.09651	6.09102
C	-3.51275	1.58898	-0.66439
C	-3.68370	2.00292	0.68608
C	-4.19583	2.30061	-1.69105
C	-4.48829	3.10780	0.99198
C	-5.00795	3.39513	-1.36754
C	-5.15223	3.81092	-0.03055
H	-3.15987	1.45200	1.48155
H	-4.09274	1.95930	-2.73189
H	-4.60076	3.42404	2.04117
H	-5.53277	3.93493	-2.17163
H	-5.78409	4.67867	0.21465
C	-0.92021	-0.60726	-3.75238
C	0.30729	-1.21447	-4.08531
C	-1.14074	0.73445	-4.13481
C	1.30082	-0.49755	-4.77087
C	-0.16067	1.43841	-4.84911
C	1.07020	0.83230	-5.16037
H	0.47302	-2.26096	-3.79608
H	-2.08774	1.22194	-3.87558
H	2.25712	-0.98796	-5.01301
H	-0.35859	2.47665	-5.15870
H	1.84338	1.39442	-5.70774
C	-3.42389	-1.12975	-3.18868
O	-3.88758	-0.25287	-3.90118
O	-4.18683	-1.98542	-2.47367
C	-5.59261	-1.70975	-2.50258
H	-5.80138	-0.71354	-2.05903
H	-5.98038	-1.71987	-3.54143
H	-6.07115	-2.50446	-1.90203
C	-0.53531	-5.27108	-1.00089
H	-0.86768	-5.98206	-0.21073
H	-1.44436	-4.99672	-1.57877
C	1.19886	2.01371	-0.12997
C	-0.14597	2.50211	0.45342
C	-0.56234	3.88510	-0.06743
C	-0.56172	3.92752	-1.60191
C	0.81260	3.52415	-2.15129
C	1.20690	2.12172	-1.67030
H	2.04104	2.61306	0.28530
H	-0.11180	2.49980	1.56270
H	-0.91803	1.76009	0.16155
H	-1.56995	4.13373	0.32844
H	0.13737	4.66047	0.32309
H	-0.85155	4.93721	-1.96368
H	-1.32998	3.21869	-1.98446
H	0.81583	3.54237	-3.26079
H	1.57773	4.26387	-1.81868
H	2.20245	1.84453	-2.07498
H	0.48642	1.37931	-2.07914
C	3.07773	-0.99300	-1.58223
C	4.45796	-1.54995	-1.96139
C	5.55712	-0.48357	-1.82788
C	5.58068	0.11902	-0.41352
C	4.20796	0.69831	-0.02672
C	3.10167	-0.35827	-0.17701
H	2.30203	-1.78661	-1.61584
H	2.76526	-0.23261	-2.33039
H	4.42981	-1.95671	-2.99511
H	4.70037	-2.40997	-1.29471
H	6.55054	-0.91172	-2.08204
H	5.36720	0.32918	-2.56647
H	6.36131	0.90627	-0.33570
H	5.86017	-0.67345	0.31835
H	4.22860	1.09053	1.01284
H	3.98180	1.56205	-0.69103
H	3.30463	-1.17374	0.55736
C	0.44856	-6.02394	-1.93887
C	-0.29355	-7.26954	-2.46871
H	0.35389	-7.85809	-3.15254
H	-0.60690	-7.93808	-1.63840
H	-1.20512	-6.97830	-3.03181
C	1.70197	-6.47651	-1.16242
H	1.43152	-7.09606	-0.28022
H	2.36673	-7.08539	-1.81061
H	2.29870	-5.61186	-0.80336
C	0.87274	-5.14943	-3.13894
H	1.47451	-5.74596	-3.85737
H	-0.01343	-4.75120	-3.67487
H	1.47958	-4.28306	-2.81121

L7-PC-R(s-trans)

C	-2.07515	-0.65477	-2.05471

O	-1.81937	-2.02344	-1.97351
Cu	-0.35049	-0.32146	0.40175
C	-1.99123	0.02661	-0.73190
C	-2.10951	0.80032	0.25417
H	-0.97749	-2.13385	-1.35620
O	0.18455	-2.04727	-0.44013
C	0.35298	-3.04767	0.52362
H	1.39892	-2.99721	0.93691
C	-0.59988	-2.83705	1.72733
H	-0.34087	-3.58021	2.52629
N	-0.46038	-1.46408	2.30362
P	1.46368	0.92347	0.90974
C	-2.10435	-2.92919	1.38759
H	-2.26287	-2.68862	0.31677
H	-2.47811	-3.95782	1.55627
C	-2.80311	-1.88266	2.29562
H	-3.58537	-2.32553	2.94300
H	-3.28576	-1.09475	1.68553
C	-1.66675	-1.28633	3.13833
H	-1.81261	-0.22142	3.40120
H	-1.54010	-1.85829	4.09408
C	0.78859	-1.29028	3.06201
H	1.62186	-1.60390	2.40205
H	0.79621	-1.99264	3.93412
C	1.43614	1.19594	2.73519
C	1.69006	2.45894	3.31110
C	1.03011	0.11869	3.58030
C	1.51471	2.68335	4.68676
H	2.02616	3.28988	2.67382
C	0.86609	0.36292	4.95715
C	1.09063	1.63315	5.51401
H	1.71311	3.68111	5.10867
H	0.55071	-0.47027	5.60637
H	0.94222	1.79634	6.59270
C	-2.69462	1.74484	1.16351
C	-2.04644	2.13770	2.35978
C	-3.96170	2.30029	0.84336
C	-2.64909	3.06733	3.21768
C	-4.55377	3.23029	1.70814
C	-3.90343	3.61679	2.89622
H	-1.06101	1.71375	2.59641
H	-4.45070	1.98394	-0.09082
H	-2.13084	3.36429	4.14281
H	-5.53549	3.66010	1.45333
H	-4.37616	4.34740	3.57127
C	-1.07843	0.10317	-2.98342
C	0.12625	-0.52552	-3.34042
C	-1.34369	1.41357	-3.42755
C	1.05447	0.14675	-4.15330
C	-0.42457	2.07304	-4.26009
C	0.77781	1.44114	-4.62613
H	0.32587	-1.53942	-2.97029
H	-2.27645	1.91303	-3.12161
H	1.99893	-0.35122	-4.42447
H	-0.64646	3.08898	-4.62298
H	1.49900	1.95983	-5.27722
C	-3.50509	-0.42569	-2.62138
O	-4.25227	0.47923	-2.28606
O	-3.79244	-1.32758	-3.57334
C	-5.07353	-1.16784	-4.20361
H	-5.89112	-1.26381	-3.46045
H	-5.15283	-0.17161	-4.68497
H	-5.14144	-1.97091	-4.95911
C	0.15442	-4.45927	-0.06026
H	0.09102	-5.18361	0.78442
H	-0.83586	-4.48059	-0.56677
C	1.61497	2.61967	0.14338
C	0.32228	3.42810	0.37389
C	0.37567	4.80013	-0.31217
C	0.63383	4.65102	-1.81775
C	1.90824	3.83694	-2.07787
C	1.87101	2.47280	-1.37202
H	2.47851	3.15339	0.60393
H	0.10930	3.54687	1.45453
H	-0.52590	2.84497	-0.04404
H	-0.57549	5.34368	-0.12797
H	1.18476	5.41482	0.14669
H	0.69878	5.64615	-2.30821
H	-0.22929	4.12163	-2.27914
H	2.05428	3.67942	-3.16731
H	2.79337	4.41149	-1.71853
H	2.82055	1.93353	-1.56072
H	1.06394	1.84564	-1.81037
C	3.14926	-0.74068	-0.65150
C	4.44921	-1.55261	-0.74593
C	5.68912	-0.65444	-0.60452
C	5.64804	0.15485	0.70285
C	4.35213	0.97819	0.81884
C	3.11900	0.07407	0.65740
H	2.24415	-1.38392	-0.70815
H	3.08440	-0.05355	-1.52307
H	4.47667	-2.11747	-1.70212
H	4.46029	-2.31783	0.06450
H	6.61963	-1.26004	-0.65311
H	5.72841	0.04985	-1.46756
H	6.53256	0.82368	0.77781
H	5.71017	-0.54494	1.56787
H	4.31482	1.51359	1.79220
H	4.34705	1.75695	0.02406
H	3.13779	-0.66067	1.49584
C	1.22796	-4.98688	-1.05091
C	0.80763	-6.41180	-1.47082
H	1.53973	-6.85077	-2.18166
H	0.73891	-7.08907	-0.59258
H	-0.18469	-6.40226	-1.96950
C	2.60768	-5.05328	-0.36558
H	2.57469	-5.68041	0.55131
H	3.36695	-5.49056	-1.04793
H	2.96756	-4.04500	-0.07575
C	1.32251	-4.10434	-2.31320
H	2.02269	-4.55524	-3.04911
H	0.33101	-3.99666	-2.80012
H	1.67708	-3.08701	-2.06105

L7-AC-R(s-cis)

C	-1.96341	-0.85307	-2.14539
O	-1.65221	-1.99471	-1.75975
Cu	0.05000	-0.23628	0.67486
C	-1.53718	0.63310	0.16947
C	-2.56465	1.32791	-0.01773
H	-0.31764	-2.17814	-0.92285
O	0.46832	-2.15592	-0.28830
C	0.37164	-3.24766	0.63167
H	1.41315	-3.42745	0.98030
C	-0.46680	-2.84853	1.86373
H	-0.33234	-3.65831	2.63049
N	-0.00209	-1.56235	2.44053
P	1.96586	0.76079	1.16415
C	-1.97131	-2.61050	1.61659
H	-2.15093	-2.26075	0.58044
H	-2.54218	-3.55006	1.75120
C	-2.37756	-1.50521	2.62824
H	-3.15395	-1.84423	3.34218
H	-2.76449	-0.62368	2.08248
C	-1.07567	-1.14234	3.36478
H	-0.97819	-0.06274	3.59518

H	-0.98395	-1.70863	4.32788
C	1.31816	-1.66858	3.08019
H	2.04044	-1.99849	2.30475
H	1.29324	-2.47602	3.85689
C	2.21290	0.75546	2.99495
C	2.73277	1.87162	3.68442
C	1.82629	-0.39658	3.73856
C	2.86054	1.87289	5.08302
H	3.04390	2.76223	3.11789
C	1.96025	-0.37762	5.14082
C	2.46685	0.74410	5.81692
H	3.26795	2.75805	5.59638
H	1.65719	-1.26978	5.71314
H	2.55472	0.73284	6.91446
C	-3.74990	2.08356	-0.23041
C	-5.01178	1.59521	0.21491
C	-3.71957	3.33015	-0.91880
C	-6.18509	2.32220	-0.02265
C	-4.89928	4.04708	-1.15918
C	-6.13898	3.54805	-0.71392
H	-5.04497	0.62524	0.73144
H	-2.74757	3.72293	-1.25431
H	-7.15113	1.92559	0.32864
H	-4.85335	5.00868	-1.69515
H	-7.06417	4.11551	-0.90068
C	-0.96852	0.04784	-2.79606
C	0.28112	-0.51559	-3.14639
C	-1.20490	1.41444	-3.08058
C	1.26562	0.25865	-3.77405
C	-0.22122	2.18265	-3.71384
C	1.01581	1.61053	-4.06546
H	0.46826	-1.57474	-2.92408
H	-2.15876	1.86942	-2.79240
H	2.22989	-0.19851	-4.04468
H	-0.41826	3.24444	-3.92795
H	1.78672	2.22160	-4.56088
C	-3.47465	-0.55096	-2.07728
O	-4.22972	-1.16350	-1.34924
O	-3.84685	0.37394	-2.99478
C	-5.24570	0.70715	-3.08217
H	-5.54811	0.59072	-4.14145
H	-5.83607	0.04030	-2.42642
H	-5.37354	1.76055	-2.76510
C	-0.15833	-4.52566	-0.02582
H	-0.40295	-5.24483	0.78790
H	-1.12343	-4.28518	-0.52341
C	1.92805	2.56776	0.69383
C	0.67690	3.20920	1.33575
C	0.42912	4.63896	0.83239
C	0.34314	4.68212	-0.70012
C	1.61243	4.09337	-1.32839
C	1.85153	2.65676	-0.84478
H	2.84979	3.08677	1.04446
H	0.75944	3.19880	2.44246
H	-0.19874	2.57184	1.07918
H	-0.50508	5.03323	1.28648
H	1.25309	5.30950	1.17174
H	0.17123	5.72078	-1.05631
H	-0.53219	4.07665	-1.02899
H	1.54596	4.10474	-2.43681
H	2.48792	4.72880	-1.05884
H	2.77510	2.25000	-1.30685
H	1.01549	2.00617	-1.18503
C	3.43554	-0.58222	-0.83800
C	4.72528	-1.29355	-1.27404
C	5.94056	-0.35432	-1.20042
C	6.09421	0.25489	0.20327
C	4.80959	0.97627	0.65010
C	3.59432	0.03982	0.56396
H	2.56756	-1.27378	-0.84003
H	3.18941	0.21699	-1.57072
H	4.60767	-1.70183	-2.30124
H	4.90005	-2.17132	-0.60923
H	6.86861	-0.89145	-1.49233
H	5.80865	0.46822	-1.94109
H	6.95646	0.95564	0.23257
H	6.32534	-0.55673	0.93102
H	4.92207	1.36827	1.68374
H	4.64034	1.85694	-0.00936
H	3.76038	-0.79843	1.28206
C	0.77105	-5.25040	-1.03905
C	0.03035	-6.52078	-1.50822
H	0.63961	-7.08950	-2.24199
H	-0.19251	-7.19680	-0.65537
H	-0.93346	-6.26210	-1.99517
C	2.09635	-5.65889	-0.36379
H	1.91645	-6.27807	0.54116
H	2.72438	-6.25349	-1.05987
H	2.69429	-4.77420	-0.05977
C	1.07435	-4.36767	-2.26919
H	1.64798	-4.94374	-3.02606
H	0.13647	-4.01534	-2.74696
H	1.66825	-3.47537	-1.99024

L7-TS-R(s-cis)

C	-2.07241	-1.33619	-2.76378
O	-1.76846	-2.58437	-2.61863
Cu	-0.28088	-0.96943	-0.31886
C	-1.91434	-0.32505	-1.13820
C	-2.77478	0.55904	-0.87536
H	-0.63581	-2.81079	-1.86403
O	0.17594	-2.84140	-1.15937
C	0.00740	-3.90912	-0.23331
H	1.01874	-4.10886	0.19156
C	-0.90666	-3.48463	0.94107
H	-0.79370	-4.25399	1.75053
N	-0.51125	-2.15802	1.48677
P	1.46605	0.24037	0.24451
C	-2.39855	-3.29493	0.60118
H	-2.52006	-2.98115	-0.45640
H	-2.95185	-4.24610	0.72439
C	-2.89588	-2.18160	1.56020
H	-3.68024	-2.53616	2.25766
H	-3.31306	-1.34032	0.97610
C	-1.64331	-1.72846	2.33358
H	-1.59341	-0.63494	2.50870
H	-1.57964	-2.23590	3.33025
C	0.78154	-2.18390	2.19199
H	1.55351	-2.50651	1.46236
H	0.75333	-2.96678	2.99222
C	1.57248	0.28466	2.08816
C	1.98697	1.44313	2.77936
C	1.19245	-0.86849	2.83620
C	2.01801	1.48692	4.18268
H	2.29318	2.33398	2.21070
C	1.23222	-0.80538	4.24322
C	1.63422	0.35743	4.92006
H	2.34432	2.40494	4.69615
H	0.93716	-1.69738	4.81978
H	1.64873	0.37707	6.02083
C	-3.83525	1.46967	-0.65362
C	-5.08007	1.00223	-0.14022
C	-3.70908	2.84838	-0.98624
C	-6.15271	1.88593	0.02968
C	-4.78699	3.72348	-0.80776
C	-6.01121	3.24796	-0.29968

H	-5.18438	-0.06782	0.09244
H	-2.75268	3.20840	-1.39213
H	-7.11178	1.51105	0.42069
H	-4.67590	4.78727	-1.07068
H	-6.85719	3.93951	-0.16228
C	-1.13581	-0.48238	-3.62064
C	0.03652	-1.10081	-4.10027
C	-1.35361	0.88021	-3.92523
C	0.97108	-0.38158	-4.86354
C	-0.43814	1.58796	-4.71647
C	0.73400	0.96483	-5.18432
H	0.20405	-2.15996	-3.86215
H	-2.24705	1.38423	-3.53642
H	1.88442	-0.88384	-5.22026
H	-0.63463	2.64459	-4.95827
H	1.45808	1.52896	-5.79334
C	-3.61241	-1.16213	-2.87118
O	-4.40221	-1.88434	-2.29517
O	-3.98763	-0.14197	-3.68365
C	-5.40330	0.08870	-3.76909
H	-5.93493	-0.83873	-4.06195
H	-5.79926	0.43444	-2.79248
H	-5.53608	0.87491	-4.53425
C	-0.50665	-5.18946	-0.90200
H	-0.76718	-5.91043	-0.09404
H	-1.45735	-4.94655	-1.42441
C	1.44152	2.02237	-0.29268
C	0.11541	2.65506	0.18439
C	-0.07226	4.07774	-0.36286
C	0.02347	4.10078	-1.89568
C	1.35173	3.49632	-2.36963
C	1.54224	2.07175	-1.83129
H	2.30619	2.57258	0.14483
H	0.05801	2.65468	1.29289
H	-0.71564	2.00585	-0.16973
H	-1.05070	4.48045	-0.02262
H	0.70779	4.75011	0.06359
H	-0.09850	5.13552	-2.28223
H	-0.81030	3.49826	-2.32369
H	1.40077	3.47867	-3.47839
H	2.19358	4.14021	-2.02416
H	2.51789	1.66948	-2.17230
H	0.76222	1.40412	-2.25865
C	3.07421	-1.15959	-1.61253
C	4.40231	-1.86133	-1.93502
C	5.59773	-0.90343	-1.80360
C	5.64725	-0.25858	-0.40850
C	4.32826	0.46080	-0.07291
C	3.13070	-0.49108	-0.22329
H	2.22419	-1.87285	-1.64314
H	2.86097	-0.39439	-2.39050
H	4.36097	-2.29934	-2.95533
H	4.54119	-2.71748	-1.23453
H	6.55021	-1.43450	-2.01726
H	5.50702	-0.10116	-2.57207
H	6.49838	0.45242	-0.33532
H	5.83289	-1.04946	0.35427
H	4.36214	0.87853	0.95639
H	4.20139	1.32322	-0.76472
H	3.24976	-1.30563	0.53022
C	0.44390	-5.90918	-1.89901
C	-0.26932	-7.20122	-2.35214
H	0.35294	-7.76643	-3.07786
H	-0.48021	-7.87097	-1.49102
H	-1.23691	-6.96799	-2.84436
C	1.77472	-6.28064	-1.21383
H	1.60535	-6.88869	-0.29899
H	2.41637	-6.87411	-1.89866
H	2.35319	-5.37855	-0.92366
C	0.73115	-5.03866	-3.14227
H	1.30039	-5.62132	-3.89779
H	-0.21206	-4.68963	-3.61103
H	1.32187	-4.14083	-2.87628

L7-PC-R(s-cis)

C	-2.05914	-0.53505	-2.12744
O	-1.82987	-1.90985	-2.07731
Cu	-0.36661	-0.29644	0.36285
C	-1.98201	0.11161	-0.78692
C	-2.10088	0.86343	0.21587
H	-1.00018	-2.05180	-1.44995
O	0.14832	-2.01116	-0.51433
C	0.28106	-3.03823	0.42673
H	1.32051	-3.01834	0.85843
C	-0.68824	-2.83920	1.61936
H	-0.45721	-3.60714	2.40333
N	-0.53279	-1.48418	2.23277
P	1.46247	0.89761	0.93247
C	-2.18809	-2.89370	1.25207
H	-2.32348	-2.62364	0.18513
H	-2.58419	-3.91873	1.38862
C	-2.88273	-1.85654	2.17384
H	-3.68456	-2.29997	2.79644
H	-3.33948	-1.04464	1.57533
C	-1.75030	-1.30336	3.05056
H	-1.88048	-0.24253	3.33717
H	-1.65155	-1.90084	3.99391
C	0.70536	-1.35408	3.01728
H	1.54398	-1.66874	2.36458
H	0.68325	-2.07717	3.87204
C	1.40690	1.12635	2.76307
C	1.67456	2.36983	3.37416
C	0.96515	0.03705	3.57411
C	1.47868	2.56430	4.75159
H	2.03773	3.20920	2.76331
C	0.78086	0.25115	4.95341
C	1.01958	1.50289	5.54516
H	1.68840	3.54751	5.20129
H	0.43757	-0.59110	5.57622
H	0.85471	1.64283	6.62473
C	-2.68241	1.79675	1.13894
C	-3.93324	2.38447	0.81253
C	-2.04669	2.14723	2.35490
C	-4.52174	3.30408	1.69076
C	-2.64566	3.06687	3.22604
C	-3.88388	3.64832	2.89844
H	-4.41274	2.10075	-0.13693
H	-1.07359	1.69848	2.59674
H	-5.49079	3.75900	1.43112
H	-2.13721	3.33065	4.16654
H	-4.35375	4.37099	3.58395
C	-1.03451	0.22703	-3.02182
C	0.16578	-0.41372	-3.37227
C	-1.26995	1.55162	-3.43982
C	1.11903	0.26080	-4.15367
C	-0.32543	2.21413	-4.24097
C	0.87228	1.57015	-4.60120
H	0.34240	-1.43864	-3.02129
H	-2.19934	2.05997	-3.13832
H	2.05967	-0.24690	-4.41999
H	-0.52395	3.24173	-4.58402
H	1.61317	2.09103	-5.22798
C	-3.47620	-0.26491	-2.70812
O	-4.21015	0.64686	-2.36224
O	-3.76804	-1.13929	-3.68403
C	-5.03709	-0.94023	-4.32732

H	-5.86634	-1.03537	-3.59709
H	-5.08994	0.06740	-4.78814
H	-5.11086	-1.72550	-5.10077
C	0.06595	-4.43093	-0.19543
H	-0.02436	-5.17454	0.62985
H	-0.91623	-4.42117	-0.71779
C	1.66108	2.60782	0.20963
C	0.38119	3.43729	0.43718
C	0.47448	4.82327	-0.21587
C	0.75579	4.70350	-1.71991
C	2.01791	3.86966	-1.97725
C	1.94051	2.49067	-1.30429
H	2.52717	3.11260	0.69726
H	0.15203	3.53565	1.51656
H	-0.47152	2.88170	-0.00822
H	-0.46847	5.38212	-0.03525
H	1.28779	5.41033	0.27086
H	0.84928	5.70812	-2.18580
H	-0.10955	4.20265	-2.20819
H	2.17999	3.73443	-3.06741
H	2.90798	4.41736	-1.58932
H	2.88203	1.93622	-1.48923
H	1.12869	1.89062	-1.77108
C	3.14506	-0.76338	-0.63644
C	4.43087	-1.59816	-0.72552
C	5.68476	-0.72794	-0.53984
C	5.63422	0.05121	0.78537
C	4.35224	0.89667	0.89666
C	3.10527	0.02108	0.69056
H	2.22903	-1.38722	-0.72525
H	3.11031	-0.05511	-1.49268
H	4.46586	-2.14123	-1.69400
H	4.41178	-2.38194	0.06683
H	6.60428	-1.35031	-0.58551
H	5.75393	-0.00455	-1.38503
H	6.52968	0.70080	0.89261
H	5.66653	-0.66965	1.63457
H	4.30640	1.40967	1.88165
H	4.37737	1.69390	0.12080
H	3.09351	-0.73348	1.51140
C	1.14555	-4.95399	-1.18185
C	0.70708	-6.36085	-1.64210
H	1.44231	-6.79571	-2.35225
H	0.61323	-7.05742	-0.78145
H	-0.27716	-6.32198	-2.15514
C	2.51306	-5.06110	-0.47744
H	2.45468	-5.70798	0.42433
H	3.27477	-5.49675	-1.15810
H	2.88651	-4.06637	-0.15946
C	1.27524	-4.04352	-2.42097
H	1.97917	-4.48896	-3.15662
H	0.29362	-3.90671	-2.92045
H	1.64309	-3.03886	-2.13882

L7-AC-S(s-trans)

C	-2.15250	-0.79085	-2.23691
O	-1.74593	-1.86931	-1.76097
Cu	0.36869	-0.24085	0.58306
C	-1.14095	0.71624	0.00912
C	-2.09851	1.46163	-0.30814
H	-0.18576	-1.99551	-1.13935
O	0.63395	-2.06547	-0.55748
C	0.54779	-3.22793	0.26939
H	1.59778	-3.45434	0.56031
C	-0.23058	-2.92655	1.57134
H	-0.08626	-3.80697	2.25322
N	0.29070	-1.71098	2.24476
P	2.23117	0.74517	1.26083
C	-1.73916	-2.63340	1.40914
H	-1.94124	-2.25402	0.38894
H	-2.33077	-3.55973	1.54821
C	-2.06548	-1.54008	2.46111
H	-2.88362	-1.83271	3.14850
H	-2.35559	-0.60258	1.94722
C	-0.74819	-1.32993	3.22185
H	-0.58783	-0.28757	3.56210
H	-0.68797	-1.99775	4.12057
C	1.61820	-1.91598	2.84490
H	2.32666	-2.15017	2.02279
H	1.59205	-2.81656	3.51109
C	2.49778	0.50385	3.07235
C	3.02456	1.52269	3.89451
C	2.14390	-0.74455	3.65813
C	3.19434	1.33221	5.27542
H	3.30599	2.48844	3.44788
C	2.31926	-0.91812	5.04555
C	2.83589	0.10599	5.85543
H	3.60628	2.14373	5.89564
H	2.04050	-1.88407	5.49777
H	2.95761	-0.05624	6.93771
C	-3.19116	2.30063	-0.65704
C	-4.44124	2.18493	0.01152
C	-3.07492	3.25103	-1.71082
C	-5.52770	2.98302	-0.36329
C	-4.16857	4.04649	-2.07756
C	-5.39927	3.91746	-1.40876
H	-4.54104	1.44229	0.81684
H	-2.11850	3.32463	-2.24778
H	-6.49103	2.87224	0.15961
H	-4.06133	4.77297	-2.89898
H	-6.25746	4.54311	-1.70079
C	-3.59528	-0.45727	-2.24822
C	-4.43496	-1.21550	-1.39426
C	-4.16978	0.55592	-3.05557
C	-5.80875	-0.96367	-1.34123
C	-5.55143	0.79195	-3.00784
C	-6.37247	0.04053	-2.15316
H	-3.97465	-1.99366	-0.76859
H	-3.52254	1.14906	-3.71146
H	-6.44879	-1.54941	-0.66280
H	-5.98525	1.58726	-3.63229
H	-7.45435	0.24250	-2.10894
C	-1.13313	0.13833	-2.92046
O	-1.36964	1.17473	-3.52228
O	0.09839	-0.40931	-2.83711
C	1.16057	0.37018	-3.39666
H	0.91530	0.68470	-4.43081
H	1.33186	1.27412	-2.77787
H	2.05405	-0.27779	-3.37980
C	-0.02793	-4.44108	-0.46745
H	-0.14500	-5.25439	0.28418
H	-1.05275	-4.18999	-0.81699
C	2.05830	2.59648	1.05077
C	0.82209	3.06743	1.85036
C	0.47372	4.53641	1.56713
C	0.26559	4.78050	0.06577
C	1.50974	4.35731	-0.72764
C	1.87043	2.88917	-0.45451
H	2.97058	3.12144	1.41630
H	0.98122	2.91113	2.93736
H	-0.03689	2.42299	1.55704
H	-0.43850	4.81577	2.13645
H	1.29276	5.19584	1.93935
H	0.02362	5.84746	-0.12833
H	-0.60865	4.18490	-0.27965
H	1.34715	4.50133	-1.81763

H	2.36885	5.01071	-0.44790
H	2.78544	2.60883	-1.01960
H	1.04510	2.23470	-0.81668
C	3.79214	-0.23616	-0.87539
C	5.12690	-0.80276	-1.38334
C	6.27182	0.20731	-1.20002
C	6.38493	0.66948	0.26231
C	5.05178	1.23959	0.78043
C	3.90993	0.22999	0.58863
H	2.97085	-0.97880	-0.96483
H	3.49830	0.63034	-1.50791
H	5.03598	-1.10107	-2.45015
H	5.36425	-1.73402	-0.81875
H	7.23480	-0.22782	-1.54363
H	6.08050	1.09400	-1.84777
H	7.19226	1.42556	0.37096
H	6.67910	-0.19666	0.89854
H	5.14110	1.52282	1.85098
H	4.81303	2.17221	0.22113
H	4.14221	-0.67019	1.20612
C	0.78528	-5.00617	-1.66706
C	0.10406	-6.32336	-2.09615
H	0.62066	-6.77232	-2.97059
H	0.11335	-7.07145	-1.27467
H	-0.95454	-6.14807	-2.38249
C	2.24054	-5.30044	-1.25020
H	2.28385	-5.96667	-0.36149
H	2.78917	-5.80333	-2.07409
H	2.79368	-4.36875	-1.00922
C	0.78156	-4.03218	-2.86583
H	1.27755	-4.49985	-3.74305
H	-0.25365	-3.76160	-3.15828
H	1.30871	-3.09012	-2.62416

L7-TS-S(s-trans)

C	-2.21483	-1.01412	-2.55377
O	-2.04298	-2.29325	-2.42889
Cu	-0.25869	-0.84177	-0.16179
C	-1.91546	-0.14306	-0.88864
C	-2.79858	0.67291	-0.50779
H	-0.82410	-2.58419	-1.80582
O	0.05558	-2.64321	-1.18027
C	0.01390	-3.75354	-0.29734
H	1.06063	-3.90426	0.05757
C	-0.84539	-3.44202	0.95314
H	-0.68404	-4.26962	1.69350
N	-0.44378	-2.15312	1.58062
P	1.50659	0.30984	0.45953
C	-2.35213	-3.25079	0.68934
H	-2.50000	-2.87033	-0.34341
H	-2.89113	-4.21473	0.77200
C	-2.82603	-2.20153	1.72775
H	-3.58666	-2.60235	2.42647
H	-3.26253	-1.32691	1.20994
C	-1.55261	-1.78791	2.48640
H	-1.50533	-0.70710	2.72795
H	-1.45502	-2.35576	3.44717
C	0.86198	-2.22015	2.25853
H	1.62184	-2.49261	1.49648
H	0.85047	-3.05299	3.00721
C	1.63388	0.25469	2.29931
C	2.05280	1.37239	3.05151
C	1.27758	-0.94645	2.97970
C	2.10958	1.32899	4.45418
H	2.34164	2.29856	2.53211
C	1.34374	-0.97087	4.38678
C	1.74928	0.15196	5.12645
H	2.43772	2.21612	5.01824
H	1.06685	-1.89939	4.91250
H	1.78476	0.10357	6.22586
C	-3.90584	1.51736	-0.26303
C	-5.14121	0.96575	0.17852
C	-3.84513	2.90663	-0.56696
C	-6.27600	1.77538	0.29359
C	-4.98374	3.70944	-0.43955
C	-6.20239	3.14740	-0.01309
H	-5.19445	-0.11276	0.38608
H	-2.89541	3.32584	-0.92860
H	-7.23218	1.33360	0.61478
H	-4.92765	4.78131	-0.68639
H	-7.09938	3.78032	0.07569
C	-3.64708	-0.58092	-2.86462
C	-4.66793	-1.50751	-2.57102
C	-4.00103	0.69245	-3.36124
C	-6.01767	-1.16448	-2.74696
C	-5.35187	1.02937	-3.54179
C	-6.36658	0.10842	-3.23038
H	-4.37089	-2.49930	-2.20021
H	-3.21018	1.42147	-3.58137
H	-6.80371	-1.89831	-2.50568
H	-5.61334	2.03074	-3.91893
H	-7.42515	0.38256	-3.36296
C	-1.10036	-0.23688	-3.30733
O	-1.05418	0.96199	-3.54047
O	-0.13556	-1.09616	-3.70876
C	0.96231	-0.50689	-4.41466
H	0.62115	-0.06946	-5.37531
H	1.43285	0.29578	-3.81315
H	1.68080	-1.32652	-4.59520
C	-0.44207	-5.04113	-0.99241
H	-0.48098	-5.83913	-0.21611
H	-1.48447	-4.90414	-1.35421
C	1.45133	2.11577	0.00125
C	0.13711	2.70851	0.55892
C	-0.13331	4.12552	0.03198
C	-0.13135	4.15574	-1.50340
C	1.20076	3.62853	-2.05262
C	1.48180	2.20886	-1.54016
H	2.32878	2.65932	0.42083
H	0.14742	2.70129	1.66863
H	-0.69452	2.03727	0.25000
H	-1.10485	4.48726	0.43269
H	0.64383	4.82683	0.41553
H	-0.33185	5.18267	-1.87730
H	-0.94808	3.50379	-1.88841
H	1.18737	3.61979	-3.16289
H	2.02723	4.31089	-1.74572
H	2.45955	1.85123	-1.92685
H	0.70212	1.52726	-1.94495
C	3.12255	-1.01967	-1.44772
C	4.44174	-1.73666	-1.77236
C	5.64745	-0.79577	-1.61046
C	5.68982	-0.16951	-0.20630
C	4.37233	0.55137	0.13377
C	3.17392	-0.39421	-0.04025
H	2.25244	-1.70635	-1.52377
H	2.95119	-0.21706	-2.19764
H	4.40602	-2.15427	-2.80146
H	4.55955	-2.60842	-1.08776
H	6.59569	-1.33561	-1.82043
H	5.57630	0.01782	-2.36902
H	6.54461	0.53534	-0.11882
H	5.86657	-0.97126	0.54704
H	4.40286	0.94972	1.17071
H	4.25022	1.42706	-0.54271
H	3.29692	-1.23132	0.68722

C	0.42973	-5.55260	-2.17622
C	-0.04997	-6.98134	-2.51089
H	0.51009	-7.39695	-3.37515
H	0.09129	-7.67025	-1.65039
H	-1.12874	-6.98583	-2.77510
C	1.91982	-5.59624	-1.78089
H	2.08165	-6.18808	-0.85375
H	2.52347	-6.06419	-2.58684
H	2.32537	-4.57633	-1.61564
C	0.25909	-4.66608	-3.42958
H	0.79919	-5.11316	-4.29171
H	-0.80982	-4.56169	-3.70670
H	0.64376	-3.64371	-3.25949

L7-PC-S(s-trans)

C	-1.90803	-0.48105	-2.10609
O	-1.80879	-1.86652	-2.02602
Cu	-0.21183	-0.28807	0.37401
C	-1.84453	0.15655	-0.75385
C	-2.01408	0.80878	0.30739
H	-0.91555	-2.03737	-1.49573
O	0.28659	-1.93824	-0.62764
C	0.50481	-3.00169	0.25434
H	1.56223	-2.96233	0.63977
C	-0.41611	-2.90012	1.49955
H	-0.11817	-3.69212	2.23489
N	-0.28853	-1.56800	2.17217
P	1.62039	0.92147	0.91535
C	-1.92819	-3.00289	1.19444
H	-2.12398	-2.70439	0.14520
H	-2.27748	-4.04661	1.31675
C	-2.62483	-2.02238	2.17412
H	-3.39567	-2.51092	2.80207
H	-3.12208	-1.20336	1.61895
C	-1.48395	-1.46865	3.03655
H	-1.64008	-0.42710	3.37642
H	-1.33173	-2.10416	3.94723
C	0.96688	-1.42461	2.92843
H	1.79656	-1.68296	2.24145
H	0.99397	-2.17954	3.75479
C	1.55148	1.09399	2.75248
C	1.73239	2.33531	3.39876
C	1.17746	-0.04394	3.53107
C	1.52616	2.47644	4.78117
H	2.03176	3.21593	2.81176
C	0.98654	0.11681	4.91658
C	1.14563	1.36336	5.54502
H	1.66707	3.45905	5.25817
H	0.69717	-0.76311	5.51416
H	0.97650	1.46021	6.62862
C	-2.60302	1.64795	1.31273
C	-1.90860	1.98048	2.50198
C	-3.91134	2.16134	1.11216
C	-2.50305	2.80780	3.46379
C	-4.49798	2.98772	2.07992
C	-3.79902	3.31397	3.25793
H	-0.89456	1.58737	2.65233
H	-4.44993	1.90127	0.18809
H	-1.94511	3.05626	4.38001
H	-5.51293	3.38224	1.91440
H	-4.26666	3.96277	4.01499
C	-3.22335	-0.06629	-2.79039
C	-4.05287	-1.05702	-3.34110
C	-3.59803	1.29030	-2.86105
C	-5.26175	-0.69047	-3.95805
C	-4.80530	1.65112	-3.47932
C	-5.64154	0.66080	-4.02768
H	-3.73111	-2.10515	-3.26666
H	-2.93378	2.05654	-2.43260
H	-5.91339	-1.46874	-4.38649
H	-5.09615	2.71229	-3.53340
H	-6.59039	0.94462	-4.50996
C	-0.73324	0.15602	-2.92711
O	-0.39170	1.32629	-2.84233
O	-0.15890	-0.74238	-3.73744
C	0.97985	-0.27130	-4.47551
H	0.72644	0.63858	-5.05582
H	1.81028	-0.03337	-3.77967
H	1.27055	-1.10009	-5.14491
C	0.31225	-4.36811	-0.43098
H	0.35060	-5.16202	0.35051
H	-0.71603	-4.39544	-0.85478
C	1.78031	2.65880	0.24359
C	0.41960	3.37466	0.38287
C	0.42819	4.77117	-0.25479
C	0.84320	4.70010	-1.73108
C	2.21086	4.01759	-1.87263
C	2.21069	2.61660	-1.23930
H	2.55922	3.20704	0.82305
H	0.11425	3.43944	1.44667
H	-0.34561	2.74824	-0.12378
H	-0.57808	5.23012	-0.14922
H	1.13541	5.43147	0.29950
H	0.86415	5.71520	-2.18288
H	0.08831	4.10756	-2.29372
H	2.49818	3.93710	-2.94298
H	2.99135	4.64571	-1.38293
H	3.21944	2.16775	-1.33669
H	1.50344	1.96870	-1.80038
C	3.36579	-0.62946	-0.69544
C	4.66977	-1.43483	-0.79889
C	5.90174	-0.55355	-0.53450
C	5.80745	0.14448	0.83273
C	4.50992	0.96282	0.96146
C	3.28202	0.08410	0.66922
H	2.46493	-1.26604	-0.83570
H	3.33964	0.12369	-1.51263
H	4.73840	-1.92268	-1.79444
H	4.64730	-2.26102	-0.05134
H	6.83338	-1.15605	-0.59619
H	5.97551	0.21961	-1.33406
H	6.68960	0.79944	1.00022
H	5.83149	-0.62548	1.63798
H	4.42804	1.41052	1.97533
H	4.54368	1.80953	0.24097
H	3.26319	-0.71506	1.44567
C	1.31397	-4.75742	-1.55289
C	0.92445	-6.16646	-2.04918
H	1.60682	-6.50711	-2.85690
H	0.97058	-6.91376	-1.22796
H	-0.10933	-6.17243	-2.45518
C	2.75117	-4.80159	-0.99735
H	2.83214	-5.49801	-0.13491
H	3.46555	-5.14312	-1.77614
H	3.08617	-3.80059	-0.65922
C	1.25068	-3.77541	-2.74248
H	1.90776	-4.12881	-3.56682
H	0.21785	-3.68157	-3.13598
H	1.56123	-2.75900	-2.43688

L7-AC-S(s-cis)

C	-2.07940	-0.75879	-2.16558
O	-1.67190	-1.84255	-1.70614
Cu	0.31073	-0.15617	0.68079
C	-1.24705	0.75969	0.17037
C	-2.26893	1.42476	-0.12731

H	-0.15126	-1.94182	-1.05007
O	0.64918	-1.97700	-0.43750
C	0.60654	-3.13577	0.39168
H	1.66212	-3.30712	0.70083
C	-0.20046	-2.86175	1.68104
H	-0.03785	-3.73413	2.36898
N	0.27345	-1.62541	2.35523
P	2.25937	0.74443	1.23164
C	-1.71392	-2.61620	1.49643
H	-1.90999	-2.23412	0.47565
H	-2.27857	-3.56176	1.61721
C	-2.09266	-1.54266	2.55143
H	-2.89678	-1.87685	3.23655
H	-2.42617	-0.61816	2.04084
C	-0.78836	-1.27293	3.31765
H	-0.67103	-0.22125	3.64678
H	-0.70946	-1.92791	4.22463
C	1.59554	-1.79116	2.97804
H	2.30953	-2.07463	2.17724
H	1.56457	-2.65134	3.69570
C	2.51801	0.62113	3.05527
C	3.05486	1.68186	3.81521
C	2.12156	-0.57455	3.72114
C	3.18901	1.58651	5.21010
H	3.37555	2.60445	3.30813
C	2.26281	-0.65277	5.12044
C	2.78577	0.41501	5.86804
H	3.60992	2.42970	5.78013
H	1.95281	-1.57856	5.63253
H	2.87946	0.32826	6.96172
C	-3.45035	2.14683	-0.44898
C	-4.70589	1.76726	0.10243
C	-3.42366	3.24301	-1.35680
C	-5.87771	2.44587	-0.24960
C	-4.60107	3.91816	-1.70345
C	-5.83497	3.52204	-1.15599
H	-4.73831	0.91651	0.79869
H	-2.45693	3.54785	-1.78258
H	-6.84059	2.12788	0.18082
H	-4.55766	4.76355	-2.40891
H	-6.76016	4.05196	-1.43206
C	-3.53781	-0.49958	-2.27301
C	-4.38190	-1.36021	-1.52550
C	-4.13256	0.50840	-3.07120
C	-5.77168	-1.21740	-1.57026
C	-5.52775	0.63825	-3.12219
C	-6.35114	-0.21779	-2.37496
H	-3.91118	-2.13603	-0.90497
H	-3.49822	1.18506	-3.65246
H	-6.41120	-1.88647	-0.97335
H	-5.97350	1.43116	-3.74136
H	-7.44568	-0.10052	-2.40917
C	-0.97364	0.19115	-2.67071
O	0.20257	-0.12266	-2.63610
O	-1.42346	1.35254	-3.19763
C	-0.40386	2.28995	-3.57338
H	0.11957	2.65175	-2.66639
H	0.33723	1.82034	-4.25026
H	-0.92558	3.12449	-4.07516
C	0.11945	-4.38403	-0.34806
H	0.08926	-5.20997	0.39828
H	-0.92981	-4.22401	-0.67917
C	2.27066	2.57668	0.86391
C	1.03866	3.21157	1.54812
C	0.81507	4.66843	1.11805
C	0.69623	4.77983	-0.40839
C	1.94622	4.20291	-1.08767
C	2.19214	2.74525	-0.66912
H	3.20650	3.05302	1.23669
H	1.13106	3.14328	2.65190
H	0.14741	2.60539	1.26868
H	-0.09950	5.06340	1.60956
H	1.66220	5.30361	1.46828
H	0.53461	5.83518	-0.71666
H	-0.20046	4.20672	-0.73863
H	1.86001	4.26491	-2.19487
H	2.83141	4.82187	-0.81316
H	3.11887	2.36514	-1.14747
H	1.36298	2.09842	-1.03660
C	3.67924	-0.53414	-0.85441
C	4.94340	-1.27473	-1.31687
C	6.19474	-0.38928	-1.19380
C	6.36526	0.14824	0.23712
C	5.10616	0.89424	0.71547
C	3.85891	0.00715	0.57862
H	2.78269	-1.18618	-0.90028
H	3.47116	0.30884	-1.54904
H	4.81552	-1.62985	-2.36183
H	5.07910	-2.18839	-0.69214
H	7.10284	-0.94862	-1.50630
H	6.10016	0.47101	-1.89624
H	7.25259	0.81466	0.30149
H	6.56319	-0.70424	0.92705
H	5.22836	1.23015	1.76778
H	4.97209	1.81224	0.10006
H	3.99860	-0.87470	1.24868
C	0.96361	-4.85934	-1.56789
C	0.48288	-6.28187	-1.92669
H	1.01750	-6.66916	-2.81961
H	0.65711	-6.99273	-1.09056
H	-0.60403	-6.28813	-2.15559
C	2.46400	-4.90328	-1.21388
H	2.65309	-5.51202	-0.30308
H	3.04666	-5.35348	-2.04472
H	2.87074	-3.88530	-1.04168
C	0.75510	-3.94424	-2.79511
H	1.29504	-4.35531	-3.67476
H	-0.32034	-3.87077	-3.05783
H	1.12098	-2.91616	-2.61318

L7-TS-S(s-cis)

C	-2.08811	-1.21139	-2.71025
O	-1.97788	-2.46488	-2.43019
Cu	-0.23564	-0.86015	-0.21076
C	-1.83150	-0.17288	-1.08549
C	-2.71740	0.62582	-0.67654
H	-0.75283	-2.72724	-1.73898
O	0.06446	-2.76182	-1.05795
C	-0.10514	-3.81187	-0.11636
H	0.91273	-4.04037	0.27635
C	-0.95612	-3.34189	1.08773
H	-0.86488	-4.11892	1.89280
N	-0.46401	-2.04215	1.61844
P	1.52433	0.32003	0.37525
C	-2.44148	-3.05515	0.79666
H	-2.55375	-2.71565	-0.25496
H	-3.05120	-3.97170	0.91777
C	-2.84104	-1.92793	1.78485
H	-3.61072	-2.25219	2.51272
H	-3.24466	-1.05861	1.23267
C	-1.53400	-1.54903	2.50860
H	-1.41482	-0.46026	2.67984
H	-1.46280	-2.05919	3.50336
C	0.85267	-2.15032	2.26859
H	1.57781	-2.48835	1.49872
H	0.81705	-2.95471	3.04689

C	1.76301	0.27721	2.20528
C	2.29896	1.37844	2.90693
C	1.36597	-0.88184	2.93283
C	2.43117	1.36140	4.30471
H	2.61879	2.27253	2.35034
C	1.50372	-0.87888	4.33557
C	2.02477	0.22807	5.02443
H	2.85140	2.23459	4.82796
H	1.19338	-1.77445	4.89832
H	2.11515	0.20115	6.12146
C	-3.85661	1.38532	-0.30547
C	-5.13992	0.77023	-0.36834
C	-3.77275	2.75174	0.07702
C	-6.29243	1.50657	-0.07192
C	-4.93133	3.47344	0.38955
C	-6.19460	2.85690	0.31334
H	-5.20786	-0.28072	-0.68683
H	-2.78546	3.23158	0.12316
H	-7.27911	1.02277	-0.14358
H	-4.85126	4.53062	0.68830
H	-7.10380	3.43083	0.55194
C	-3.48435	-0.76700	-3.14884
C	-4.48602	-1.76013	-3.14556
C	-3.84008	0.55132	-3.51030
C	-5.81156	-1.44795	-3.49087
C	-5.16304	0.86056	-3.85818
C	-6.15666	-0.13473	-3.84999
H	-4.19132	-2.78056	-2.86175
H	-3.07950	1.34115	-3.49281
H	-6.57886	-2.23884	-3.48054
H	-5.42396	1.89721	-4.12386
H	-7.19552	0.11559	-4.11758
C	-0.85231	-0.64597	-3.46392
O	0.15242	-1.30276	-3.66115
O	-0.99459	0.62824	-3.91380
C	0.09028	1.11475	-4.71797
H	1.03841	1.11605	-4.14484
H	0.22572	0.47967	-5.61693
H	-0.18683	2.14387	-5.00984
C	-0.66783	-5.09028	-0.74621
H	-0.80615	-5.82583	0.07900
H	-1.68111	-4.87663	-1.15021
C	1.38940	2.13213	-0.04434
C	0.14537	2.70992	0.66542
C	-0.14687	4.15203	0.22372
C	-0.30205	4.25029	-1.30128
C	0.94704	3.70483	-2.00711
C	1.24114	2.26107	-1.57472
H	2.30284	2.68019	0.28178
H	0.26482	2.65914	1.76717
H	-0.71996	2.05678	0.41154
H	-1.05990	4.52425	0.73788
H	0.68365	4.81822	0.55315
H	-0.50042	5.29943	-1.60858
H	-1.18521	3.65042	-1.61876
H	0.82268	3.74642	-3.11023
H	1.82196	4.35169	-1.76564
H	2.15442	1.88613	-2.08273
H	0.40239	1.60139	-1.89006
C	2.99098	-0.96564	-1.65678
C	4.30490	-1.59943	-2.13725
C	5.46645	-0.59262	-2.10890
C	5.63727	0.02676	-0.71191
C	4.33179	0.67932	-0.22168
C	3.16696	-0.32224	-0.26578
H	2.17124	-1.71327	-1.62703
H	2.66379	-0.19526	-2.38904
H	4.16963	-2.01666	-3.15754
H	4.55396	-2.46283	-1.47739
H	6.41242	-1.07656	-2.43421
H	5.26195	0.21998	-2.84411
H	6.46011	0.77399	-0.71142
H	5.93615	-0.76939	0.00811
H	4.45920	1.08056	0.80695
H	4.09467	1.54744	-0.87719
H	3.39528	-1.14350	0.45464
C	0.18093	-5.76405	-1.86435
C	-0.41898	-7.16607	-2.10589
H	0.11778	-7.69264	-2.92315
H	-0.35675	-7.79877	-1.19420
H	-1.48858	-7.09509	-2.39690
C	1.65131	-5.91056	-1.42324
H	1.73780	-6.44093	-0.44971
H	2.22658	-6.49208	-2.17407
H	2.14440	-4.92125	-1.32538
C	0.11924	-4.96534	-3.18519
H	0.64684	-5.51991	-3.99074
H	-0.93011	-4.80487	-3.50658
H	0.57894	-3.96400	-3.09029

L7-PC-S(s-cis)

C	-1.98411	-0.44647	-2.05106
O	-1.89018	-1.83277	-1.97847
Cu	-0.22216	-0.27440	0.38493
C	-1.88054	0.18566	-0.69875
C	-2.01852	0.83333	0.36983
H	-0.98374	-2.01041	-1.47336
O	0.24171	-1.92196	-0.63737
C	0.47673	-2.99173	0.23255
H	1.54458	-2.96168	0.58895
C	-0.40888	-2.89087	1.50299
H	-0.09582	-3.68854	2.22585
N	-0.25392	-1.56318	2.17873
P	1.63132	0.92312	0.88180
C	-1.92969	-2.98233	1.24001
H	-2.15331	-2.67717	0.19827
H	-2.28210	-4.02445	1.36693
C	-2.59175	-2.00271	2.24442
H	-3.34850	-2.48946	2.89064
H	-3.09810	-1.17670	1.70813
C	-1.42328	-1.46244	3.07784
H	-1.56288	-0.42244	3.42946
H	-1.24915	-2.10557	3.97921
C	1.02372	-1.43128	2.89906
H	1.83171	-1.69000	2.18678
H	1.07043	-2.19143	3.71973
C	1.61717	1.08501	2.72107
C	1.82415	2.32134	3.36907
C	1.25957	-0.05553	3.50355
C	1.65937	2.45520	4.75774
H	2.11134	3.20377	2.77871
C	1.11021	0.09784	4.89500
C	1.29491	1.33965	5.52585
H	1.81997	3.43407	5.23623
H	0.83339	-0.78406	5.49559
H	1.15820	1.43088	6.61450
C	-2.57317	1.67136	1.39538
C	-1.84329	1.99065	2.56688
C	-3.88178	2.19747	1.23377
C	-2.40327	2.81779	3.54926
C	-4.43391	3.02347	2.22196
C	-3.69974	3.33673	3.38195
H	-0.82913	1.58731	2.68639
H	-4.44795	1.94767	0.32347
H	-1.81800	3.05604	4.45103
H	-5.44940	3.42806	2.08676

H	-4.14041	3.98544	4.15511
C	-3.31513	-0.02237	-2.69830
C	-4.16395	-1.00668	-3.23082
C	-3.68458	1.33636	-2.75376
C	-5.38684	-0.63157	-3.81409
C	-4.90591	1.70573	-3.33847
C	-5.76142	0.72184	-3.86837
H	-3.84570	-2.05671	-3.16912
H	-3.00532	2.09754	-2.34008
H	-6.05354	-1.40483	-4.22822
H	-5.19262	2.76856	-3.38074
H	-6.72120	1.01237	-4.32430
C	-0.82863	0.18729	-2.90140
O	-0.47846	1.35535	-2.82154
O	-0.28148	-0.71105	-3.73043
C	0.83984	-0.24346	-4.49687
H	0.57639	0.67032	-5.06646
H	1.68993	-0.01333	-3.82250
H	1.10762	-1.07109	-5.17718
C	0.25614	-4.35305	-0.45455
H	0.30943	-5.15149	0.32142
H	-0.78317	-4.37071	-0.85111
C	1.78267	2.66342	0.21570
C	0.43248	3.38896	0.39970
C	0.43351	4.78898	-0.23044
C	0.80357	4.72326	-1.71885
C	2.16074	4.03059	-1.90517
C	2.16757	2.62614	-1.27974
H	2.58290	3.20253	0.77433
H	0.15957	3.45019	1.47250
H	-0.35257	2.77145	-0.08718
H	-0.56528	5.25555	-0.09222
H	1.16248	5.44029	0.30604
H	0.81922	5.74080	-2.16535
H	0.02730	4.14016	-2.26198
H	2.41511	3.95386	-2.98409
H	2.96067	4.64959	-1.43566
H	3.16880	2.16883	-1.40975
H	1.43806	1.98760	-1.82275
C	3.31771	-0.63062	-0.78767
C	4.61322	-1.44276	-0.93470
C	5.85781	-0.56950	-0.70507
C	5.80989	0.12348	0.66714
C	4.52172	0.94863	0.83928
C	3.28040	0.07796	0.58157
H	2.40910	-1.26145	-0.90191
H	3.27024	0.12588	-1.60081
H	4.64821	-1.92692	-1.93380
H	4.60920	-2.27190	-0.19008
H	6.78364	-1.17698	-0.79783
H	5.91138	0.20647	-1.50352
H	6.70057	0.77274	0.80979
H	5.85441	-0.64989	1.46824
H	4.47393	1.39236	1.85706
H	4.53812	1.79830	0.12163
H	3.28110	-0.72428	1.35505
C	1.22492	-4.74346	-1.60469
C	0.81165	-6.14670	-2.09826
H	1.46992	-6.48791	-2.92552
H	0.87361	-6.89896	-1.28264
H	-0.23246	-6.14257	-2.47693
C	2.67588	-4.80168	-1.08749
H	2.77420	-5.50391	-0.23159
H	3.36693	-5.14379	-1.88681
H	3.02730	-3.80529	-0.75242
C	1.13774	-3.75431	-2.78684
H	1.76995	-4.10810	-3.63025
H	0.09559	-3.65040	-3.15221
H	1.46421	-2.74198	-2.48431

Listing S3: Absolute electronic energes and Cartesian coordinates of **L7-TS-R(t)-1q** and **L7-TS-S(t)-1q** at the DF-BP86-D3(BJ)/SVP level of theory in hartree (a.u.).

L7-TS-R(t)-1q

E(DF-BP86-D3(BJ)/SVP) -4247.35781666

Cu	-0.22471	-1.00234	-0.37471
C	-1.79892	-0.31183	-1.28570
C	-2.52849	0.65836	-0.94775
H	-0.61234	-2.84689	-1.97181
O	0.17672	-2.87458	-1.26322
C	-0.01741	-3.95838	-0.35914
H	0.98626	-4.18040	0.07192
C	-0.93902	-3.54238	0.81309
H	-0.85070	-4.33170	1.60618
N	-0.52487	-2.23755	1.39421
P	1.45082	0.23614	0.34767
C	-2.42287	-3.31898	0.45494
H	-2.52547	-3.02039	-0.60830
H	-2.99873	-4.25542	0.58843
C	-2.90538	-2.17903	1.39065
H	-3.73875	-2.48725	2.05267
H	-3.24859	-1.31811	0.78544
C	-1.66648	-1.79329	2.21830
H	-1.58279	-0.70733	2.42753
H	-1.65495	-2.32951	3.20180
C	0.74915	-2.31436	2.12882
H	1.53747	-2.60198	1.40145
H	0.69389	-3.14112	2.88197
C	1.54052	0.15476	2.19125
C	1.96693	1.26062	2.95777
C	1.15673	-1.04401	2.85896
C	2.00283	1.20887	4.36047
H	2.27674	2.18571	2.44864
C	1.19734	-1.07604	4.26761
C	1.60974	0.03509	5.02022
H	2.33834	2.08692	4.93427
H	0.89698	-2.00276	4.78342
H	1.62620	-0.02000	6.11978
C	-3.33509	1.77744	-0.62224
C	-3.60149	2.12683	0.73047
C	-3.88410	2.58237	-1.66004
C	-4.37301	3.25638	1.03137
C	-4.66653	3.70002	-1.34411
C	-4.90867	4.04819	-0.00175
H	-3.18014	1.50316	1.53326
H	-3.70255	2.28832	-2.70411
H	-4.56267	3.52158	2.08358
H	-5.09099	4.31136	-2.15616
H	-5.51714	4.93370	0.23932
C	-0.54525	-5.21661	-1.05836
H	-0.81898	-5.95120	-0.26712
H	-1.48928	-4.94780	-1.57963
C	1.29401	2.05423	-0.04853
C	-0.01491	2.57571	0.58348
C	-0.36799	3.99996	0.12997

C	-0.40196	4.11294	-1.40083
C	0.92512	3.64544	-2.01368
C	1.25360	2.20830	-1.58464
H	2.16652	2.61838	0.35313
H	0.03822	2.52610	1.69042
H	-0.82760	1.88446	0.27799
H	-1.35193	4.28501	0.55945
H	0.38118	4.71824	0.53759
H	-0.63212	5.15524	-1.70886
H	-1.22895	3.47839	-1.78961
H	0.88675	3.70003	-3.12290
H	1.74616	4.32779	-1.69255
H	2.21387	1.88010	-2.03534
H	0.46996	1.52270	-1.97463
C	3.18771	-0.95262	-1.54061
C	4.57053	-1.53077	-1.87788
C	5.68015	-0.48061	-1.70709
C	5.66606	0.12356	-0.29331
C	4.28762	0.71518	0.05324
C	3.17780	-0.33194	-0.12973
H	2.39797	-1.72955	-1.61205
H	2.92341	-0.17275	-2.28829
H	4.56935	-1.93566	-2.91246
H	4.77684	-2.39486	-1.20458
H	6.67489	-0.92383	-1.92784
H	5.52800	0.33386	-2.45254
H	6.45125	0.90364	-0.19284
H	5.91596	-0.67030	0.44756
H	4.28302	1.10631	1.09331
H	4.08724	1.58141	-0.61653
H	3.36279	-1.15717	0.59835
C	0.39874	-5.92694	-2.06825
C	-0.33554	-7.19329	-2.55883
H	0.28158	-7.75202	-3.29387
H	-0.56625	-7.88047	-1.71665
H	-1.29429	-6.92897	-3.05276
C	1.71719	-6.34129	-1.38396
H	1.53014	-6.97248	-0.48834
H	2.35426	-6.92571	-2.08076
H	2.30849	-5.45802	-1.06358
C	0.70974	-5.02965	-3.28672
H	1.27112	-5.60405	-4.05440
H	-0.22303	-4.64641	-3.74899
H	1.31536	-4.14993	-2.99559
C	-1.67418	-1.01904	-5.35473
C	-1.30372	-0.58861	-3.91483
C	0.20676	-0.74500	-3.70077
C	1.01255	0.07017	-4.71735
C	0.64423	-0.32561	-6.15683
C	-0.87095	-0.21679	-6.39160
H	-2.76153	-0.87892	-5.51989
H	-1.45882	-2.10551	-5.46261
H	-1.58524	0.47867	-3.79239
H	0.45927	-0.45372	-2.65752
H	0.47072	-1.82110	-3.78996
H	2.10258	-0.06588	-4.54397
H	0.80436	1.15501	-4.56792
H	1.19832	0.29955	-6.89048
H	0.96485	-1.37814	-6.33297
H	-1.12920	-0.55586	-7.41841
H	-1.17344	0.85403	-6.33155
C	-2.11208	-1.41410	-2.89569
O	-1.80383	-2.64997	-2.72636
C	-3.62644	-1.08316	-2.88347
O	-4.12571	-0.15242	-3.50378
O	-4.29134	-1.97160	-2.12555
C	-5.73460	-1.82537	-1.85608
C	-5.99158	-0.49737	-1.13197
H	-7.05554	-0.44079	-0.82346
H	-5.36154	-0.42319	-0.22324
H	-5.76394	0.36560	-1.78363
C	-6.02766	-3.01564	-0.93624
H	-7.10481	-3.04188	-0.67491
H	-5.75959	-3.96769	-1.43641
H	-5.43971	-2.93698	-0.00053
C	-6.52324	-1.93844	-3.16803
H	-6.29990	-1.08632	-3.83562
H	-6.26431	-2.88138	-3.69141
H	-7.61092	-1.95058	-2.94970

L7-TS-S(t)-1q

E(DF-BP86-D3(BJ)/SVP) -4247.35390860

Cu	-0.23536	-0.98239	-0.22918
C	-1.79186	-0.27975	-1.16999
C	-2.44112	0.79160	-1.01844
H	-0.66820	-2.78171	-1.88117
O	0.16665	-2.79873	-1.22334
C	0.07366	-3.92738	-0.35799
H	1.11153	-4.13074	-0.00522
C	-0.77887	-3.60046	0.89265
H	-0.62203	-4.43012	1.63210
N	-0.35849	-2.32120	1.51995
P	1.43250	0.25829	0.53992
C	-2.28779	-3.39331	0.63940
H	-2.44773	-3.07979	-0.41170
H	-2.84223	-4.33962	0.79239
C	-2.72379	-2.27406	1.62212
H	-3.55914	-2.57757	2.28325
H	-3.04480	-1.37690	1.05704
C	-1.45886	-1.95444	2.43194
H	-1.36977	-0.89062	2.73065
H	-1.40528	-2.57577	3.36314
C	0.95476	-2.41250	2.17663
H	1.71016	-2.62043	1.38934
H	0.96555	-3.29337	2.86777
C	1.62547	0.07716	2.36968
C	2.04183	1.15156	3.18489
C	1.35631	-1.18369	2.97388
C	2.17860	1.00484	4.57479
H	2.26642	2.12664	2.72747
C	1.49753	-1.31250	4.37005
C	1.90059	-0.23327	5.17248
H	2.50412	1.86023	5.18731
H	1.28353	-2.28880	4.83491
H	1.99835	-0.36256	6.26153
C	-3.15262	2.01650	-0.98523
C	-3.63258	2.57365	0.23185
C	-3.36994	2.72580	-2.20261
C	-4.28606	3.81267	0.23159
C	-4.04541	3.95196	-2.18824
C	-4.49678	4.50637	-0.97491
H	-3.46804	2.02342	1.17057
H	-2.99188	2.28156	-3.13656
H	-4.64154	4.24128	1.18206
H	-4.21204	4.49044	-3.13467
H	-5.01508	5.47794	-0.96941
C	-0.45937	-5.17762	-1.06992
H	-0.64795	-5.95149	-0.29126
H	-1.44755	-4.92628	-1.51100
C	1.26796	2.09503	0.26969
C	-0.02042	2.59619	0.95597
C	-0.27487	4.08373	0.66741
C	-0.31177	4.37103	-0.84083
C	0.95732	3.86110	-1.53858
C	1.20302	2.37297	-1.24545
H	2.15211	2.62191	0.69604

```
H       0.02018    2.41687    2.05026
H      -0.86534    1.98691    0.56987
H      -1.23144    4.39446    1.13854
H       0.52886    4.69205    1.14462
H      -0.44686    5.45827   -1.02496
H      -1.19784    3.86657   -1.28117
H       0.88697    4.01628   -2.63648
H       1.83738    4.45151   -1.19220
H       2.13914    2.03854   -1.73994
H       0.37798    1.76056   -1.67290
C       3.12466   -0.82127   -1.45688
C       4.49915   -1.37816   -1.85705
C       5.60767   -0.32911   -1.67344
C       5.62968    0.21769   -0.23646
C       4.25798    0.78446    0.17299
C       3.15168   -0.26362   -0.02222
H       2.33068   -1.59381   -1.54295
H       2.84197   -0.00711   -2.16015
H       4.47126   -1.73913   -2.90750
H       4.72698   -2.26912   -1.22737
H       6.59811   -0.75688   -1.93931
H       5.43064    0.51379   -2.38090
H       6.41279    0.99863   -0.12695
H       5.90545   -0.60352    0.46421
H       4.28012    1.13145    1.22836
H       4.03325    1.67644   -0.45386
H       3.36984   -1.11815    0.66108
C       0.43088   -5.81284   -2.17346
C      -0.29932   -7.07773   -2.67298
H       0.28184   -7.58302   -3.47324
H      -0.45329   -7.80960   -1.85109
H      -1.29624   -6.82077   -3.08909
C       1.80474   -6.21590   -1.60052
H       1.69842   -6.89469   -0.72673
H       2.40975   -6.74517   -2.36661
H       2.39000   -5.32969   -1.27715
C       0.63162   -4.84815   -3.36173
H       1.16208   -5.36348   -4.19129
H      -0.33862   -4.47003   -3.74368
H       1.22091   -3.96177   -3.06203
C      -4.29378   -1.83717   -4.15904
C      -3.82210   -1.17925   -2.84018
C      -4.64721   -1.70936   -1.65957
C      -6.15104   -1.48845   -1.87883
C      -6.62757   -2.12232   -3.19544
C      -5.79734   -1.61612   -4.38538
H      -3.71457   -1.42919   -5.01436
H      -4.07025   -2.92546   -4.10417
H      -3.97715   -0.08463   -2.93195
H      -4.31292   -1.21424   -0.72549
H      -4.43785   -2.79581   -1.54489
H      -6.72800   -1.89605   -1.02036
H      -6.35771   -0.39373   -1.90314
H      -7.70823   -1.91908   -3.35949
H      -6.52227   -3.22964   -3.12443
H      -6.11919   -2.11488   -5.32551
H      -5.98747   -0.52741   -4.52734
C      -2.31174   -1.43401   -2.67726
O      -1.95683   -2.65630   -2.49218
C      -1.47674   -0.58070   -3.67387
O      -1.87675    0.45514   -4.19066
O      -0.30622   -1.18242   -3.94142
C       0.61695   -0.63792   -4.95955
C       1.08377    0.76201   -4.54446
H       1.86658    1.11895   -5.24448
H       1.51608    0.72750   -3.52652
H       0.24253    1.47878   -4.54616
C       1.77893   -1.63487   -4.93363
H       2.57528   -1.31298   -5.63453
H       1.43376   -2.64536   -5.22695
H       2.20411   -1.70017   -3.91403
C      -0.07564   -0.64012   -6.32924
H      -0.91529    0.07860   -6.35104
H      -0.46262   -1.65338   -6.56019
H       0.65583   -0.36128   -7.11522
```

Listing S4: Absolute electronic energies of the calculated structures of the aldehyde model reaction at the DF-BP86-D3(BJ)-PCM/TZVPP//DF-BP86-D3(BJ)/SVP level of theory in hartree (a.u.).

```
Ald-L2-AC-R          -3917.24649193
Ald-L2-TS-R          -3917.23589865
Ald-L2-PC-R          -3917.27439130
Ald-L2-AC-S          -3917.23928881
Ald-L2-TS-S          -3917.22640010
Ald-L2-PC-S          -3917.26676479
```

Listing S5: Cartesian coordinates of the calculated structures for the aldehyde model reaction optimized at the DF-BP86-D3(BJ)/SVP level of theory in angstrom.

```
Alde-L2-AC-R
C     5.42217   -1.72003   -1.22776
C     5.53009   -0.90755   -0.08400
C     4.38361   -0.31401    0.46895
C     3.12018   -0.52742   -0.12439
C     3.01207   -1.36164   -1.25945
C     4.16345   -1.94870   -1.81102
P     1.56752    0.20256    0.54118
C     1.61325    1.96847    0.04600
C     2.73984    2.60136   -0.51868
C     2.65403    3.93931   -0.94211
C     1.45061    4.65165   -0.79876
C     0.32445    4.02089   -0.23764
C     0.39729    2.68180    0.17350
Cu   -0.40316   -0.76825    0.36955
N    -0.09769   -2.27580    1.91814
C    -0.53761   -3.52801    1.24976
C    -2.07575   -3.39636    1.22406
C    -2.42866   -2.45204    2.40345
C    -1.07193   -2.05914    3.01088
C     0.12404   -3.70341   -0.13318
O    -0.02906   -2.52534   -0.92412
C     1.30079   -2.29815    2.38778
C     1.71894   -1.00750    3.07322
C     1.88786    0.21177    2.35590
C     2.23184    1.38922    3.04879
C     2.42758    1.37277    4.44016
C     2.28166    0.17135    5.15026
```

C	1.92910	-1.00390	4.46444
C	-2.04319	0.14450	0.28114
C	-3.07585	0.85574	0.26571
Si	-4.46032	2.04319	0.21483
C	-5.39340	2.03384	1.86717
C	-3.76995	3.78641	-0.10261
C	-5.67356	1.59217	-1.17682
C	-2.58434	-0.85383	-2.39939
O	-2.31953	-2.02219	-2.09973
C	-1.65634	0.09480	-3.12859
H	-3.63409	-0.48020	-2.26378
H	-0.89812	-2.50256	-1.43501
H	1.22265	-3.80171	0.02331
C	-0.37795	-4.94432	-0.86866
H	-0.23824	-4.40517	1.88303
H	-2.39966	-2.93993	0.26640
H	-2.55670	-4.39105	1.30526
H	-3.07430	-2.93897	3.16086
H	-2.94884	-1.55183	2.02330
H	-1.02784	-1.00773	3.35625
H	-0.80984	-2.71855	3.87844
H	1.95515	-2.49305	1.51282
H	1.44690	-3.14707	3.10351
H	2.34571	2.32916	2.48653
H	2.69725	2.30165	4.96692
H	1.80195	-1.94616	5.02243
H	2.43427	0.14653	6.24045
H	-4.57970	4.54403	-0.16938
H	-3.08686	4.08287	0.72107
H	-3.19261	3.81095	-1.05015
H	-6.05768	0.55917	-1.04311
H	-6.54286	2.28384	-1.18870
H	-5.18352	1.64704	-2.17142
H	-5.81715	1.02840	2.07150
H	-4.70869	2.28776	2.70309
H	-6.22741	2.76783	1.86610
H	3.68065	2.04305	-0.64145
H	-0.49841	2.16291	0.55726
H	3.53321	4.42586	-1.39335
H	-0.62856	4.56454	-0.14305
H	1.38591	5.69748	-1.13804
H	4.46420	0.31347	1.37091
H	2.01549	-1.57094	-1.67877
H	6.51412	-0.73746	0.38114
H	4.07459	-2.59793	-2.69643
H	6.32284	-2.18500	-1.65894
H	-0.22881	-5.85863	-0.25804
H	-1.45964	-4.85524	-1.09864
H	0.16868	-5.07026	-1.82433
C	-0.16560	-0.24067	-2.99635
H	-1.96272	-0.08444	-4.19644
C	0.71154	0.67593	-3.85915
H	0.09873	-0.11297	-1.92014
H	0.01380	-1.30769	-3.23863
C	0.43371	2.15900	-3.57179
H	1.78306	0.44277	-3.67751
H	0.52084	0.46276	-4.93713
C	-1.05918	2.49134	-3.72223
H	0.74866	2.38778	-2.53217
H	1.04644	2.80563	-4.23581
C	-1.92305	1.58364	-2.83505
H	-1.24267	3.55529	-3.45901
H	-1.36175	2.37366	-4.78936
H	-1.69226	1.76272	-1.76539
H	-3.00290	1.81262	-2.96474

Ald-L2-TS-R

C	5.48528	-1.63620	-0.92827
C	5.37078	-0.63193	0.05091
C	4.12598	-0.03182	0.30079
C	2.98994	-0.43381	-0.43427
C	3.10199	-1.45617	-1.40289
C	4.35177	-2.04872	-1.65079
P	1.32124	0.28239	-0.16222
C	1.37167	1.96191	-0.88936
C	2.53429	2.56122	-1.41468
C	2.45573	3.82527	-2.02593
C	1.22475	4.49867	-2.10764
C	0.06187	3.89855	-1.58935
C	0.12915	2.63023	-0.99621
Cu	-0.41888	-0.96235	-0.59342
N	-0.49818	-2.15381	1.22575
C	-0.72081	-3.52989	0.70328
C	-2.23618	-3.56555	0.42245
C	-2.86403	-2.53414	1.39213
C	-1.66983	-1.84797	2.07684
C	0.16536	-3.83545	-0.52745
O	0.05503	-2.81874	-1.51857
C	0.78227	-1.97593	1.94127
C	0.97412	-0.57015	2.49199
C	1.25902	0.54938	1.65773
C	1.40021	1.83059	2.22613
C	1.26563	2.02140	3.61108
C	0.99178	0.92385	4.44063
C	0.85297	-0.35678	3.87790
C	-2.15604	-0.25001	-1.04208
C	-2.85449	0.53790	-0.35193
Si	-3.58366	1.73375	0.82969
C	-5.01669	0.93161	1.77588
C	-2.22949	2.25527	2.05562
C	-4.19841	3.25851	-0.11520
C	-2.40548	-1.32000	-2.89072
O	-2.12176	-2.54855	-2.72567
C	-1.58914	-0.43341	-3.84068
H	-3.48500	-1.02849	-2.83908
H	-0.83042	-2.85442	-2.08831
H	1.22973	-3.80613	-0.19525
C	-0.12270	-5.21754	-1.11383
H	-0.45770	-4.27011	1.50435
H	-2.42891	-3.26824	-0.62970
H	-2.63773	-4.58833	0.56169
H	-3.52510	-3.00694	2.14536
H	-3.45735	-1.79130	0.82752
H	-1.78862	-0.74994	2.16555
H	-1.50134	-2.25941	3.10367
H	1.60775	-2.23112	1.24468
H	0.83906	-2.69921	2.79403
H	1.60848	2.68956	1.56964
H	1.37356	3.03030	4.03887
H	0.63527	-1.21895	4.52938
H	0.88200	1.06071	5.52759
H	-2.63808	2.94176	2.82745
H	-1.78943	1.37827	2.57391
H	-1.39957	2.77681	1.53714
H	-4.98127	2.97931	-0.85056
H	-4.62559	4.01681	0.57491
H	-3.36423	3.73125	-0.67443
H	-5.80873	0.58870	1.07822
H	-4.66370	0.04798	2.34713
H	-5.47286	1.64593	2.49391
H	3.49913	2.03452	-1.35624
H	-0.78479	2.12320	-0.64674
H	3.36475	4.28514	-2.44460
H	-0.91013	4.40971	-1.67133
H	1.16810	5.48694	-2.59006
H	4.02920	0.74598	1.07518

H	2.19749	-1.79995	-1.93213
H	6.25667	-0.31767	0.62529
H	4.43770	-2.84541	-2.40642
H	6.46267	-2.10625	-1.12085
H	-0.00997	-6.01224	-0.34724
H	-1.15513	-5.26478	-1.51613
H	0.57889	-5.43012	-1.94460
C	-0.07499	-0.66192	-3.75677
H	-1.92898	-0.80582	-4.84269
C	0.70739	0.16374	-4.78592
H	0.24271	-0.36462	-2.72755
H	0.15447	-1.74196	-3.85379
C	0.35802	1.65627	-4.69203
H	1.79861	0.01094	-4.63756
H	0.47531	-0.20671	-5.81172
C	-1.15791	1.87927	-4.81846
H	0.70002	2.04601	-3.70839
H	0.90426	2.23574	-5.46752
C	-1.92889	1.06371	-3.76924
H	-1.39770	2.95924	-4.70838
H	-1.48920	1.58731	-5.84249
H	-1.66337	1.42662	-2.75513
H	-3.02576	1.21403	-3.87453

Ald-L2-PC-R

C	5.17617	-1.70565	-0.60845
C	5.16106	-0.74432	0.41943
C	3.96376	-0.08983	0.75140
C	2.77767	-0.39670	0.04955
C	2.78586	-1.37838	-0.96642
C	3.99043	-2.02376	-1.29382
P	1.17886	0.43252	0.39942
C	1.33055	2.08254	-0.38809
C	2.53992	2.60990	-0.88676
C	2.55140	3.86297	-1.52359
C	1.36307	4.60120	-1.66246
C	0.15180	4.07245	-1.18065
C	0.13394	2.81438	-0.56247
Cu	-0.68132	-0.79749	0.02059
N	-0.52689	-1.97852	1.87137
C	-0.62477	-3.34346	1.26209
C	-2.13849	-3.51536	1.01047
C	-2.84238	-2.51595	1.96476
C	-1.69952	-1.86578	2.75891
C	0.24290	-3.44276	-0.01751
O	-0.09925	-2.45153	-0.93815
C	0.75151	-1.74986	2.57039
C	0.94819	-0.33209	3.08119
C	1.24501	0.75425	2.20716
C	1.46222	2.04149	2.73578
C	1.37312	2.27414	4.11845
C	1.06529	1.21421	4.98442
C	0.86399	-0.07605	4.46218
C	-2.35123	-0.37185	-1.07067
C	-2.45238	0.33711	-0.02985
Si	-3.21099	1.54885	1.15407
C	-4.70297	0.74143	1.99345
C	-1.98362	2.12961	2.46340
C	-3.78614	3.02245	0.10994
C	-2.48224	-0.98949	-2.42178
O	-2.21185	-2.36663	-2.41959
C	-1.63066	-0.18804	-3.45826
H	-3.55326	-0.85392	-2.71546
H	-1.34911	-2.51053	-1.87931
H	1.31197	-3.30451	0.31254
C	0.14983	-4.83984	-0.64567
H	-0.26637	-4.09288	2.01483
H	-2.36556	-3.26566	-0.04630
H	-2.44925	-4.56348	1.18620
H	-3.57232	-3.00140	2.64245
H	-3.38713	-1.75118	1.37944
H	-1.88051	-0.80598	3.02809
H	-1.50552	-2.42469	3.71080
H	1.56864	-2.00975	1.86632
H	0.83457	-2.45602	3.43481
H	1.69166	2.87258	2.05074
H	1.54209	3.28706	4.51621
H	0.63507	-0.91281	5.14253
H	0.98591	1.38639	6.06904
H	-2.50164	2.78974	3.19087
H	-1.53638	1.28553	3.02625
H	-1.14784	2.70455	2.02001
H	-4.51218	2.69535	-0.66255
H	-4.27501	3.79045	0.74631
H	-2.93300	3.50190	-0.41206
H	-5.39732	0.30946	1.24401
H	-4.39335	-0.07077	2.68163
H	-5.25910	1.49815	2.58692
H	3.47227	2.03419	-0.78378
H	-0.81560	2.36479	-0.23946
H	3.49854	4.26476	-1.91669
H	-0.78810	4.63135	-1.31167
H	1.37743	5.58144	-2.16376
H	3.94409	0.65696	1.56145
H	1.82779	-1.66511	-1.43976
H	6.08694	-0.50582	0.96664
H	3.99742	-2.79343	-2.08172
H	6.11665	-2.21826	-0.86601
H	0.45533	-5.63776	0.06475
H	-0.88462	-5.04683	-0.98980
H	0.81368	-4.88772	-1.53195
C	-0.11982	-0.34626	-3.22836
H	-1.88156	-0.65619	-4.43892
C	0.71231	0.41771	-4.26509
H	0.11999	0.04394	-2.21178
H	0.14502	-1.42157	-3.20761
C	0.32360	1.90286	-4.30588
H	1.79382	0.30668	-4.03367
H	0.55703	-0.03537	-5.27193
C	-1.18517	2.07623	-4.54652
H	0.59304	2.37138	-3.33425
H	0.90440	2.44115	-5.08599
C	-2.01492	1.30156	-3.50929
H	-1.45895	3.15374	-4.52523
H	-1.43865	1.70921	-5.56797
H	-1.84060	1.74438	-2.50306
H	-3.10267	1.41334	-3.71681

Ald-L2-AC-S

C	6.03538	-1.98364	-0.33290
C	5.84107	-1.31359	0.88844
C	4.65961	-0.58775	1.11544
C	3.66015	-0.53278	0.11994
C	3.85377	-1.21960	-1.10039
C	5.04125	-1.93394	-1.32716
P	2.05572	0.34096	0.33894
C	2.41707	2.08129	-0.11829
C	3.72509	2.59248	-0.24992
C	3.91951	3.94265	-0.58864
C	2.81380	4.78757	-0.78976
C	1.50887	4.27633	-0.66357
C	1.30472	2.92620	-0.33906
Cu	0.09796	-0.55537	-0.25810
N	0.14388	-2.17068	1.28537
C	0.06037	-3.42769	0.49398
C	-1.45726	-3.63772	0.23502

C	-2.17352	-2.53309	1.04849
C	-1.11728	-2.06683	2.04976
C	0.94660	-3.33293	-0.76685
O	0.59573	-2.19261	-1.55997
C	1.34665	-2.11106	2.13563
C	1.50420	-0.78893	2.86081
C	1.86011	0.40558	2.17263
C	1.95703	1.61622	2.88654
C	1.71655	1.65625	4.27027
C	1.37948	0.47953	4.95578
C	1.27783	-0.73038	4.24789
C	-1.34010	0.64790	-0.19370
C	-2.14978	1.58416	0.00491
Si	-3.11315	3.07968	0.40797
C	-4.81450	2.61642	1.11470
C	-2.15544	4.08703	1.70353
C	-3.34882	4.14669	-1.14674
C	-2.11902	-1.06911	-2.64577
O	-1.75949	-2.25225	-2.71117
C	-3.51484	-0.62430	-2.34322
H	-1.37442	-0.24260	-2.79632
H	-0.23035	-2.37494	-2.11056
H	1.98839	-3.13148	-0.43066
C	0.93785	-4.61282	-1.59832
H	0.45491	-4.27465	1.11137
H	-1.70987	-3.56005	-0.84028
H	-1.75522	-4.65165	0.56862
H	-3.09643	-2.88887	1.54718
H	-2.43740	-1.67735	0.39546
H	-1.26722	-1.02503	2.39575
H	-1.07987	-2.74002	2.94565
H	2.23115	-2.29681	1.49172
H	1.31583	-2.93749	2.89066
H	2.22159	2.53938	2.34937
H	1.79581	2.61240	4.81096
H	1.00628	-1.65673	4.78042
H	1.18961	0.50058	6.04019
H	-2.69878	5.01330	1.98861
H	-1.99032	3.48182	2.61917
H	-1.15718	4.37607	1.31289
H	-3.93534	3.60707	-1.91860
H	-3.88256	5.09180	-0.90948
H	-2.36544	4.40676	-1.59140
H	-5.40500	2.02724	0.38195
H	-4.70091	1.99767	2.02921
H	-5.40251	3.52121	1.37887
H	4.59202	1.93330	-0.08741
H	0.28420	2.51249	-0.25883
H	4.94272	4.33713	-0.69386
H	0.63854	4.93106	-0.82974
H	2.96995	5.84598	-1.05218
H	4.50372	-0.06409	2.07201
H	3.05158	-1.20752	-1.85669
H	6.61668	-1.35526	1.66969
H	5.18709	-2.46538	-2.28093
H	6.96232	-2.55225	-0.50778
H	1.26768	-5.48165	-0.99235
H	-0.07914	-4.83069	-1.98580
H	1.62235	-4.51110	-2.46360
C	-4.55629	-1.74867	-2.32805
H	-3.39491	-0.20233	-1.31086
C	-5.93431	-1.20728	-1.91528
H	-4.61670	-2.20666	-3.34176
H	-4.22663	-2.55765	-1.64346
C	-6.36861	-0.02854	-2.80266
H	-6.69252	-2.01891	-1.94879
H	-5.88570	-0.86530	-0.85604
C	-5.31624	1.09208	-2.79918
H	-6.51493	-0.39110	-3.84659
H	-7.35240	0.36556	-2.46839
C	-3.93303	0.57537	-3.22123
H	-5.63159	1.92834	-3.45922
H	-5.23500	1.51332	-1.77221
H	-3.95707	0.26558	-4.29156
H	-3.17381	1.38102	-3.12681

Alde-L2-TS-S

C	5.50732	-1.94940	-0.93222
C	5.43236	-0.99007	0.09417
C	4.23800	-0.28377	0.31190
C	3.11270	-0.53093	-0.50414
C	3.18368	-1.51085	-1.51936
C	4.38253	-2.21111	-1.73462
P	1.49527	0.31204	-0.26520
C	1.70280	2.01415	-0.91461
C	2.95071	2.64234	-1.10454
C	3.00412	3.96323	-1.58192
C	1.81690	4.66129	-1.86614
C	0.57181	4.03055	-1.68910
C	0.51048	2.70714	-1.22579
Cu	-0.31215	-0.82677	-0.71779
N	-0.40965	-2.05672	1.07836
C	-0.69706	-3.40657	0.52162
C	-2.22581	-3.40141	0.30340
C	-2.78726	-2.31286	1.25291
C	-1.55648	-1.71497	1.94952
C	0.13645	-3.69459	-0.75226
O	-0.00496	-2.66146	-1.72611
C	0.88124	-1.96597	1.79113
C	1.13635	-0.58726	2.37741
C	1.43869	0.54209	1.56385
C	1.61644	1.80579	2.16040
C	1.50956	1.96601	3.55192
C	1.22710	0.85565	4.36112
C	1.04506	-0.40660	3.76999
C	-2.01604	-0.02564	-1.12462
C	-2.62234	0.86133	-0.46812
Si	-3.29377	2.14916	0.64707
C	-4.73021	1.43551	1.65724
C	-1.89259	2.68932	1.80677
C	-3.89582	3.62514	-0.37962
C	-2.50404	-1.15414	-2.92218
O	-2.28978	-2.39039	-2.72939
C	-3.94794	-0.67266	-3.08979
H	-1.73584	-0.54605	-3.46626
H	-0.93212	-2.68551	-2.22053
H	1.21310	-3.67822	-0.46199
C	-0.18618	-5.06514	-1.34805
H	-0.41971	-4.17915	1.28580
H	-2.46096	-3.14140	-0.74901
H	-2.64886	-4.40552	0.50320
H	-3.49923	-2.72035	1.99778
H	-3.30750	-1.52923	0.67076
H	-1.61463	-0.61499	2.07531
H	-1.40391	-2.16903	2.96118
H	1.69024	-2.24105	1.08303
H	0.90466	-2.71572	2.62236
H	1.83633	2.67612	1.52364
H	1.64706	2.96225	4.00043
H	0.81483	-1.27905	4.40322
H	1.14178	0.96863	5.45304
H	-2.24103	3.45526	2.53192
H	-1.49438	1.82594	2.37902
H	-1.04808	3.11696	1.22833
H	-4.69933	3.31661	-1.08051
H	-4.29555	4.43272	0.26976

H	-3.06426	4.04612	-0.98231
H	-5.54591	1.08840	0.98935
H	-4.39124	0.56556	2.25733
H	-5.15104	2.19229	2.35302
H	3.88130	2.09677	-0.88410
H	-0.45763	2.18937	-1.11423
H	3.98051	4.45018	-1.73343
H	-0.36014	4.56745	-1.92679
H	1.86317	5.69703	-2.23809
H	4.17021	0.45254	1.12872
H	2.28125	-1.74089	-2.11082
H	6.30873	-0.79523	0.73254
H	4.43437	-2.97506	-2.52637
H	6.44420	-2.50428	-1.09847
H	-0.03679	-5.87350	-0.60237
H	-1.23828	-5.10360	-1.69708
H	0.47102	-5.26437	-2.21750
C	-4.65917	-1.56164	-4.13981
H	-4.46154	-0.83241	-2.11628
C	-6.12932	-1.14709	-4.30671
H	-4.13101	-1.46568	-5.11712
H	-4.56876	-2.62365	-3.83379
C	-6.26559	0.34181	-4.66619
H	-6.62024	-1.77948	-5.07814
H	-6.66917	-1.34249	-3.35149
C	-5.53005	1.23120	-3.65012
H	-5.82937	0.51198	-5.67771
H	-7.33727	0.62922	-4.73583
C	-4.06336	0.80694	-3.48492
H	-5.59162	2.29919	-3.95236
H	-6.03879	1.15762	-2.66075
H	-3.52396	0.96323	-4.44844
H	-3.55599	1.43554	-2.72601

Ald-L2-PC-S

C	5.71499	-1.96682	-0.40379
C	5.68307	-1.13657	0.73160
C	4.54202	-0.36528	1.00622
C	3.42972	-0.42011	0.13815
C	3.45046	-1.27345	-0.98761
C	4.59837	-2.03813	-1.25528
P	1.90693	0.57304	0.41280
C	2.33198	2.24475	-0.22189
C	3.62555	2.80331	-0.14263
C	3.86447	4.09646	-0.63541
C	2.81836	4.83735	-1.21537
C	1.53335	4.27668	-1.31541
C	1.29185	2.98220	-0.82722
Cu	0.00158	-0.39254	-0.31484
N	-0.09877	-1.73238	1.54402
C	-0.26684	-3.03609	0.83737
C	-1.77938	-3.10223	0.52389
C	-2.45507	-2.05690	1.44915
C	-1.32135	-1.53204	2.34102
C	0.66522	-3.11707	-0.40237
O	0.46726	-2.05052	-1.29018
C	1.12490	-1.69609	2.35990
C	1.41748	-0.35334	3.00343
C	1.86930	0.76137	2.24173
C	2.17215	1.97541	2.88763
C	2.02115	2.10561	4.27829
C	1.56195	1.01671	5.03415
C	1.27082	-0.20107	4.39456
C	-1.70426	0.01933	-1.30246
C	-1.63409	0.88877	-0.38188
Si	-2.26771	2.22611	0.73858
C	-3.89100	1.60269	1.48931
C	-1.03471	2.63041	2.10217
C	-2.58431	3.76944	-0.31387
C	-2.19523	-0.71713	-2.50468
O	-1.73090	-2.03510	-2.59638
C	-3.74764	-0.70515	-2.52969
H	-1.84743	-0.10073	-3.38252
H	-0.79872	-2.09290	-2.14629
H	1.71728	-3.08156	-0.00348
C	0.50566	-4.46147	-1.12443
H	0.01354	-3.86107	1.54348
H	-1.95612	-2.85104	-0.54169
H	-2.16438	-4.12644	0.69541
H	-3.27628	-2.48308	2.05892
H	-2.88154	-1.23002	0.84899
H	-1.42722	-0.46348	2.62151
H	-1.25668	-2.12165	3.29267
H	1.97387	-1.98671	1.70714
H	1.06031	-2.47168	3.16560
H	2.51789	2.83414	2.29220
H	2.25842	3.06314	4.76766
H	0.92073	-1.06236	4.98685
H	1.43269	1.11003	6.12366
H	-1.46250	3.38787	2.79228
H	-0.76520	1.73533	2.69783
H	-0.09775	3.04406	1.68013
H	-3.27989	3.55101	-1.14956
H	-3.02889	4.57684	0.30605
H	-1.63813	4.15430	-0.74653
H	-4.61459	1.32079	0.69723
H	-3.71663	0.70969	2.12375
H	-4.35623	2.38883	2.12128
H	4.44953	2.22022	0.29698
H	0.29753	2.51780	-0.92592
H	4.87603	4.52757	-0.57132
H	0.71669	4.84452	-1.78851
H	3.01029	5.85025	-1.60295
H	4.50566	0.26975	1.90605
H	2.53522	-1.37977	-1.59953
H	6.55002	-1.09380	1.40990
H	4.61197	-2.70953	-2.12836
H	6.61006	-2.57301	-0.61565
H	0.69720	-5.32060	-0.44655
H	-0.51651	-4.56124	-1.54441
H	1.22255	-4.51429	-1.96804
C	-4.26957	-1.52936	-3.72036
H	-4.07883	-1.20827	-1.59042
C	-5.80520	-1.56326	-3.75204
H	-3.88805	-1.07062	-4.66287
H	-3.83993	-2.54878	-3.67056
C	-6.40295	-0.14700	-3.76553
H	-6.15798	-2.14251	-4.63257
H	-6.17588	-2.10729	-2.85270
C	-5.87544	0.68896	-2.58818
H	-6.12528	0.35631	-4.72044
H	-7.51355	-0.18919	-3.74564
C	-4.33920	0.71372	-2.55454
H	-6.27548	1.72506	-2.63447
H	-6.25035	0.25217	-1.63359
H	-3.95690	1.24664	-3.45656
H	-3.98477	1.29219	-1.67697

15 HPLC Charts

15.1 3aa

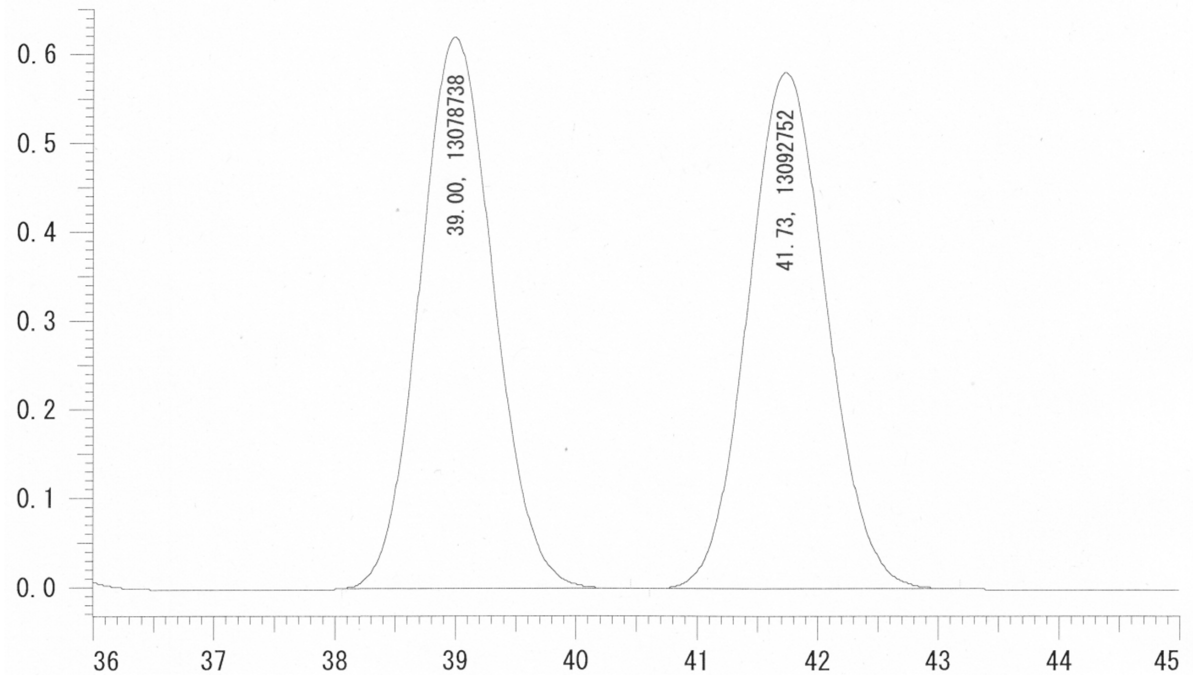

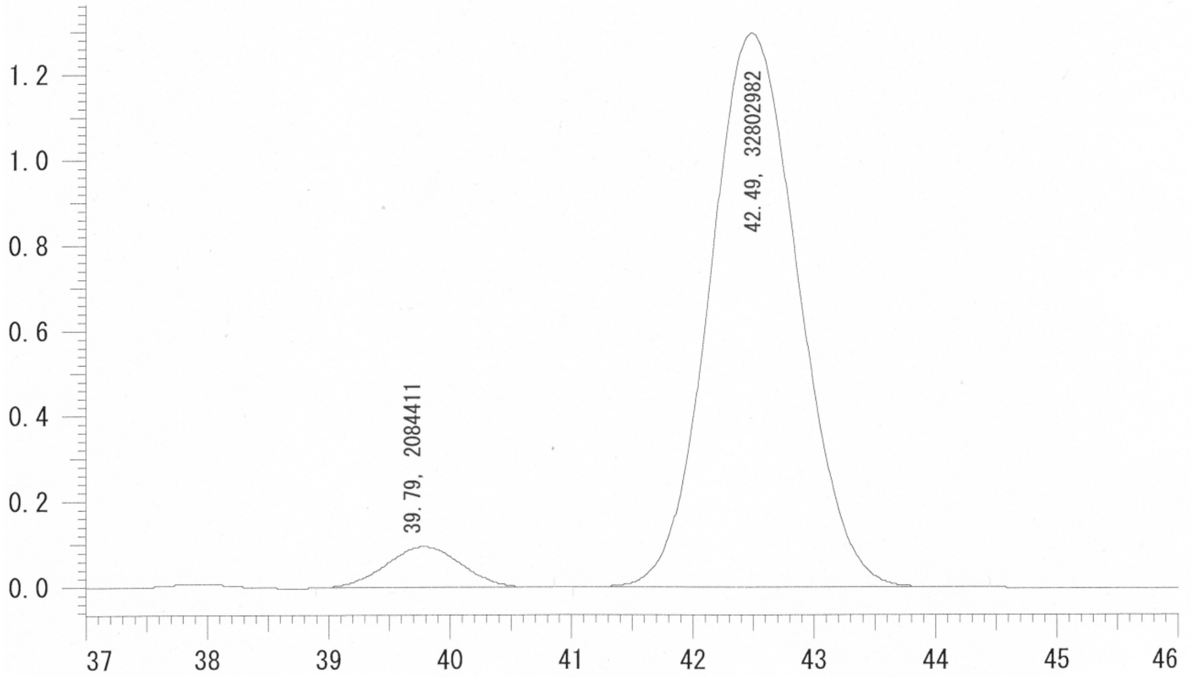

Racemic				Chiral			
No.	RT (min)	area	area (%)	No.	RT (min)	area	area (%)
1	39.00	13119319	50.001	1	39.79	2105987	6.026
2	41.73	13118696	49.999	2	42.49	32844963	93.974

15.2 3ba

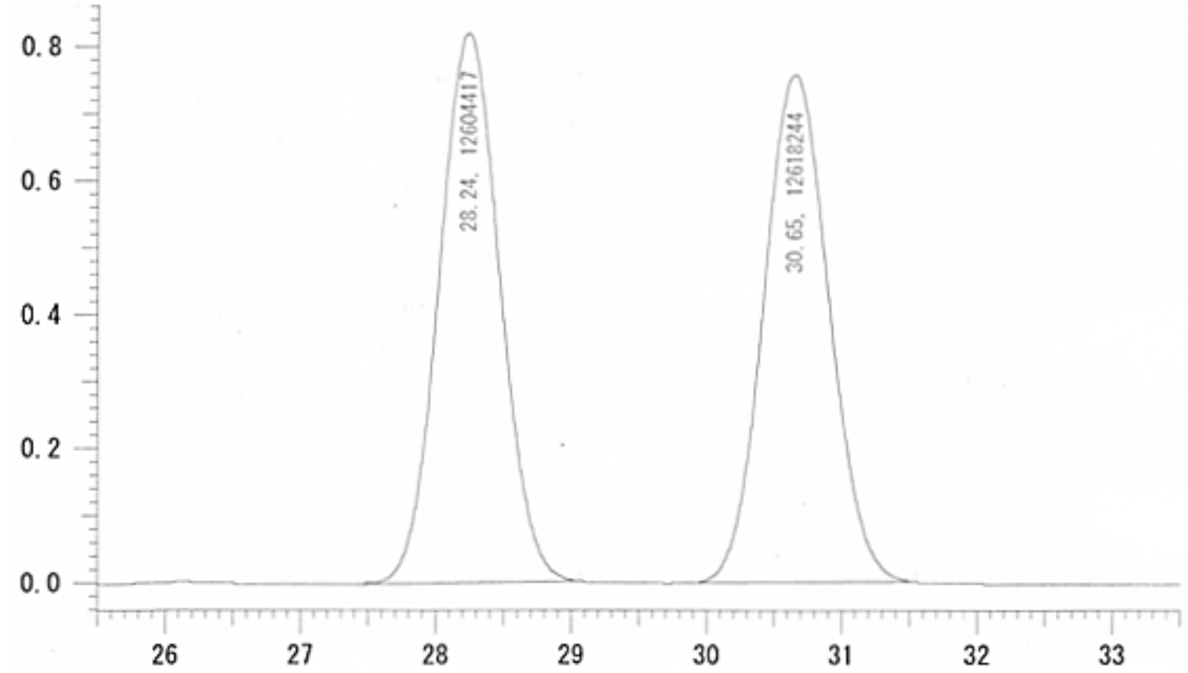

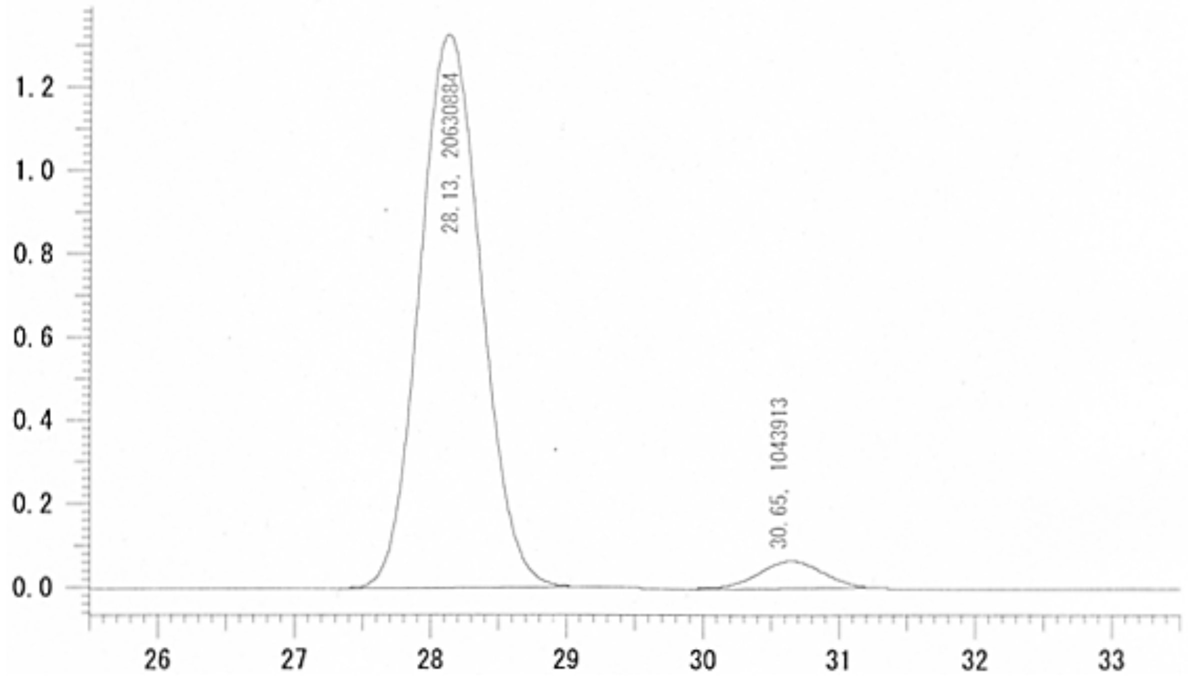

Racemic				chiral			
No.	RT (min)	area	area (%)	No.	RT (min)	area	area (%)
1	28.24	12692579	49.966	1	28.13	20755430	94.981
2	30.65	12709854	50.034	2	30.65	1096832	5.019

15.3 3ca

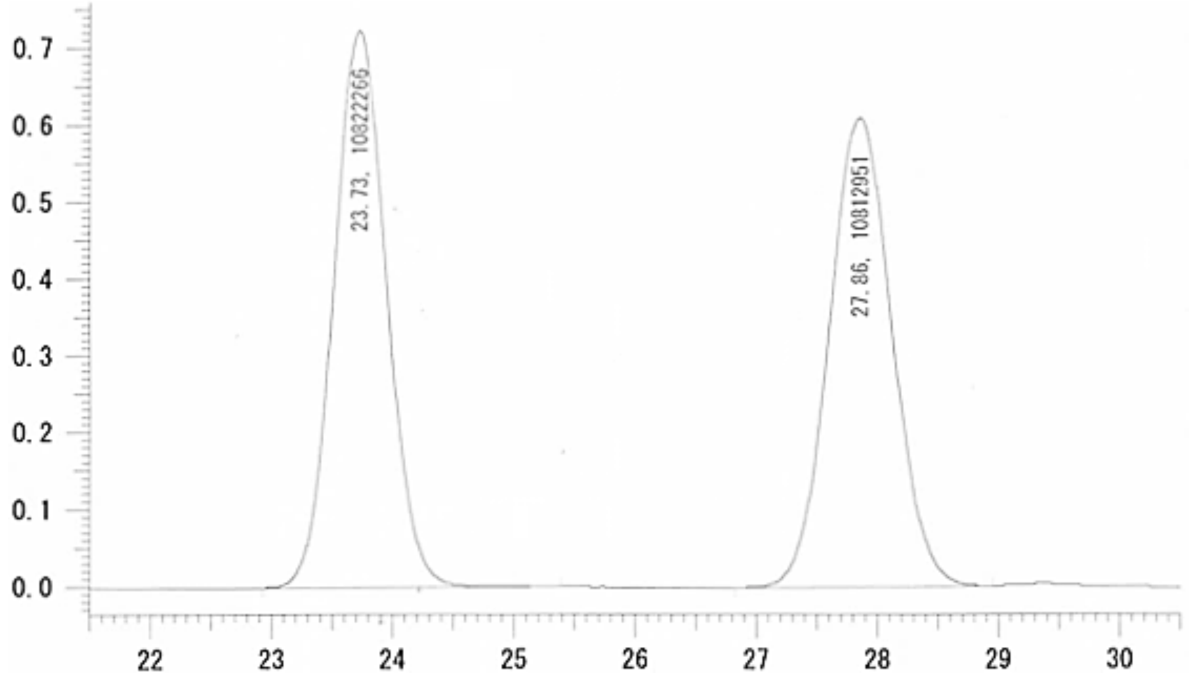

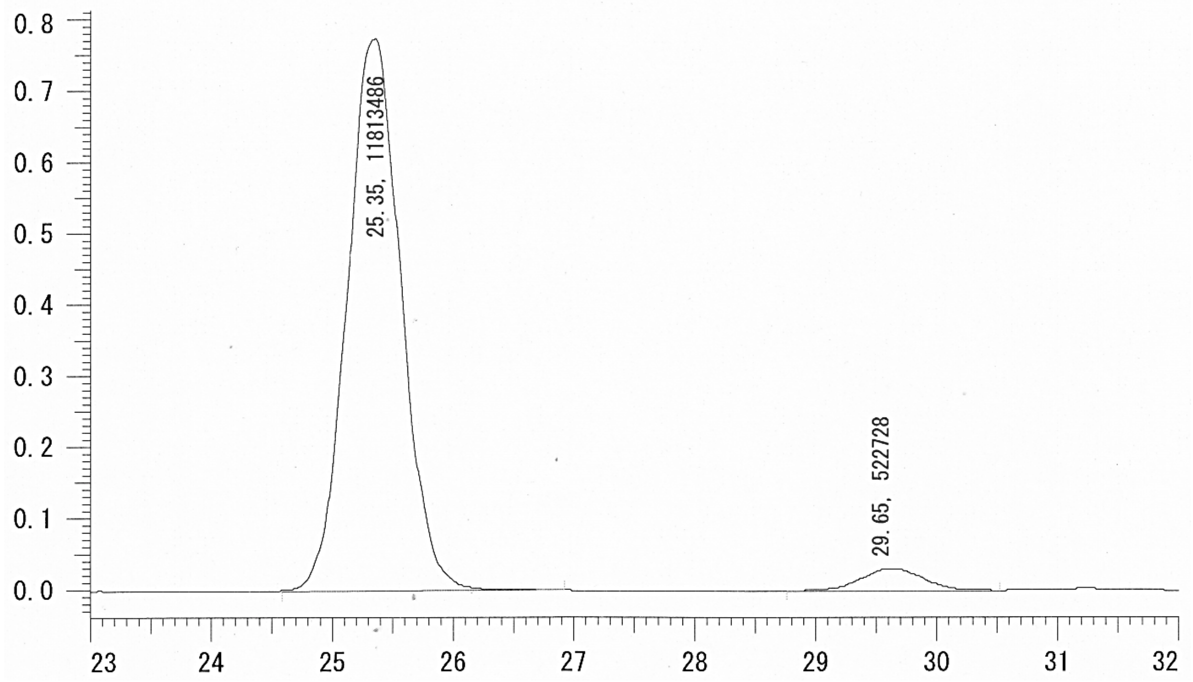

racemic				chiral			
No.	RT (min)	area	area (%)	No.	RT (min)	area	area (%)
1	23.73	10822266	50.022	1	25.35	11813486	95.763
2	29.35	10812951	49.978	2	29.65	522728	4.237

15.4 3da

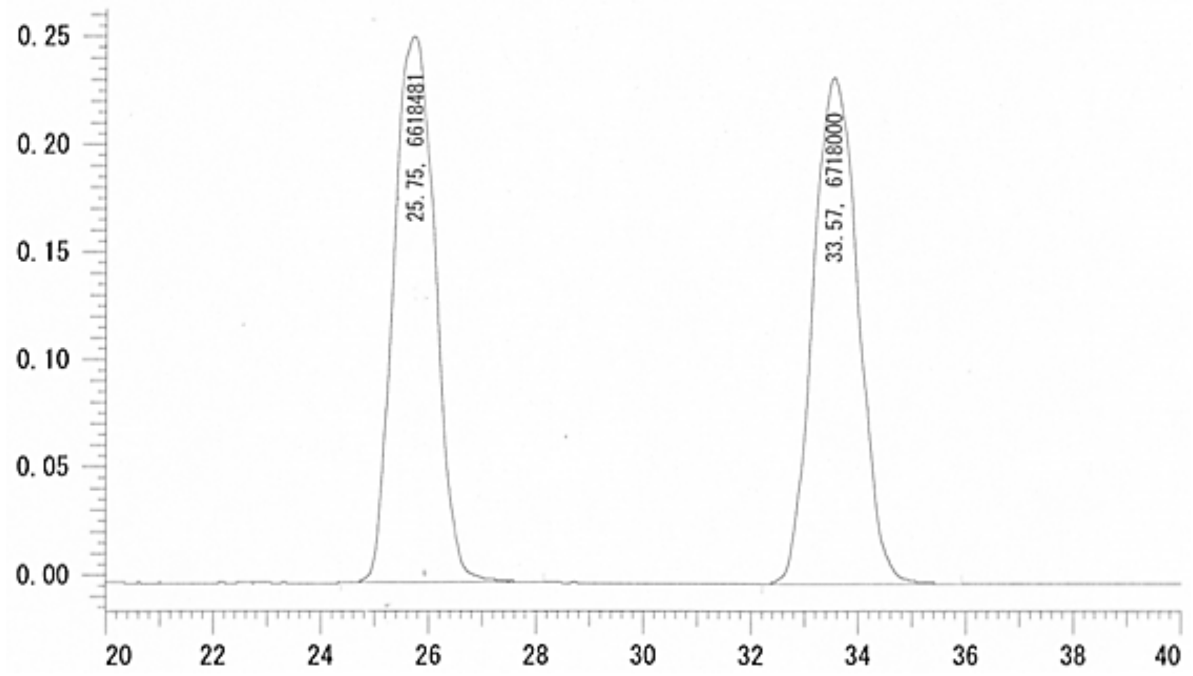

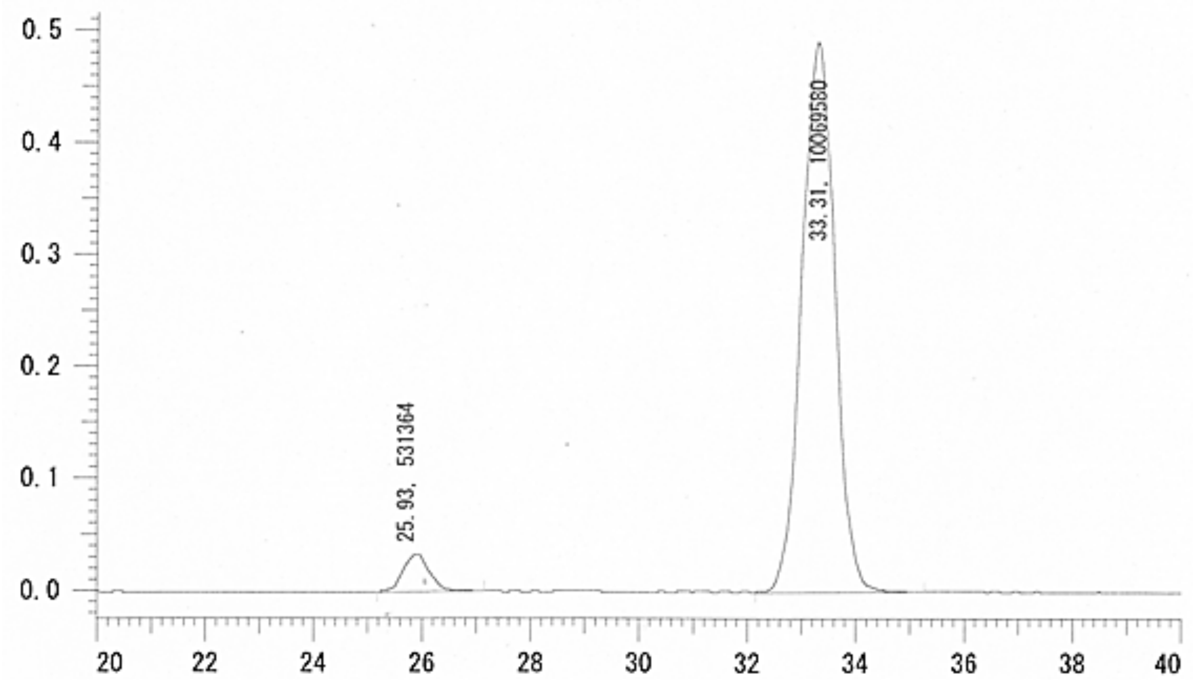

racemic				Chiral			
No.	RT (min)	area	area (%)	No.	RT (min)	area	area (%)
1	25.75	6605507	49.578	1	25.93	530572	5.005
2	33.57	6718000	50.422	2	33.31	10069580	94.995

15.5 3ea

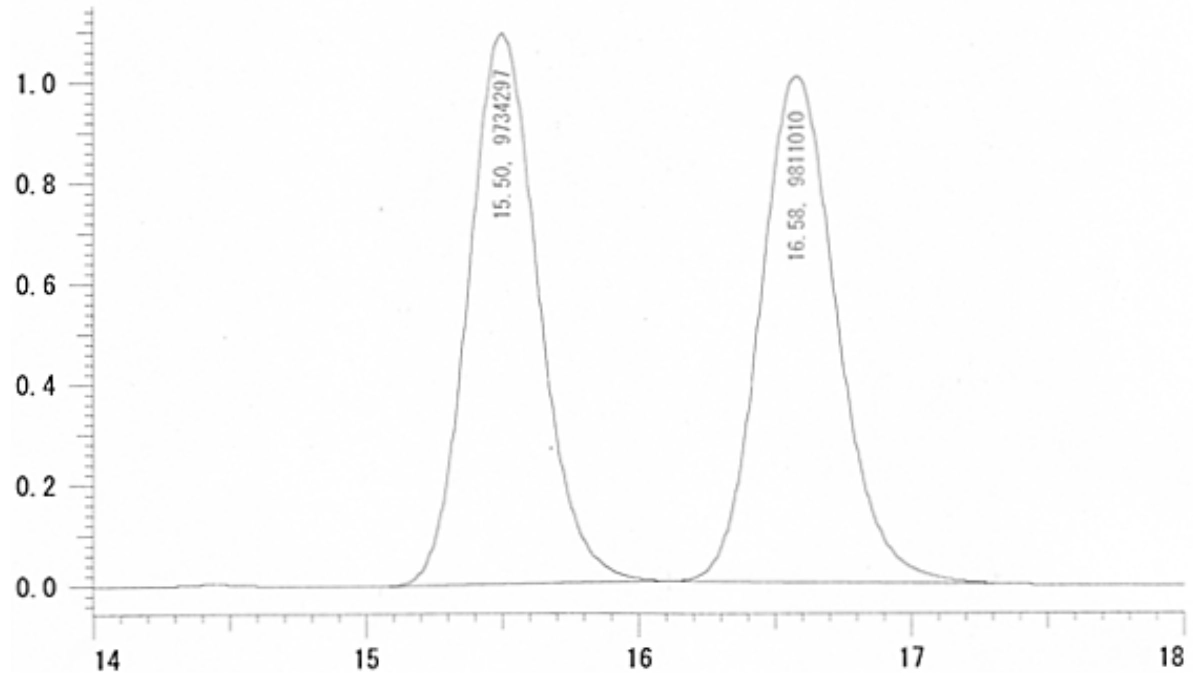

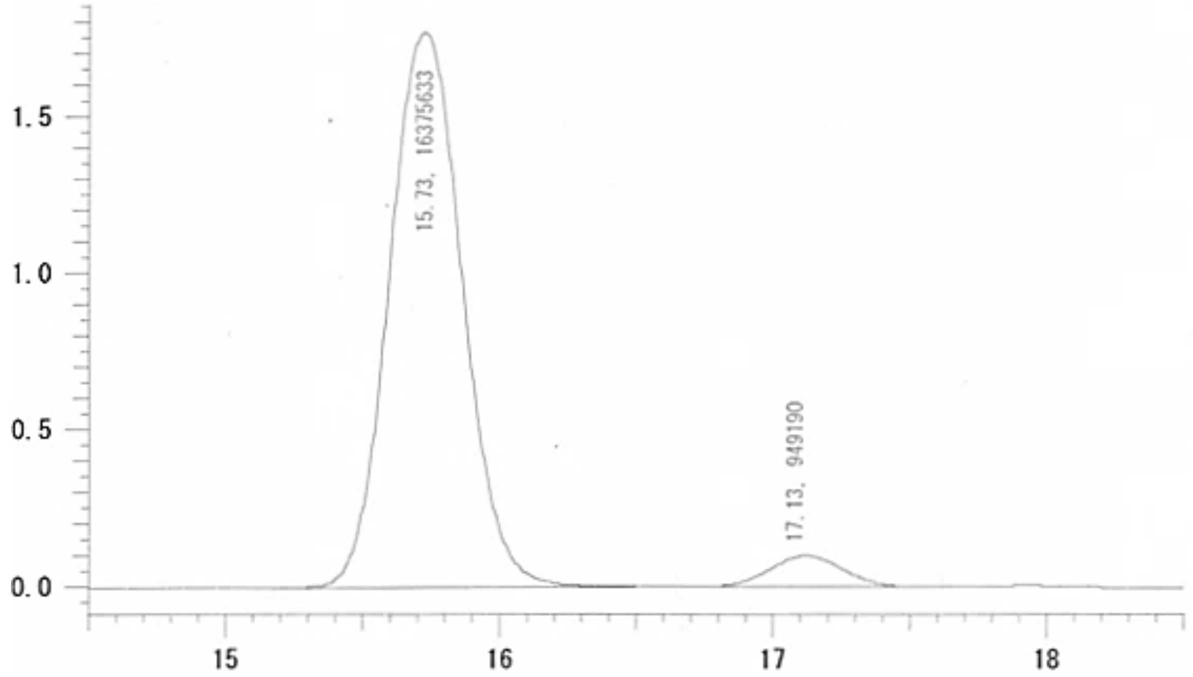

racemic				chiral			
No.	RT (min)	area	area (%)	No.	RT (min)	area	area (%)
1	15.50	9734297	49.823	1	15.73	16375633	94.521
2	16.58	9803473	50.177	2	17.13	949190	5.479

15.6 3fa

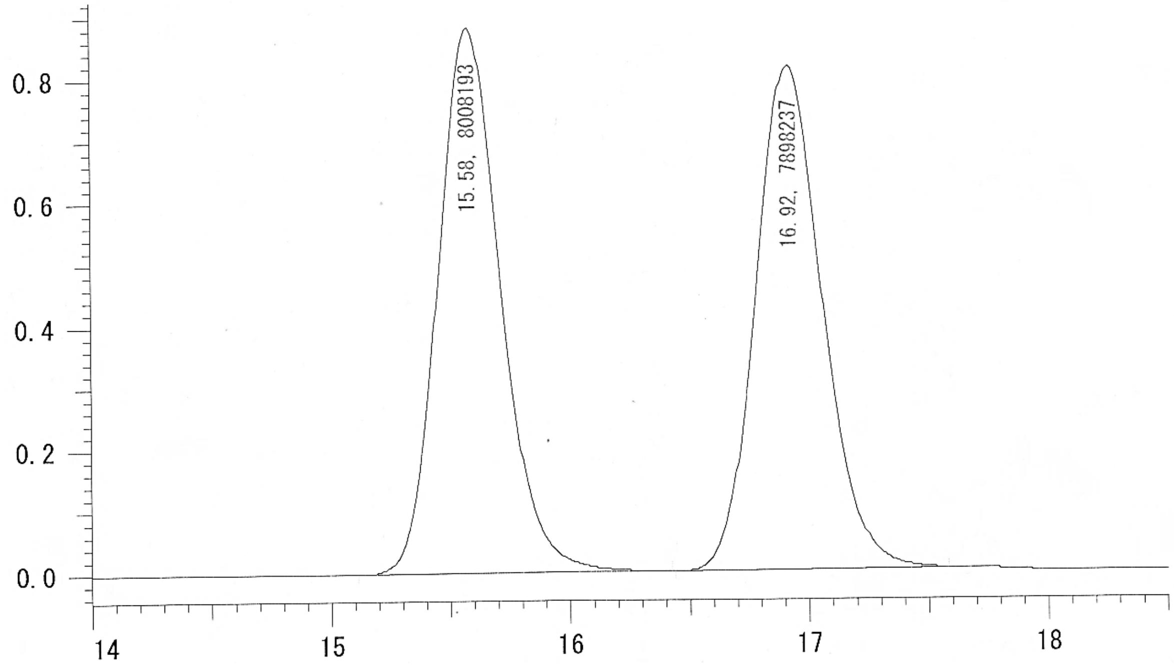

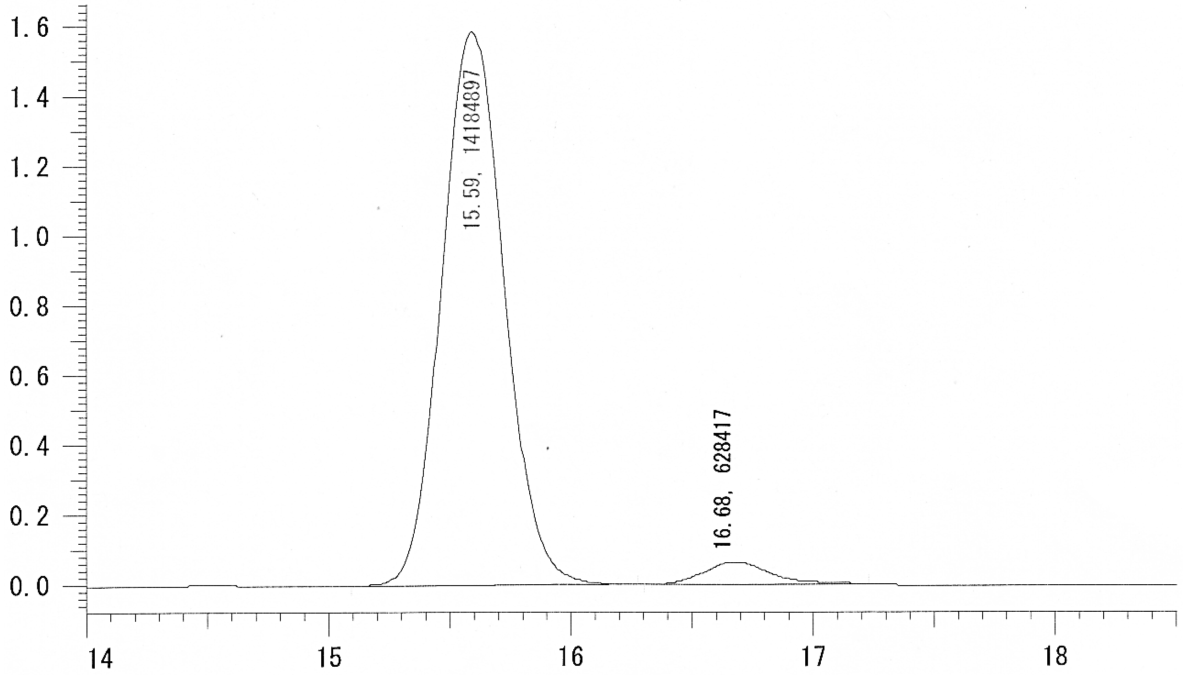

racemic				chiral			
No.	RT (min)	area	area (%)	No.	RT (min)	area	area (%)
1	15.58	8006937	49.966	1	15.59	14184897	95.758
2	16.92	7986196	50.034	2	16.68	628417	4.242

15.7 3ga

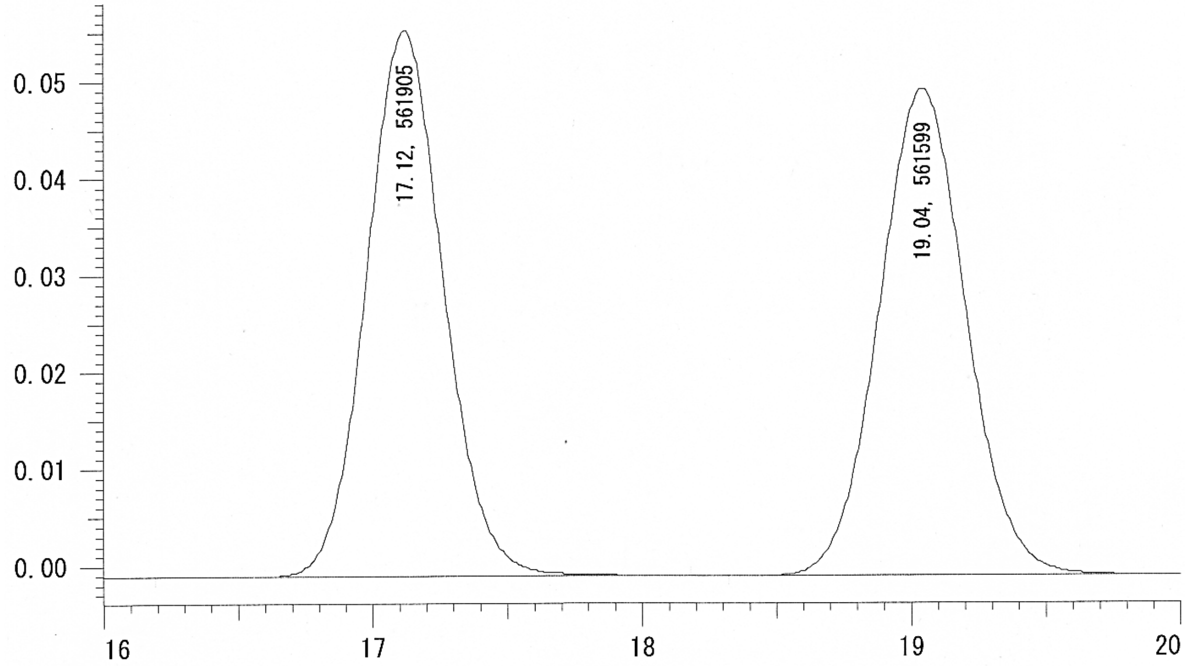

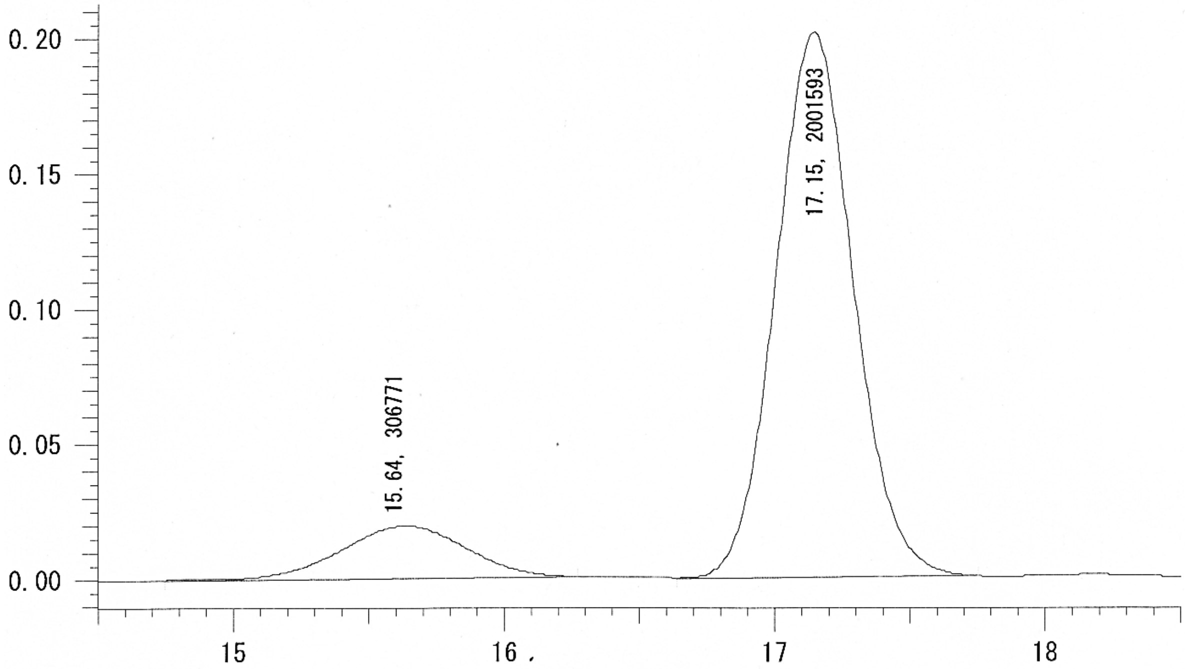

racemic				chiral			
No.	RT (min)	area	area (%)	No.	RT (min)	area	area (%)
1	17.12	560897	49.962	1	17.15	2001593	95.763
2	19.04	561750	50.038	2	19.06	88551	4.237

15.8 3ha

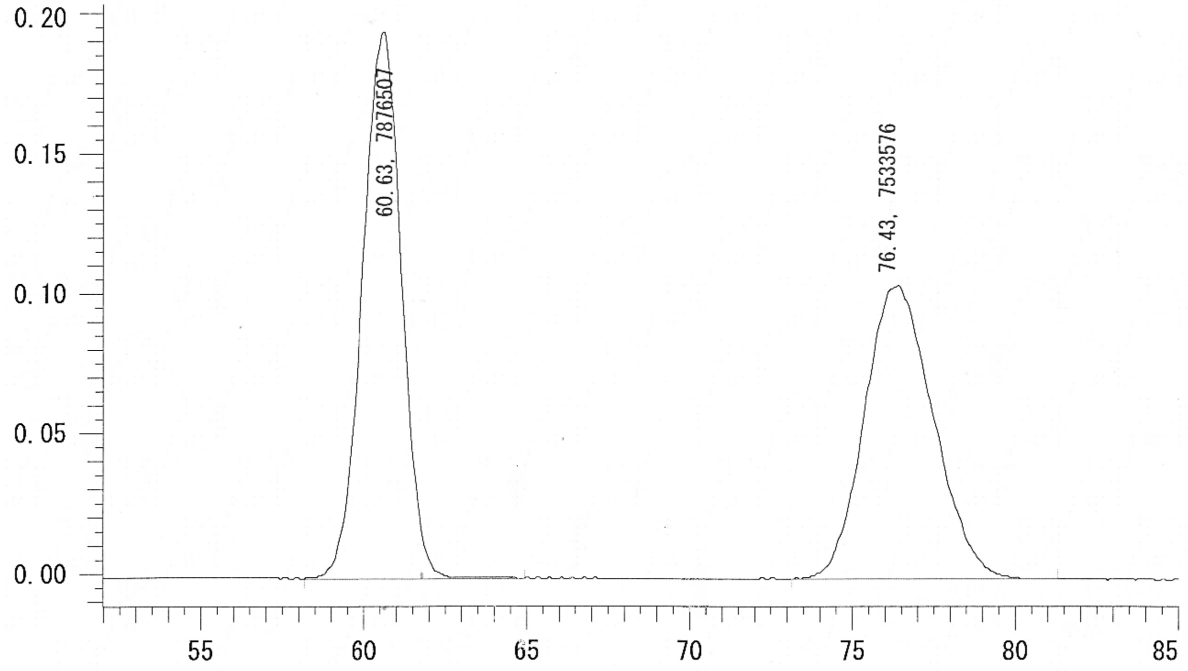

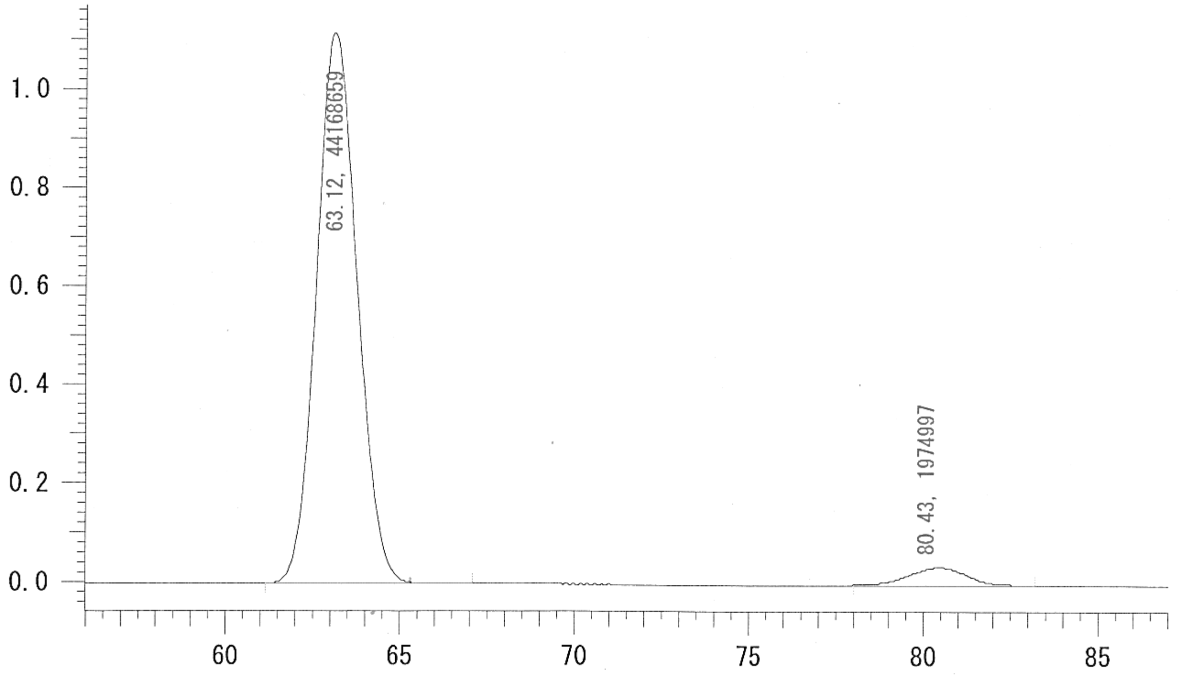

racemic				Chiral			
No.	RT (min)	area	area (%)	No.	RT (min)	area	area (%)
1	60.63	7805336	50.857	1	63.12	44168659	95.720
2	76.43	7542273	49.143	2	80.43	1974997	4.280

15.9 3ia

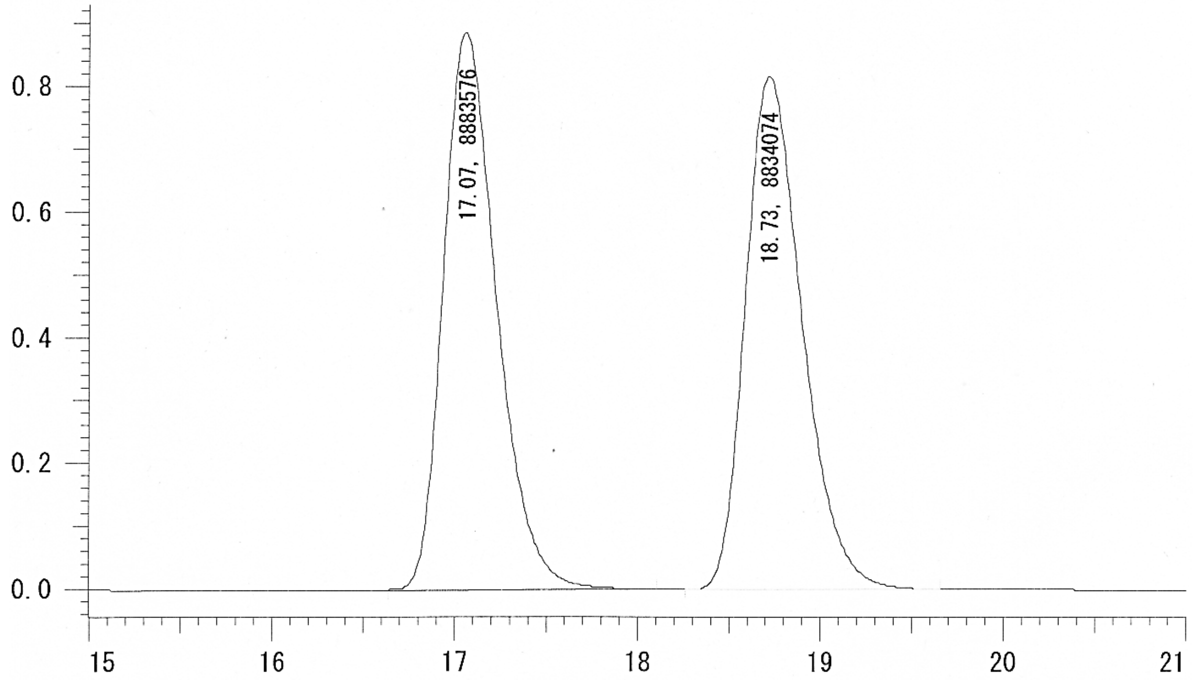

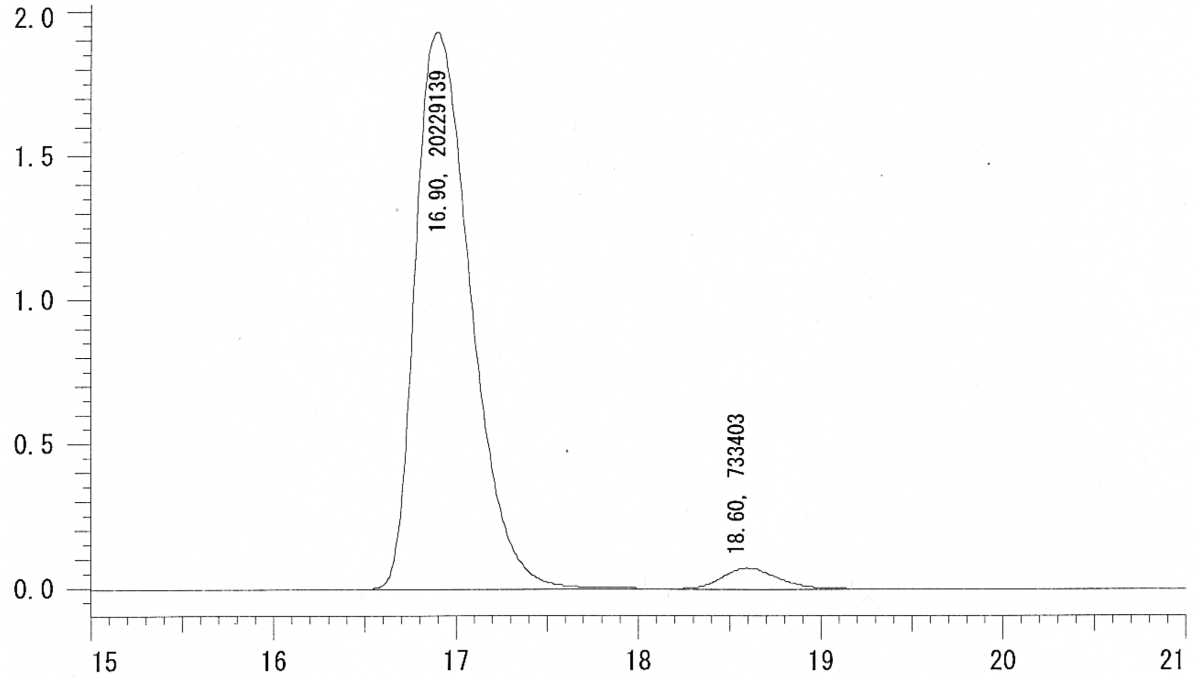

racemic				chiral			
No.	RT (min)	area	area (%)	No.	RT (min)	area	area (%)
1	17.07	8893342	50.110	1	16.90	20238195	96.447
2	18.73	8854136	49.890	2	18.60	745473	3.553

15.10 3ja

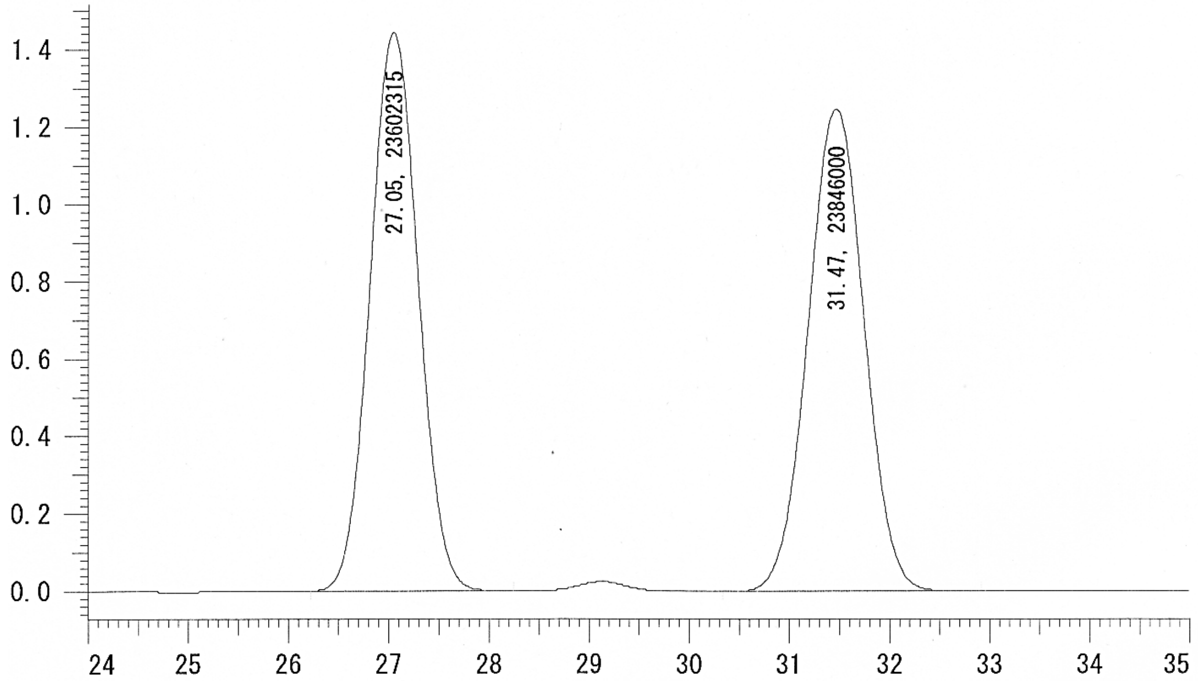

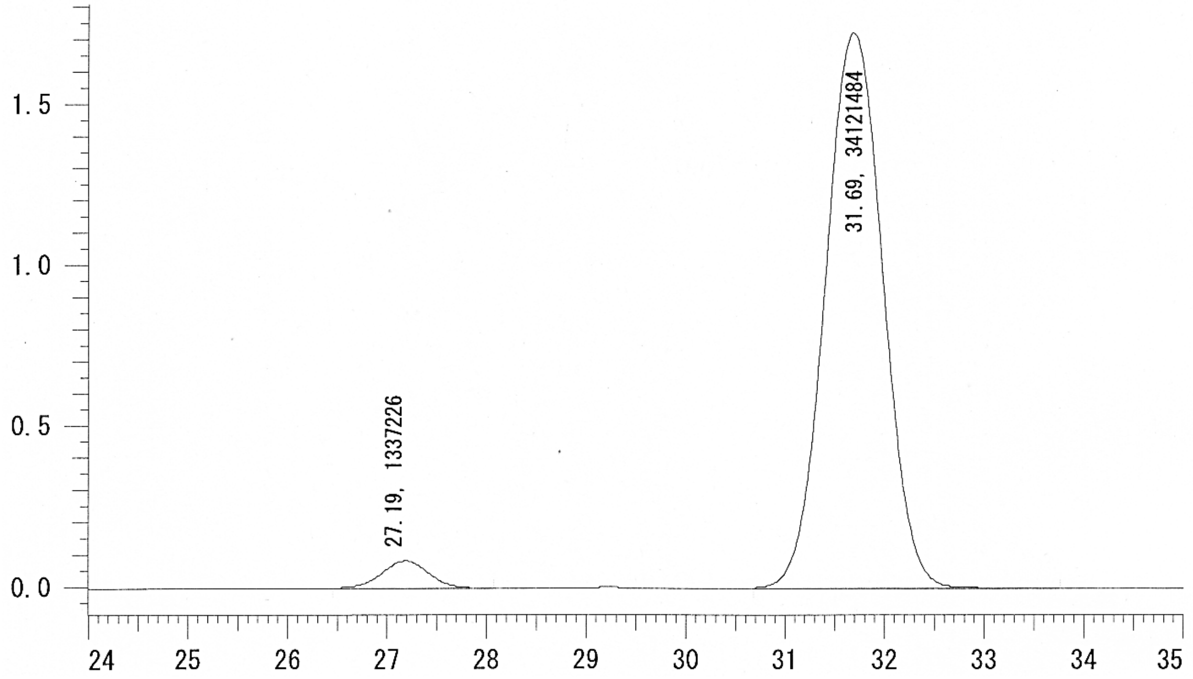

racemic				chiral			
No.	RT (min)	area	area (%)	No.	RT (min)	area	area (%)
1	27.05	23626420	49.769	1	25.10	724911	3.696
2	31.47	23846000	50.231	2	29.67	18890145	96.304

15.11 3ka

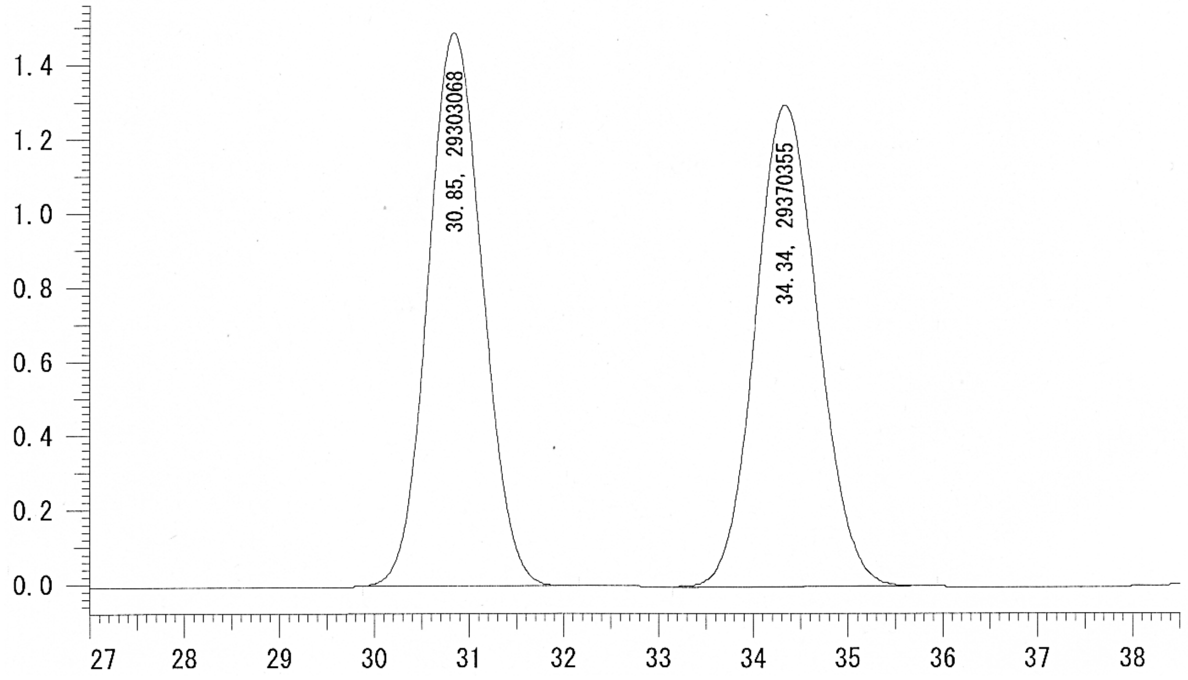

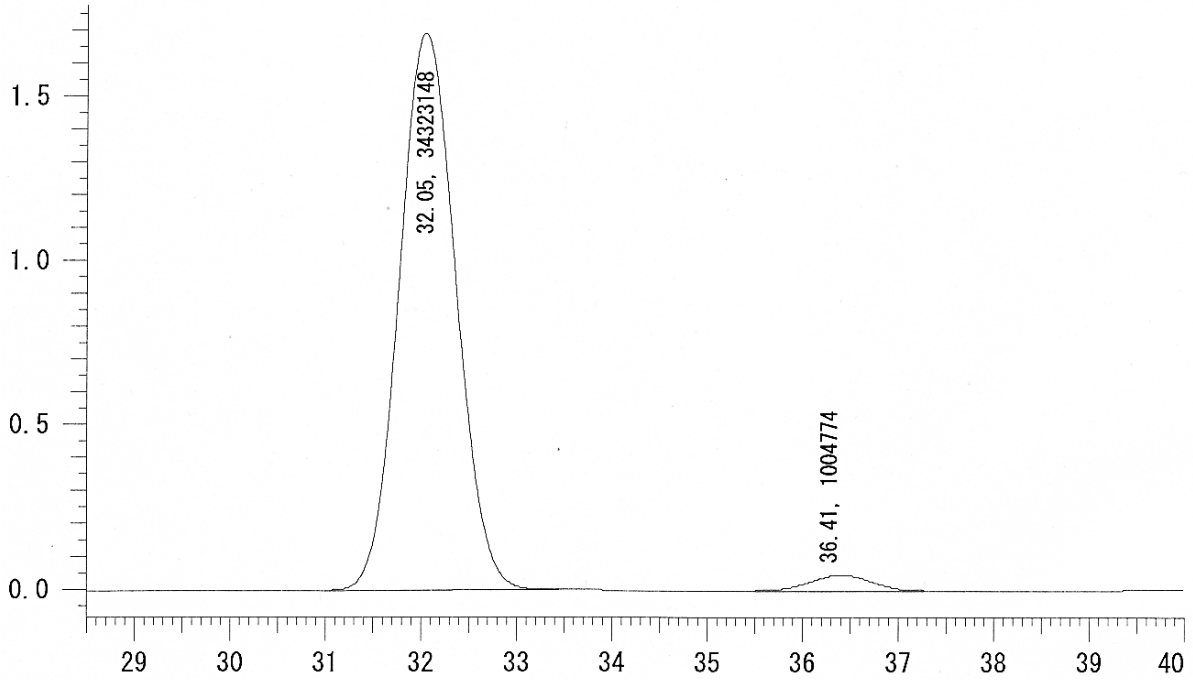

racemic				chiral			
No.	RT (min)	area	area (%)	No.	RT (min)	area	area (%)
1	30.85	29429676	50.026	1	32.05	34361248	97.080
2	34.34	29399177	49.974	2	36.41	1033514	2.920

15.12 3la

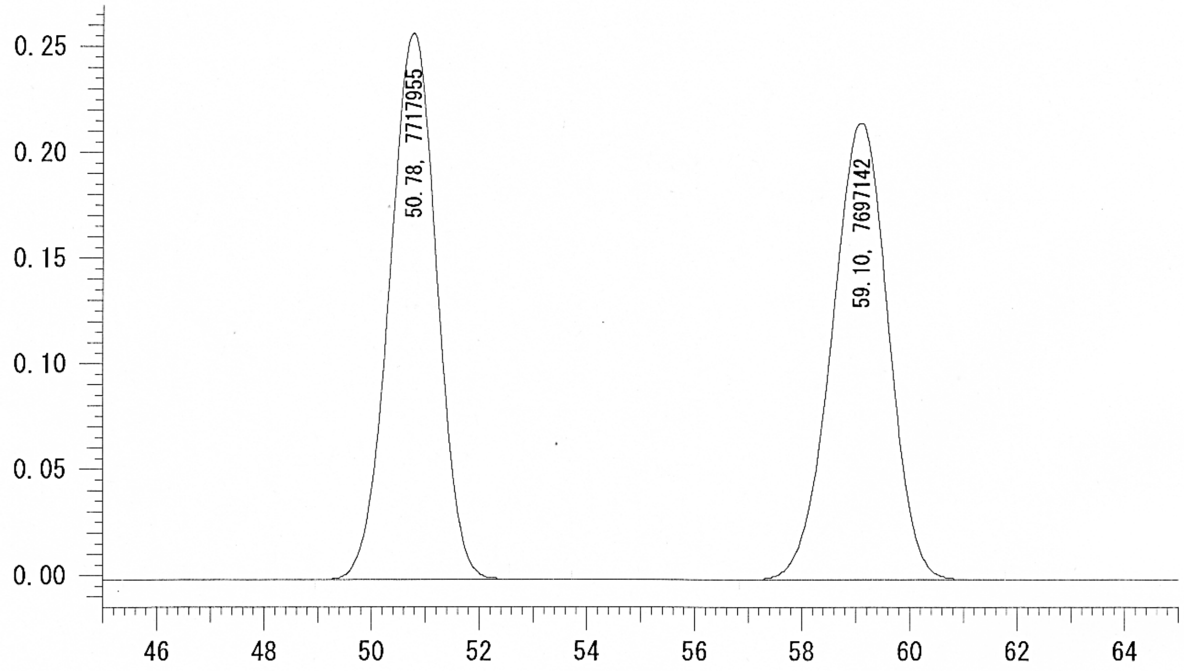

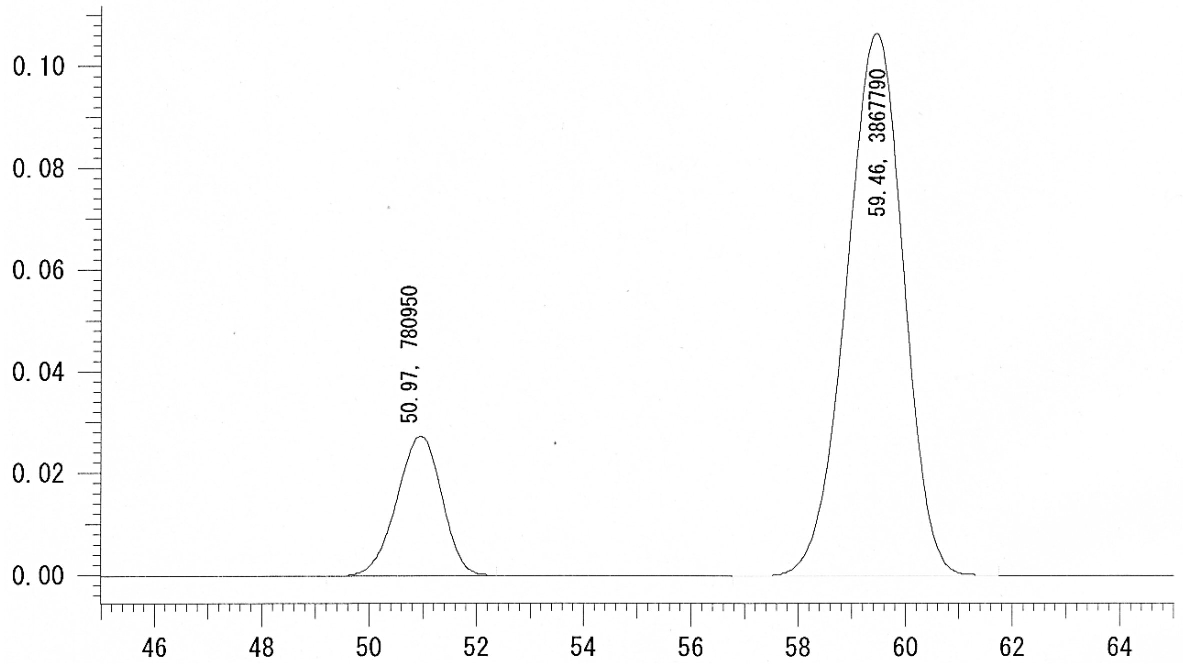

racemic				chiral			
No.	RT (min)	area	area (%)	No.	RT (min)	area	area (%)
1	50.78	7718719	50.053	1	50.97	787048	16.869
2	59.10	7702307	49.947	2	59.46	3878618	83.131

15.13 3ma

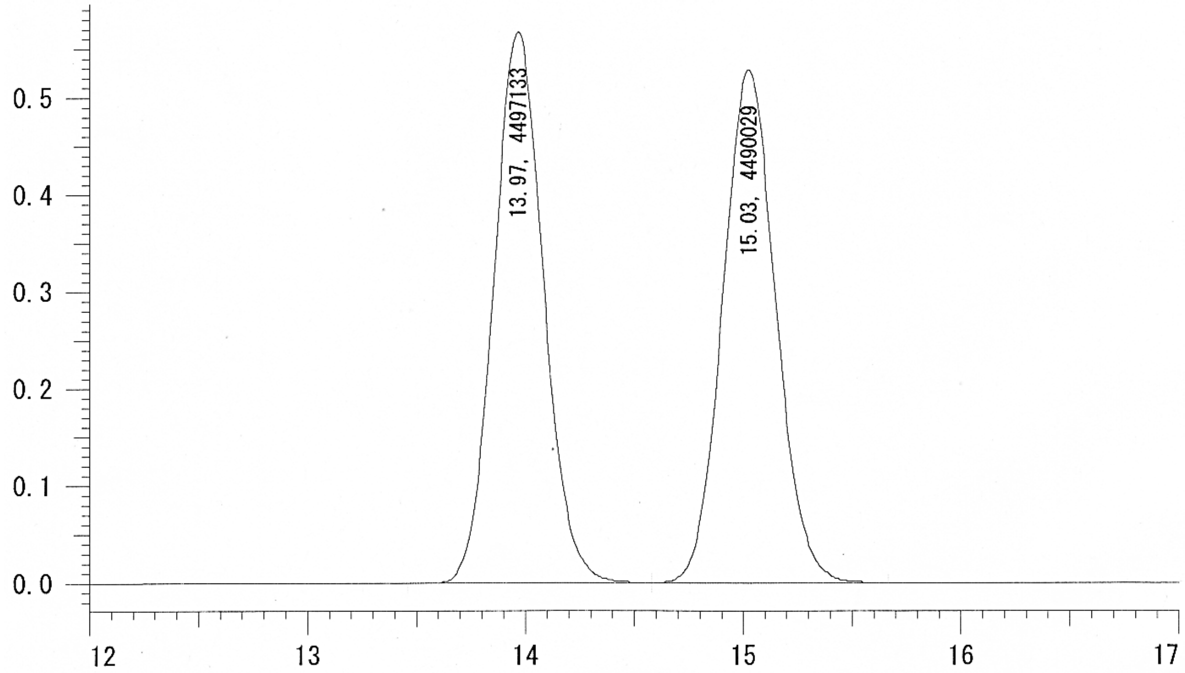

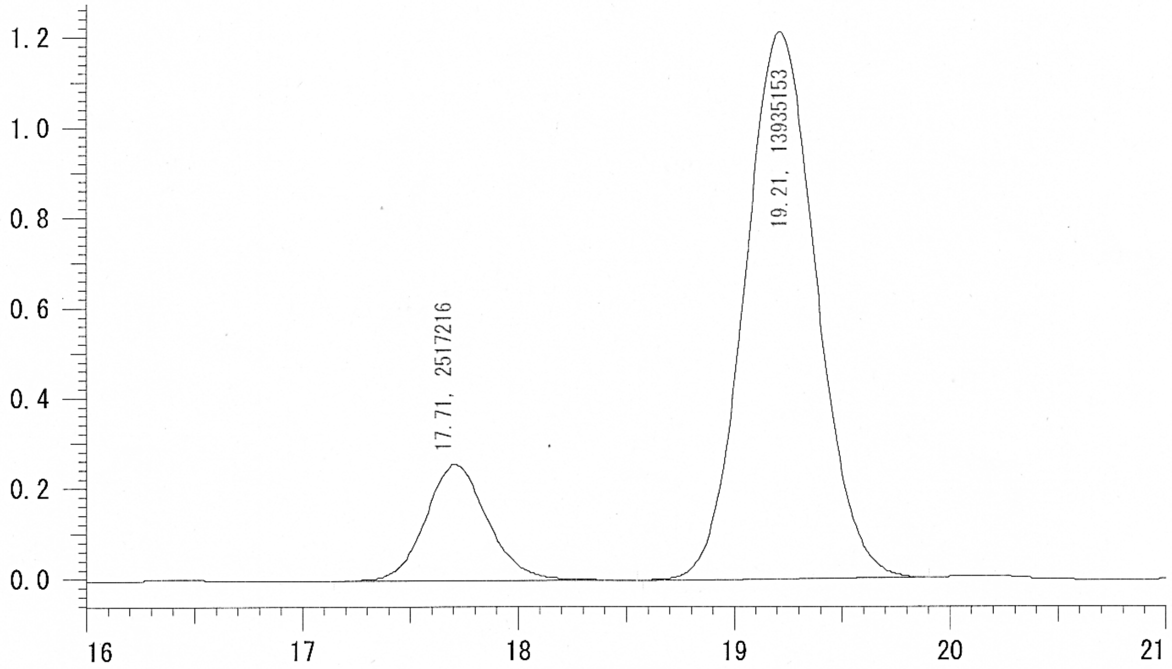

racemic				chiral			
No.	RT (min)	area	area (%)	No.	RT (min)	area	area (%)
1	17.76	4735900	50.036	1	17.71	2517216	15.298
2	19.23	4729104	49.964	2	19.22	13937451	84.702

15.14 3na

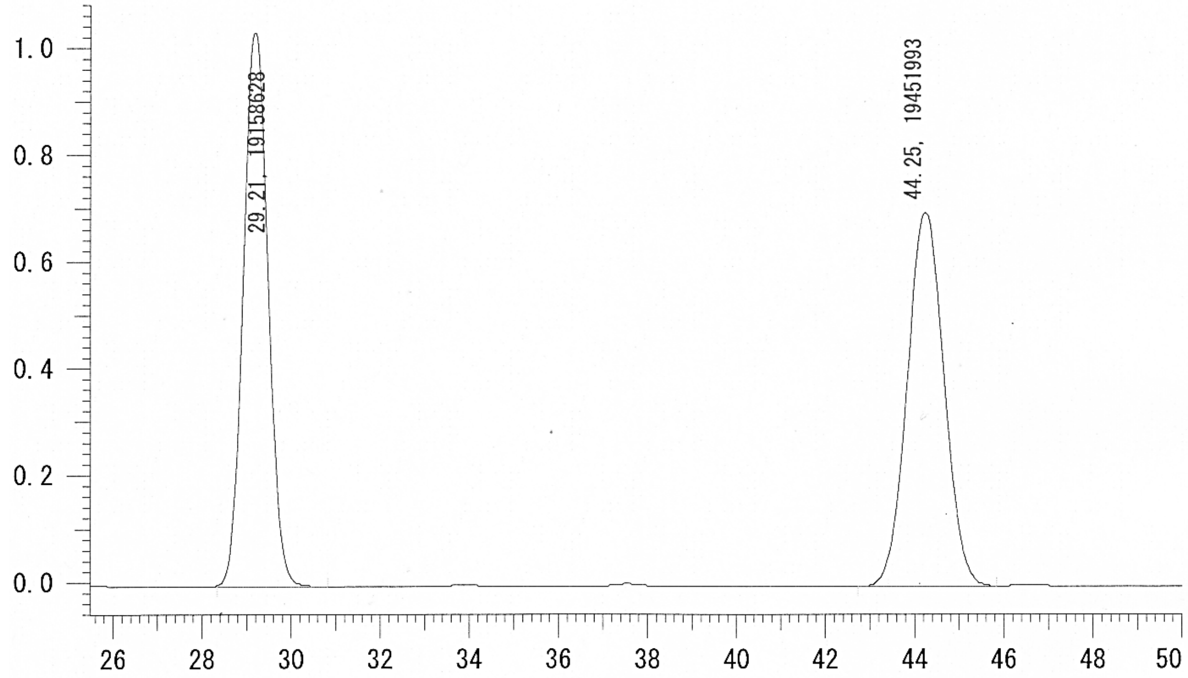

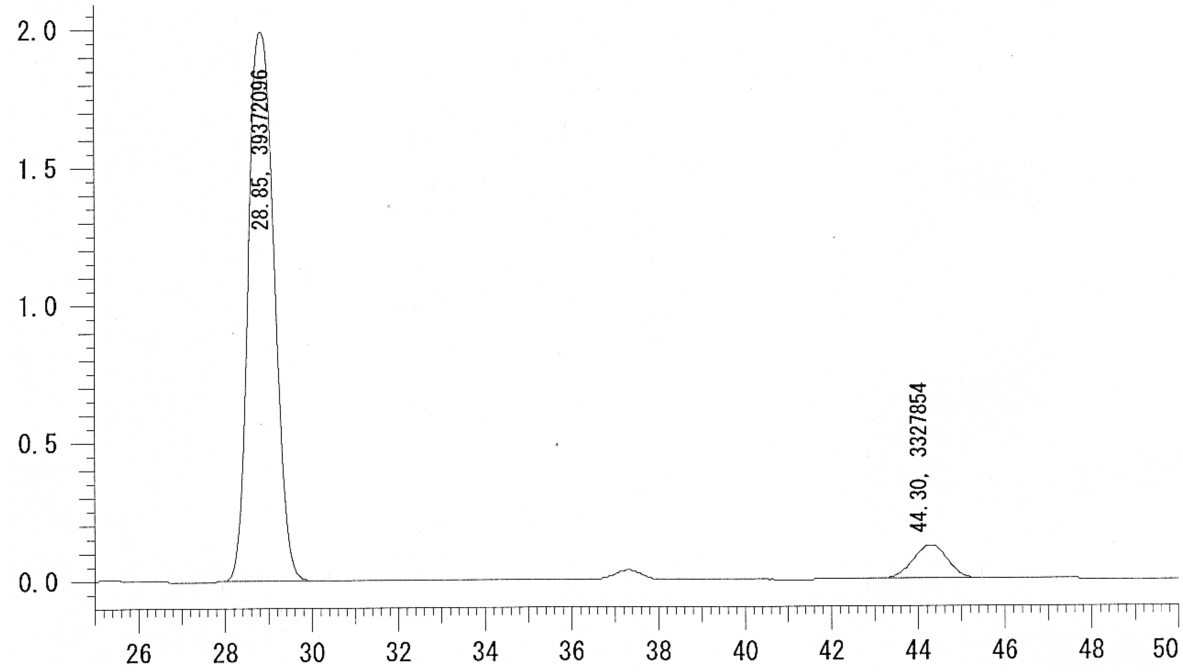

racemic				chiral			
No.	RT (min)	area	area (%)	No.	RT (min)	area	area (%)
1	29.21	19223011	49.705	1	28.85	39444992	92.177
2	44.25	19451328	50.295	2	44.30	3347562	7.823

15.15 3oa

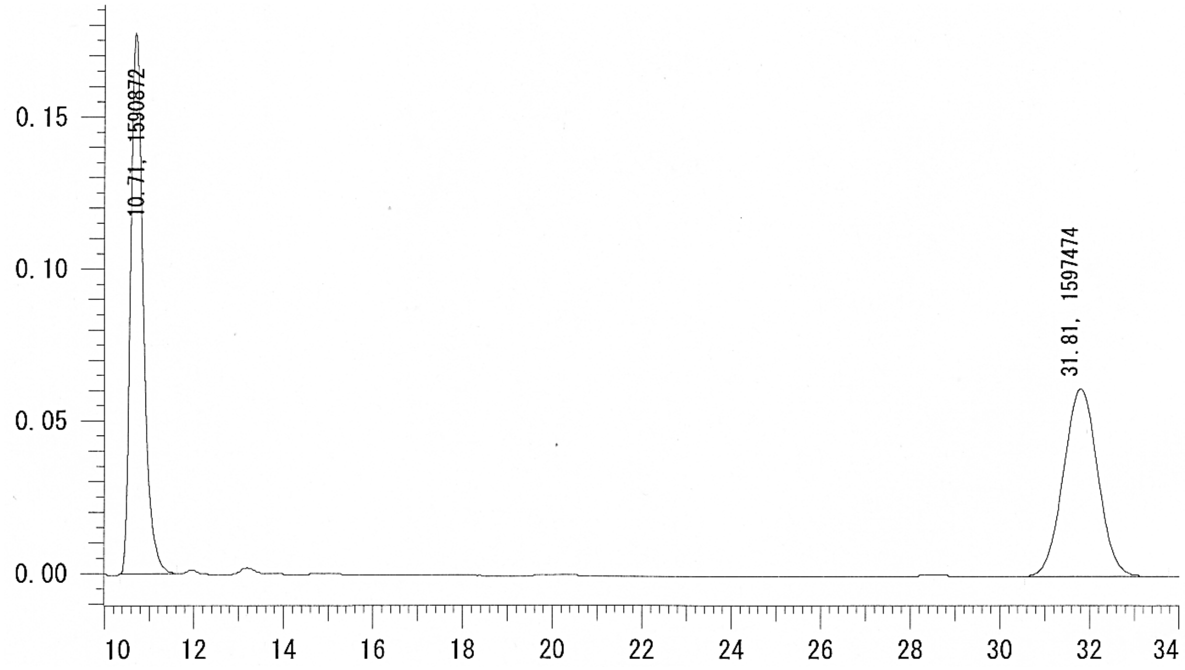

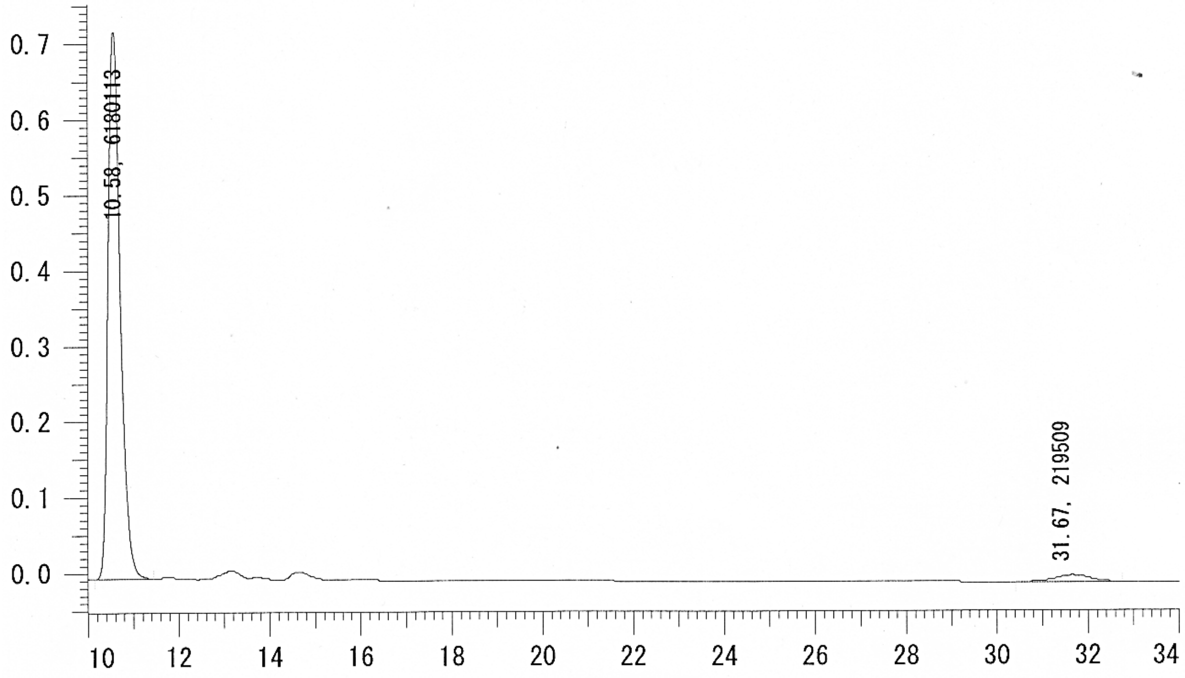

racemic				chiral			
No.	RT (min)	area	area (%)	No.	RT (min)	area	area (%)
1	10.71	1594996	49.845	1	10.58	6181633	96.520
2	31.81	1604917	50.155	2	31.67	222865	3.480

15.16 3pa

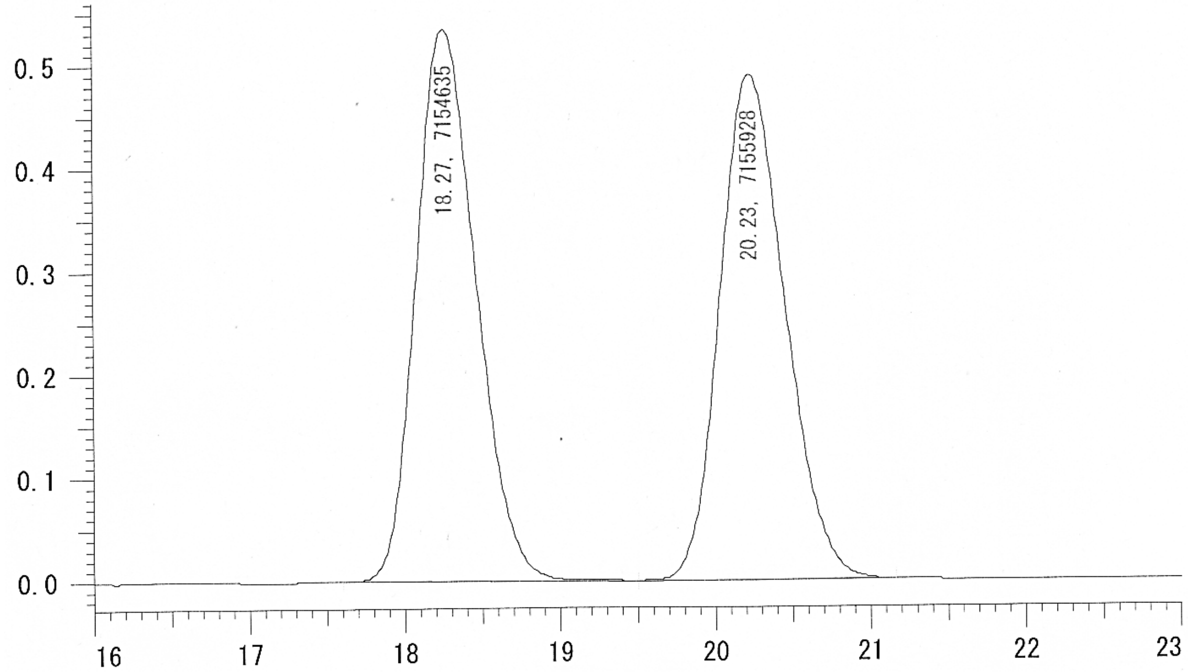

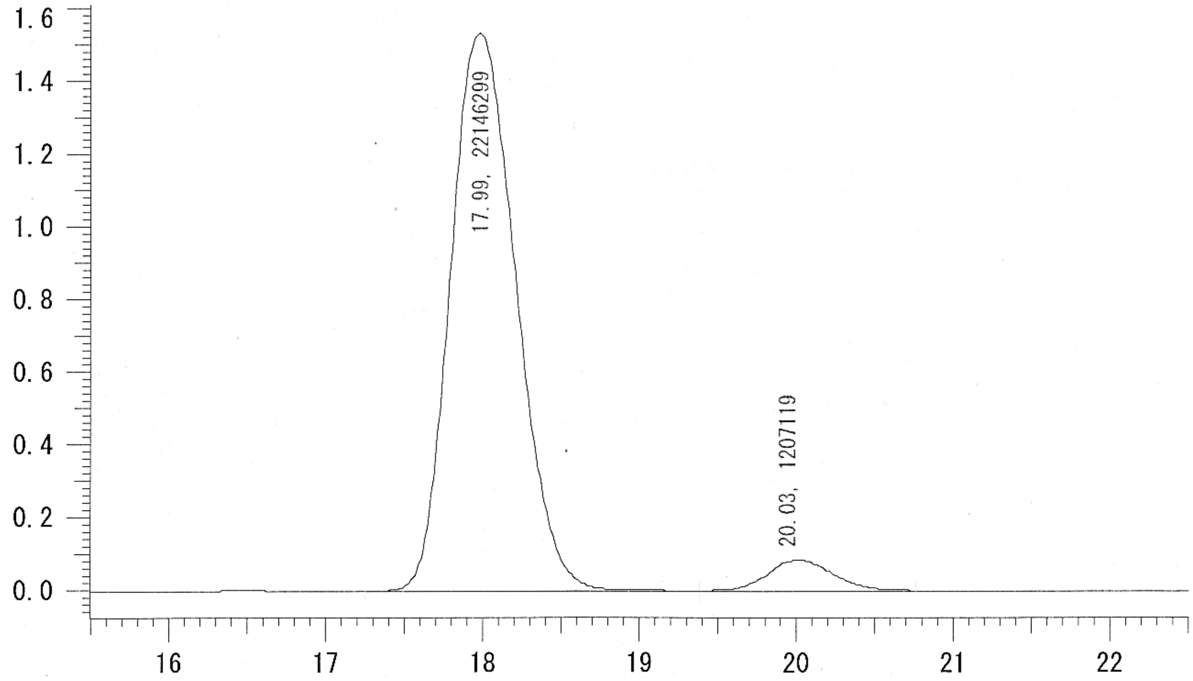

racemic				chiral			
No.	RT (min)	area	area (%)	No.	RT (min)	area	area (%)
1	18.27	7154635	49.933	1	17.99	22146299	94.795
2	20.23	7173789	50.067	2	20.03	1215950	5.205

15.17 3qa

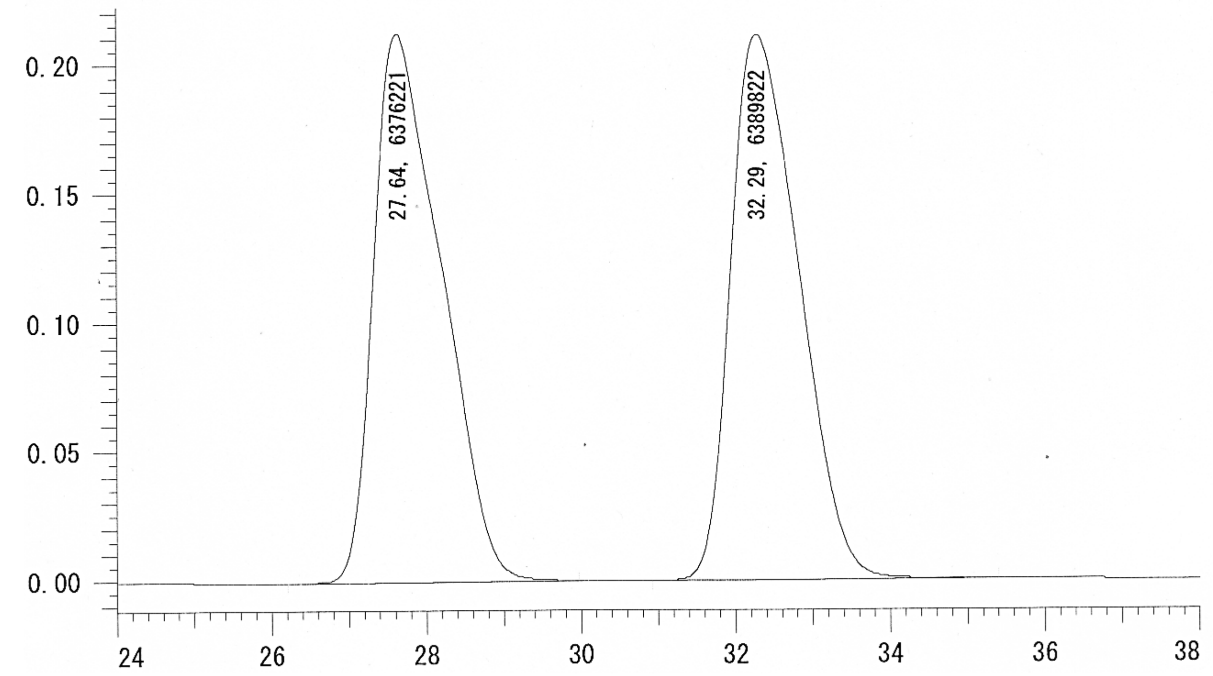

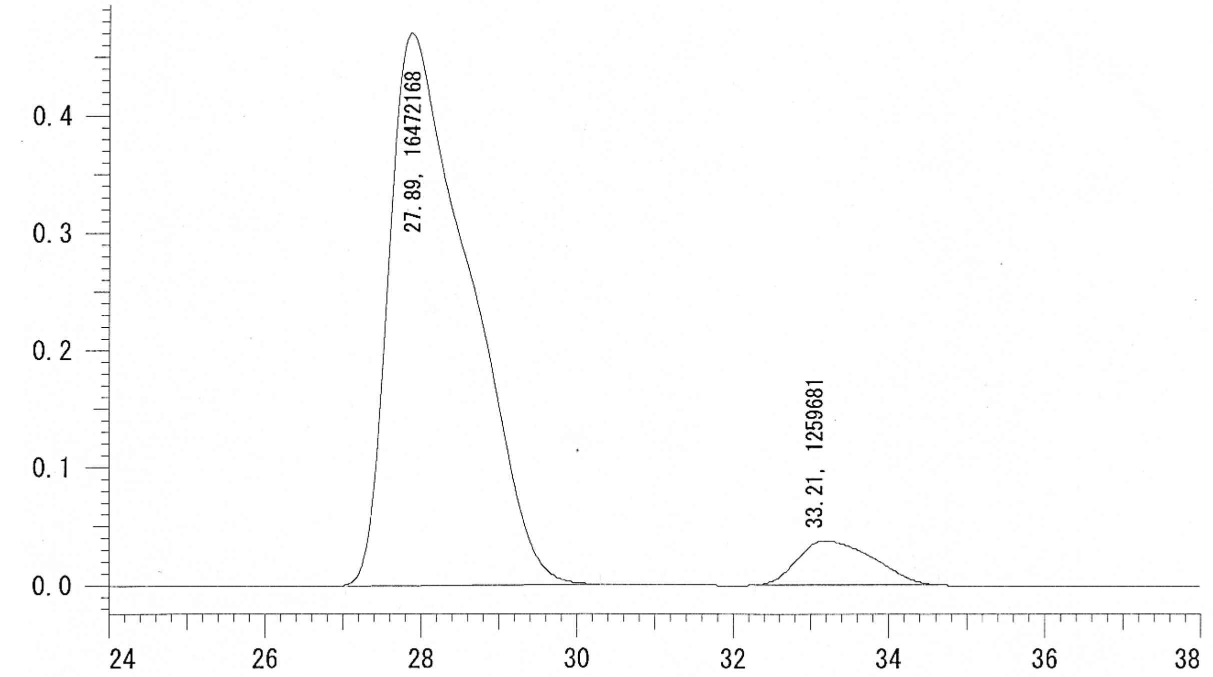

racemic				chiral			
No.	RT (min)	area	area (%)	No.	RT (min)	area	area (%)
1	27.64	6381941	49.969	1	27.89	16472168	92.896
2	32.29	6389822	50.031	2	33.21	1259681	7.104

15.18 3ra

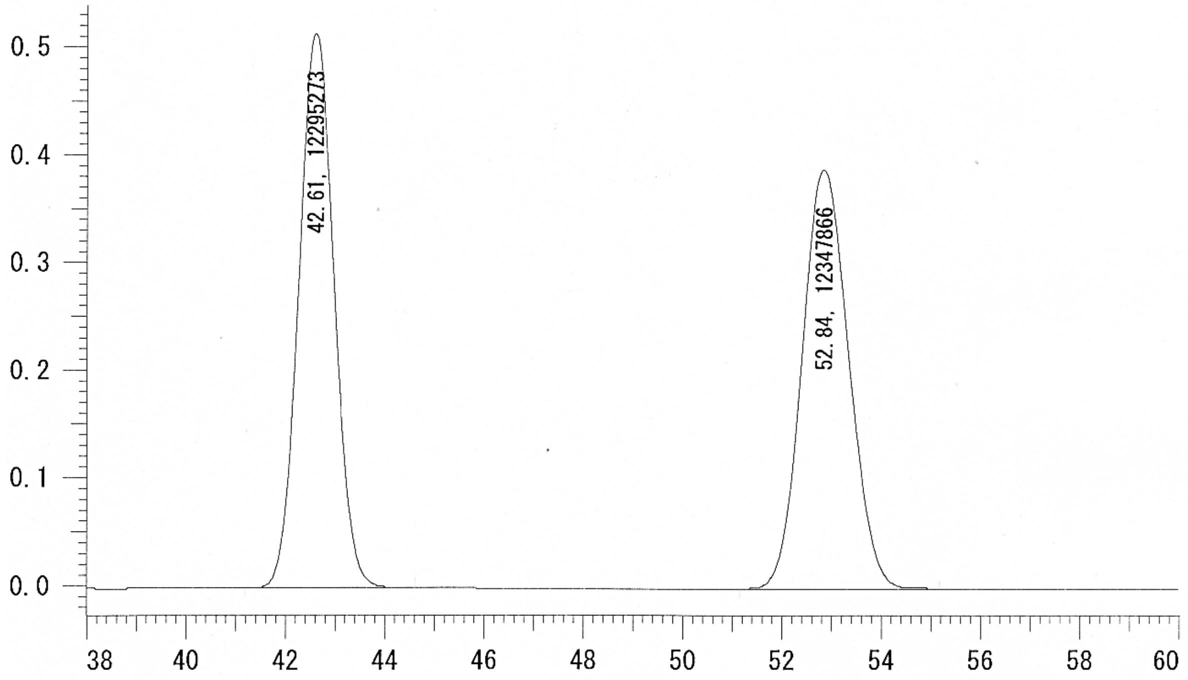

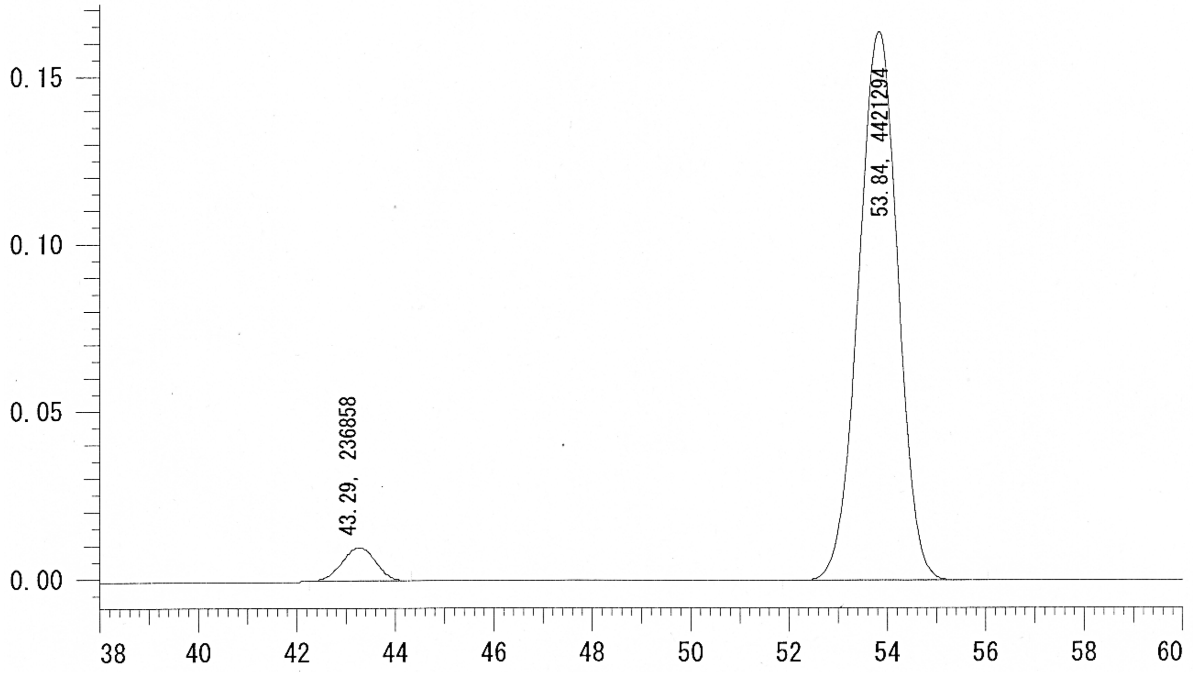

racemic				chiral			
No.	RT (min)	area	area (%)	No.	RT (min)	area	area (%)
1	42.61	12295275	49.885	1	43.29	241113	5.171
2	52.84	12351940	50.115	2	53.84	4421294	94.829

15.19 3cb

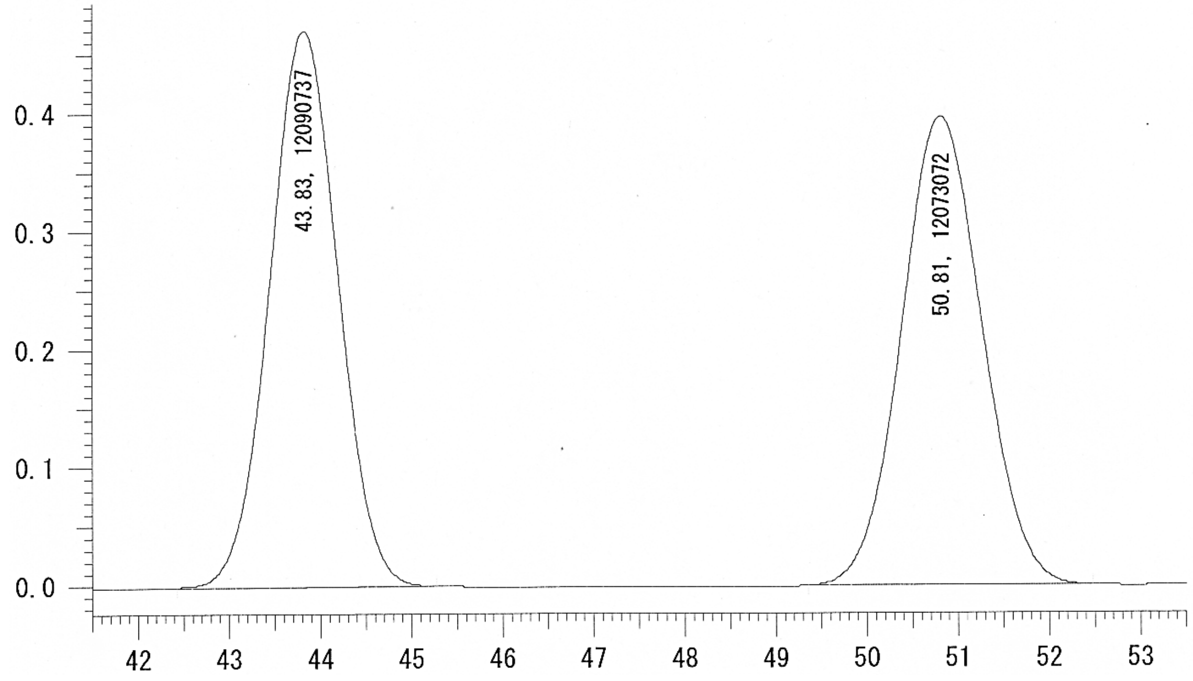

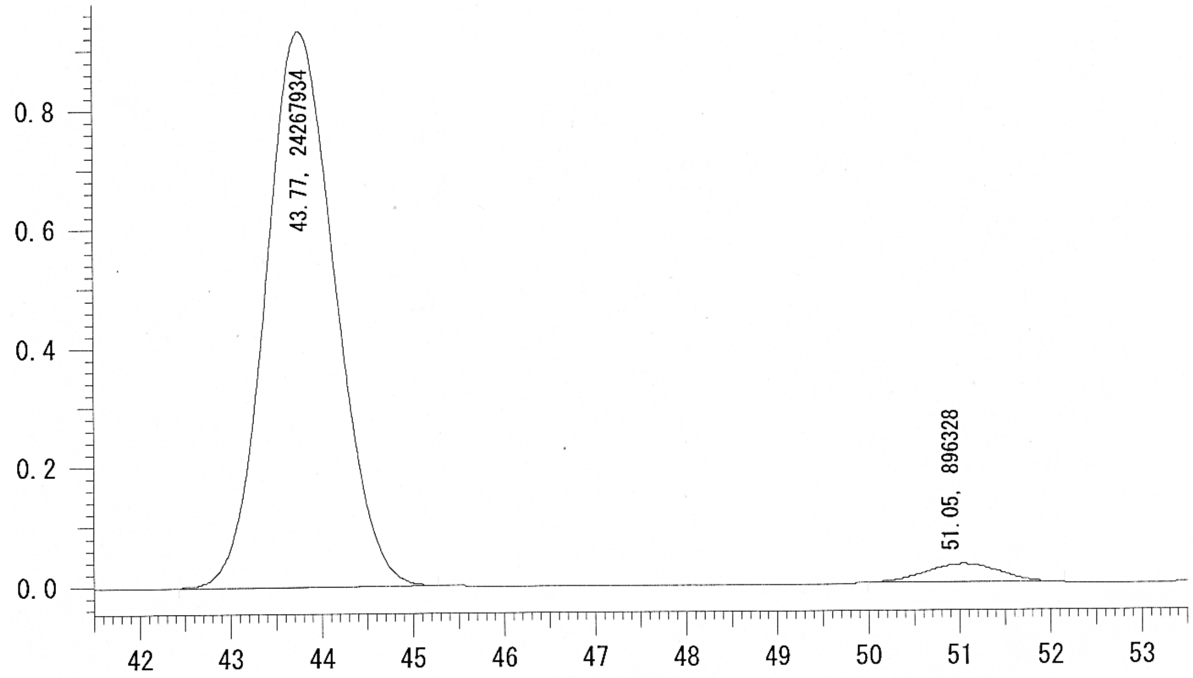

racemic				Chiral			
No.	RT (min)	Area	area (%)	No.	RT (min)	area	area (%)
1	43.83	12138321	50.034	1	43.77	24267934	96.438
2	50.81	12121898	49.966	2	51.05	896328	3.562

15.20 3cc

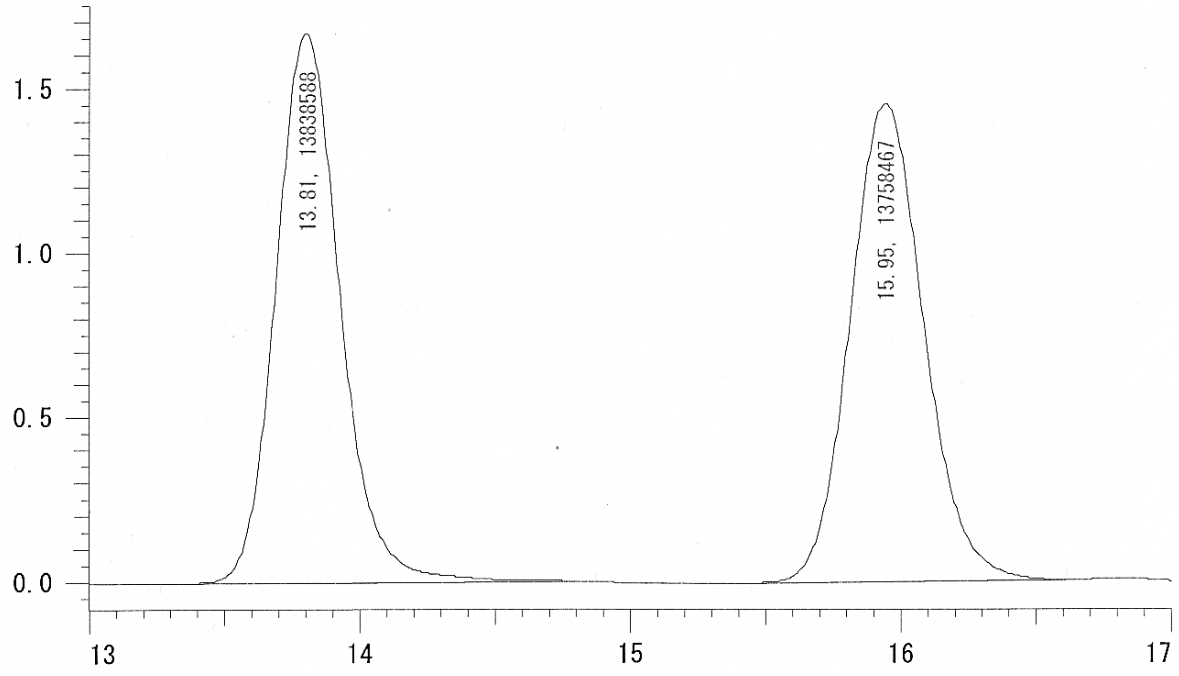

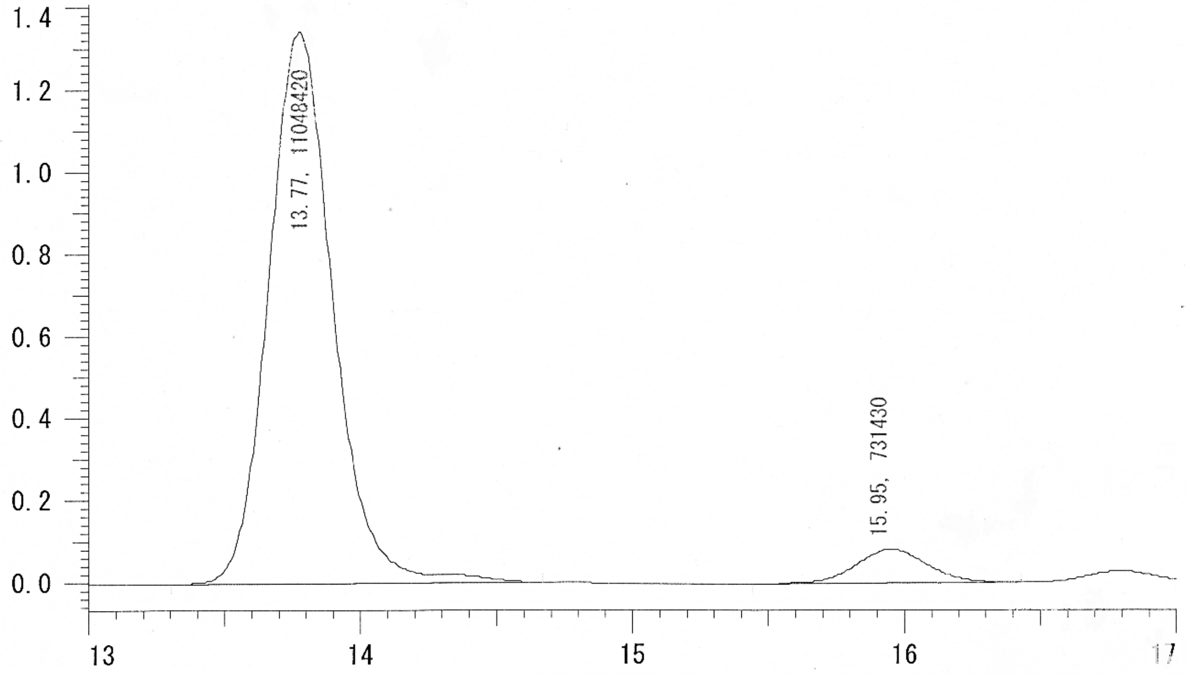

racemic				Chiral			
No.	RT (min)	area	area (%)	No.	RT (min)	area	area (%)
1	13.81	13884606	50.185	1	13.77	11107712	93.822
2	18.01	13782251	49.815	2	15.95	731445	6.178

15.21 3cd

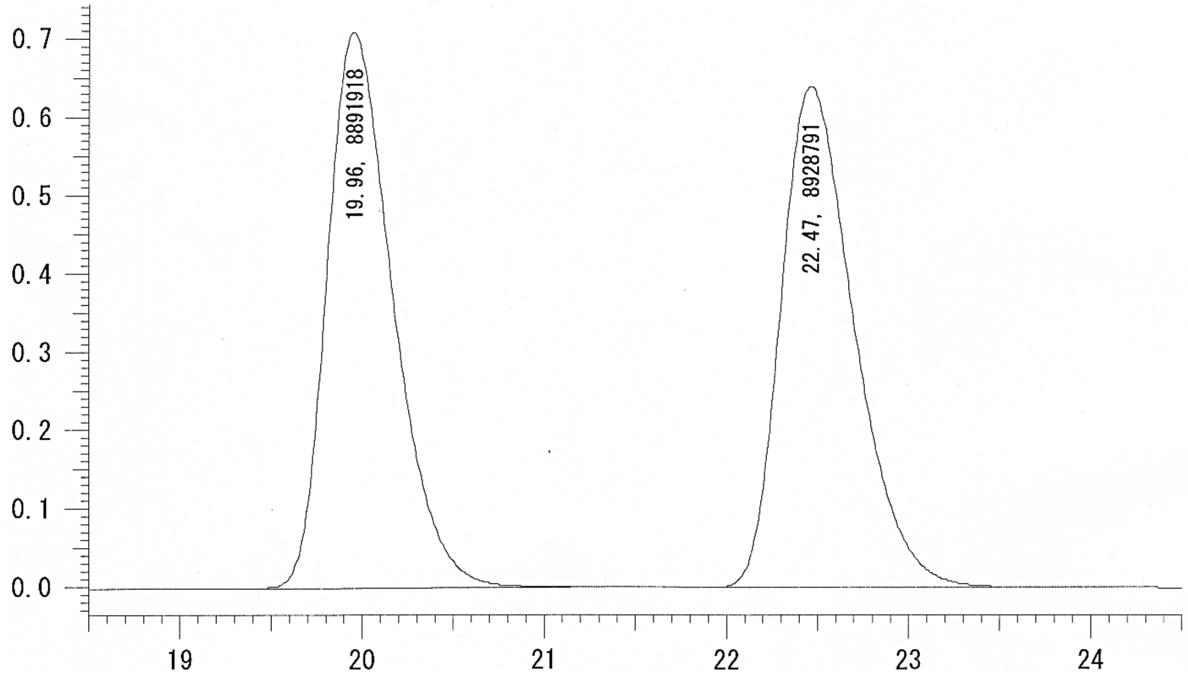

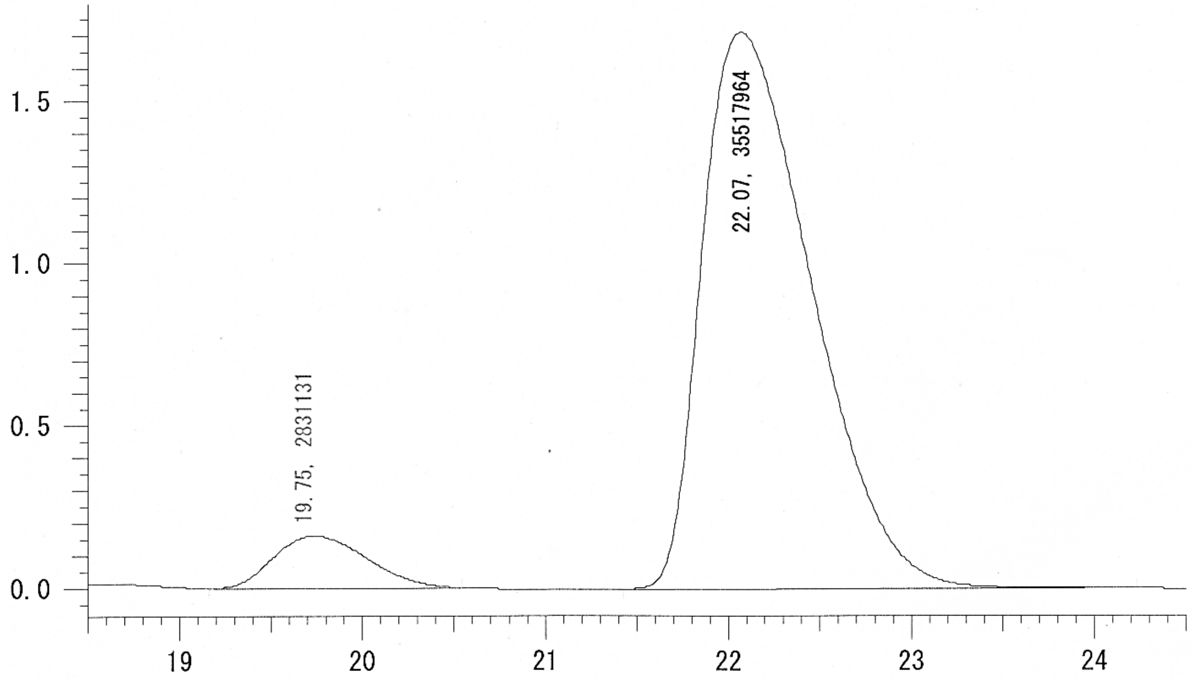

racemic				Chiral			
No.	RT (min)	area	area (%)	No.	RT (min)	area	area (%)
1	19.96	8917245	49.939	1	19.75	2831131	7.383
2	22.47	8939148	50.061	2	22.07	35517964	92.617

15.22 3ce

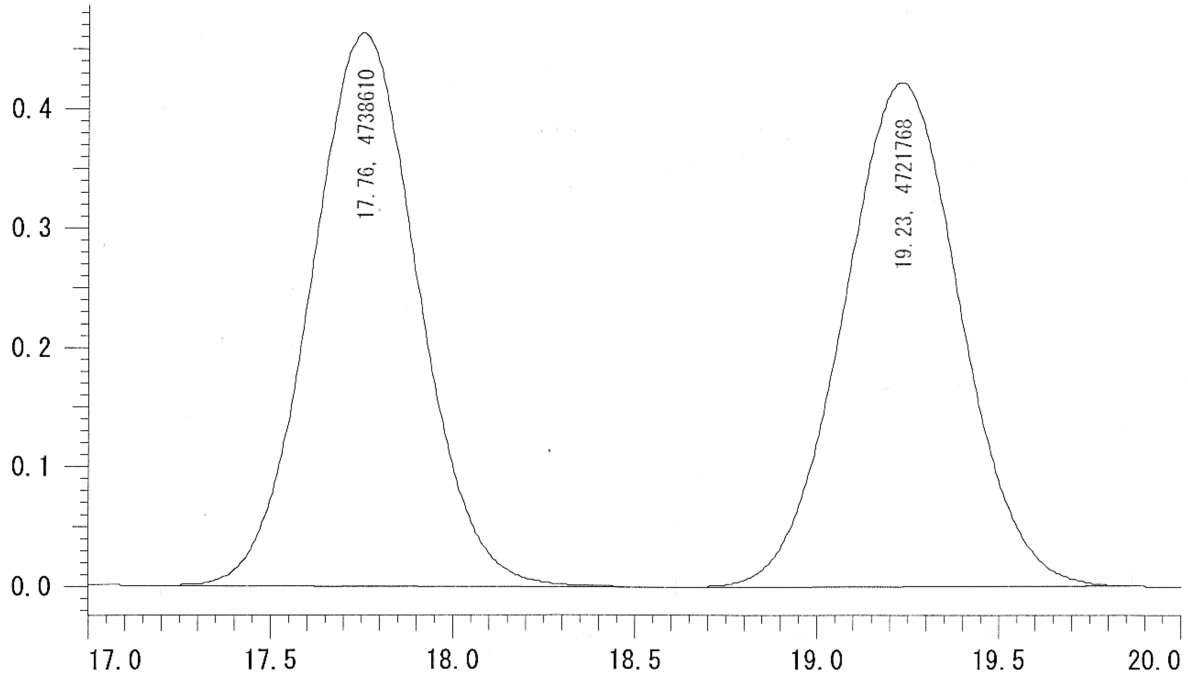

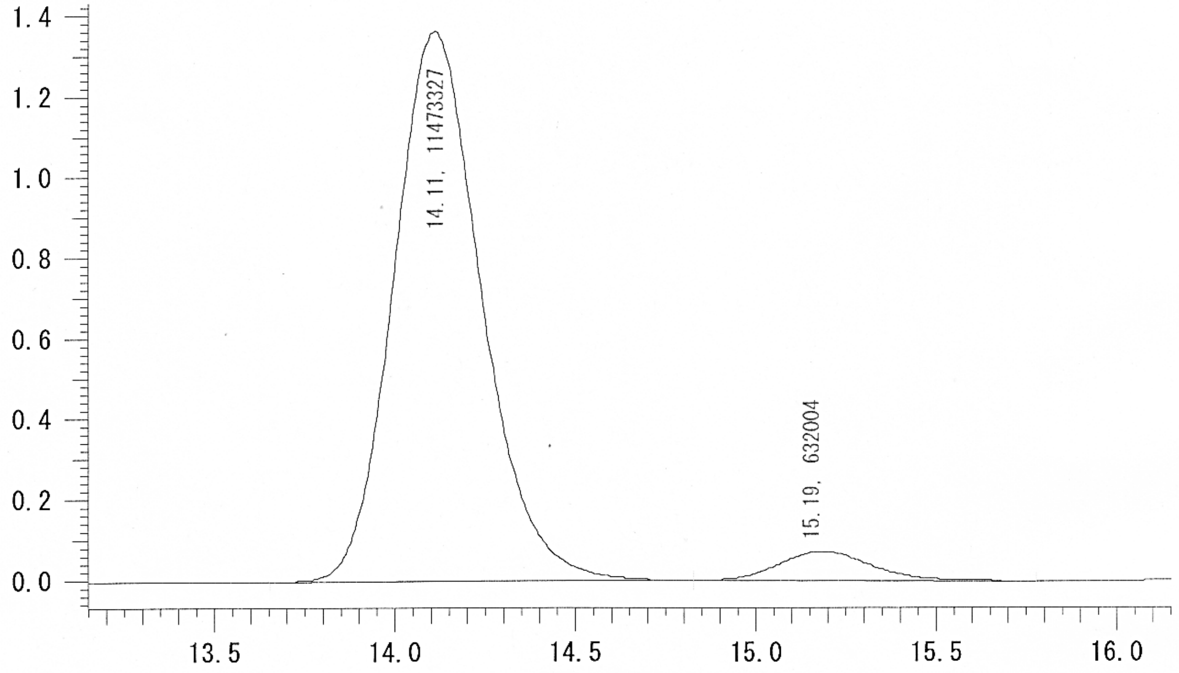

racemic				Chiral			
No.	RT (min)	area	area (%)	No.	RT (min)	area	area (%)
1	13.97	4497133	50.031	1	14.11	11473327	94.780
2	15.03	4491476	49.969	2	15.19	631894	5.220

15.23 3cf

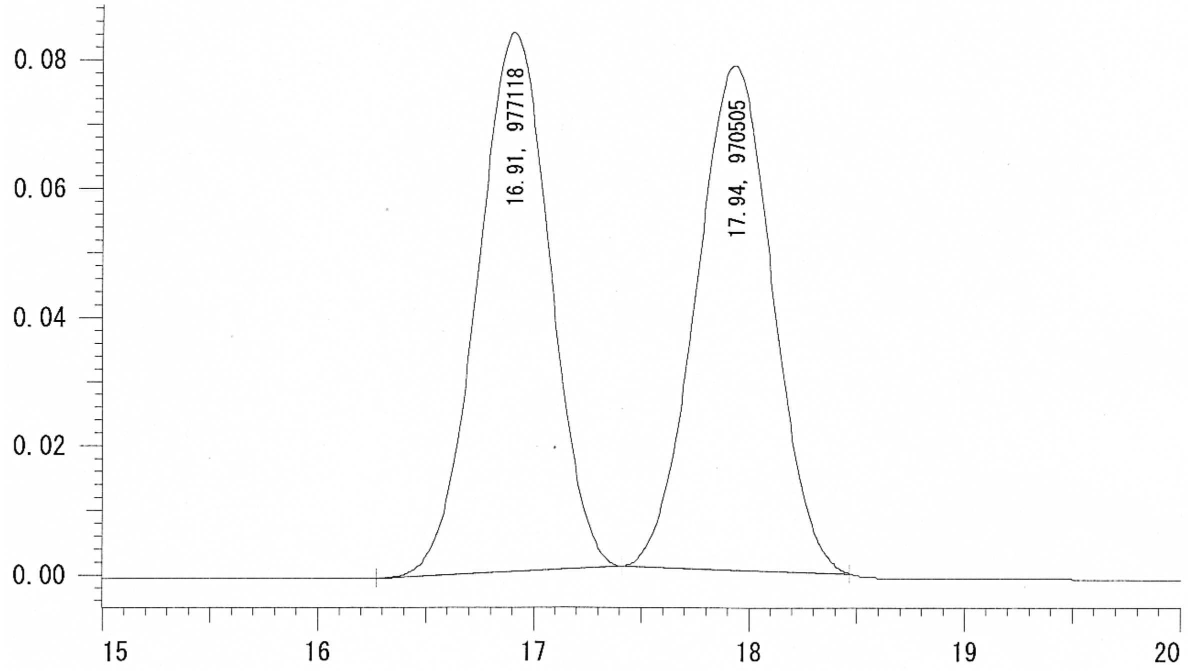

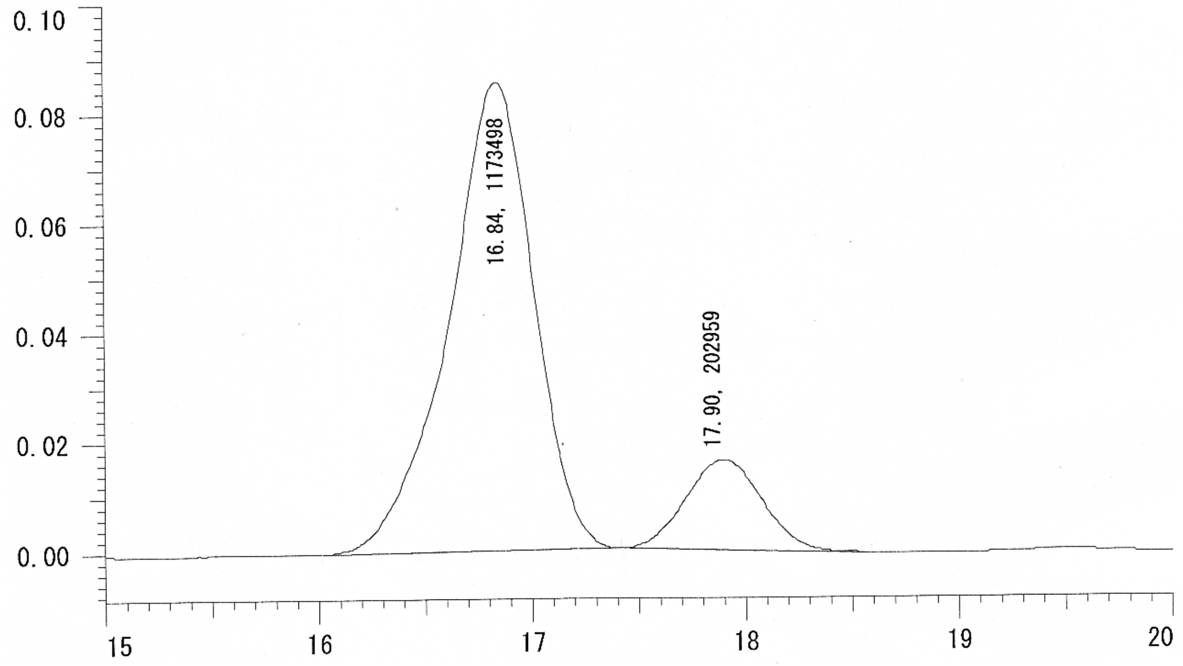

racemic				Chiral			
No.	RT (min)	area	area (%)	No.	RT (min)	area	area (%)
1	16.91	977460	50.104	1	16.84	1173498	85.255
2	17.93	973388	49.896	2	17.90	202959	14.745

15.24 3cg

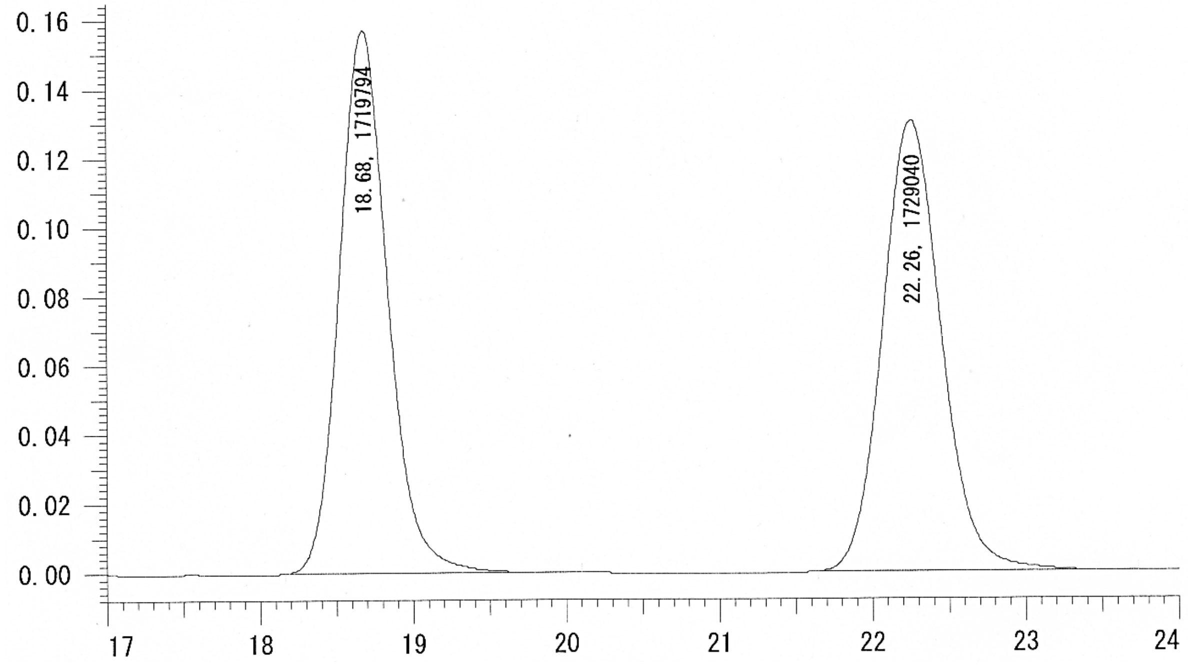

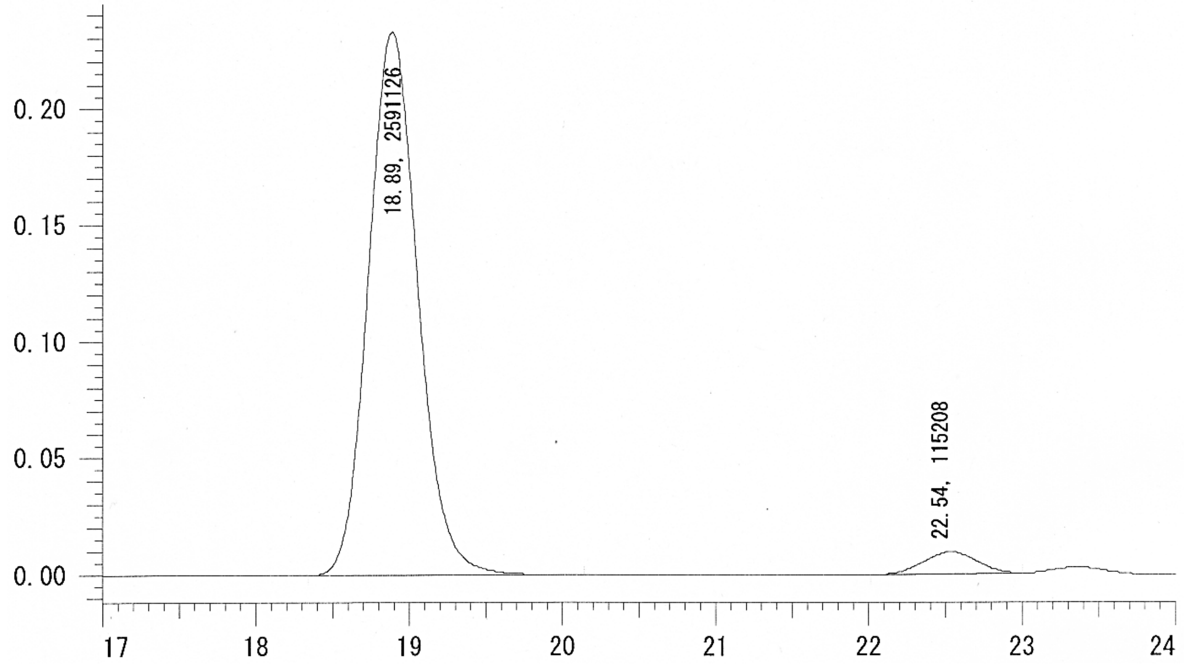

racemic				Chiral			
No.	RT (min)	area	area (%)	No.	RT (min)	area	area (%)
1	18.68	1726788	49.892	1	18.89	2595329	95.219
2	22.26	1734263	50.108	2	22.54	130300	4.781

15.25 3ch

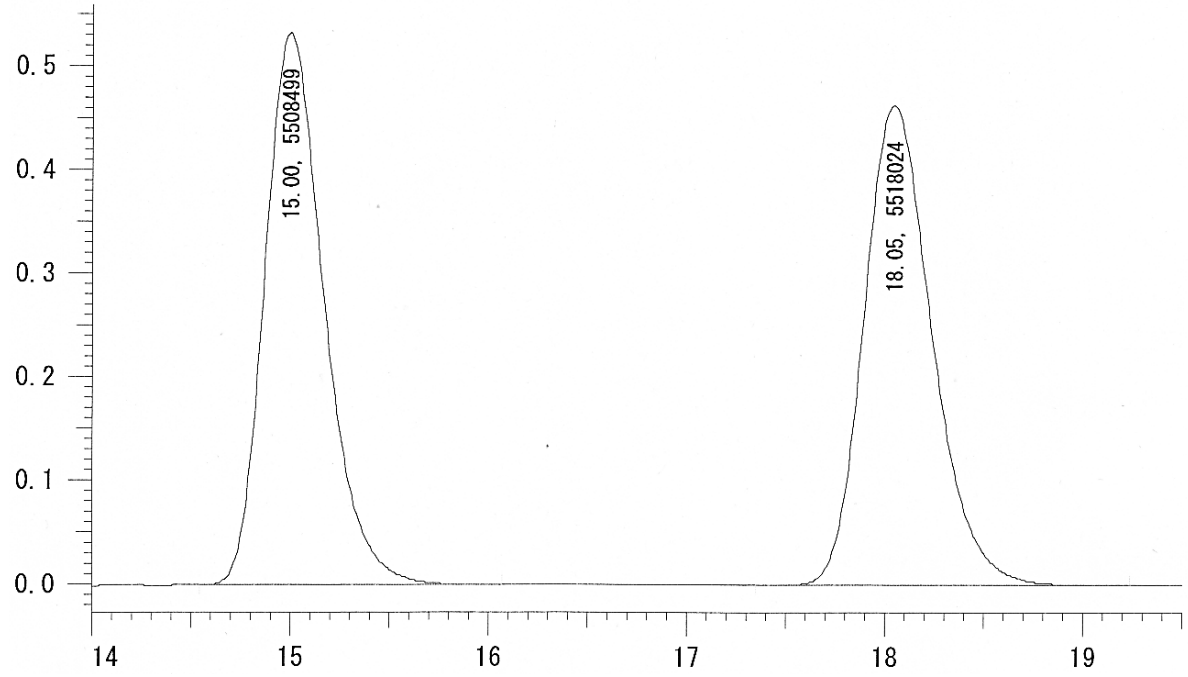

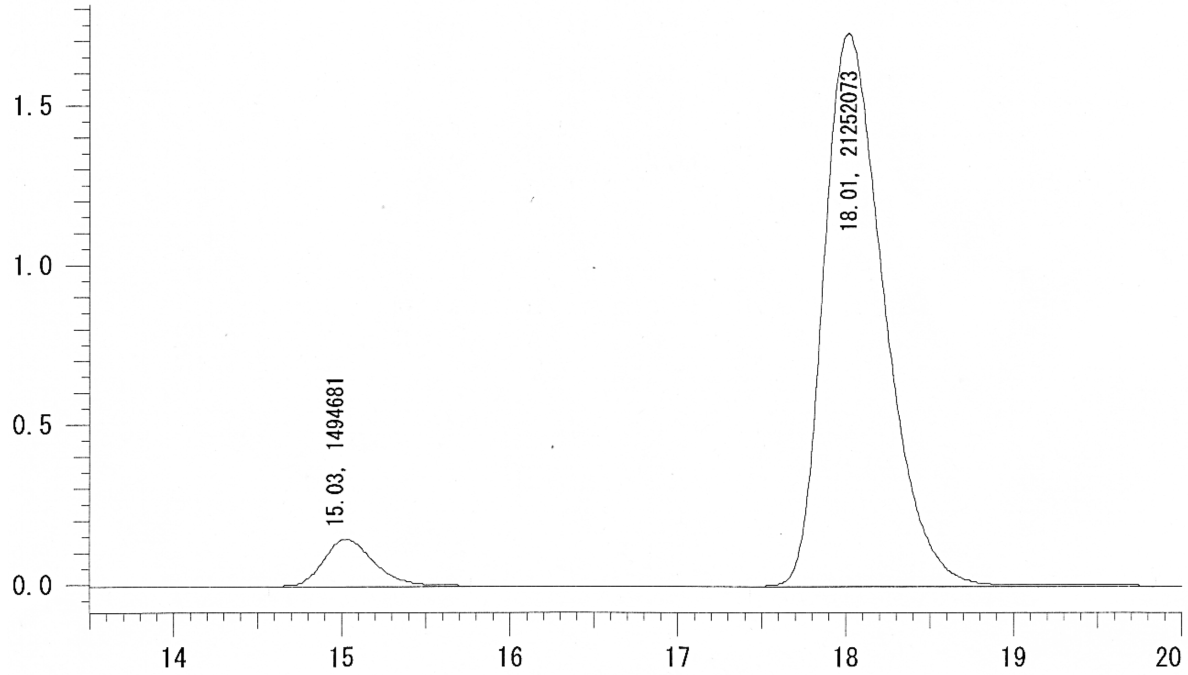

racemic				chiral			
No.	RT (min)	area	area (%)	No.	RT (min)	area	area (%)
1	15.00	5521066	49.956	1	15.03	1499935	6.590
2	18.05	5530829	50.044	2	18.01	21261080	93.410

15.26 3fi

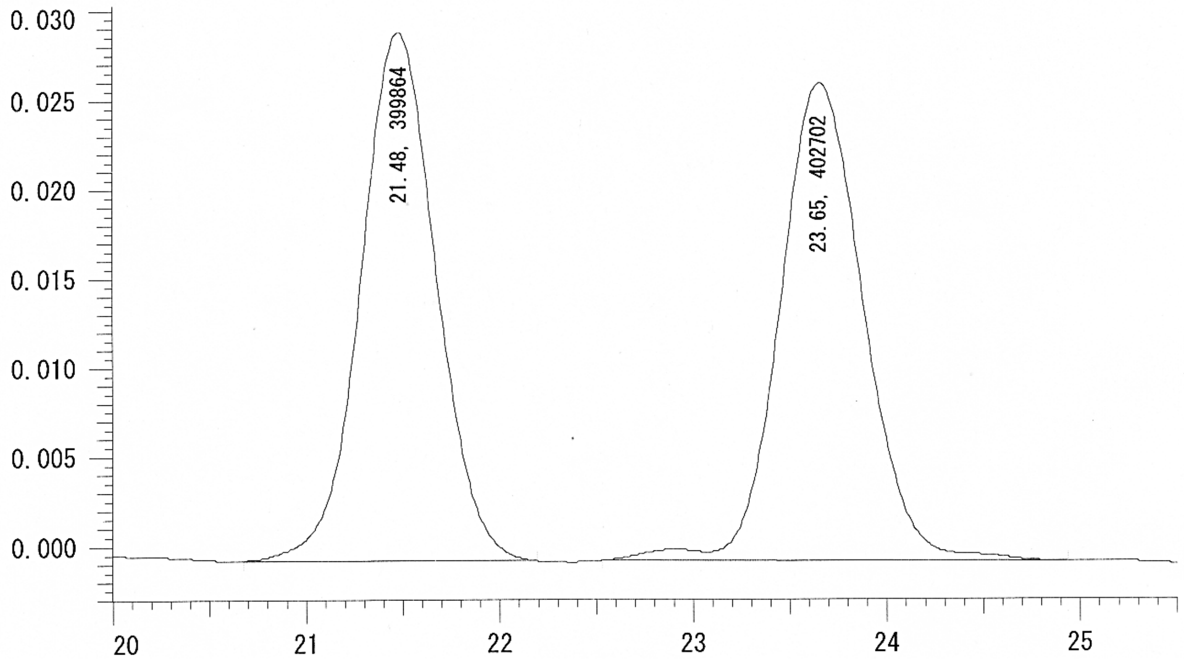

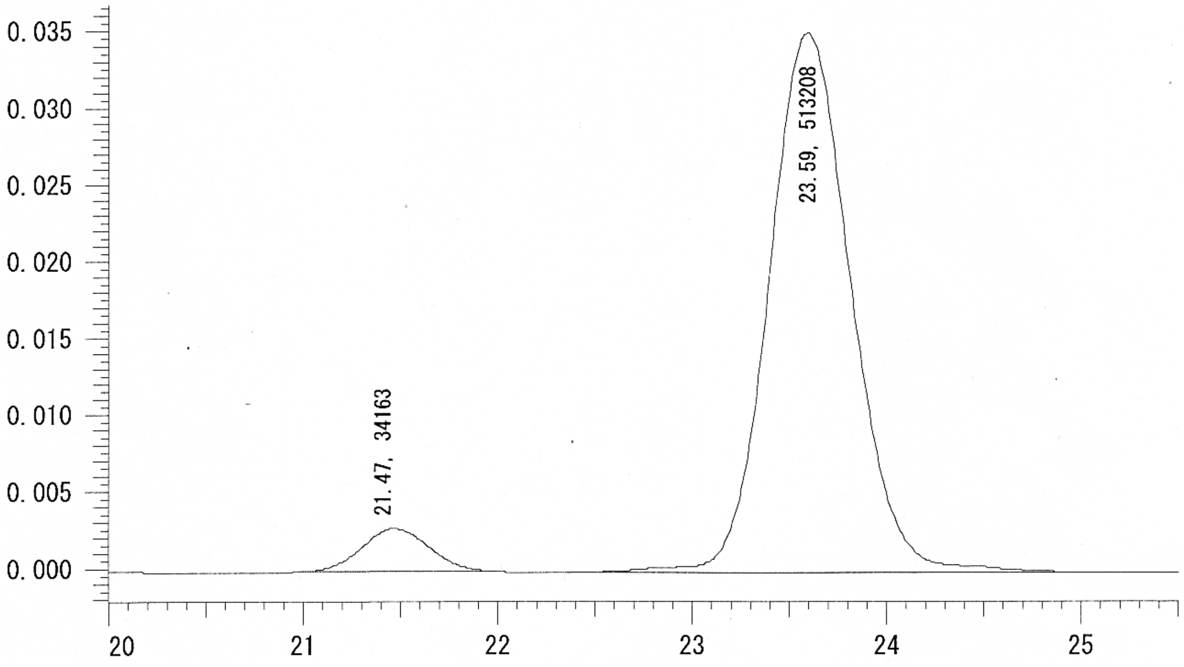

racemic				chiral			
No.	RT (min)	area	area (%)	No.	RT (min)	area	area (%)
1	21.48	401582	49.725	1	21.47	35294	6.435
2	23.65	406025	50.275	2	23.59	513208	93.565

15.27 3fj

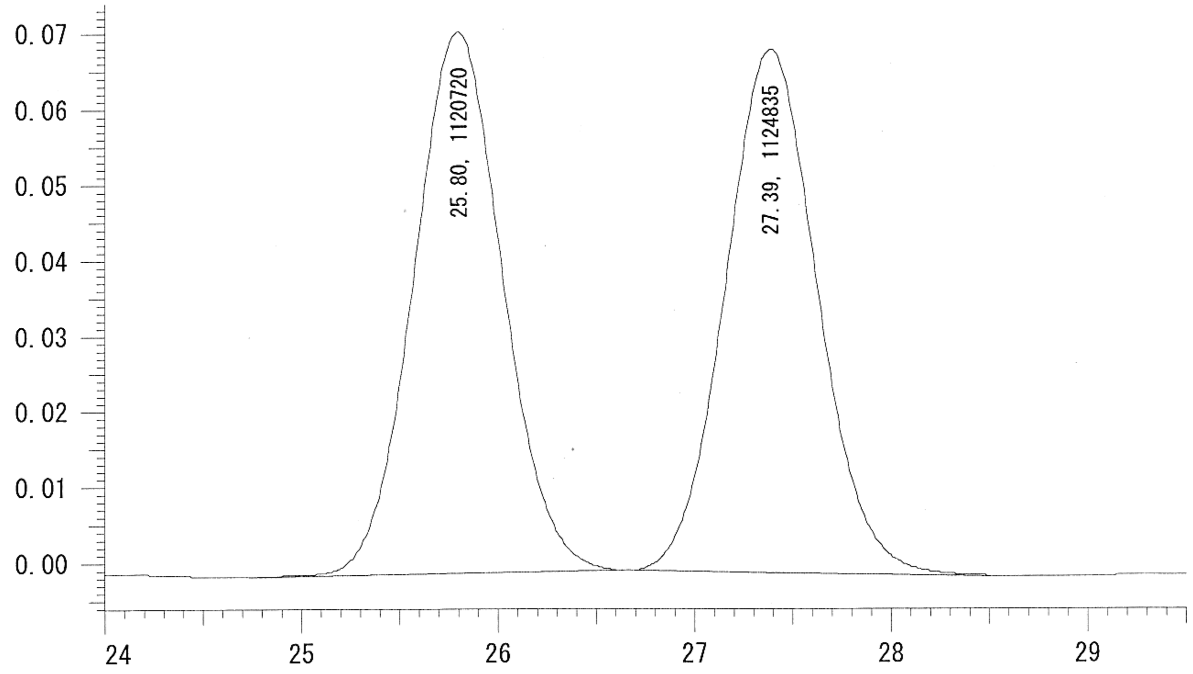

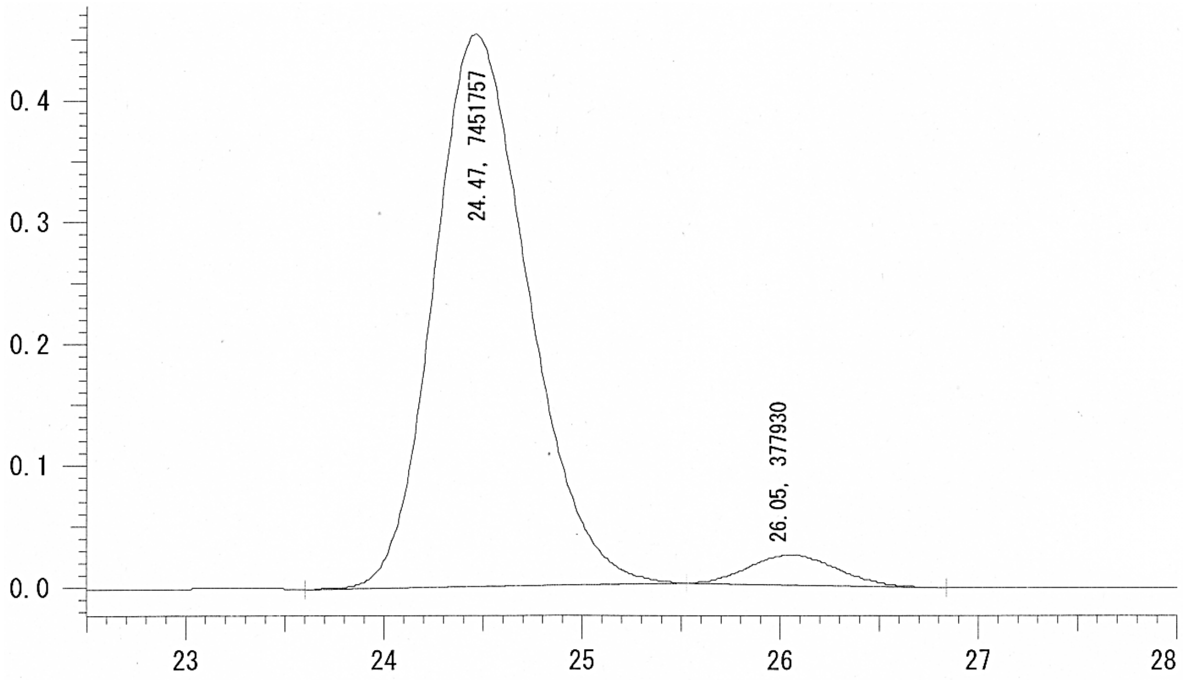

racemic				chiral			
No.	RT (min)	area	area (%)	No.	RT (min)	area	area (%)
1	25.80	1120888	49.918	1	24.47	7451757	95.173
2	27.39	1124558	50.082	2	26.05	377930	4.827

15.28 3gk

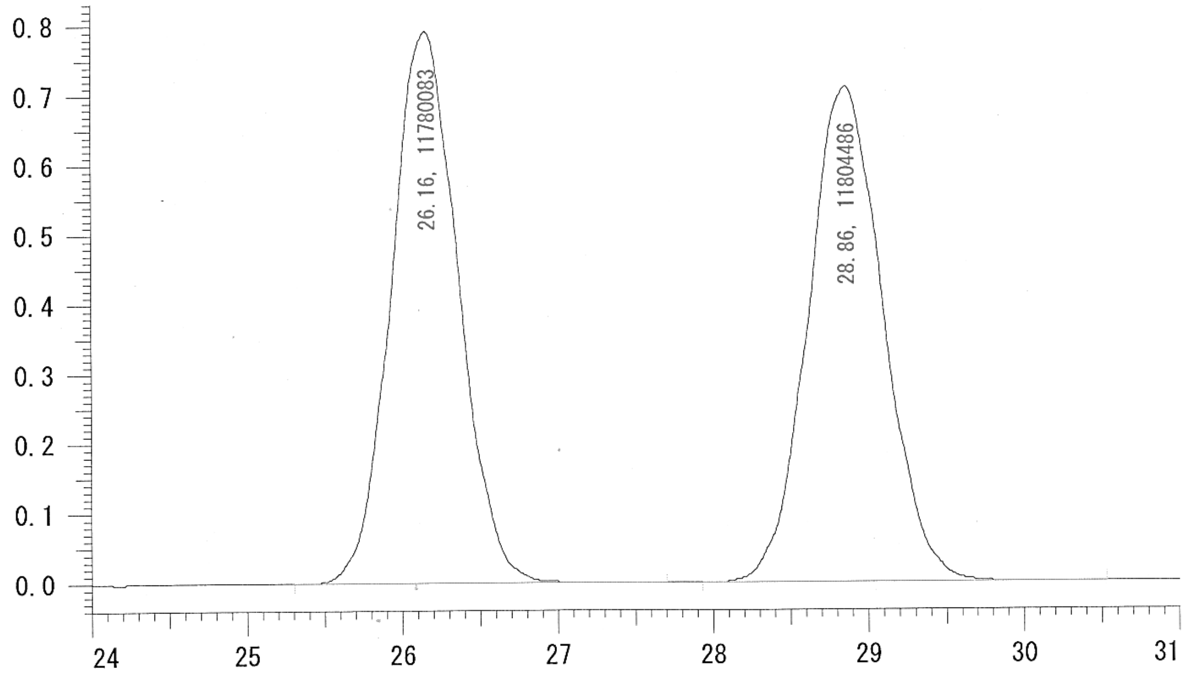

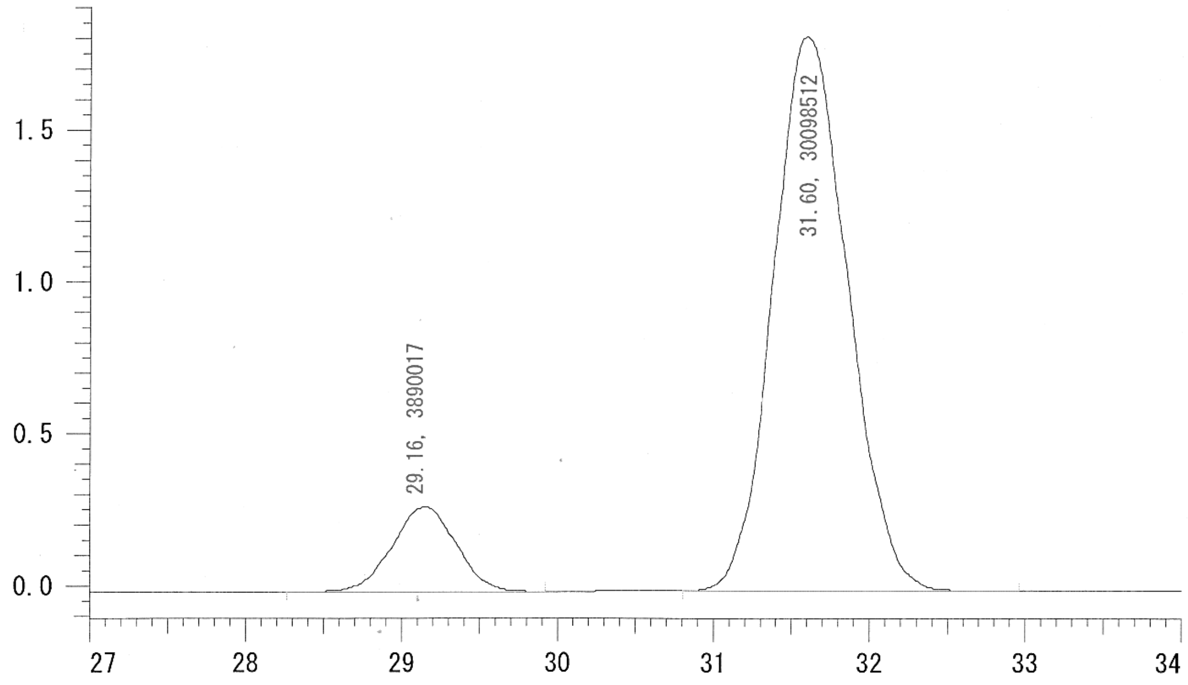

racemic				chiral			
No.	RT (min)	area	area (%)	No.	RT (min)	area	area (%)
1	26.16	11780083	49.948	1	29.16	3890017	11.445
2	28.86	11804486	50.052	2	31.60	30098512	88.555

15.29 3gl

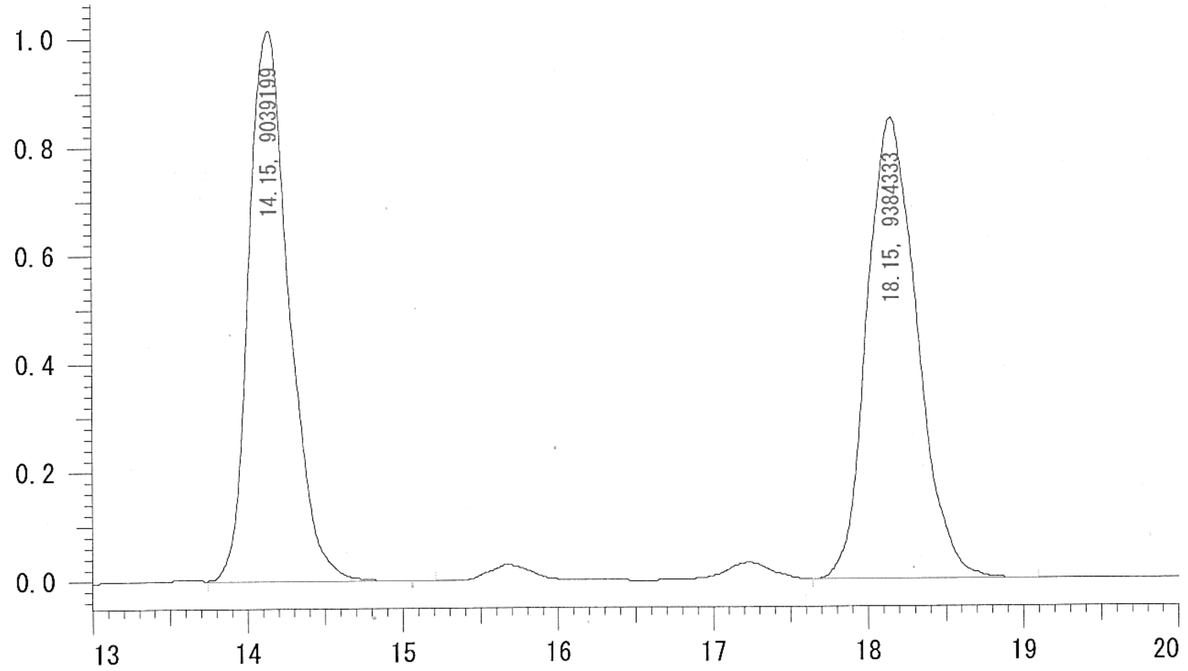

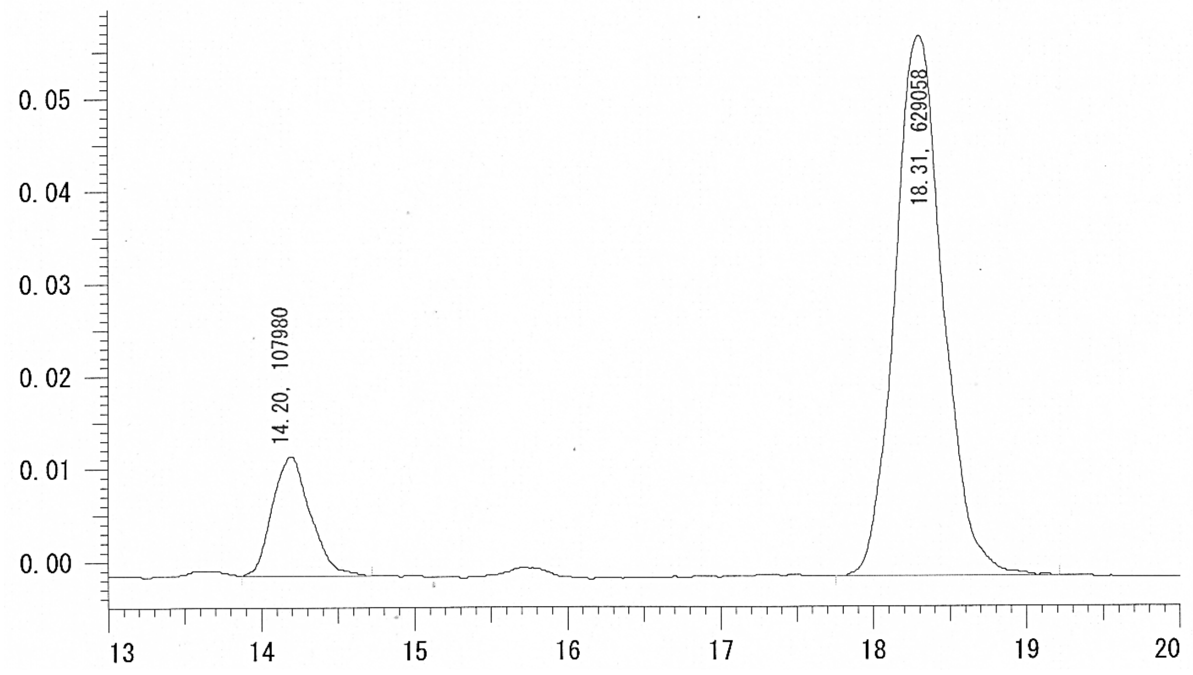

racemic				chiral			
No.	RT (min)	area	area (%)	No.	RT (min)	area	area (%)
1	14.15	9030064	49.038	1	14.20	107980	14.651
2	18.15	9384333	50.962	2	18.31	629058	85.349

15.30 3rm

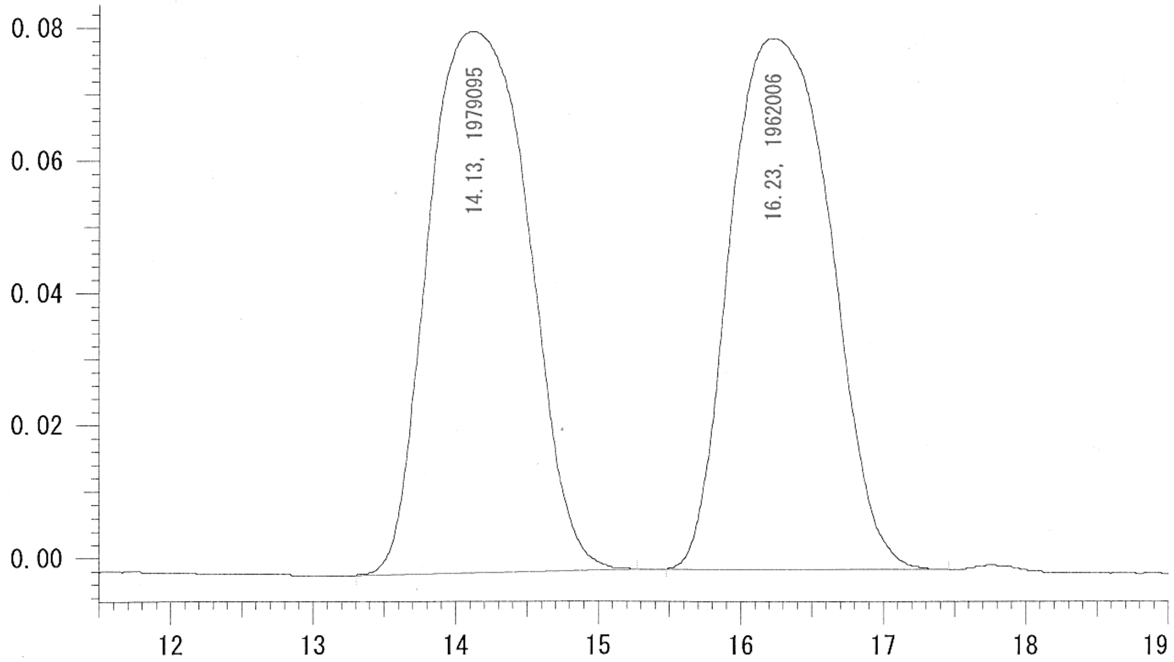

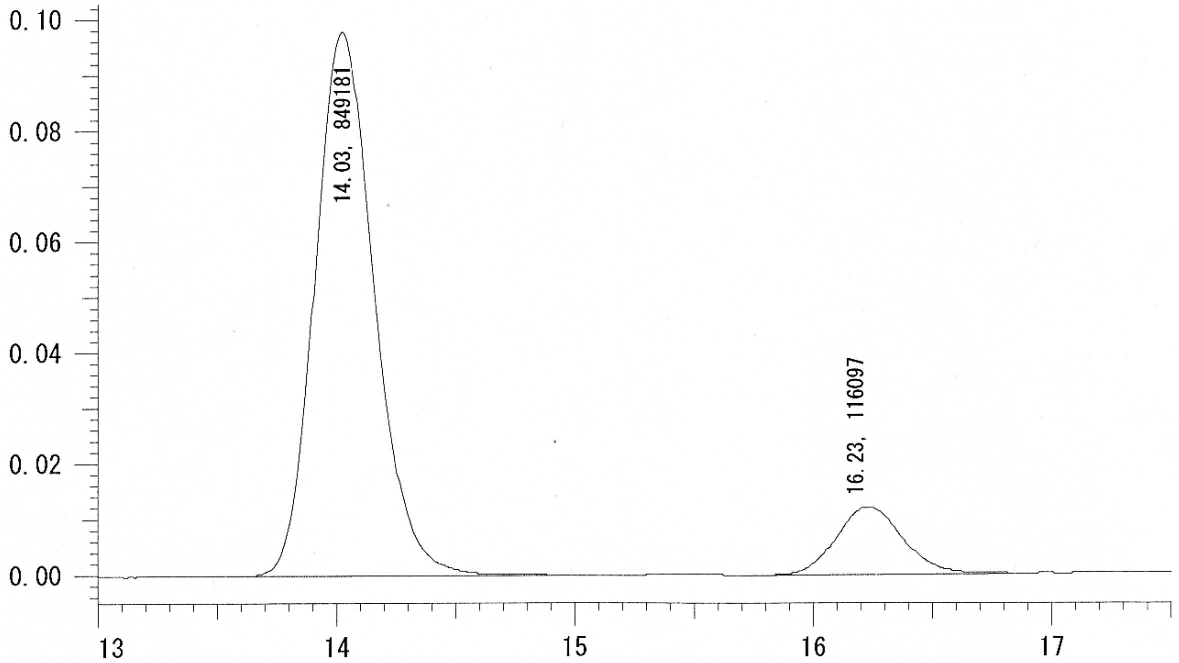

racemic				chiral			
No.	RT (min)	area	area (%)	No.	RT (min)	area	area (%)
1	14.13	1979095	50.217	1	14.03	849181	87.973
2	16.23	1962006	49.783	2	16.23	116097	12.027

16 NMR Spectra

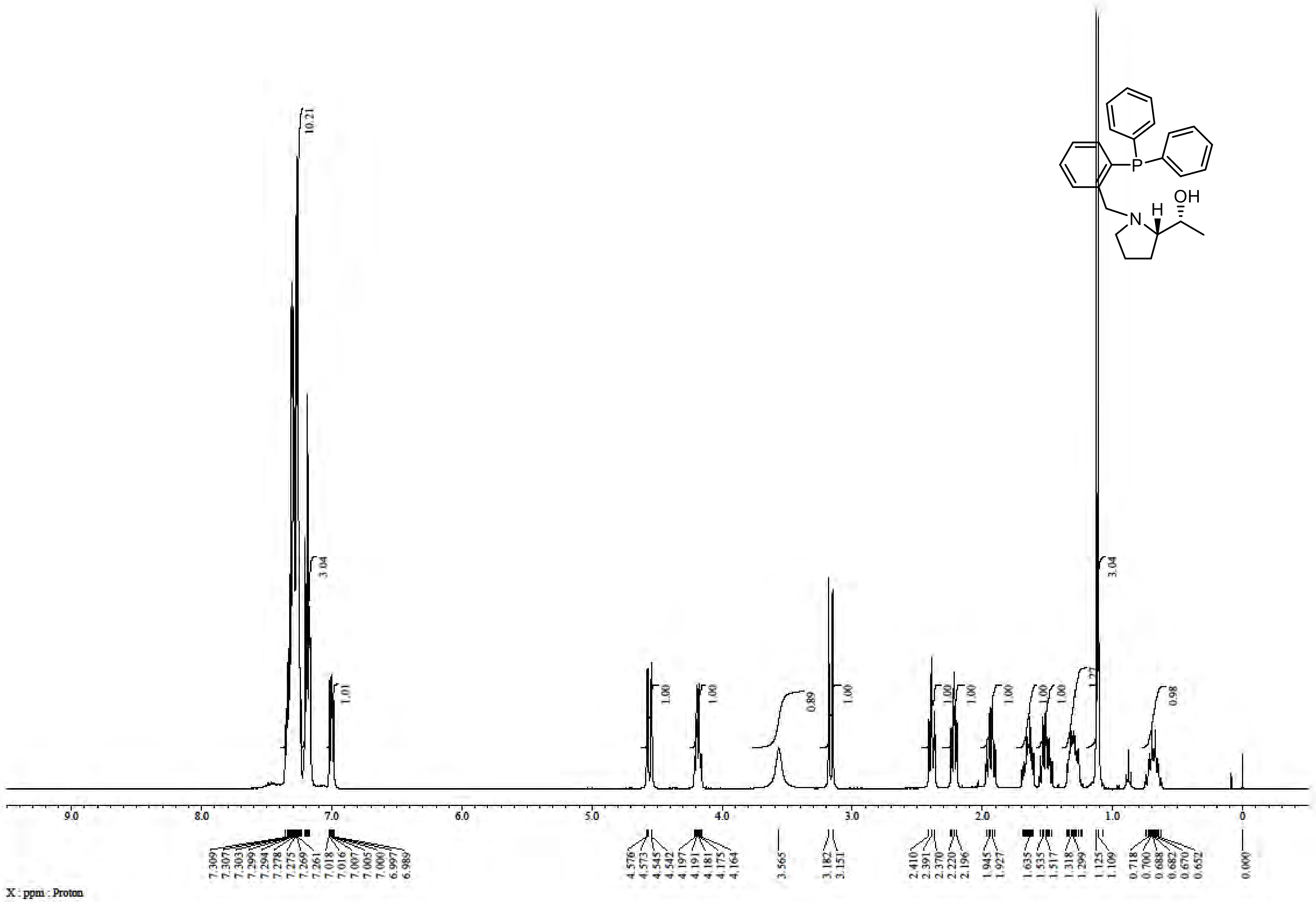

^{1}H NMR spectrum of **L2** in $CDCl_3$

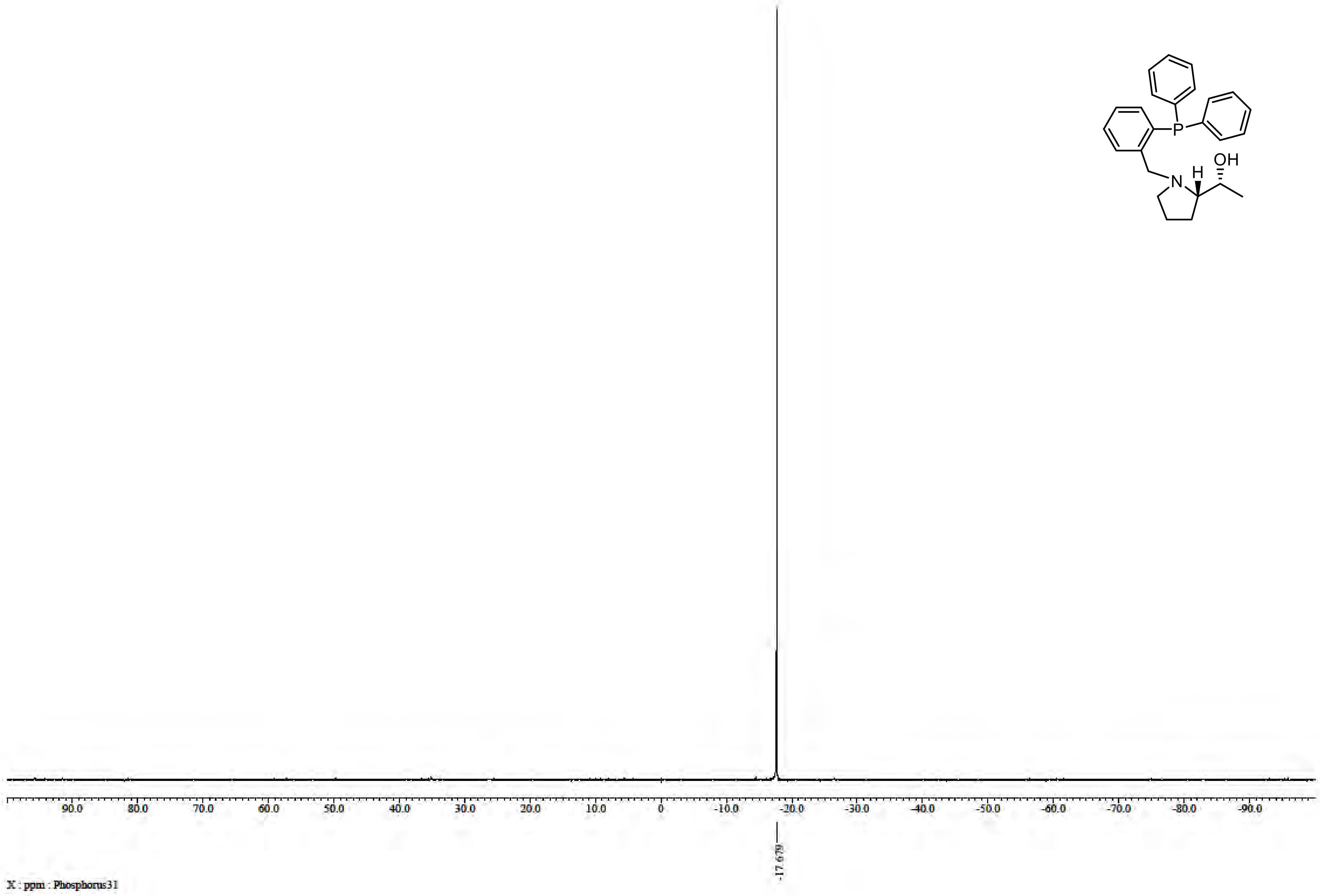

^{31}P NMR spectrum of **L2** in $CDCl_3$

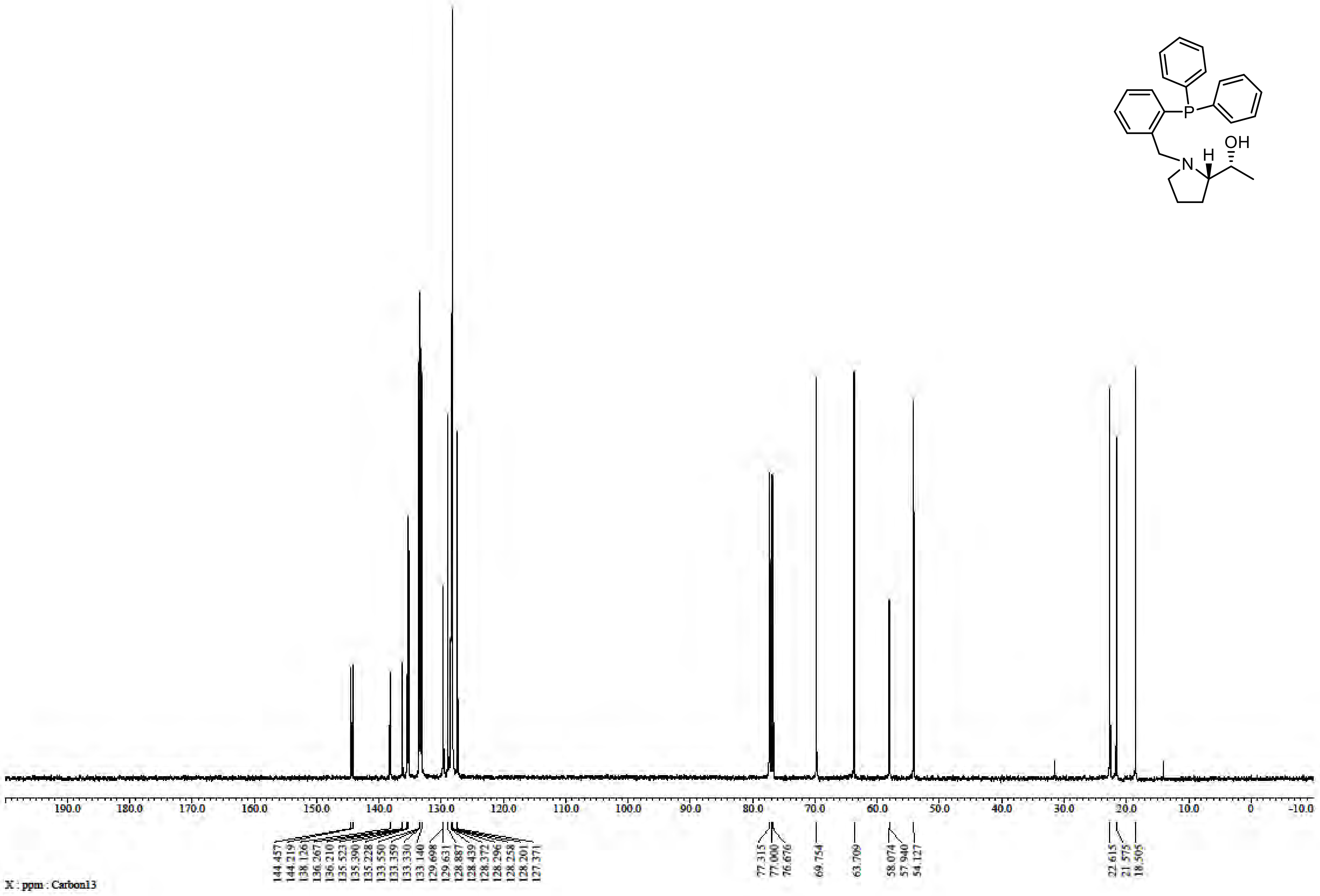

^{13}C NMR spectrum of **L2** in $CDCl_3$

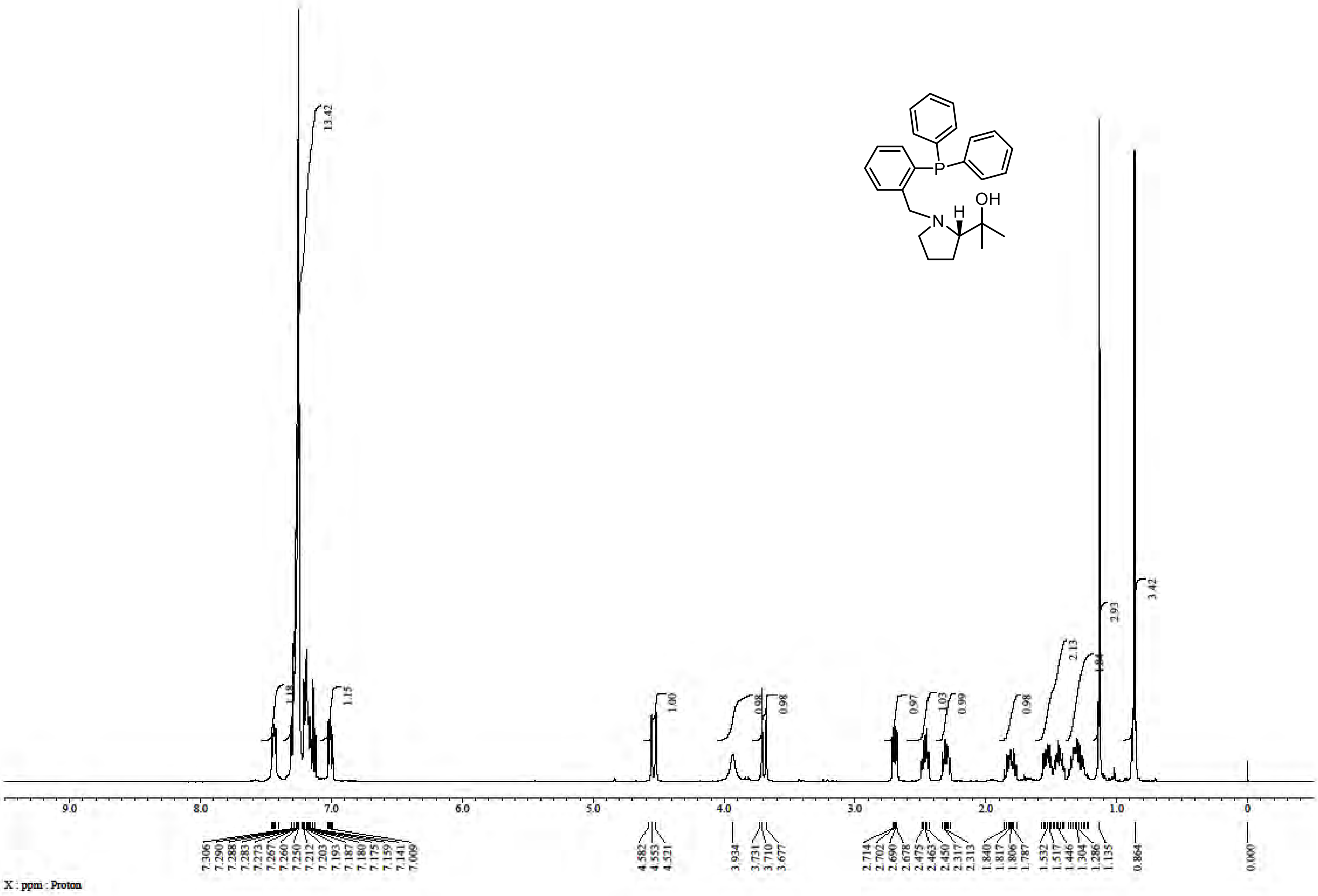

^{1}H NMR spectrum of **L3** in $CDCl_3$

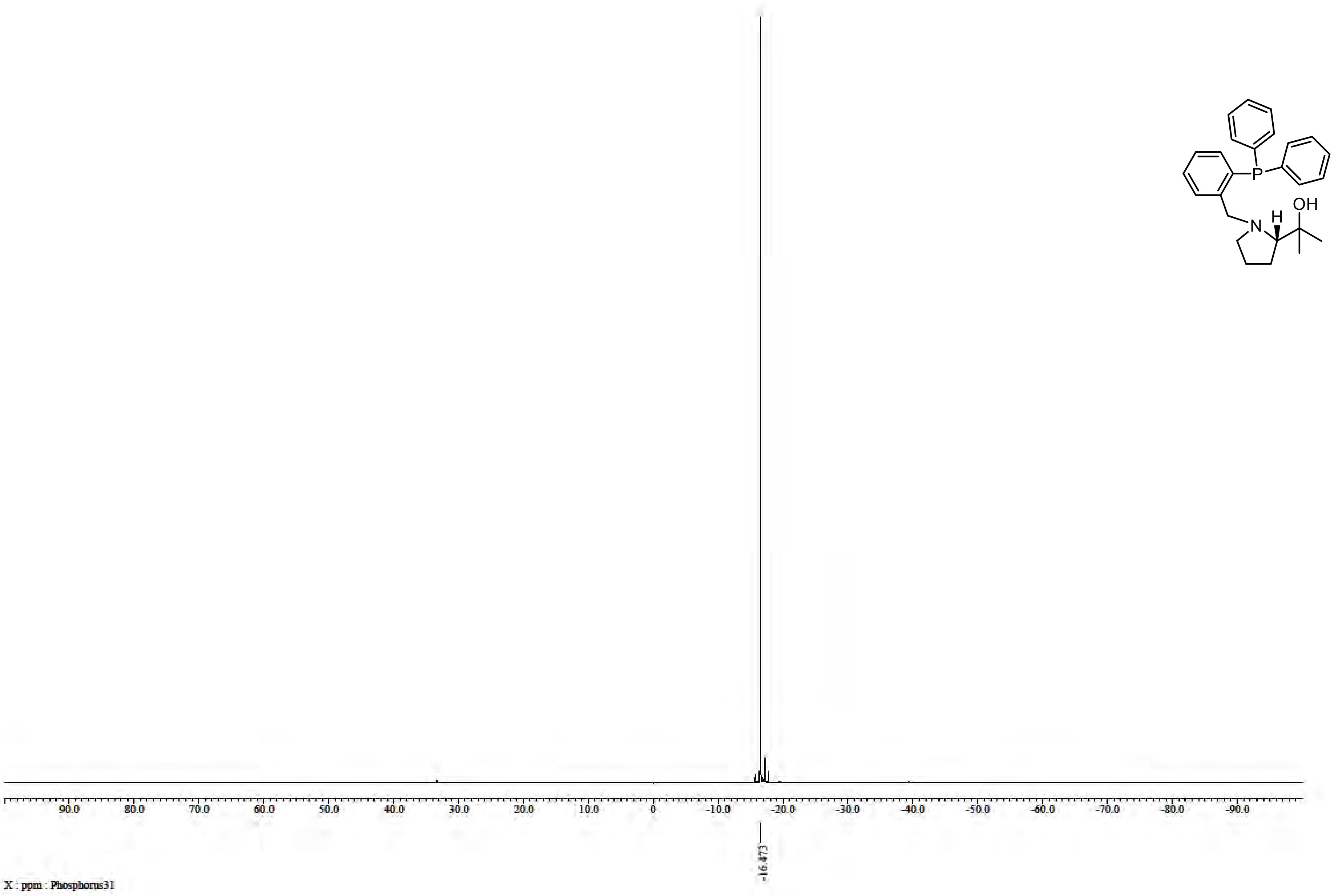

^{31}P NMR spectrum of **L3** in $CDCl_3$

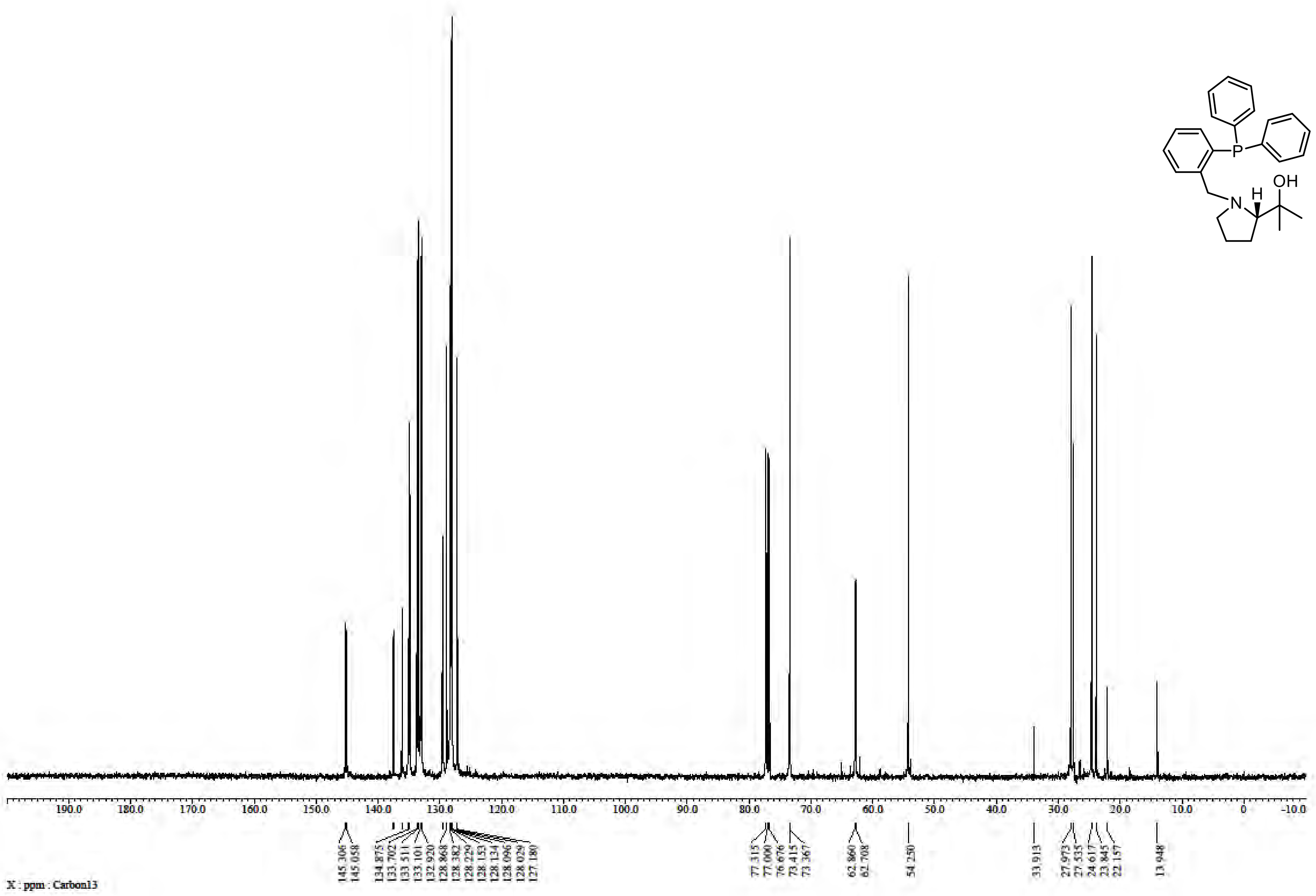

^{13}C NMR spectrum of **L3** in $CDCl_3$

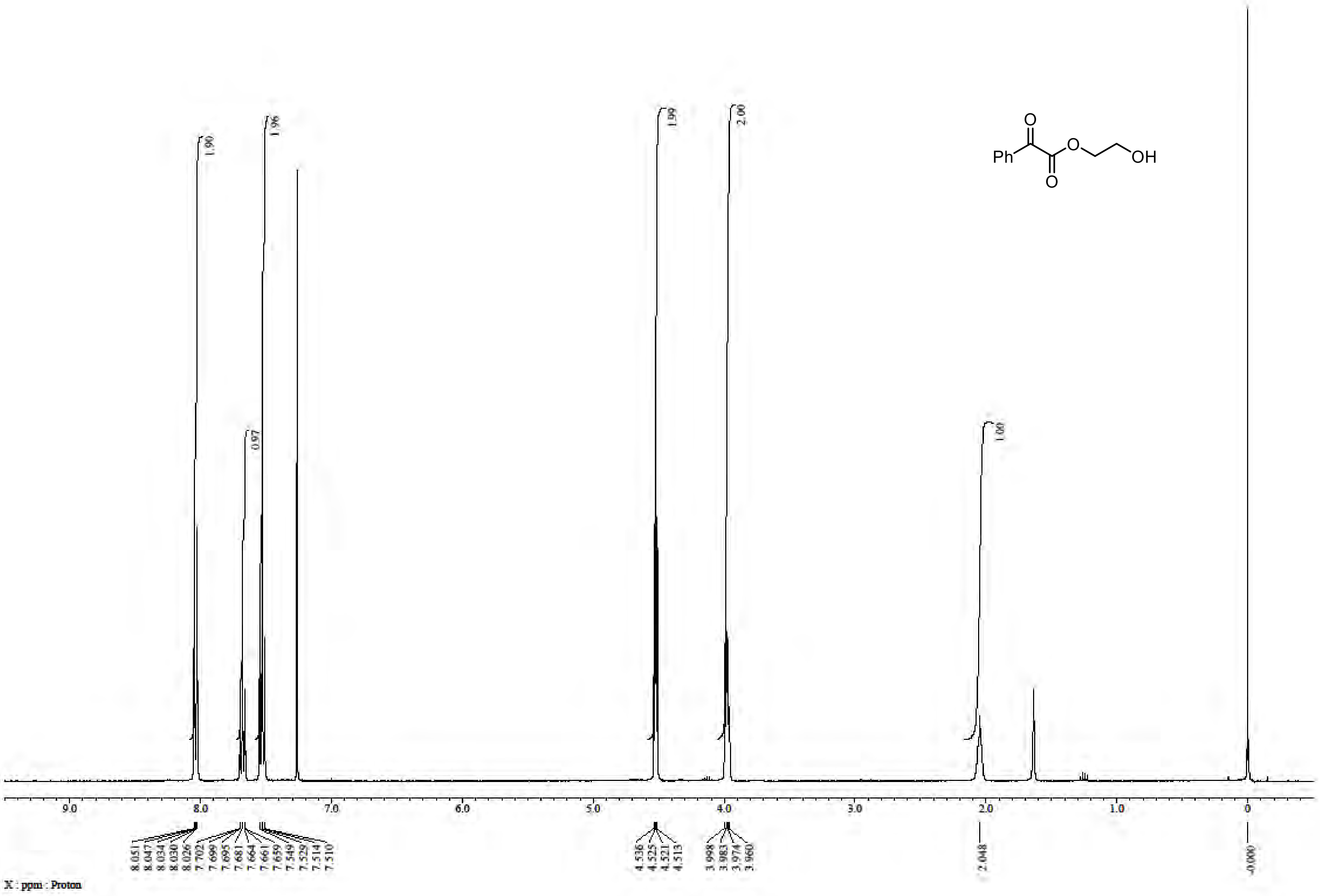

^{1}H NMR spectrum of **1d** in $CDCl_3$

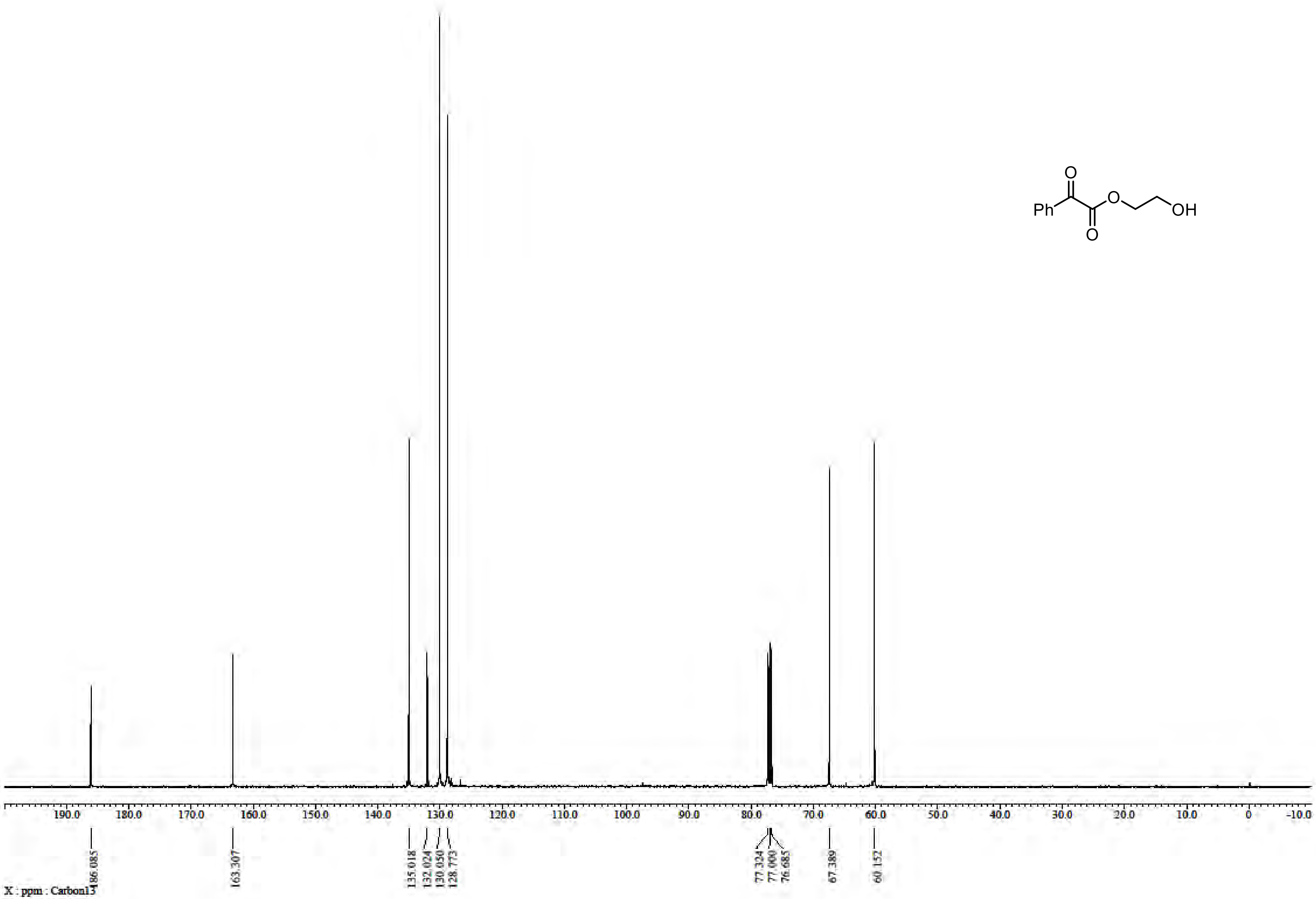

^{13}C NMR spectrum of **1d** in $CDCl_3$

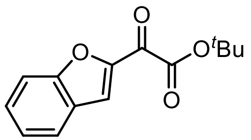

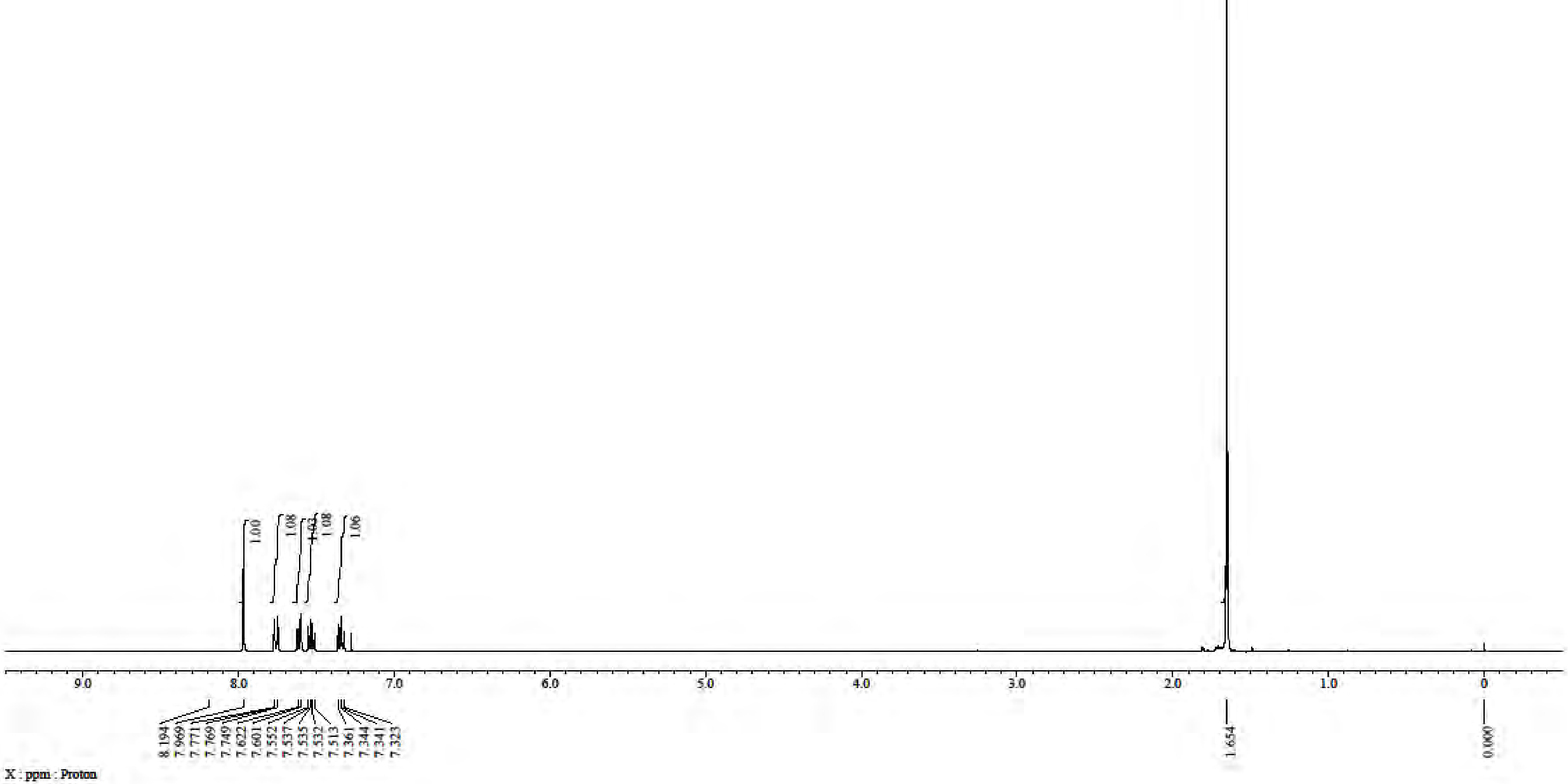

^{1}H NMR spectrum of **1m** in $CDCl_3$

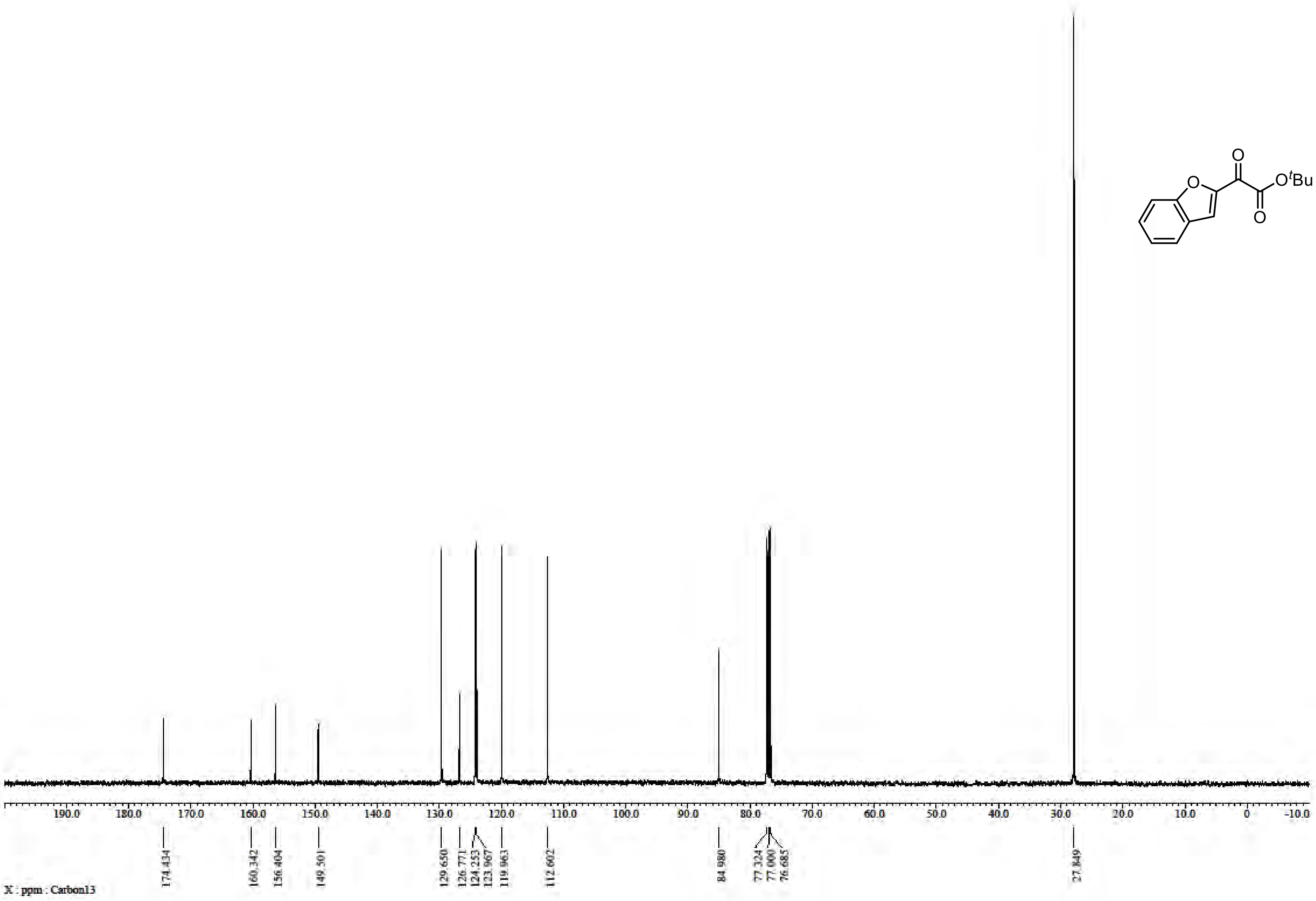

^{13}C NMR spectrum of **1m** in $CDCl_3$

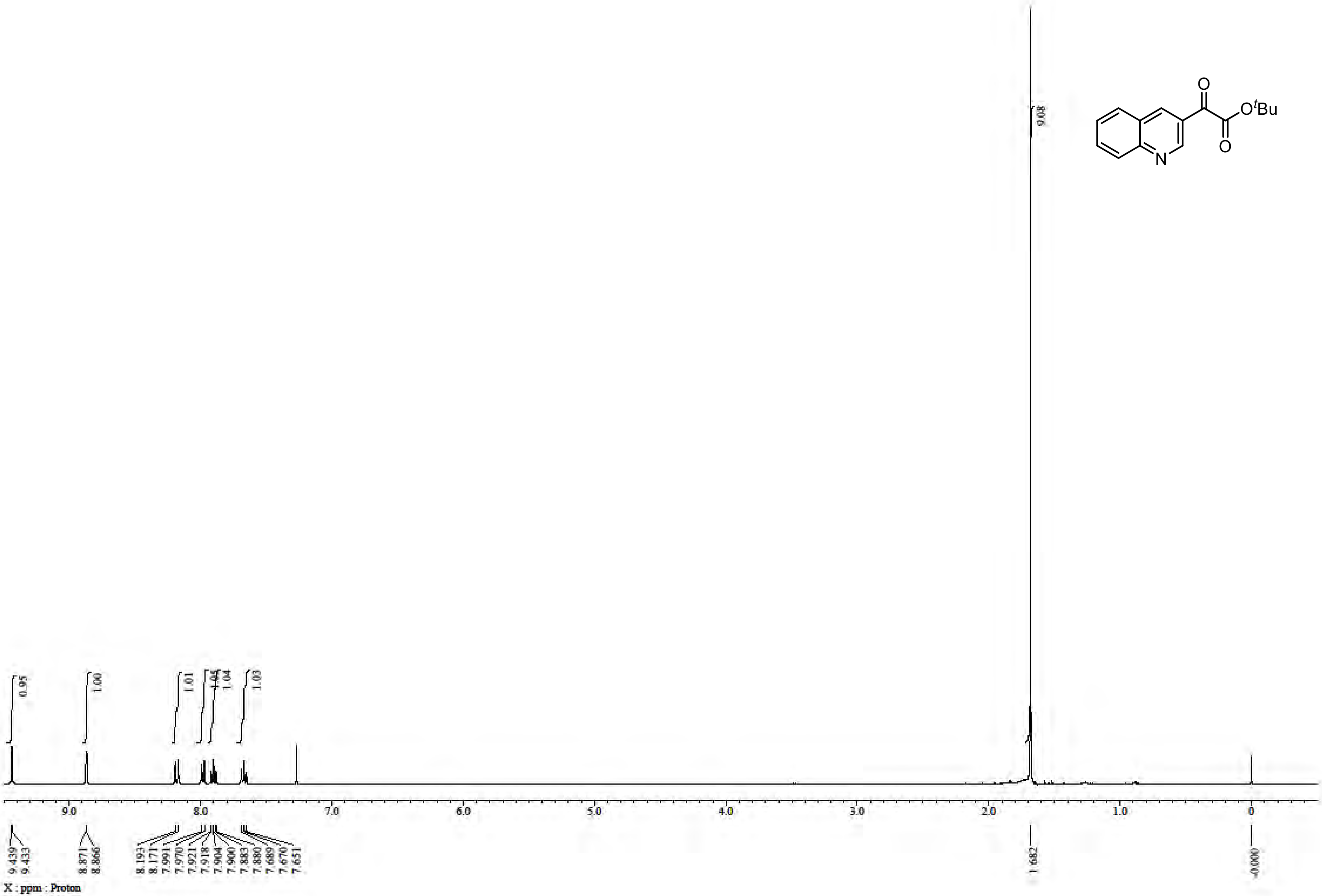

^{1}H NMR spectrum of **1o** in $CDCl_3$

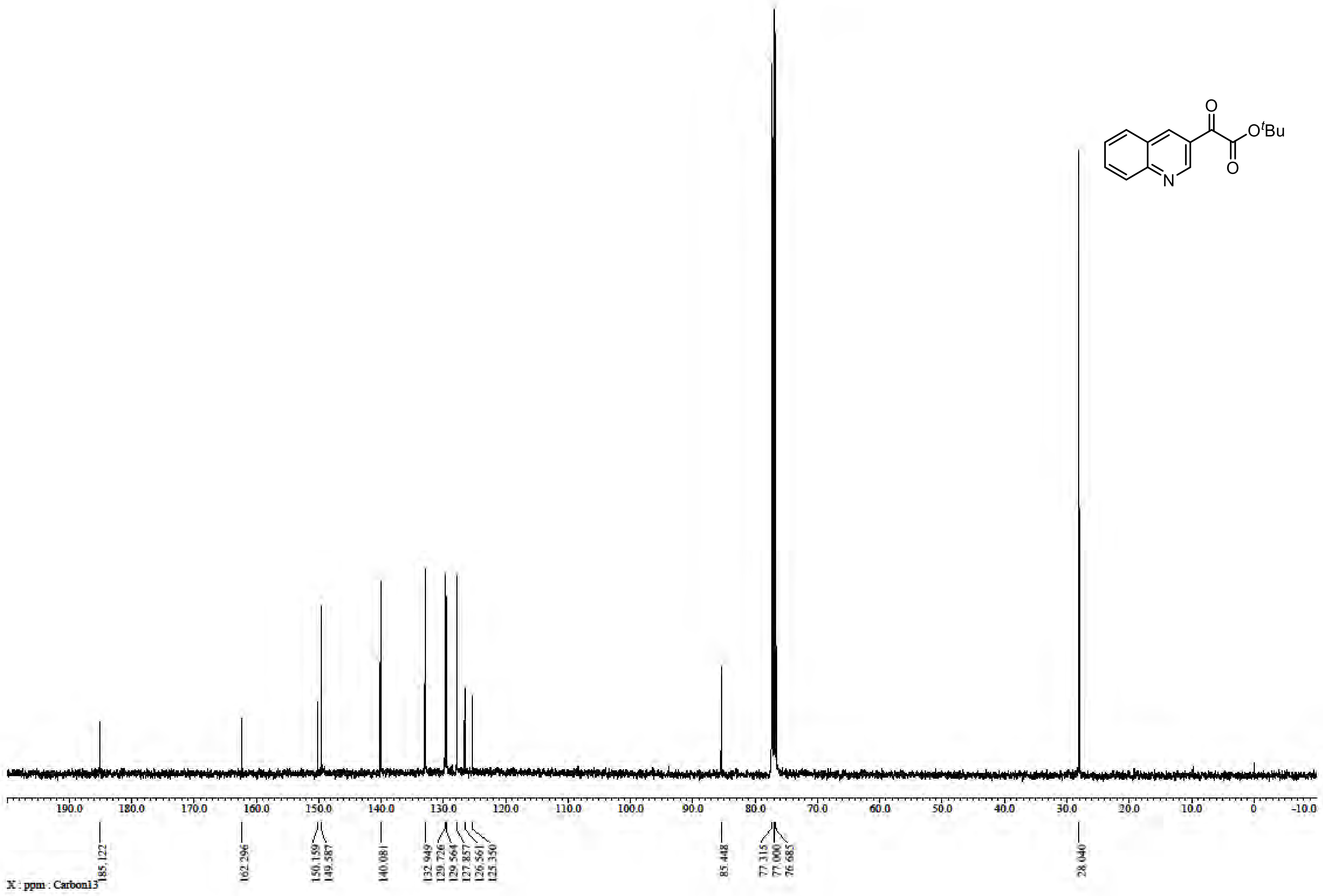

^{13}C NMR spectrum of **1o** in $CDCl_3$

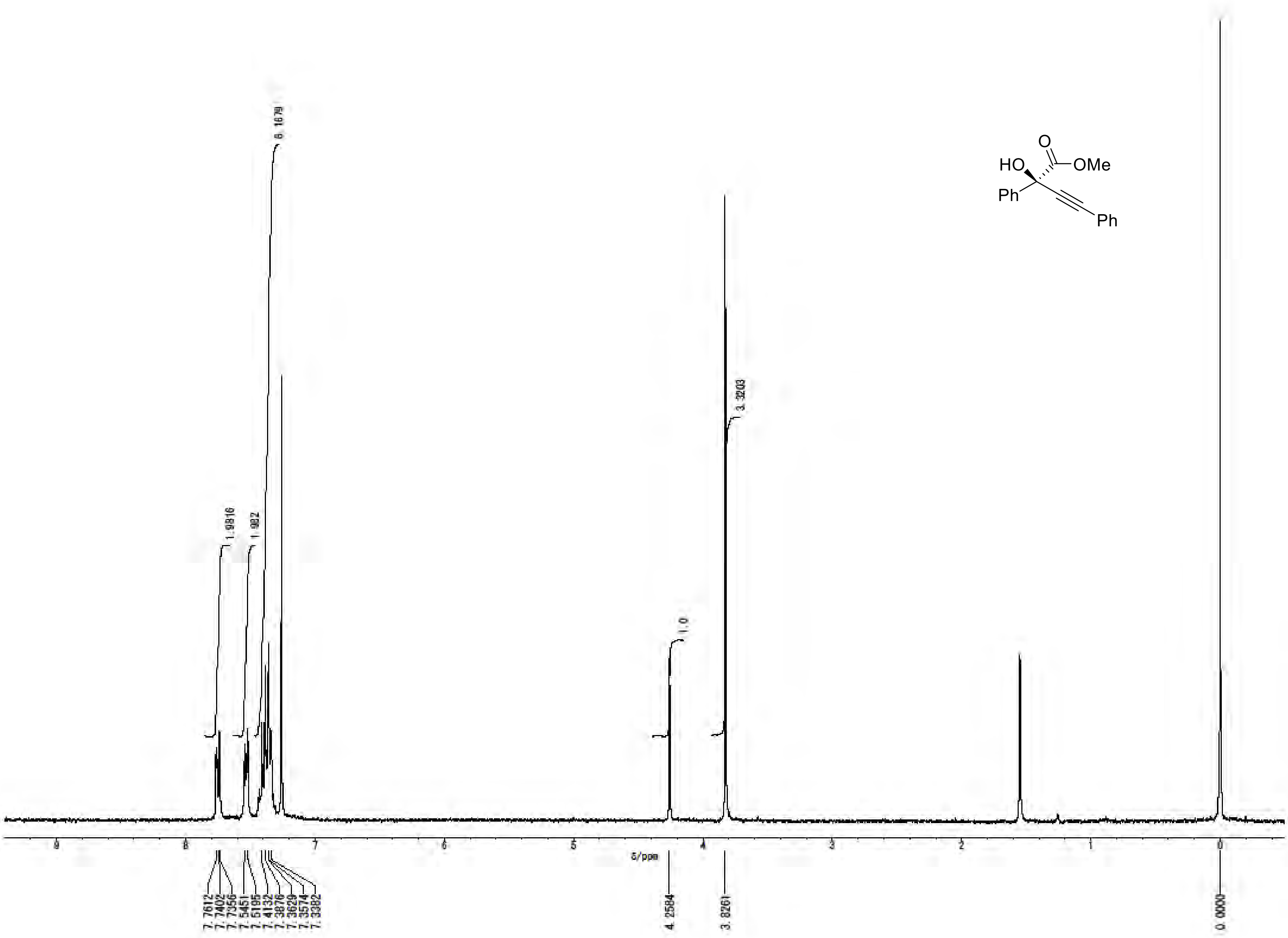

^{1}H NMR spectrum of **3aa** in $CDCl_3$

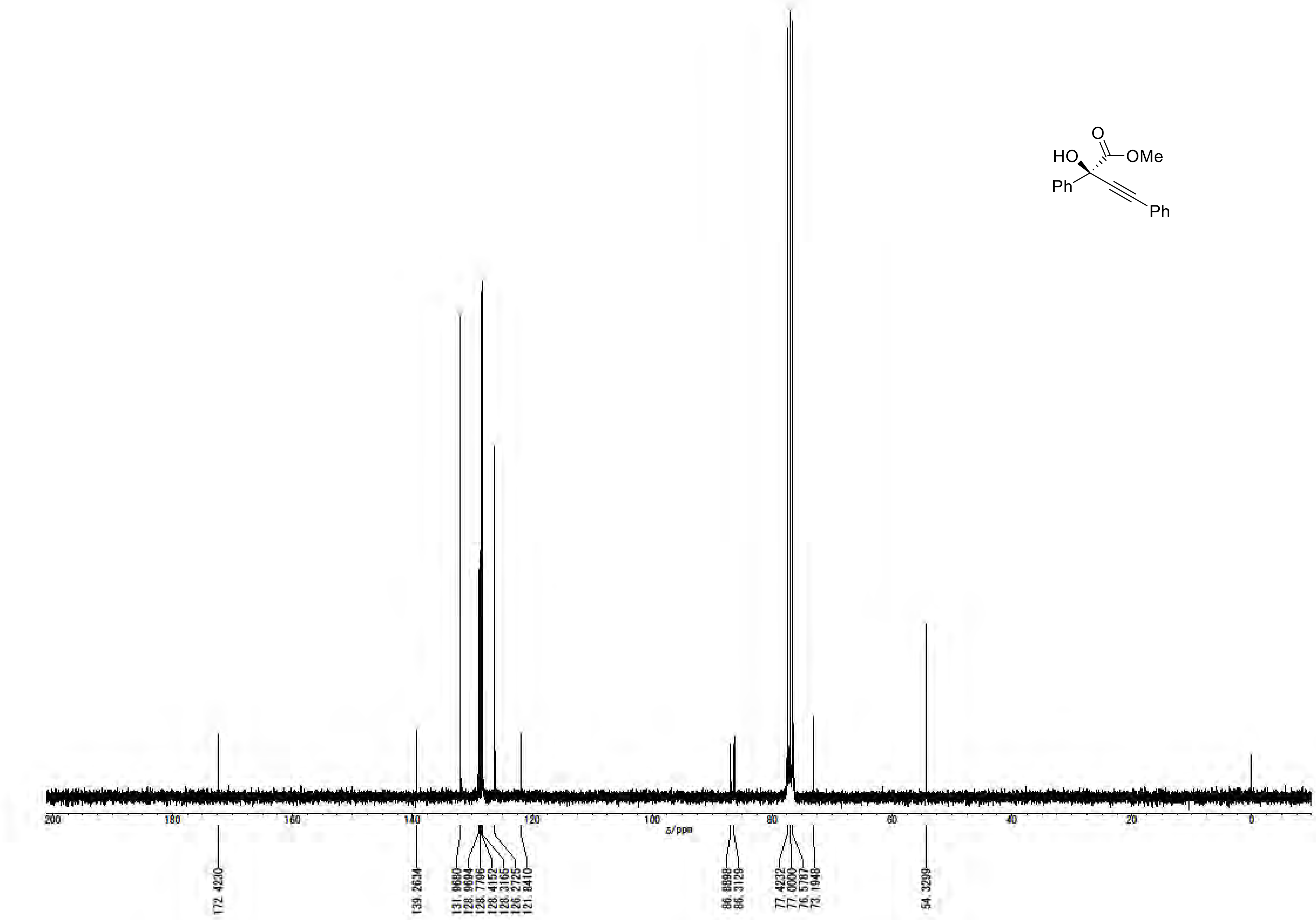

^{13}C NMR spectrum of **3aa** in $CDCl_3$

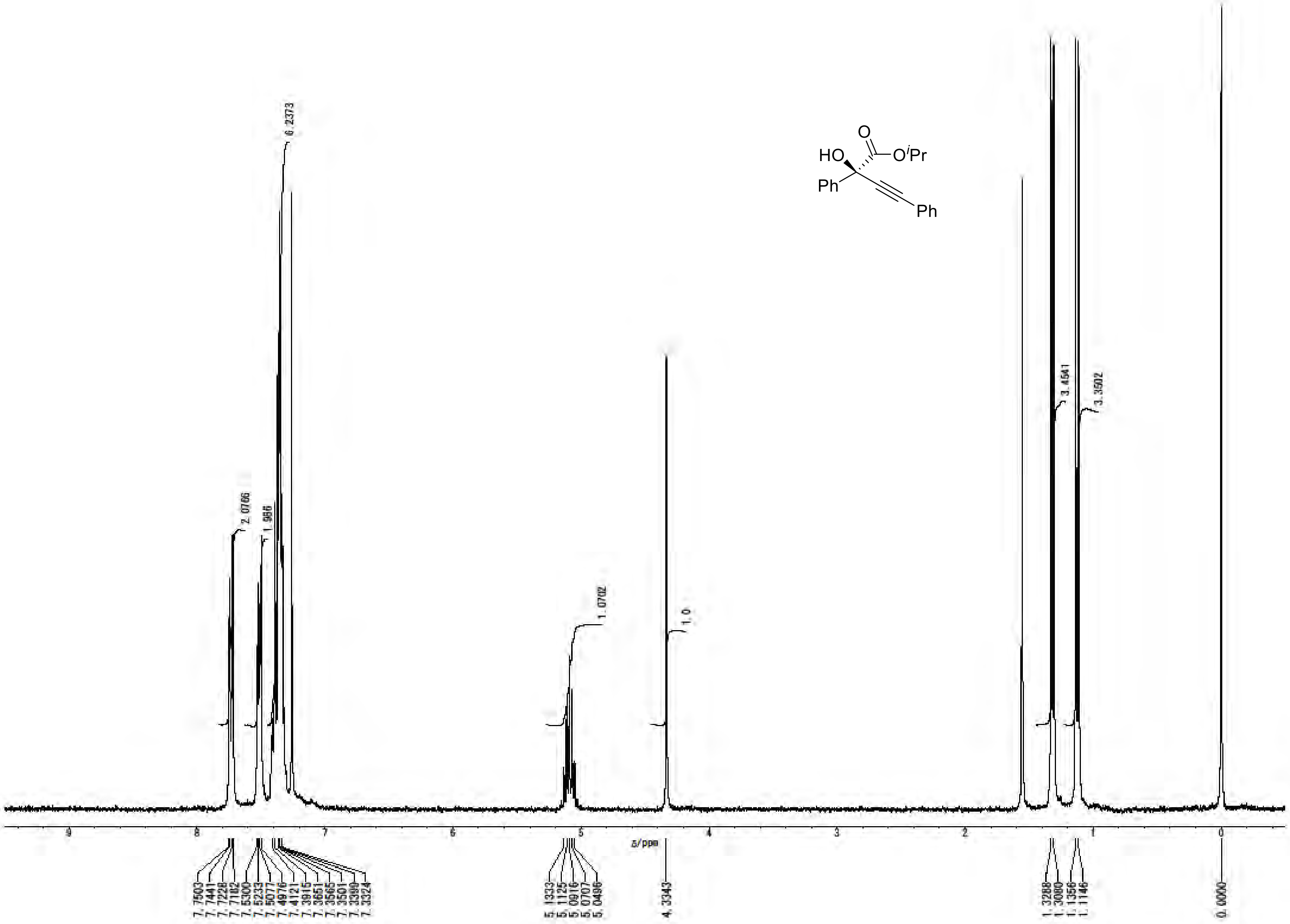

^{1}H NMR spectrum of **3ba** in $CDCl_3$

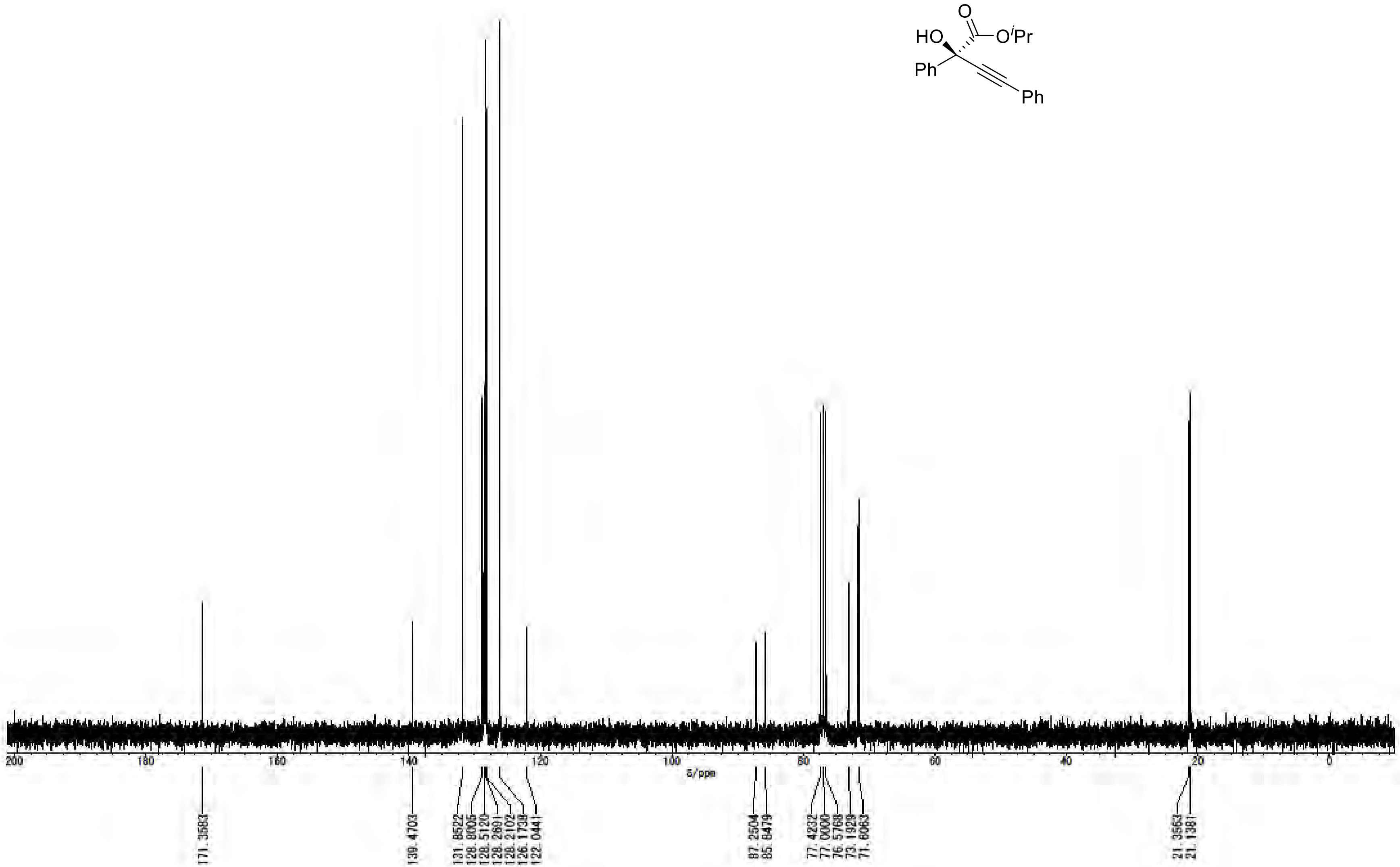

^{13}C NMR spectrum of **3ba** in $CDCl_3$

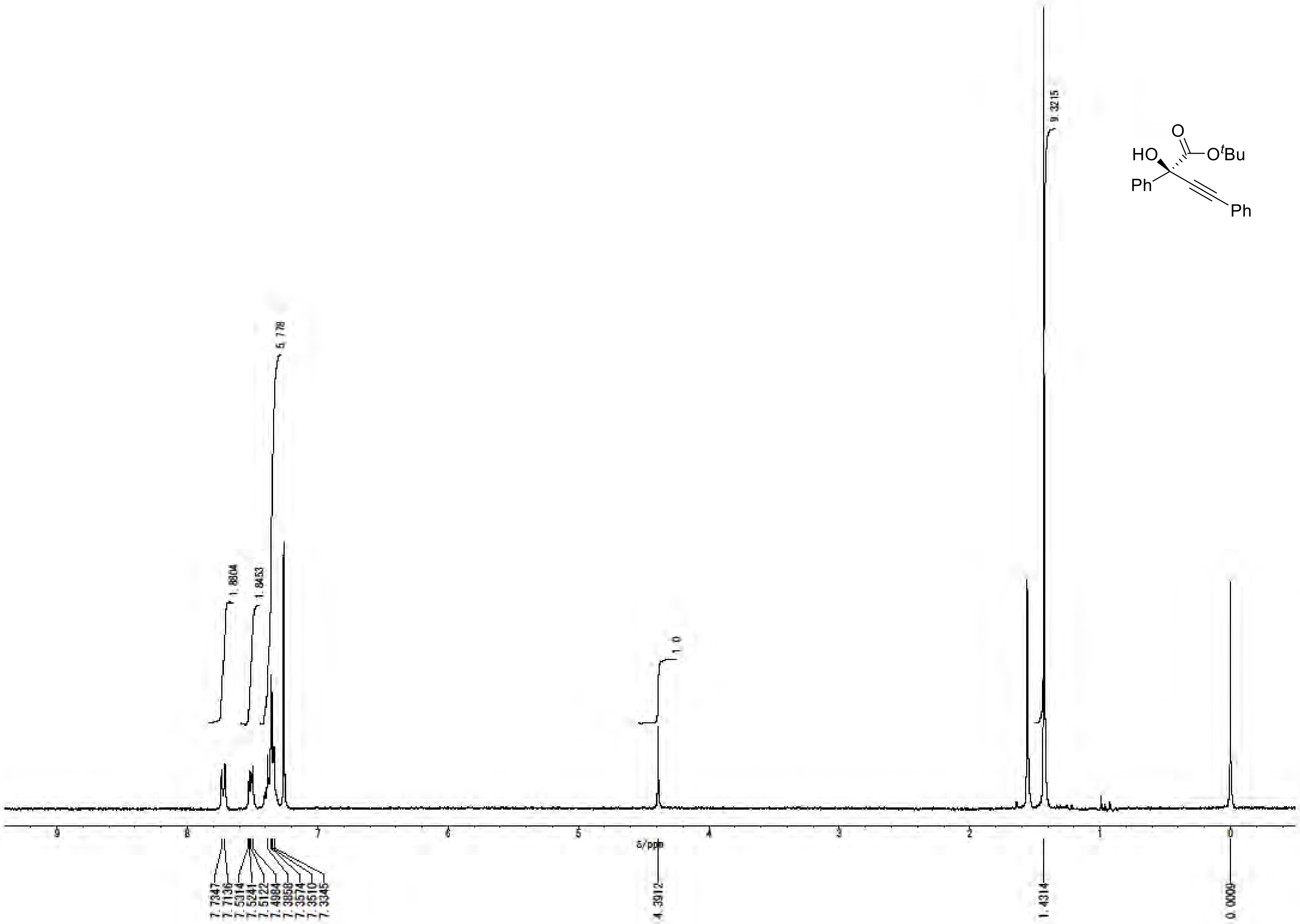

^{1}H NMR spectrum of **3ca** in $CDCl_3$

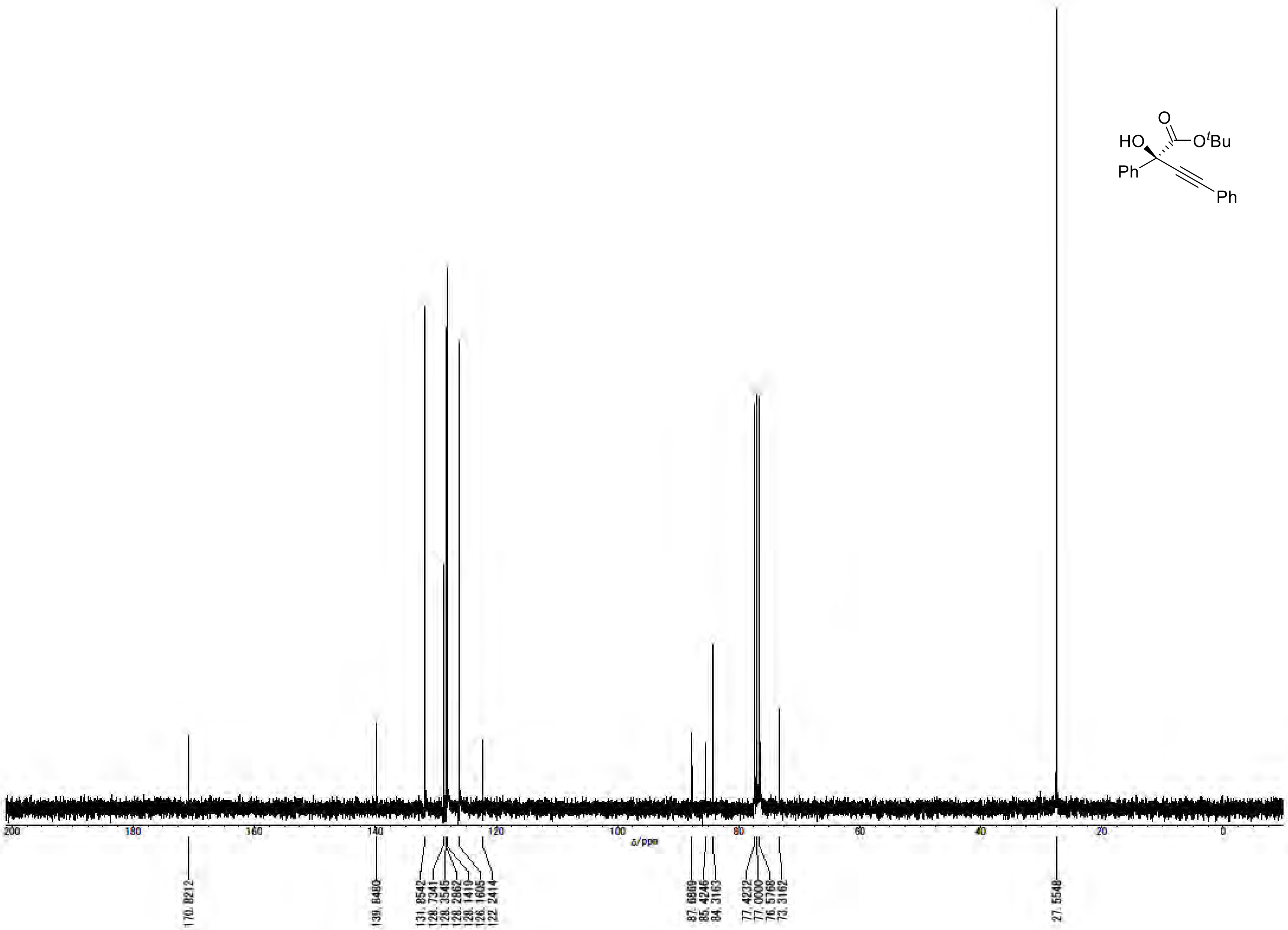

^{13}C NMR spectrum of **3ca** in $CDCl_3$

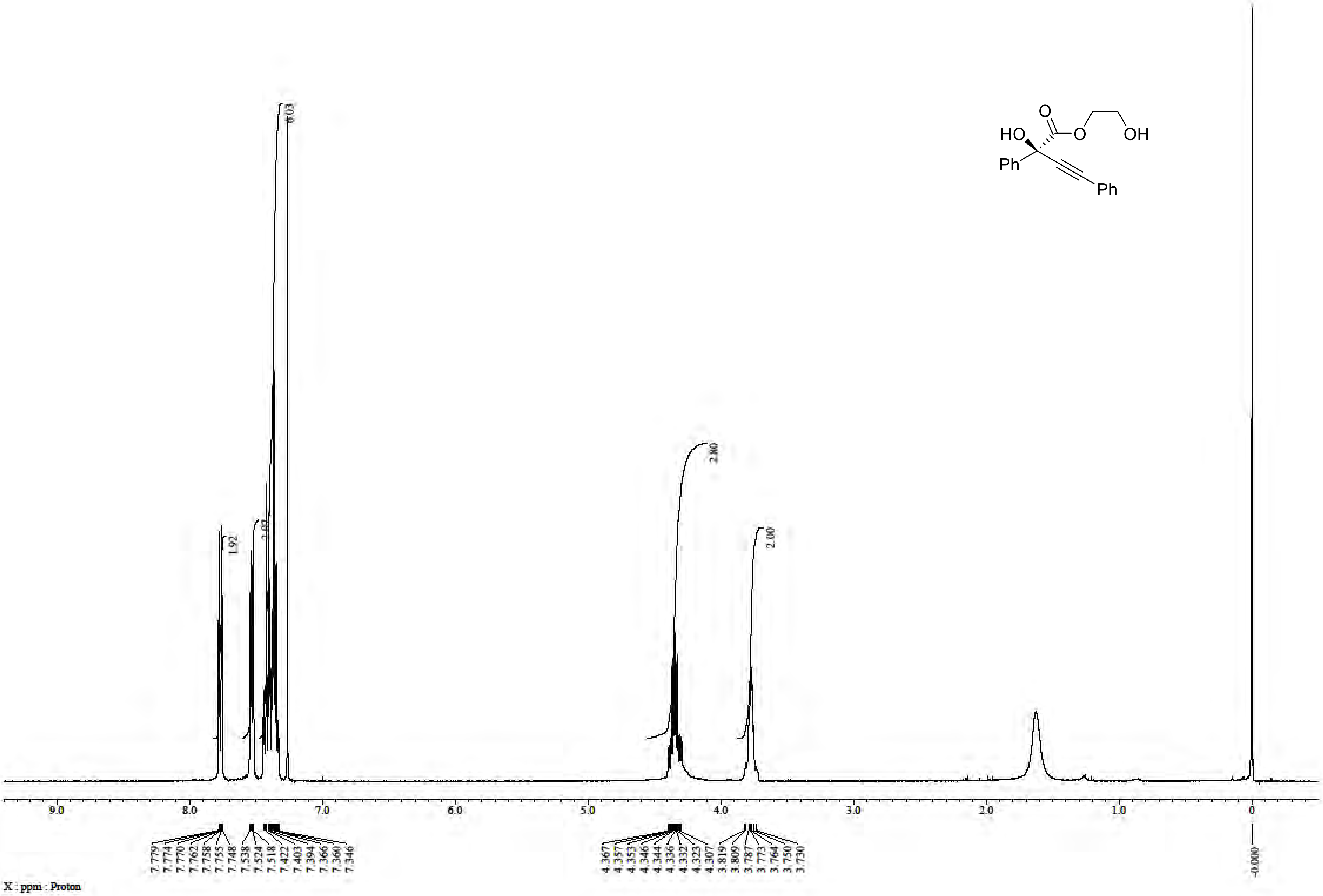

^{1}H NMR spectrum of 3**da** in $CDCl_3$

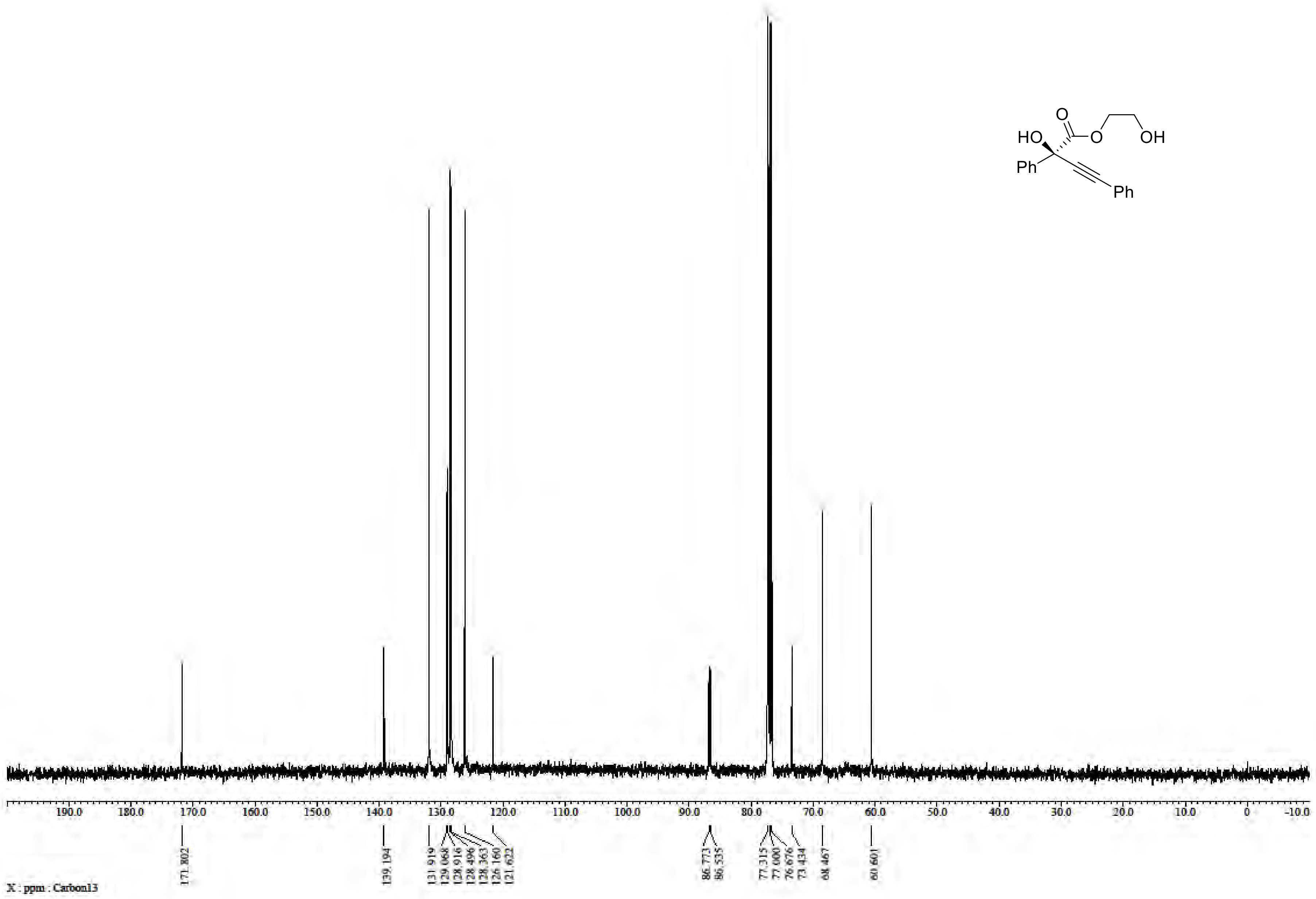

^{13}C NMR spectrum of **3da** in $CDCl_3$

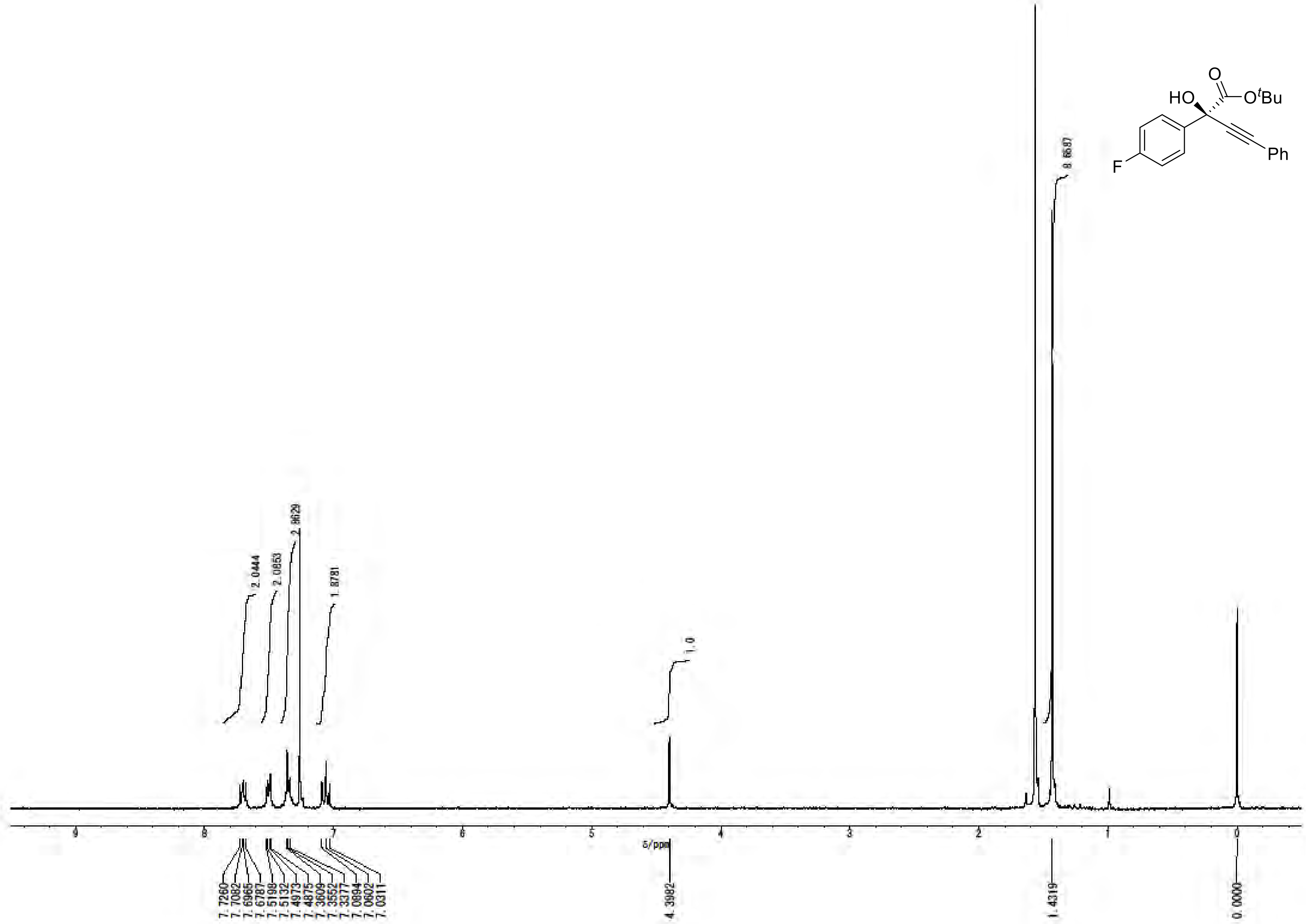

^{1}H NMR spectrum of **3ea** in $CDCl_3$

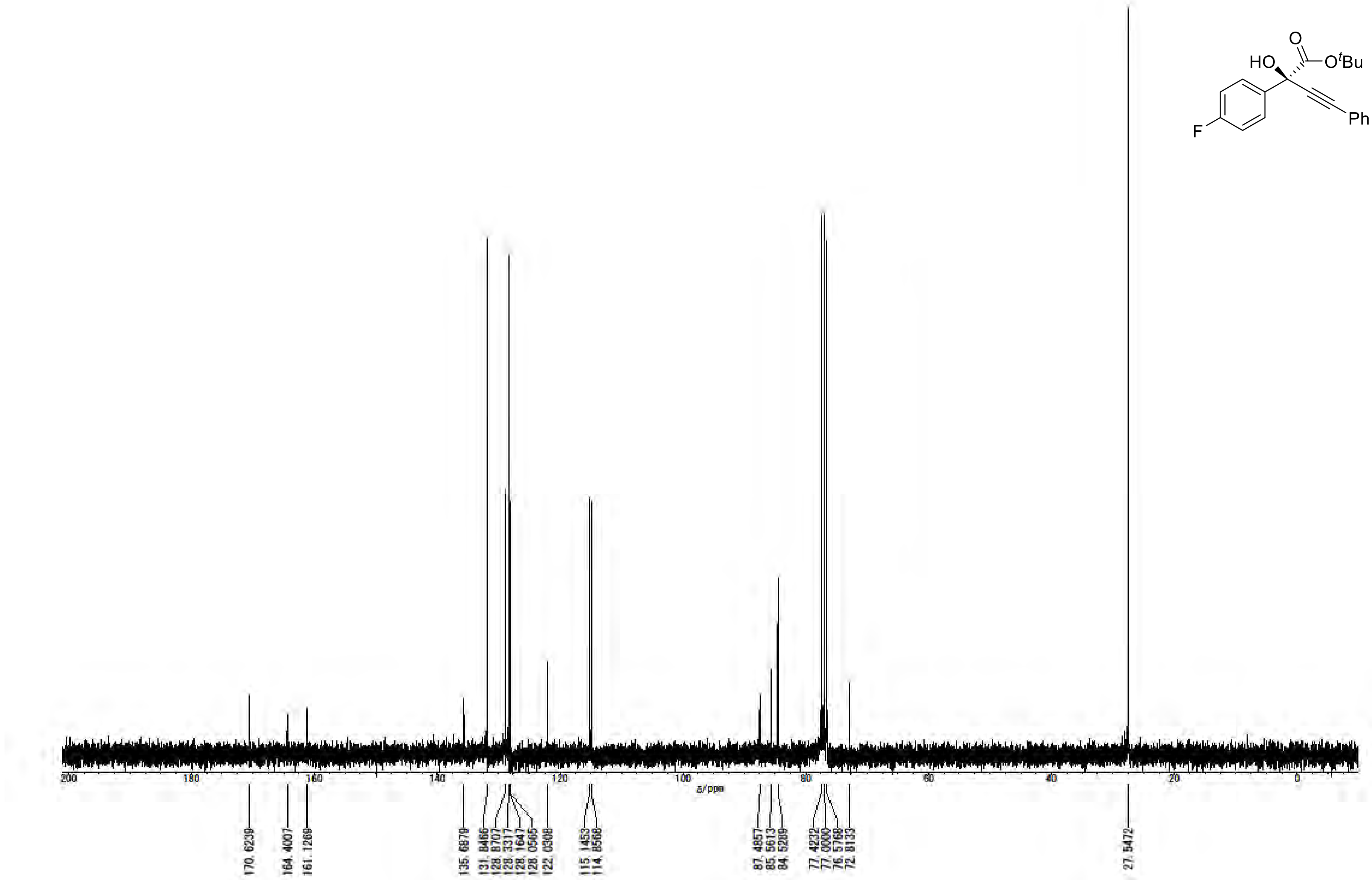

^{13}C NMR spectrum of 3**ea** in $CDCl_3$

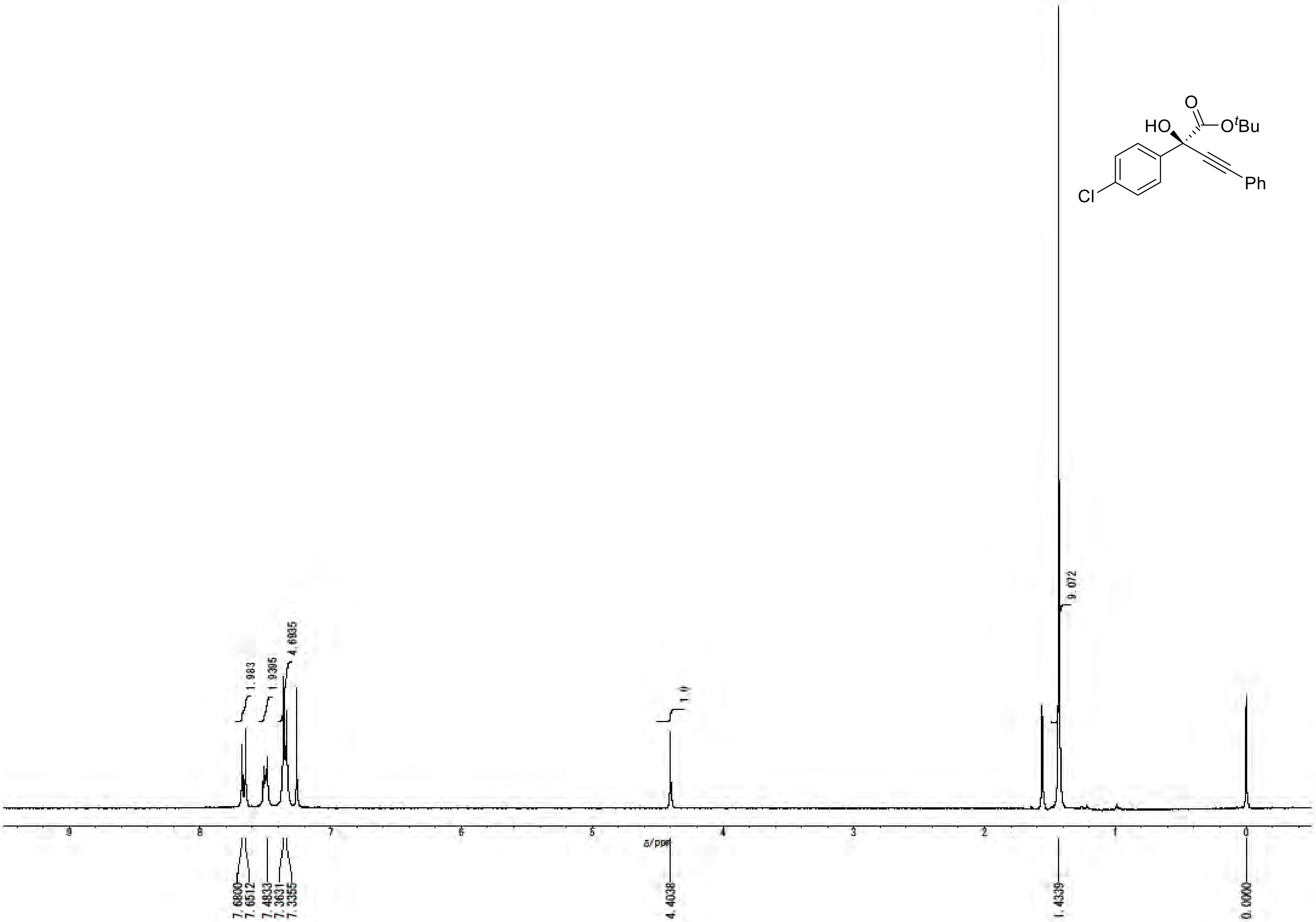

^{1}H NMR spectrum of **3fa** in $CDCl_3$

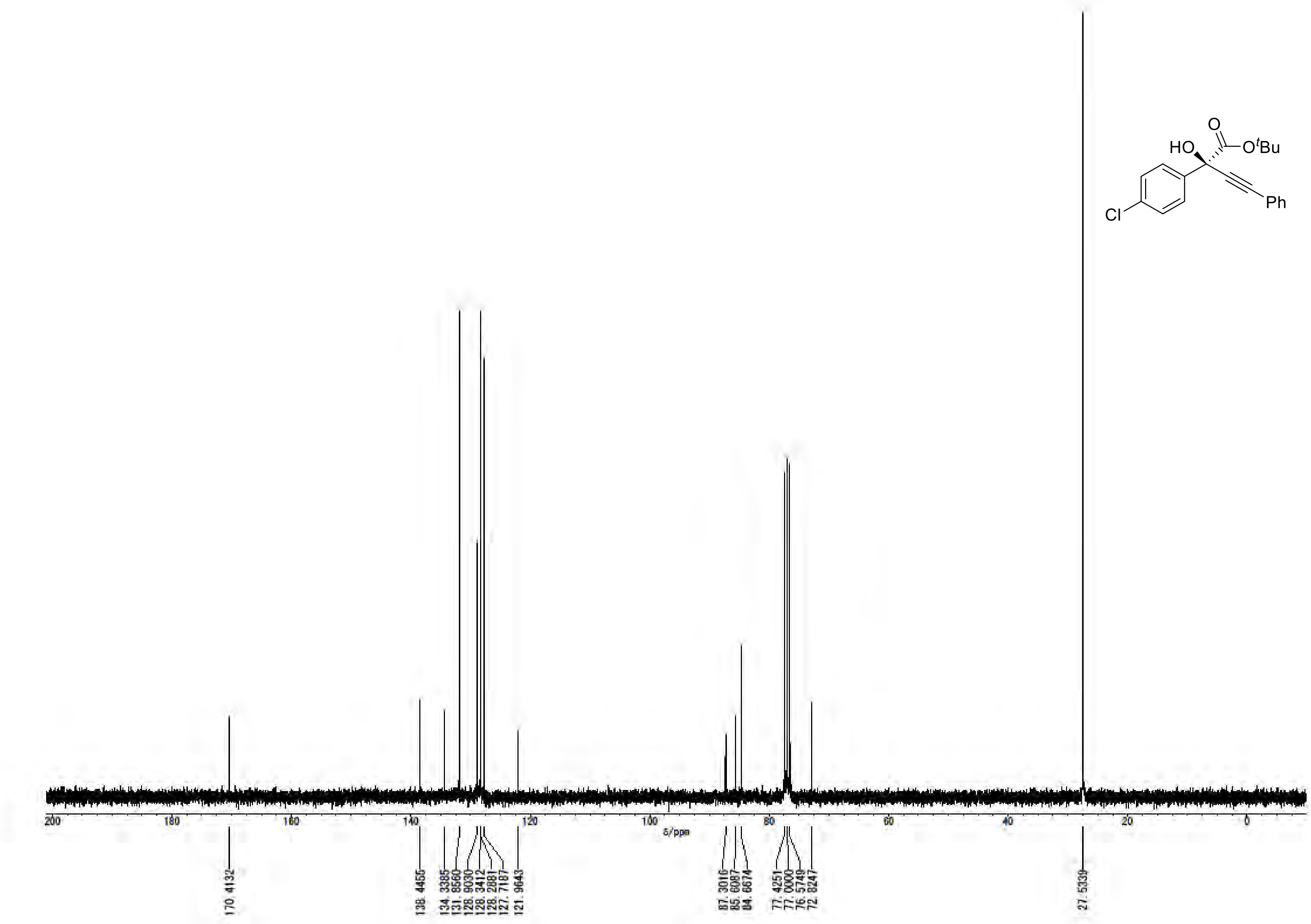

^{13}C NMR spectrum of **3fa** in $CDCl_3$

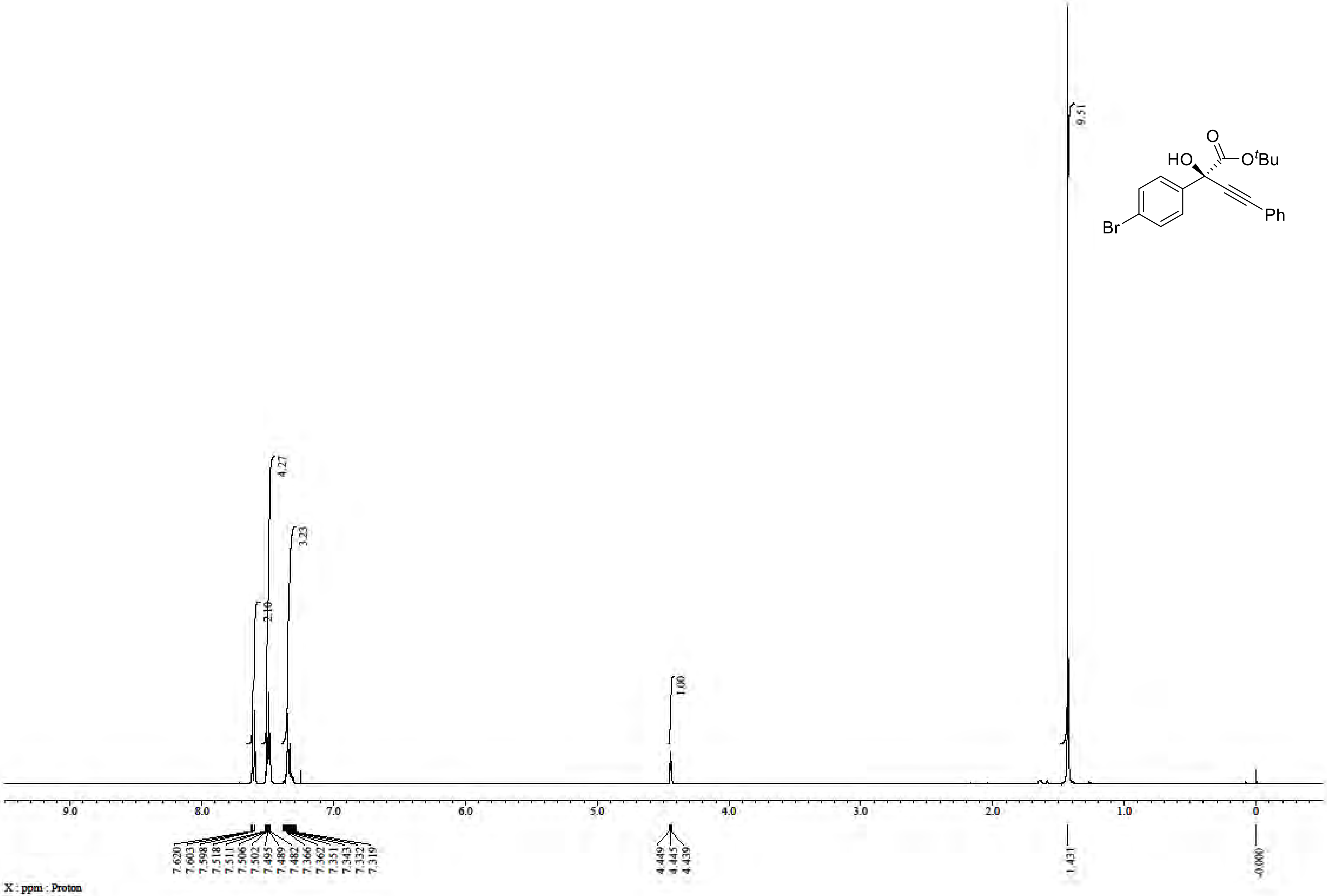

^{1}H NMR spectrum of **3ga** in $CDCl_3$

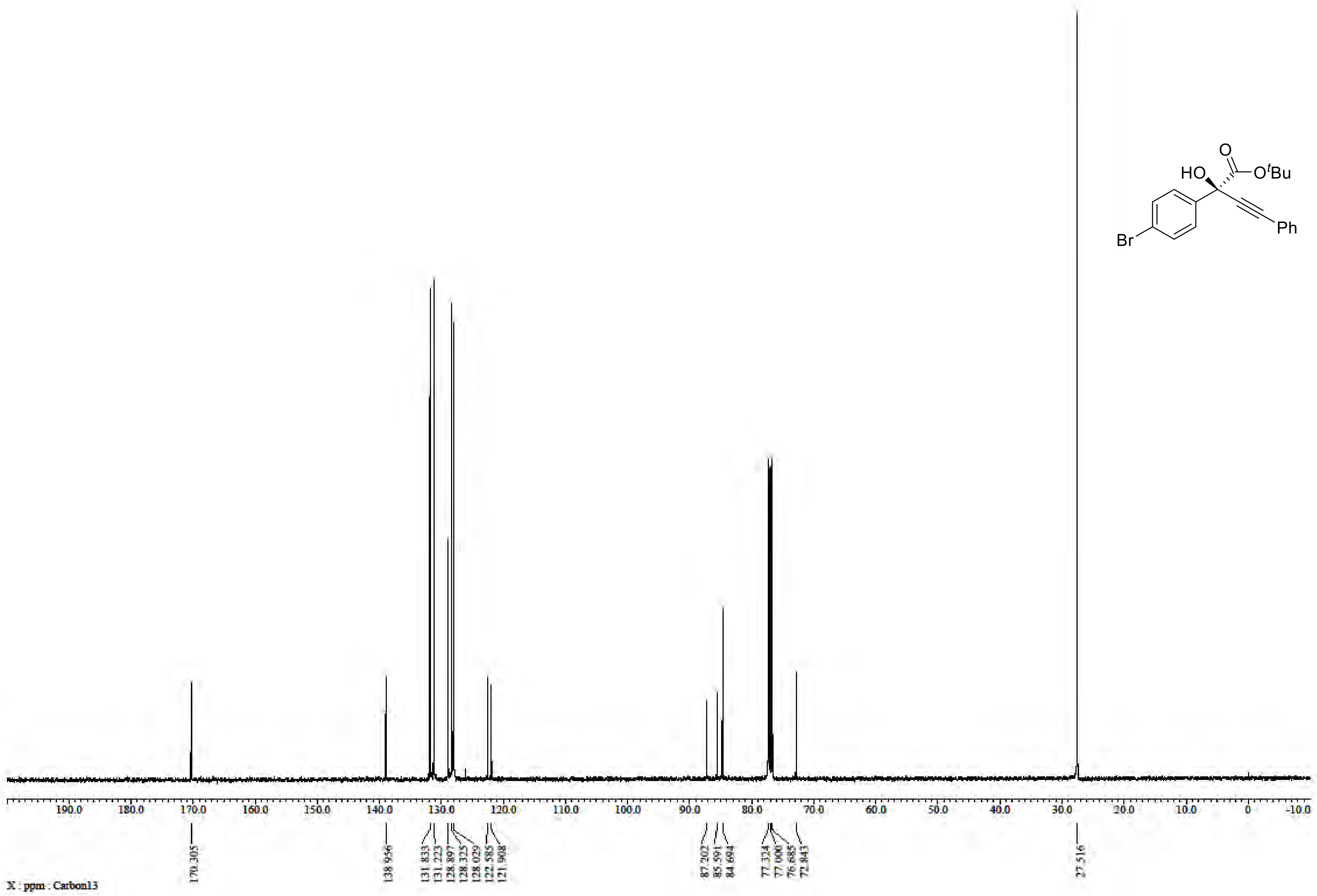

^{13}C NMR spectrum of **3ga** in $CDCl_3$

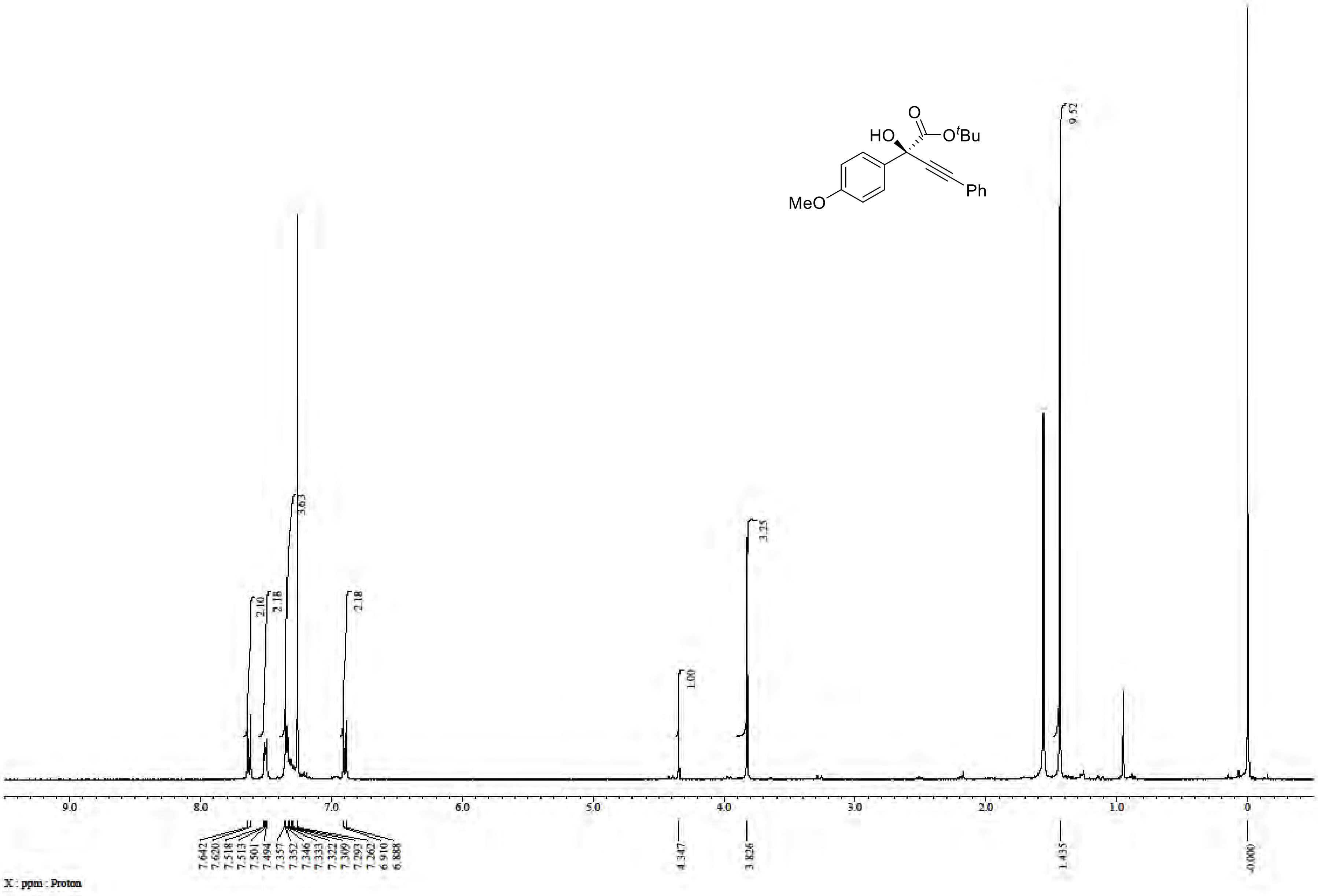

^{1}H NMR spectrum of **3ha** in $CDCl_3$

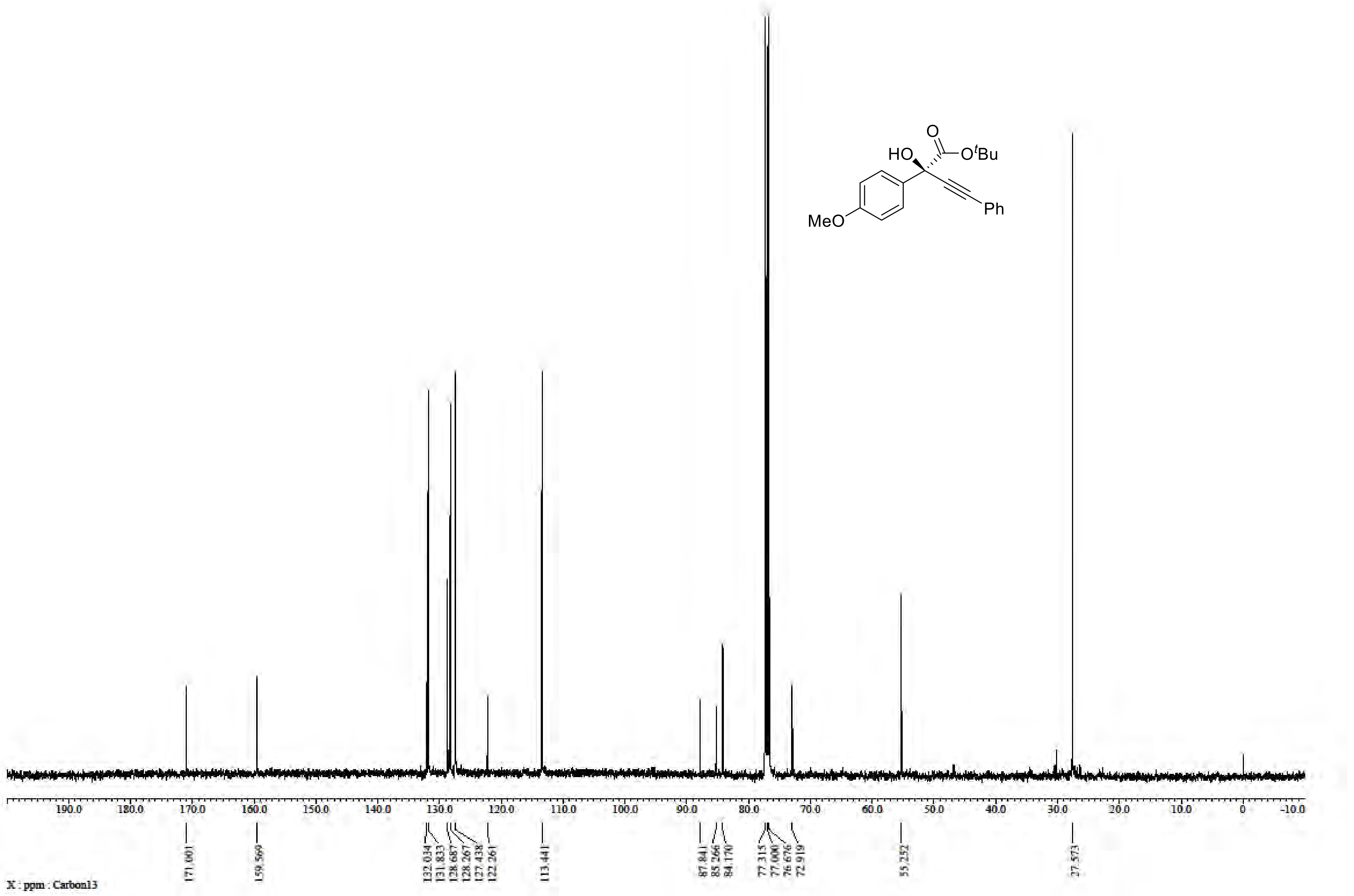

^{13}C NMR spectrum of **3ha** in $CDCl_3$

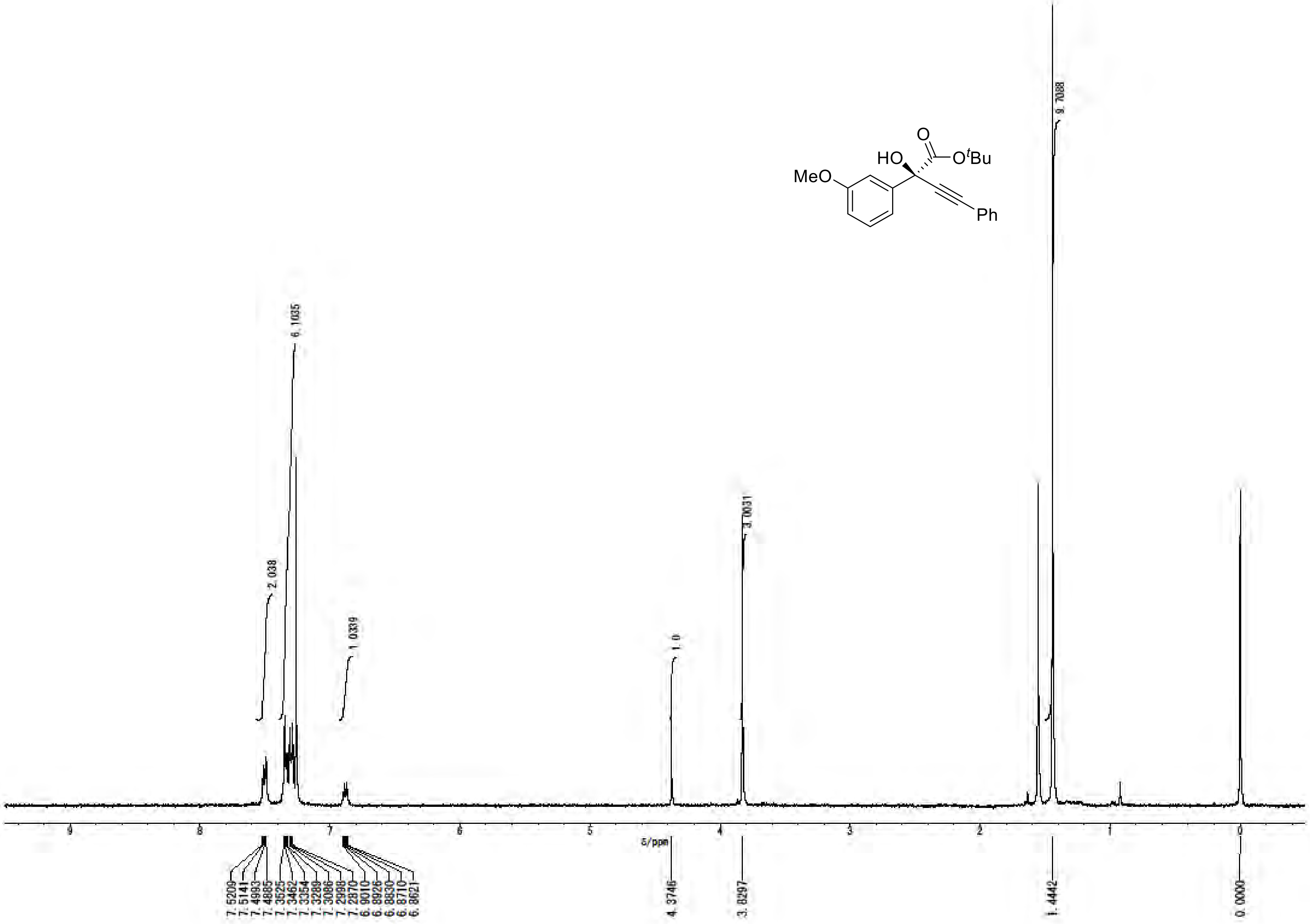

^{1}H NMR spectrum of **3ia** in $CDCl_3$

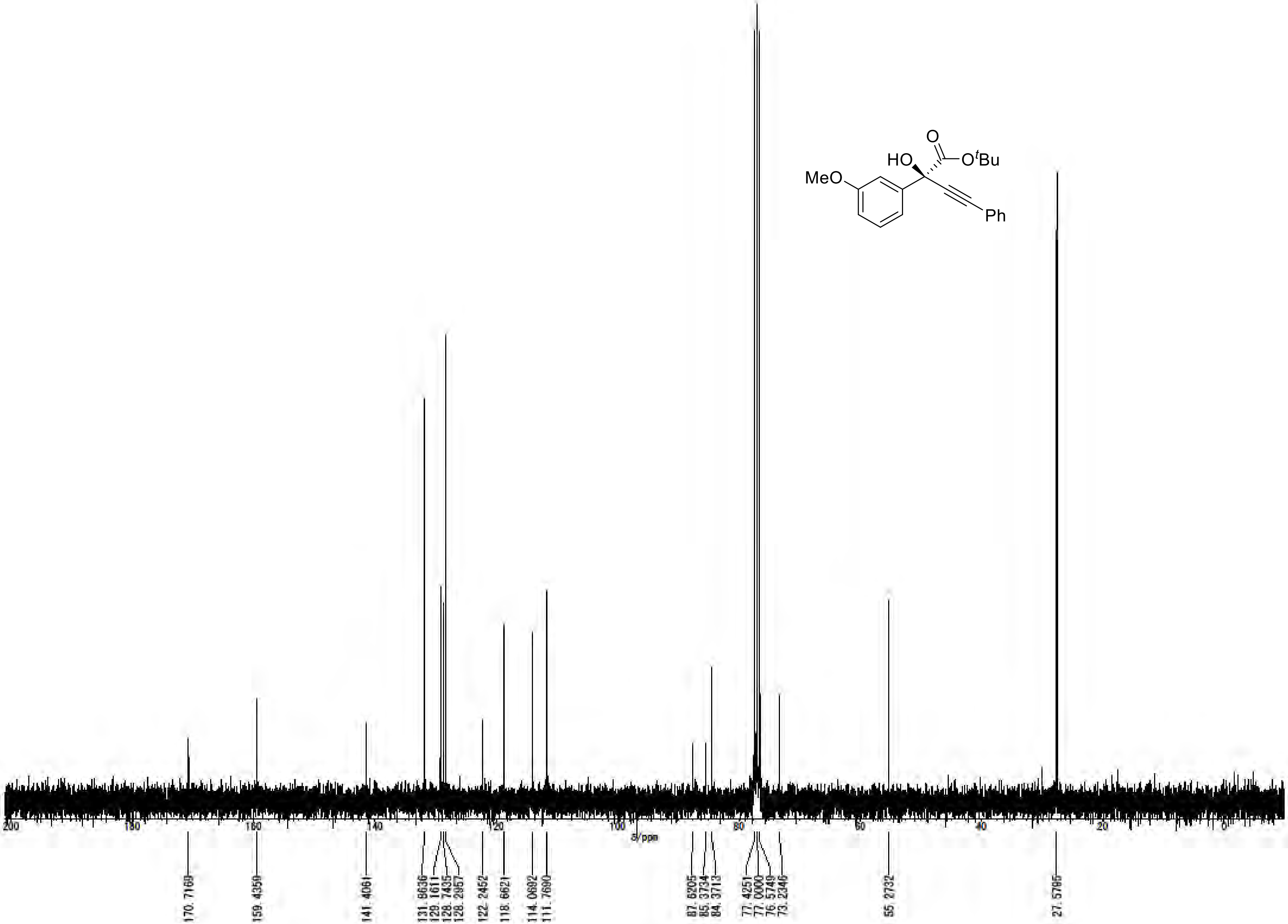

^{13}C NMR spectrum of **3ia** in $CDCl_3$

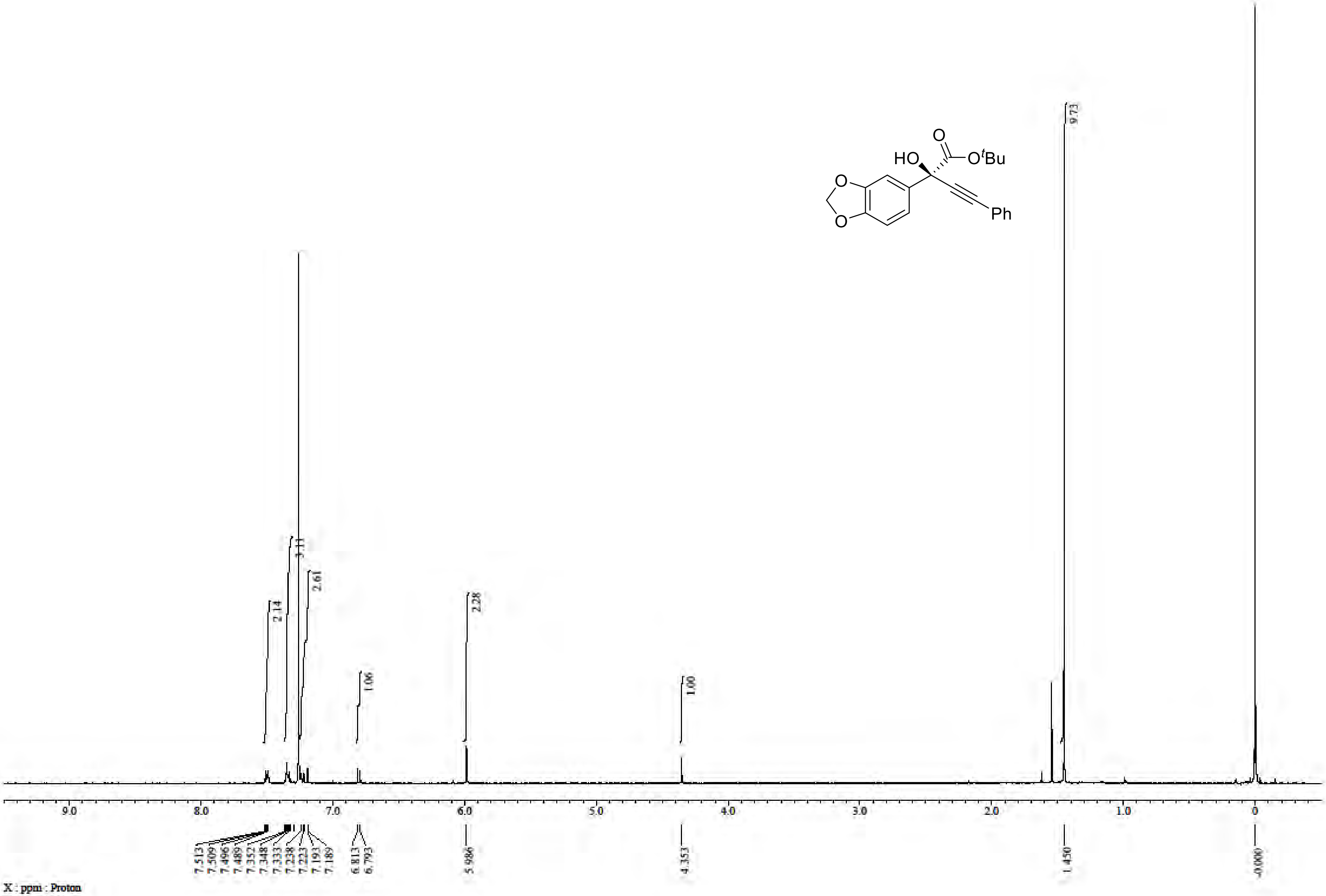

^{1}H NMR spectrum of **3ja** in $CDCl_3$

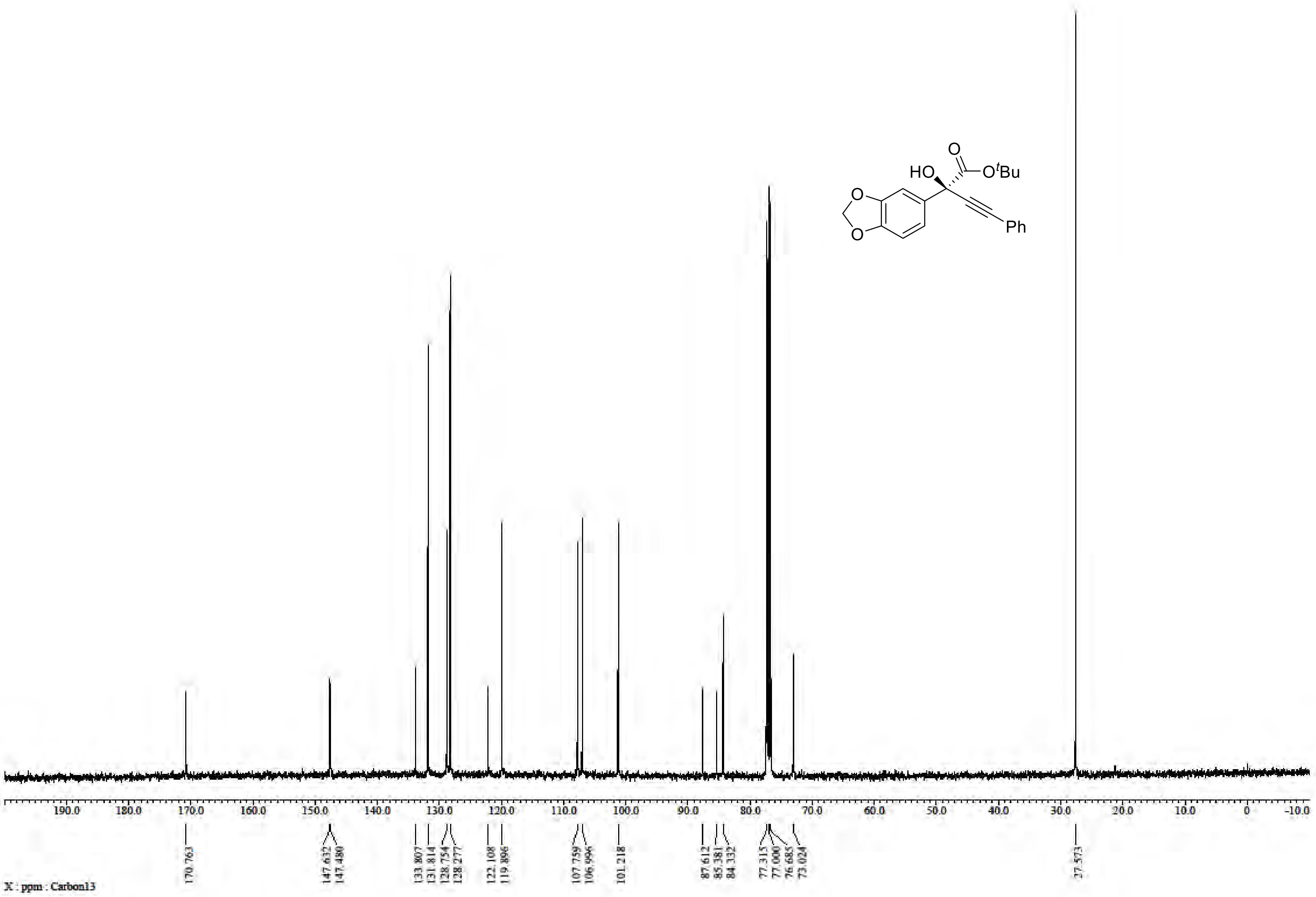

^{13}C NMR spectrum of **3ja** in $CDCl_3$

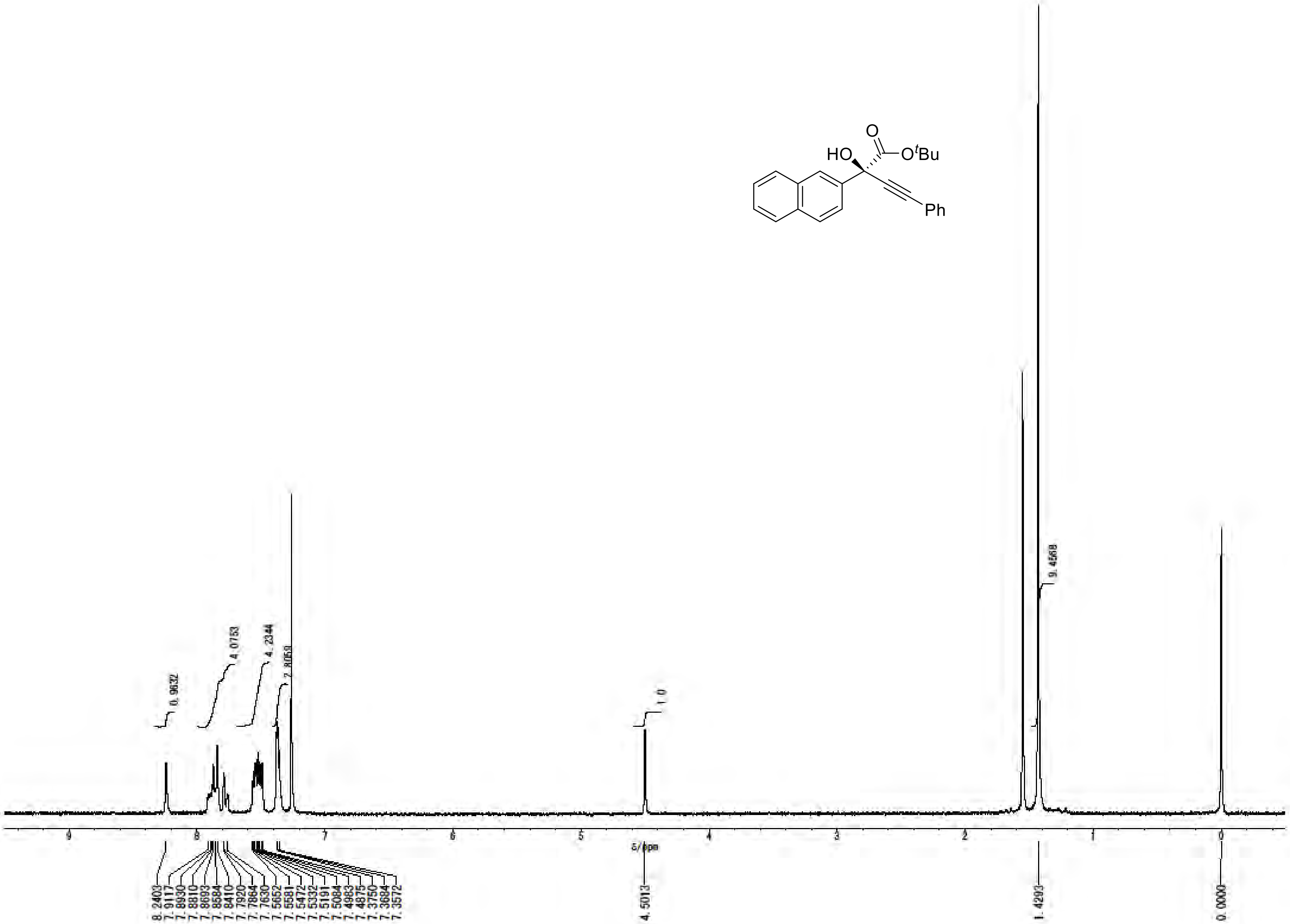

^{1}H NMR spectrum of **3ka** in $CDCl_3$

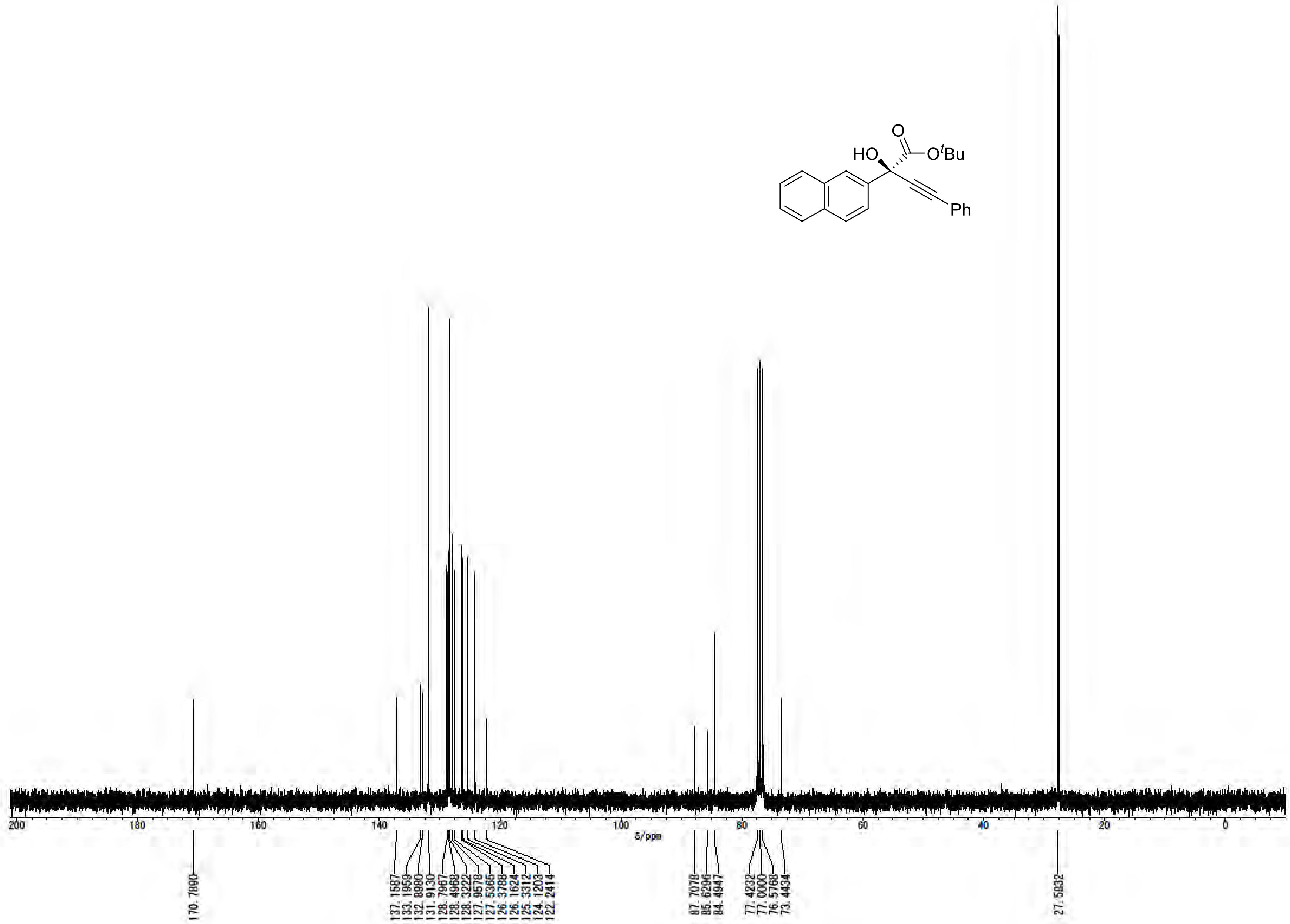

^{13}C NMR spectrum of **3ka** in $CDCl_3$

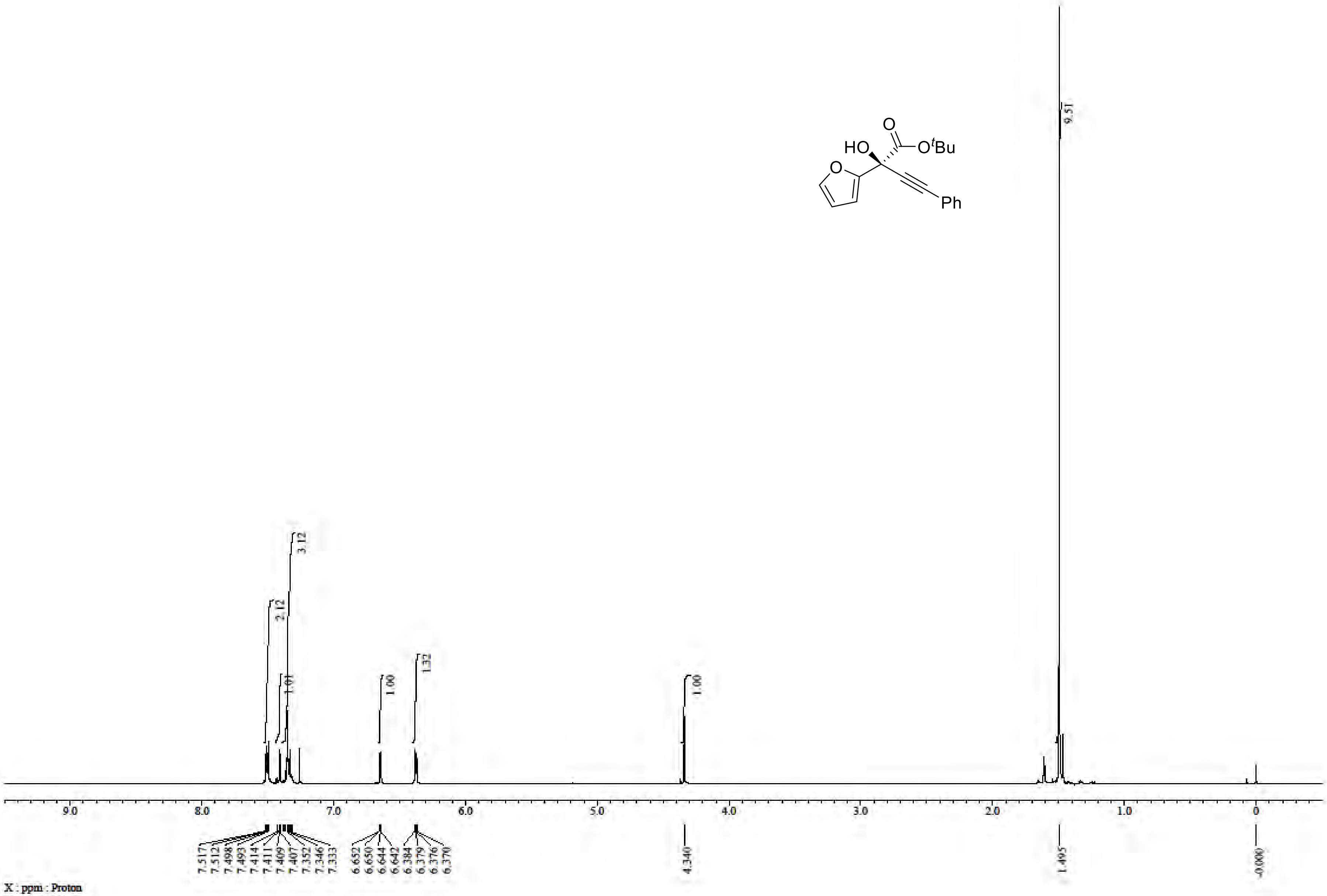

^{1}H NMR spectrum of **3la** in $CDCl_3$

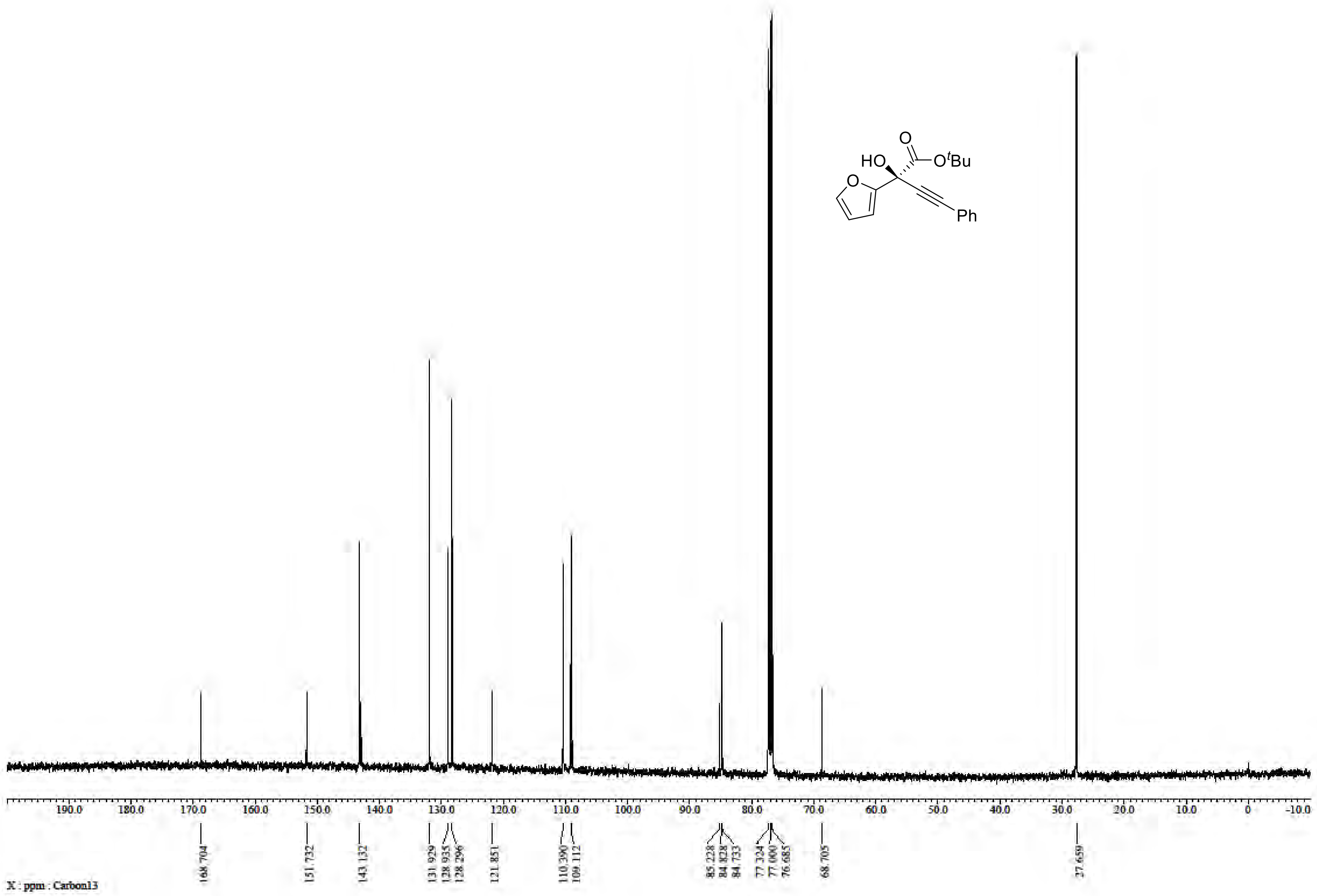

^{13}C NMR spectrum of **3la** in $CDCl_3$

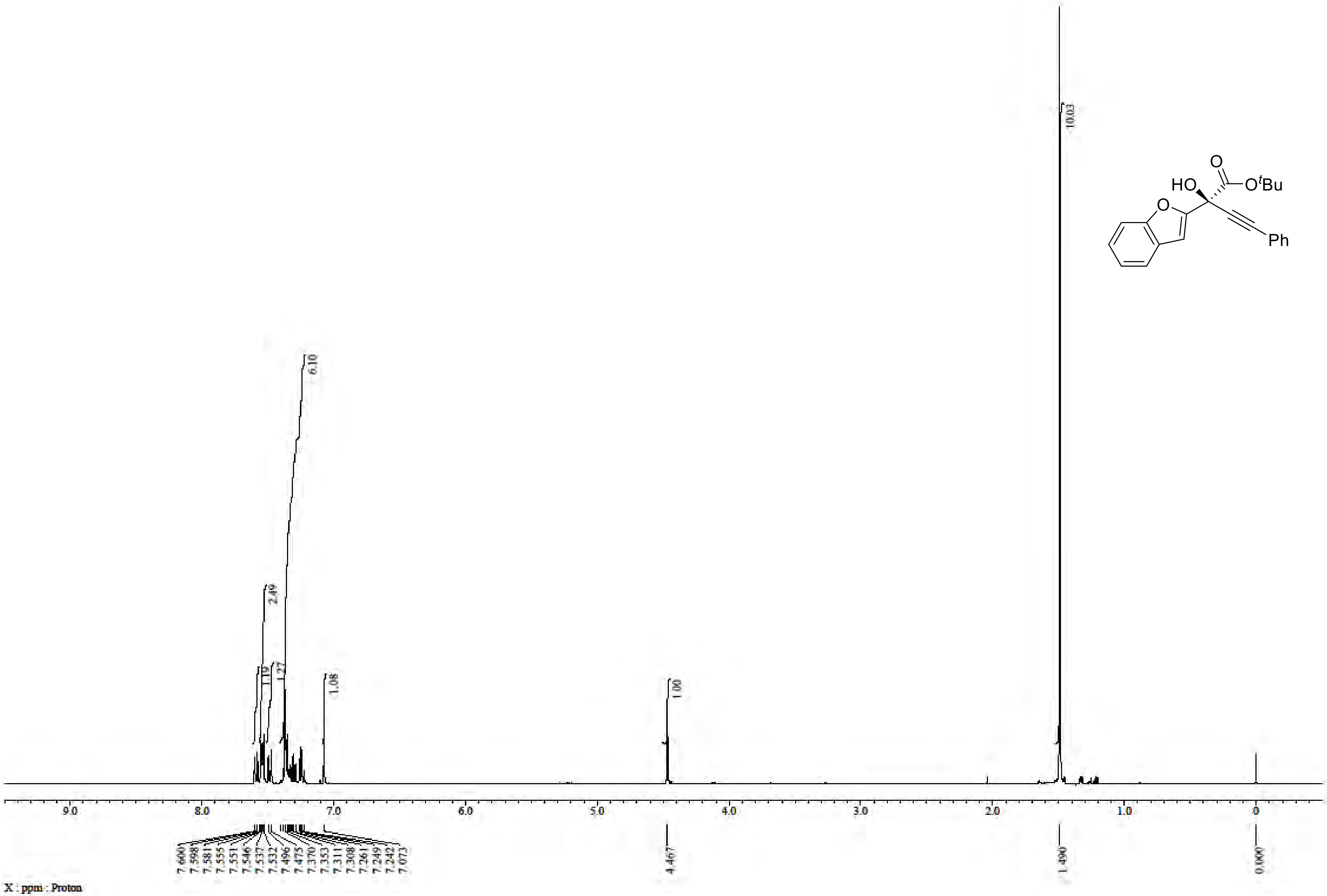

^{1}H NMR spectrum of **3ma** in $CDCl_3$

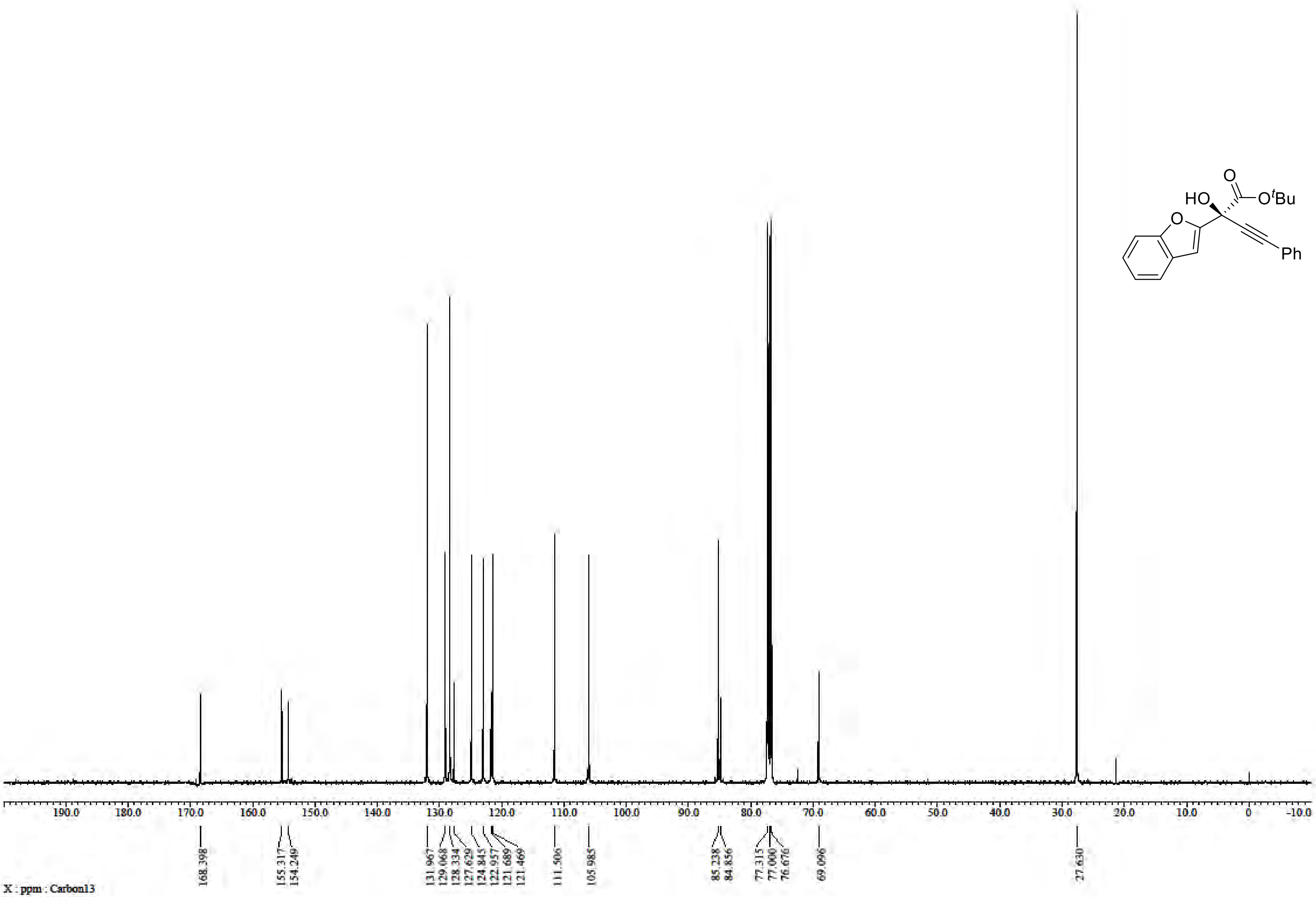

^{13}C NMR spectrum of **3ma** in $CDCl_3$

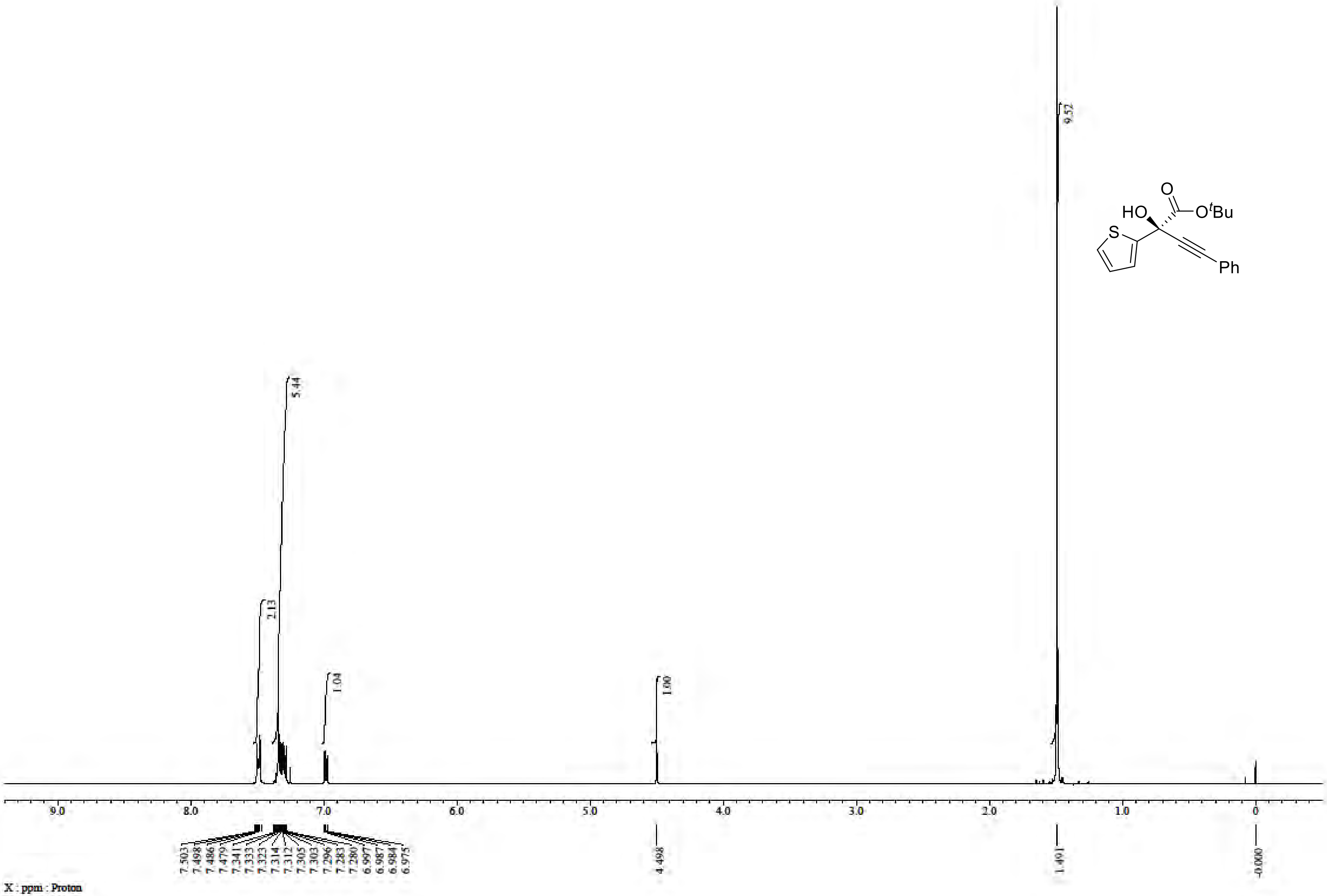

^{1}H NMR spectrum of **3na** in $CDCl_3$

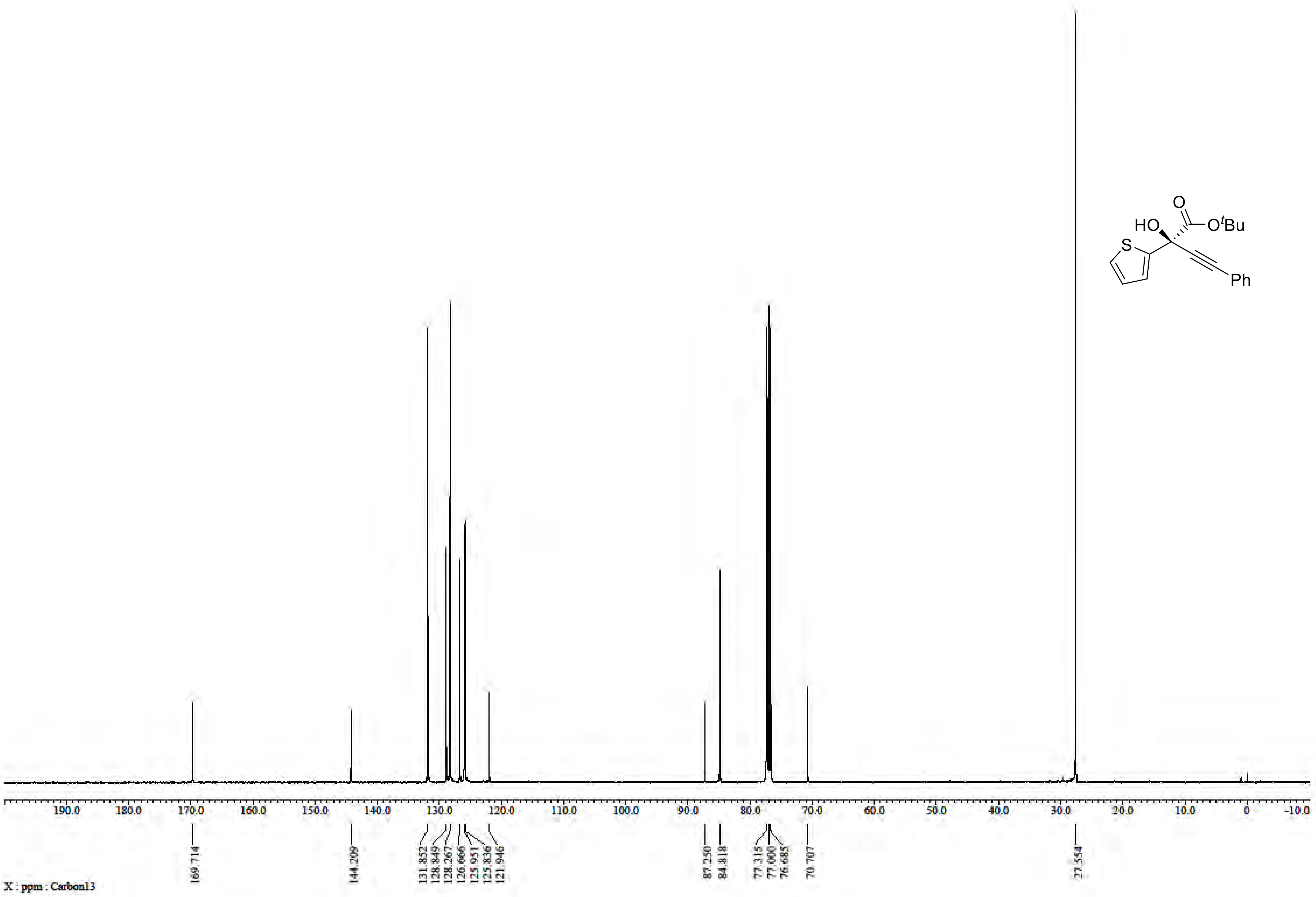

^{13}C NMR spectrum of **3na** in $CDCl_3$

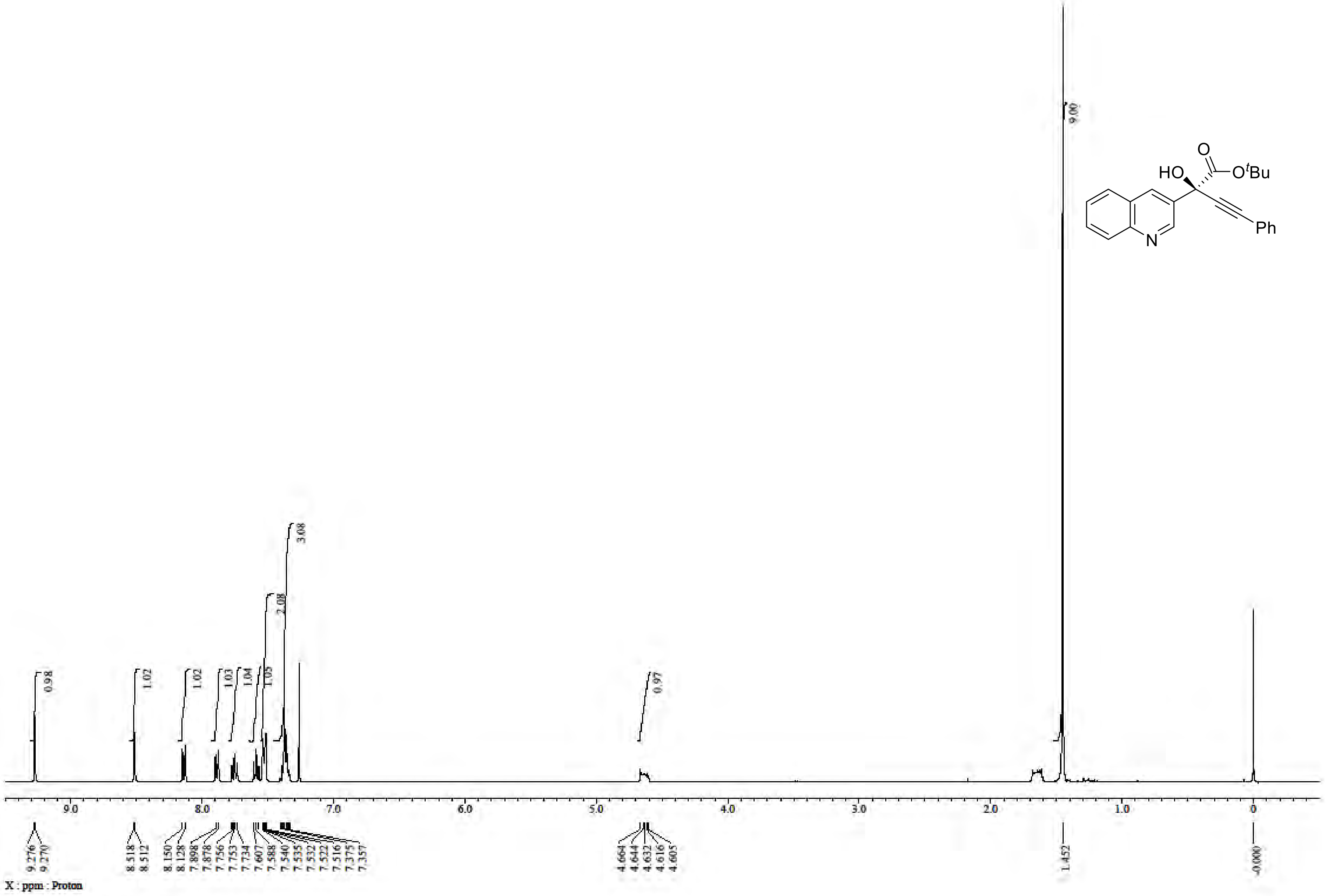

^{1}H NMR spectrum of **3oa** in $CDCl_3$

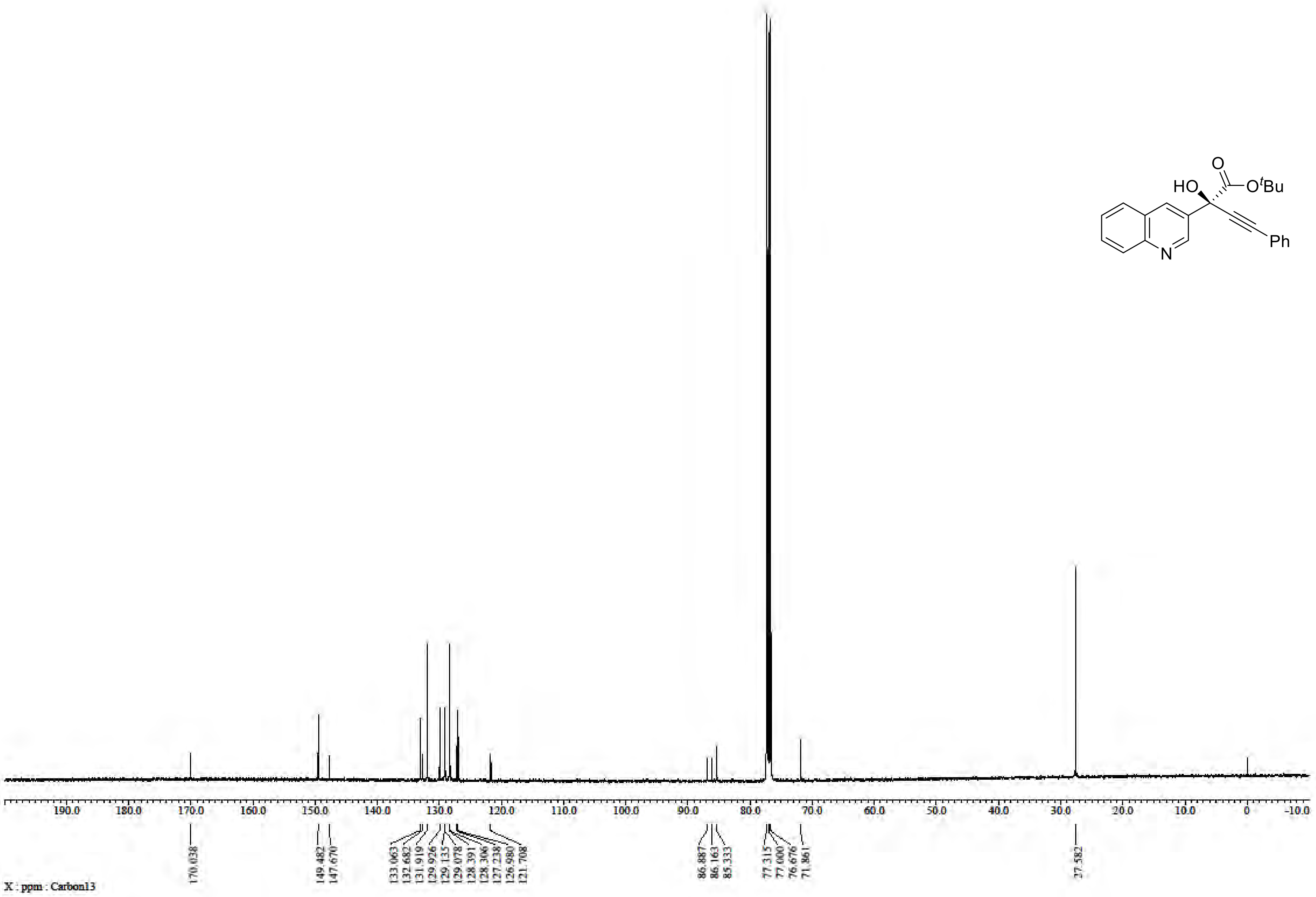

^{13}C NMR spectrum of **3oa** in $CDCl_3$

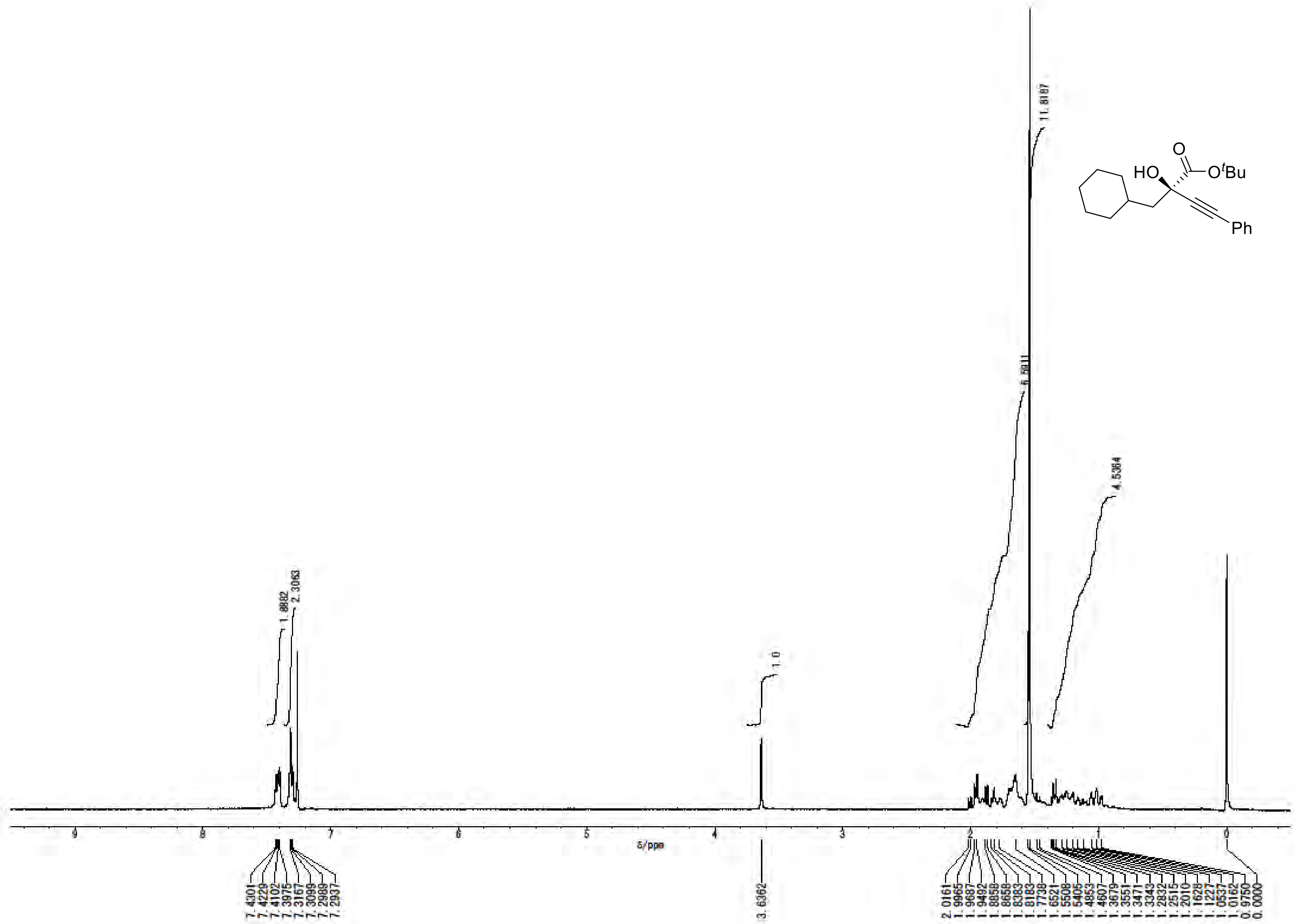

^{1}H NMR spectrum of **3pa** in $CDCl_3$

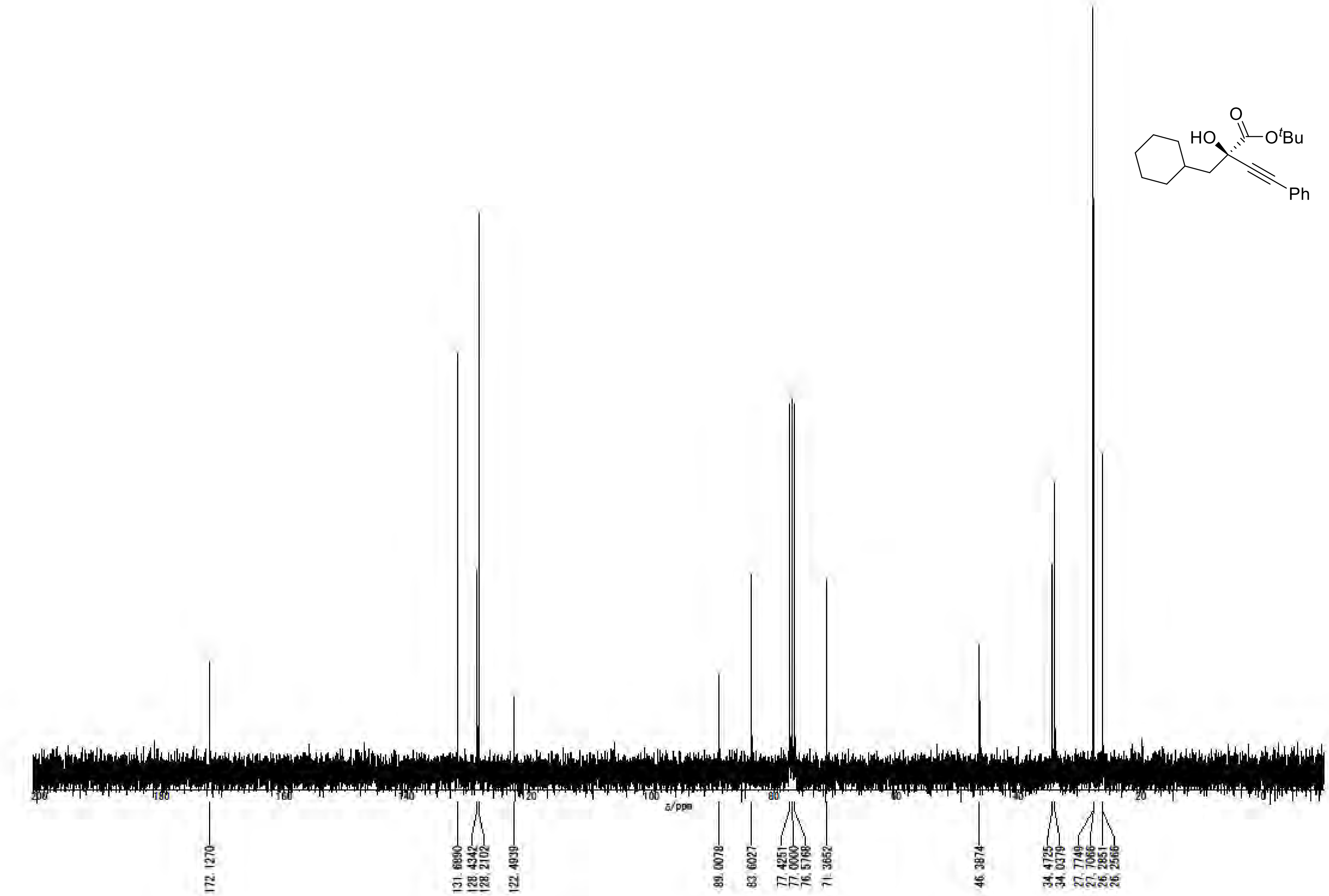

^{13}C NMR spectrum of **3pa** in $CDCl_3$

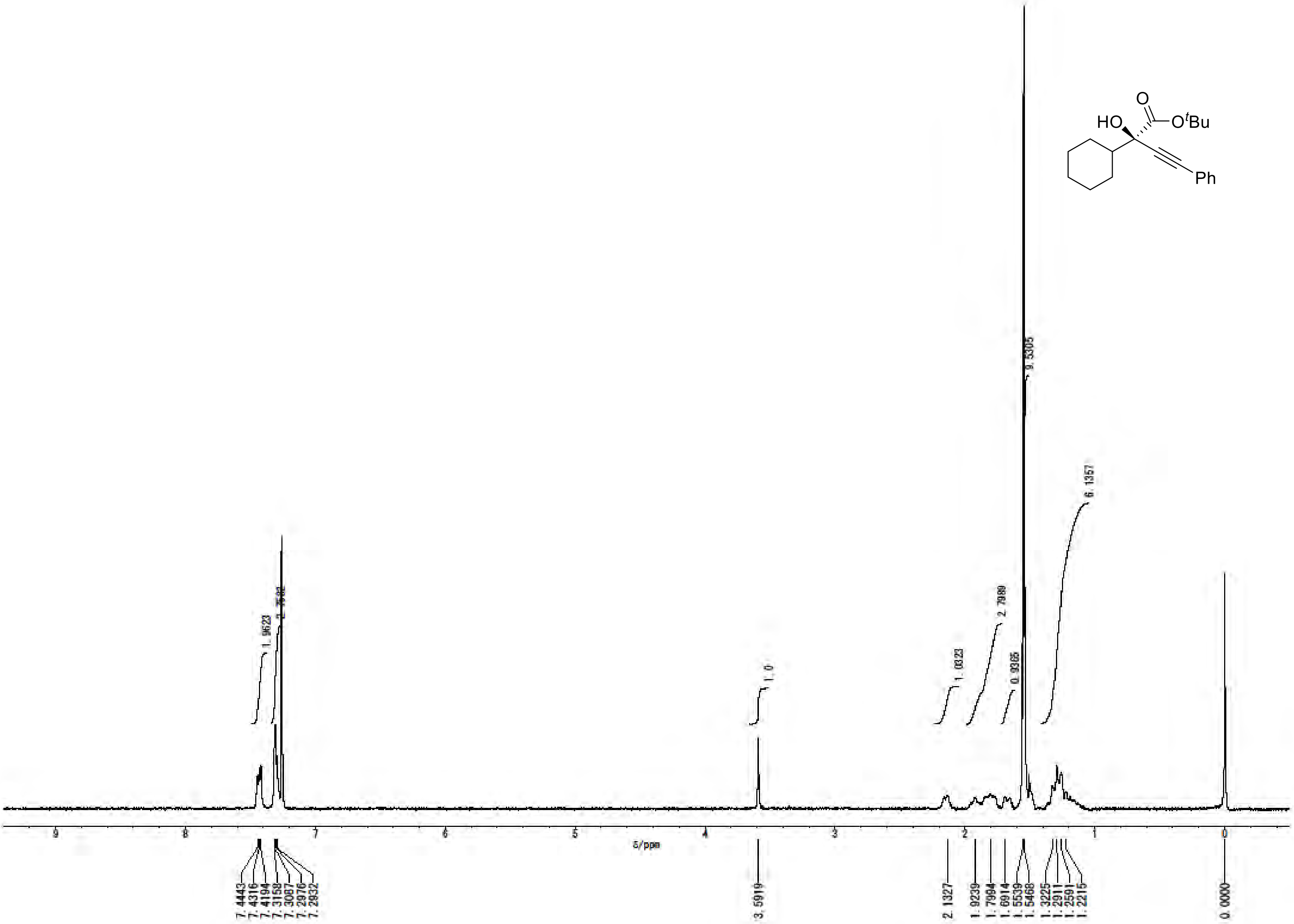

^{1}H NMR spectrum of **3qa** in $CDCl_3$

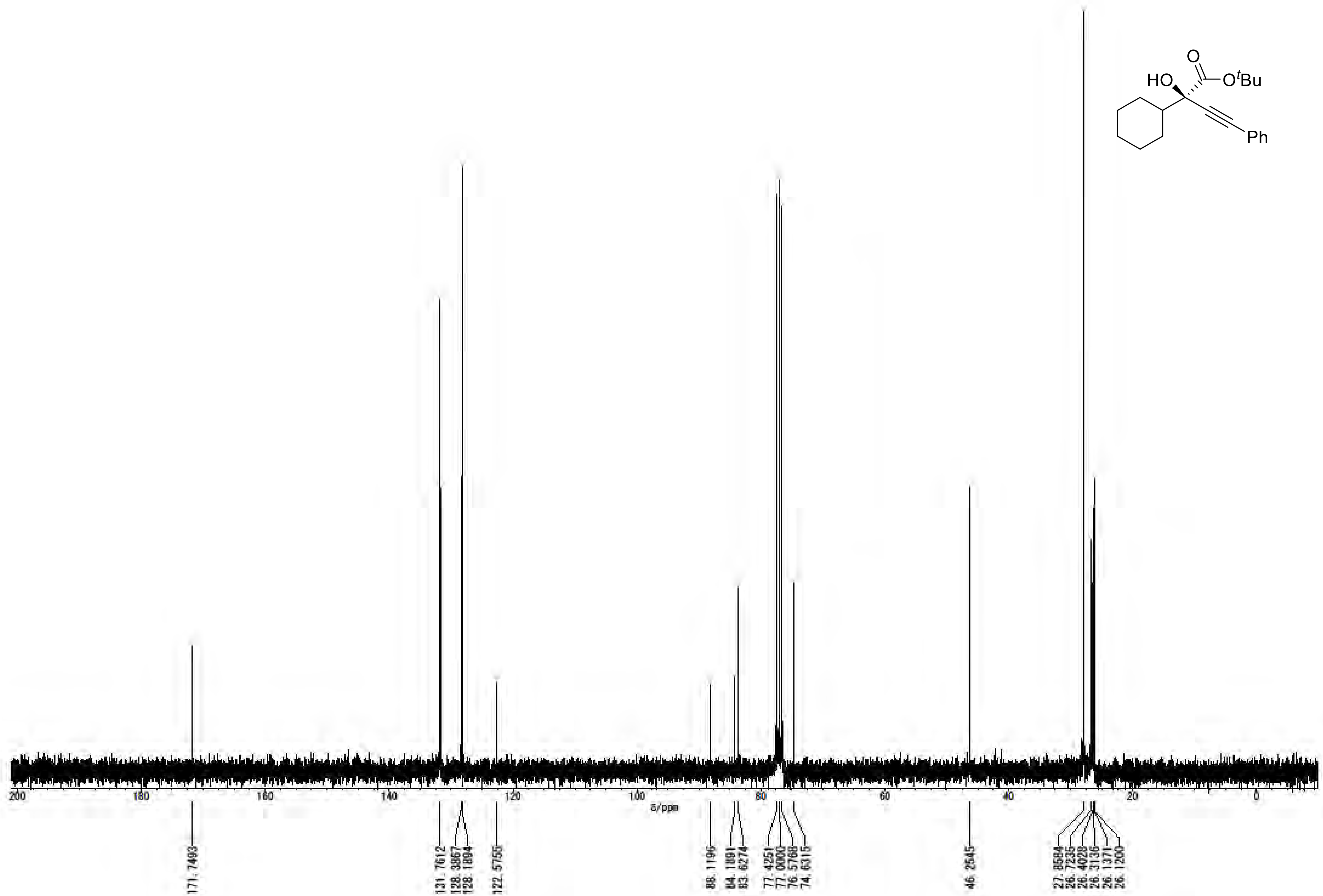

^{13}C NMR spectrum of **3qa** in $CDCl_3$

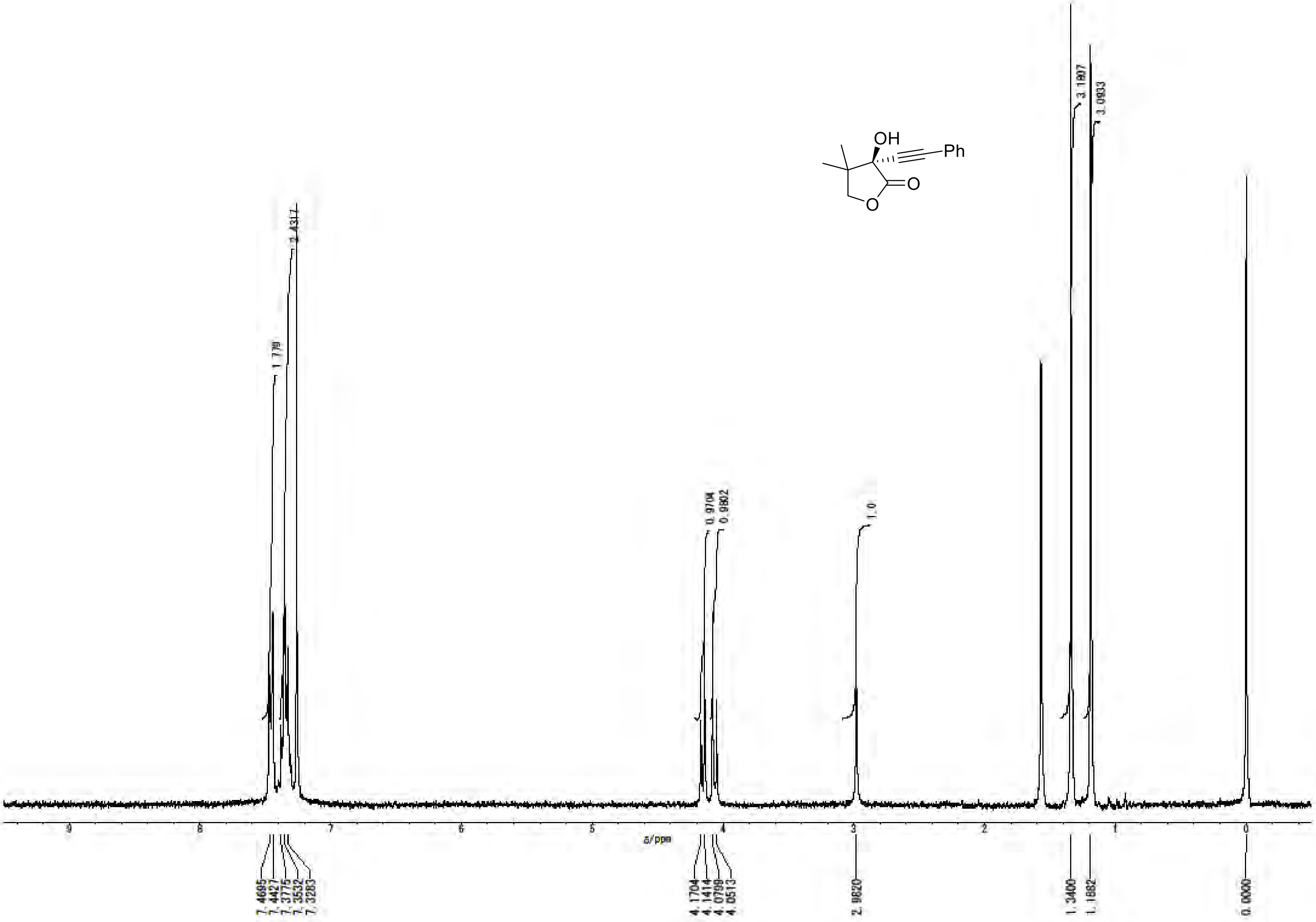

^{1}H NMR spectrum of **3ra** in $CDCl_3$

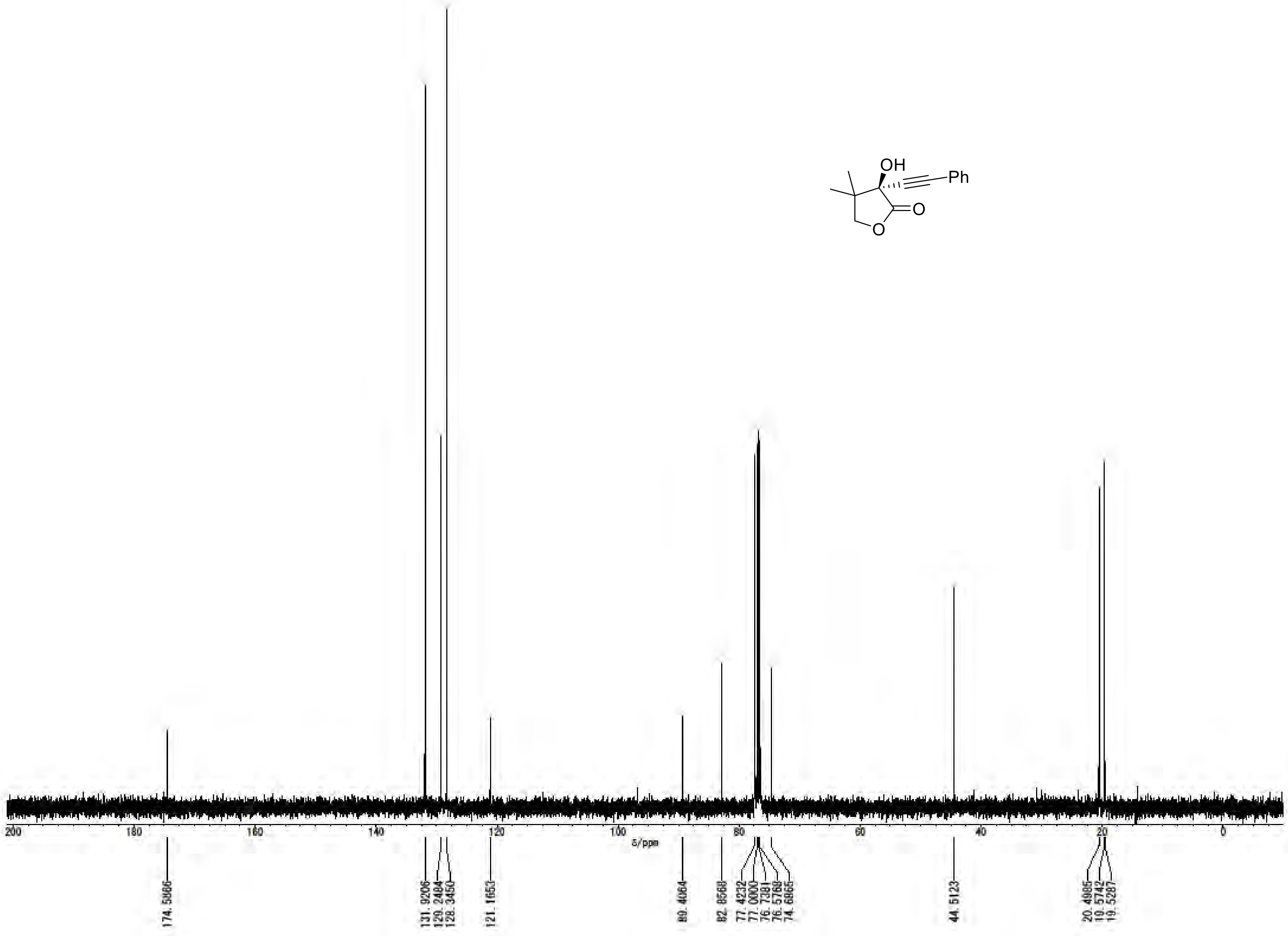

^{13}C NMR spectrum of **3ra** in $CDCl_3$

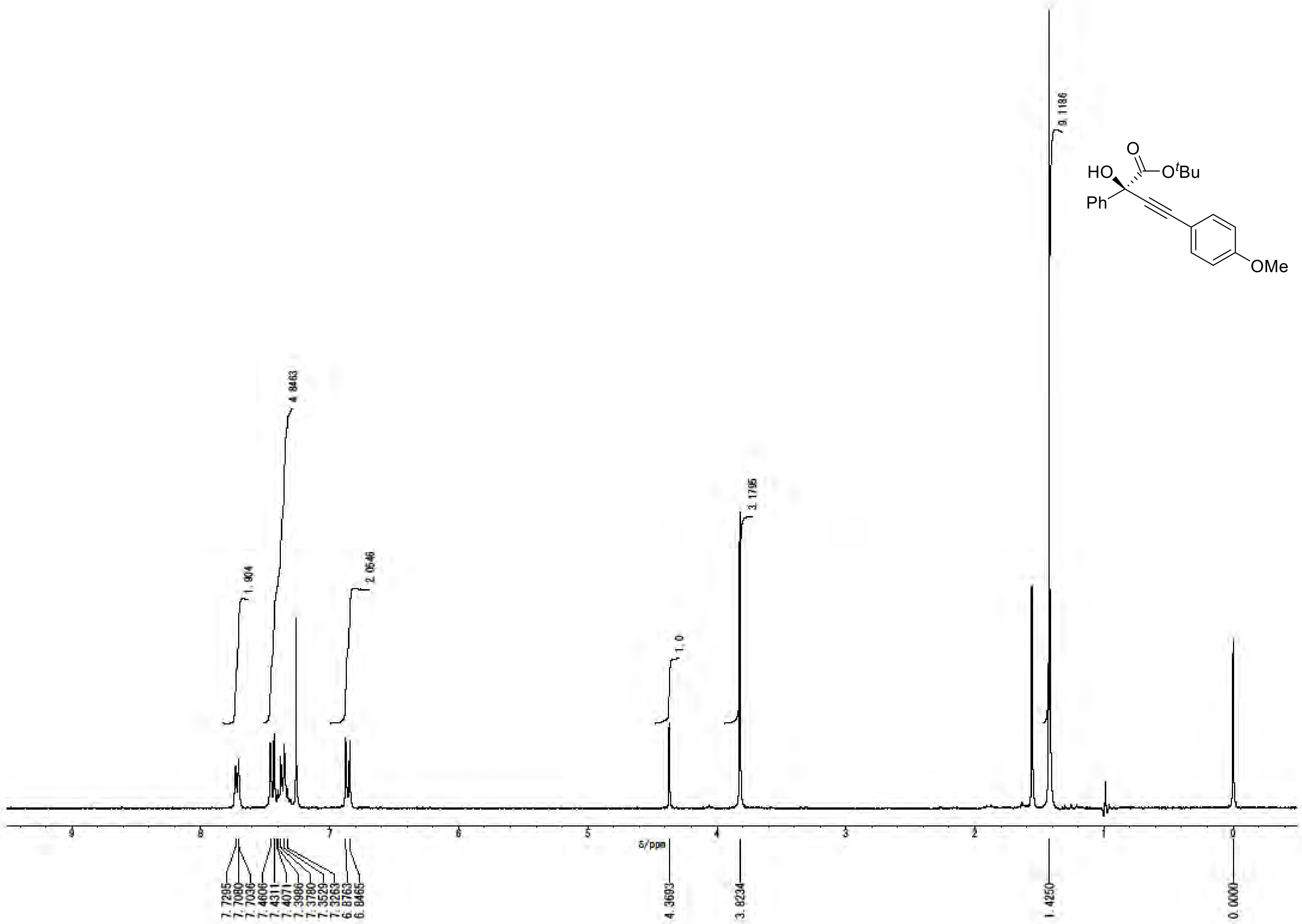

^{1}H NMR spectrum of **3cb** in $CDCl_3$

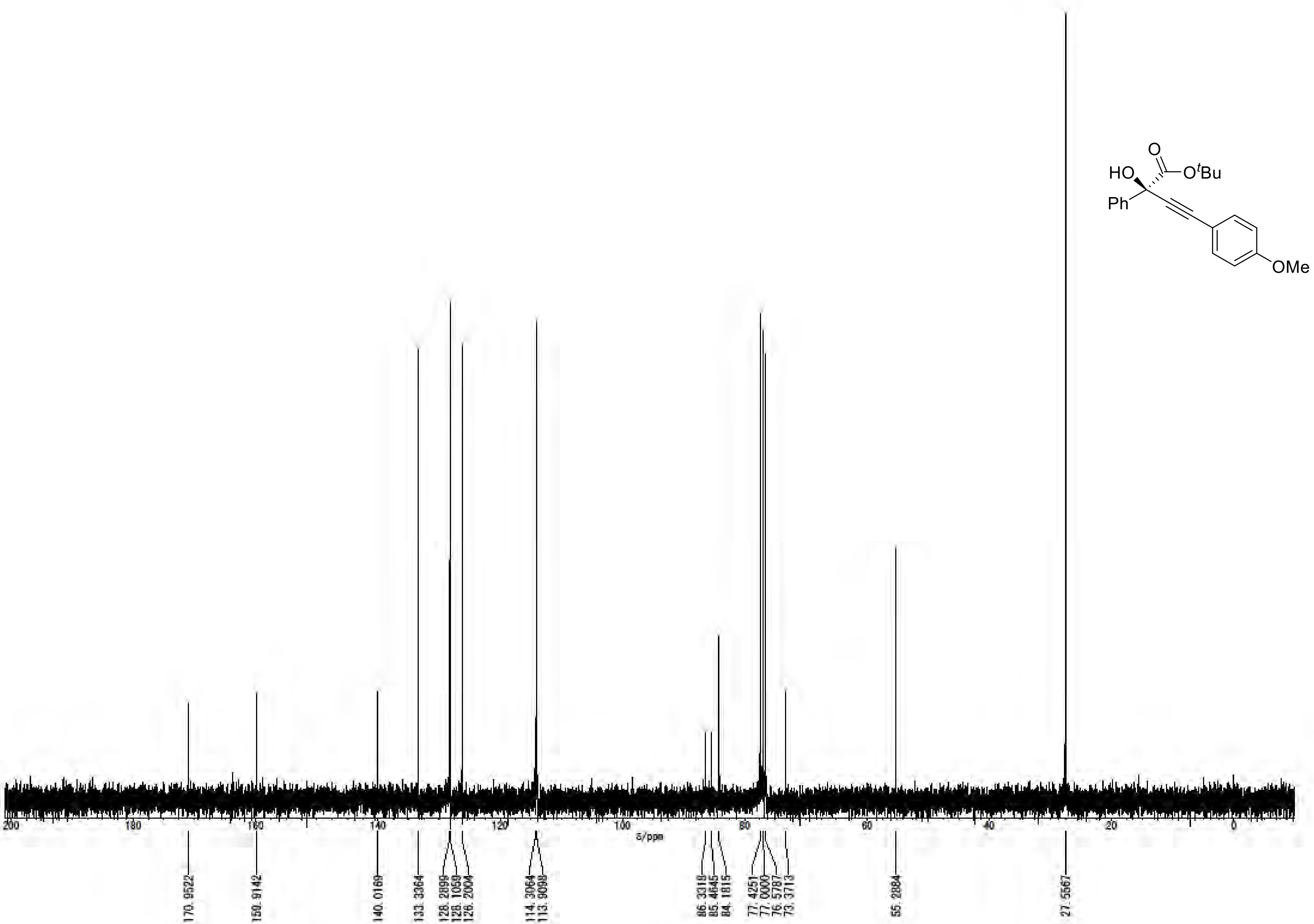

^{13}C NMR spectrum of **3cb** in $CDCl_3$

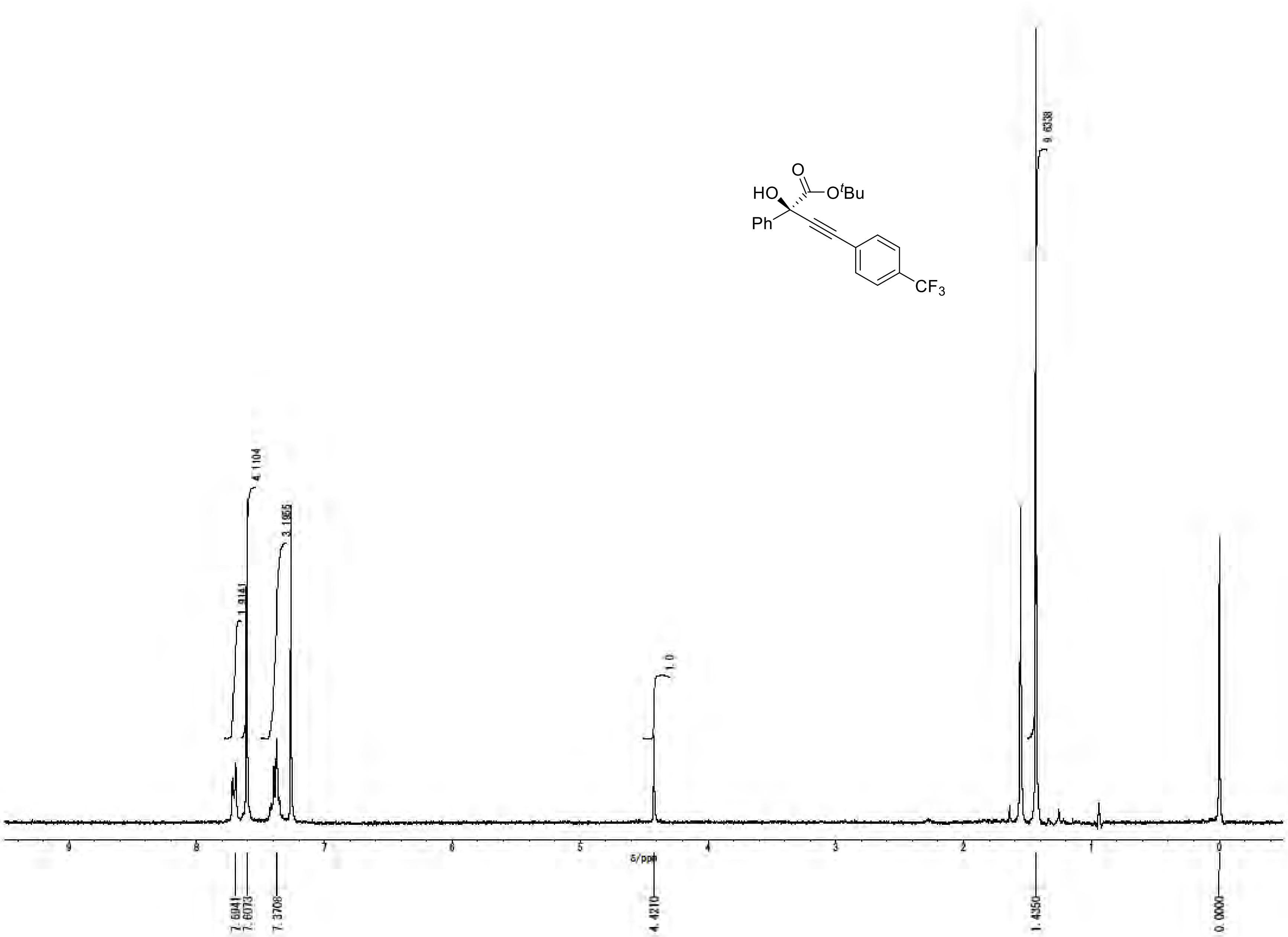

^{1}H NMR spectrum of **3cc** in $CDCl_3$

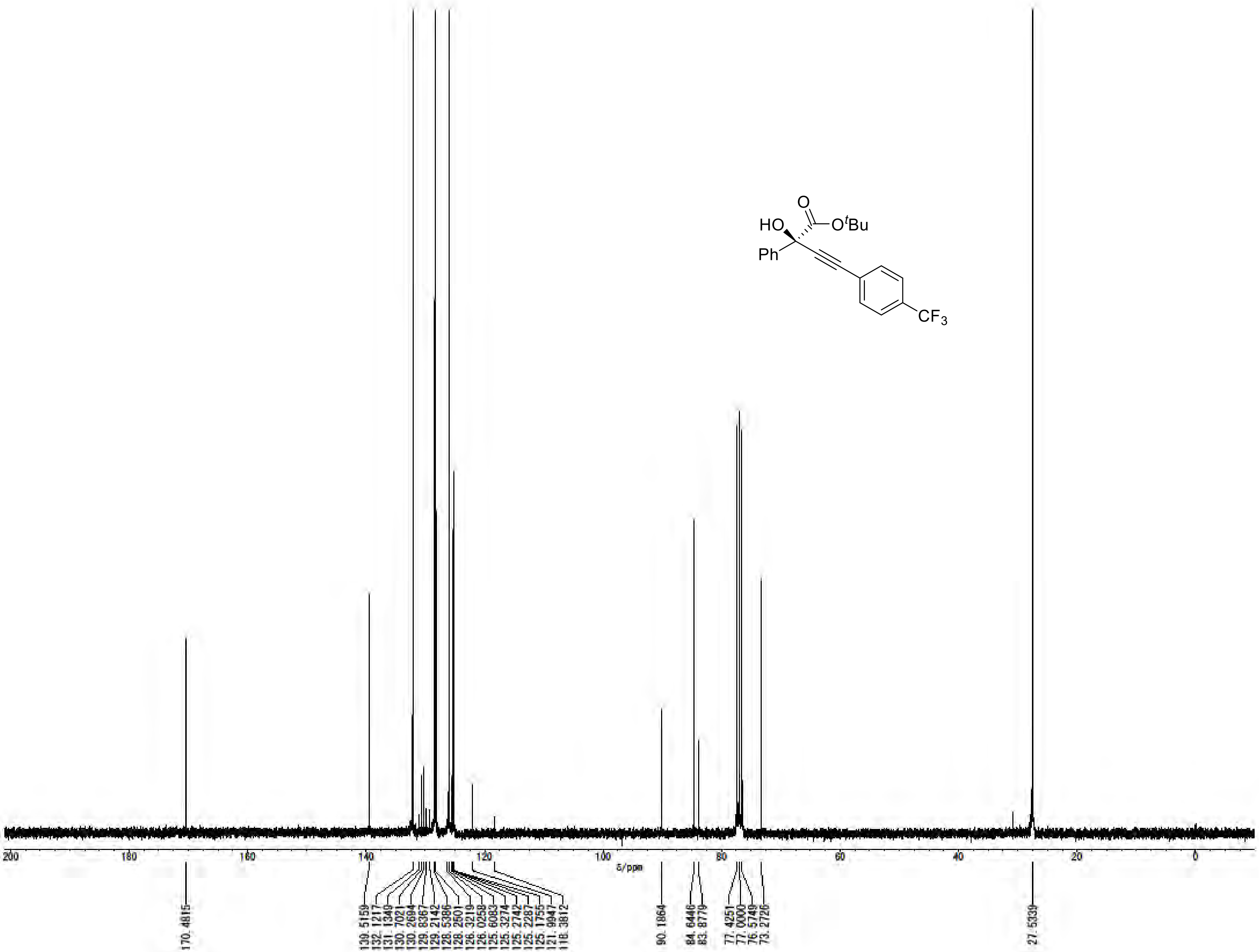

^{13}C NMR spectrum of **3cc** in $CDCl_3$

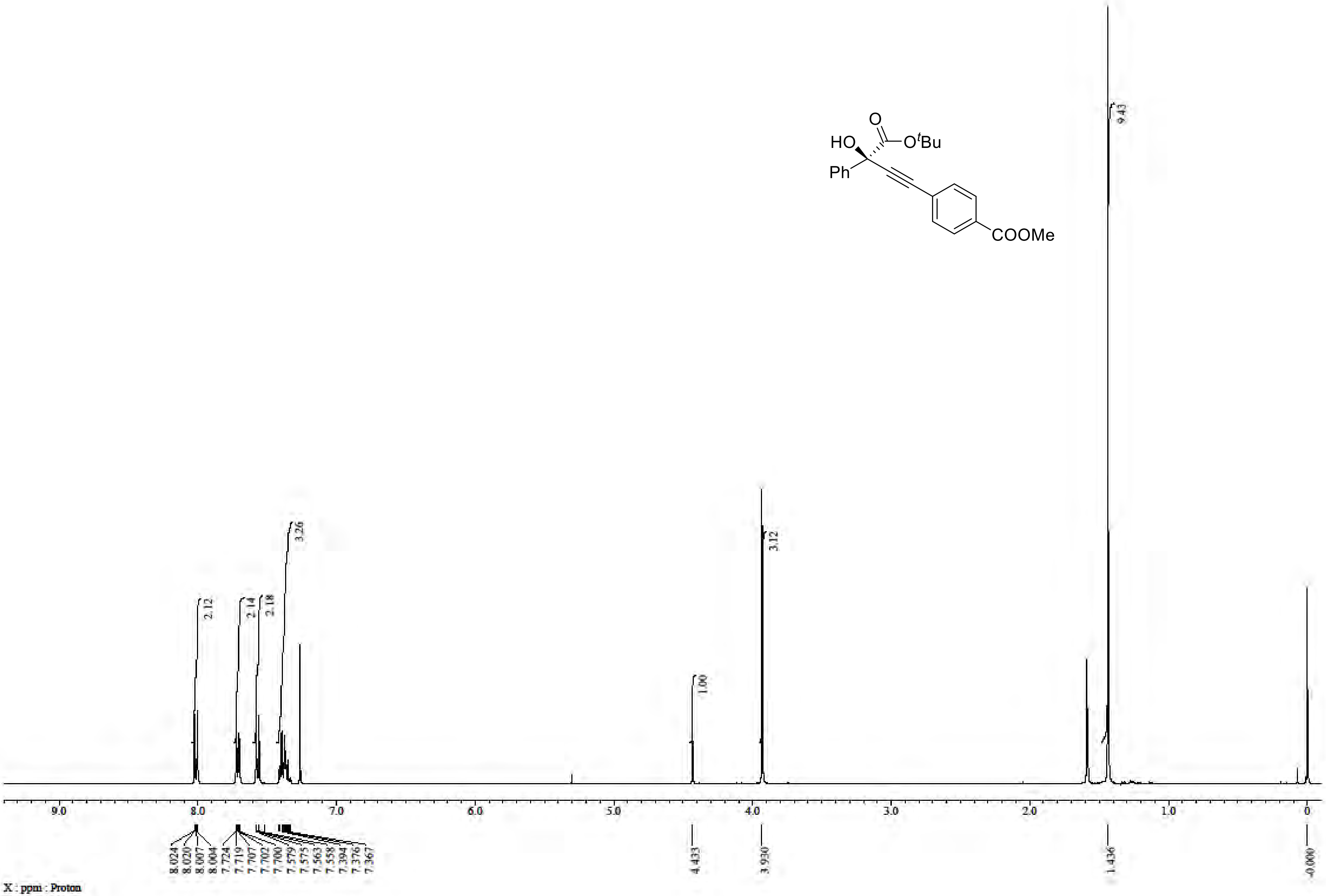

^{1}H NMR spectrum of **3cd** in $CDCl_3$

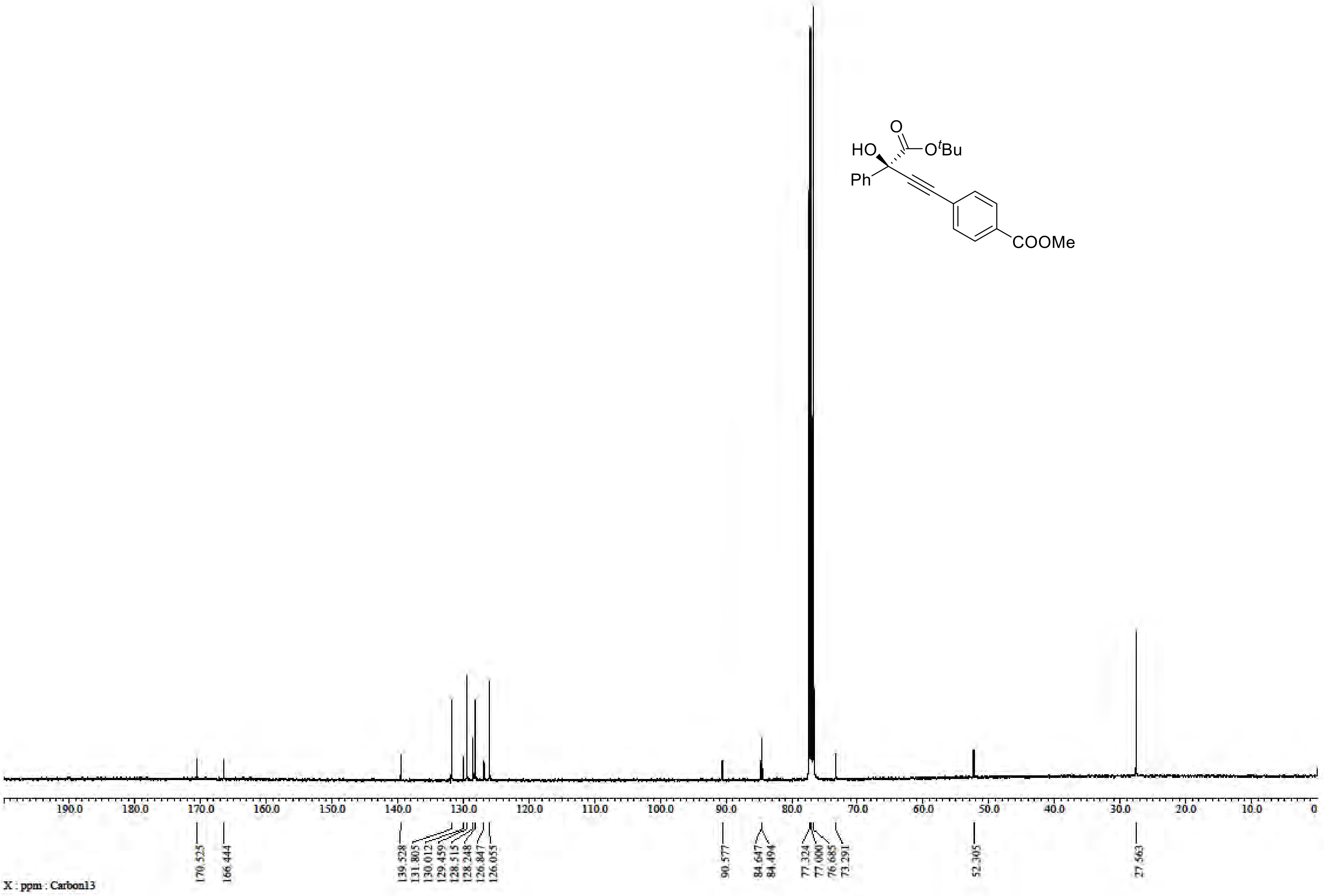

^{13}C NMR spectrum of **3cd** in $CDCl_3$

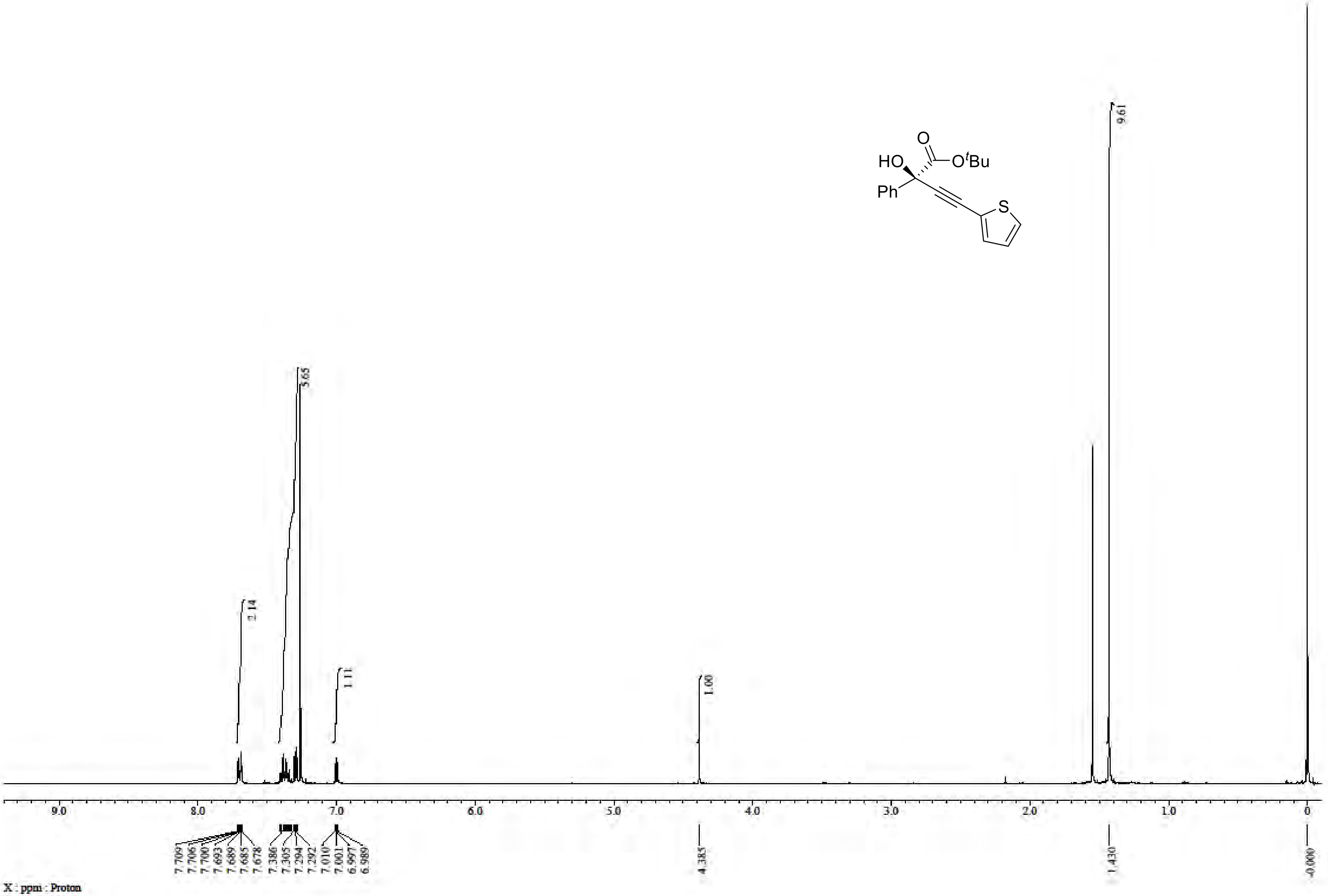

^{1}H NMR spectrum of **3ce** in $CDCl_3$

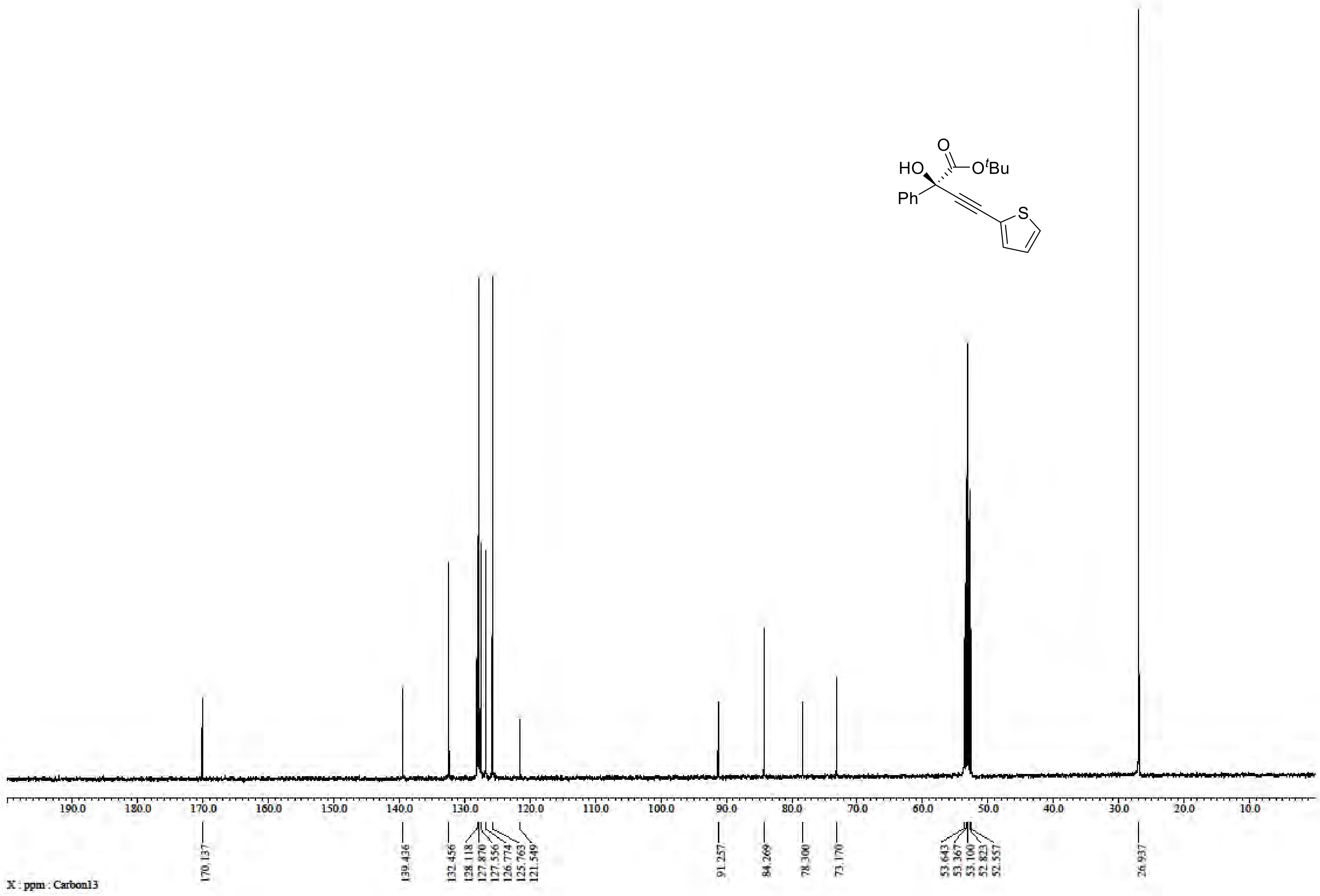

^{13}C NMR spectrum of **3ce** in CD_2Cl_2

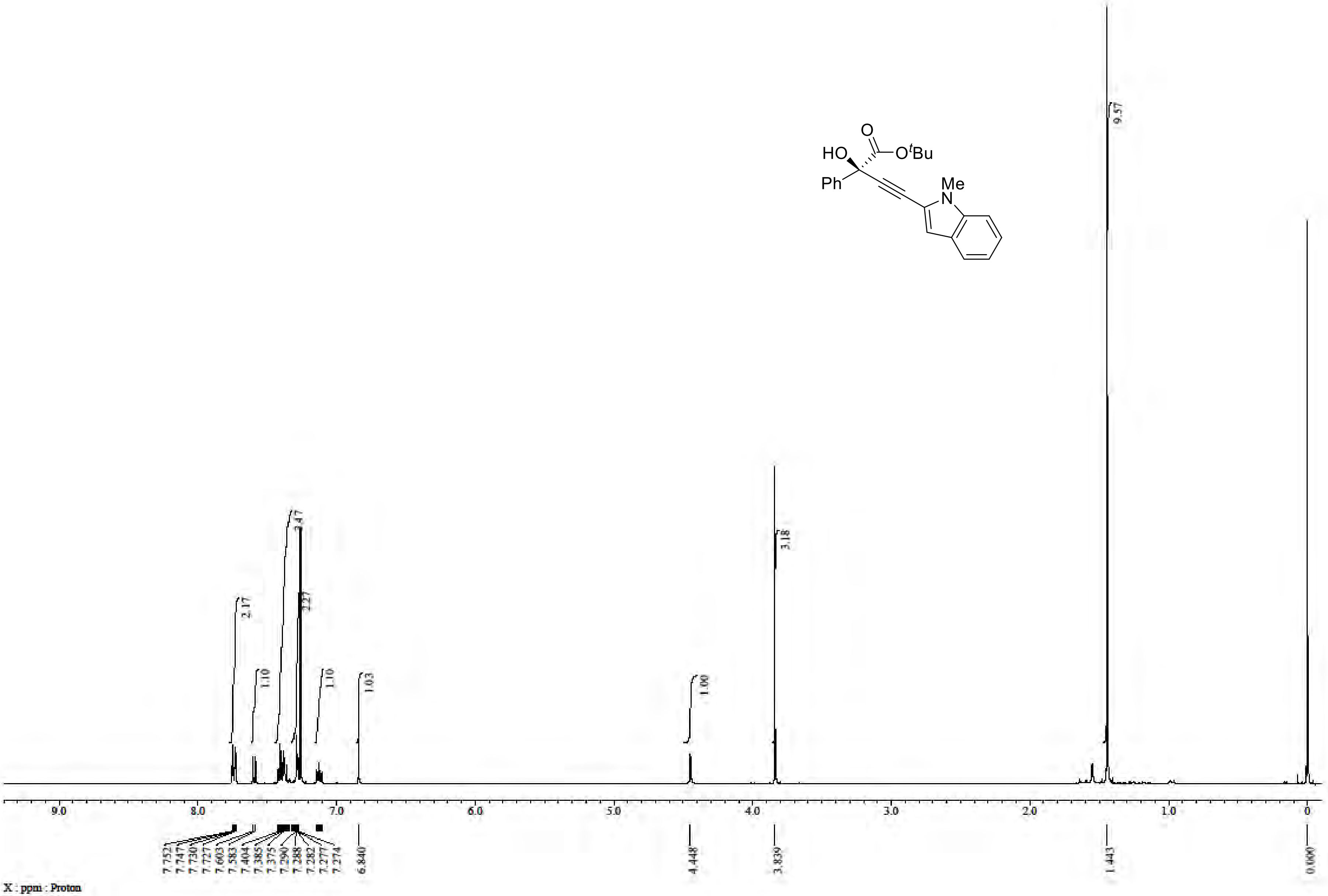

^{1}H NMR spectrum of **3cf** in $CDCl_3$

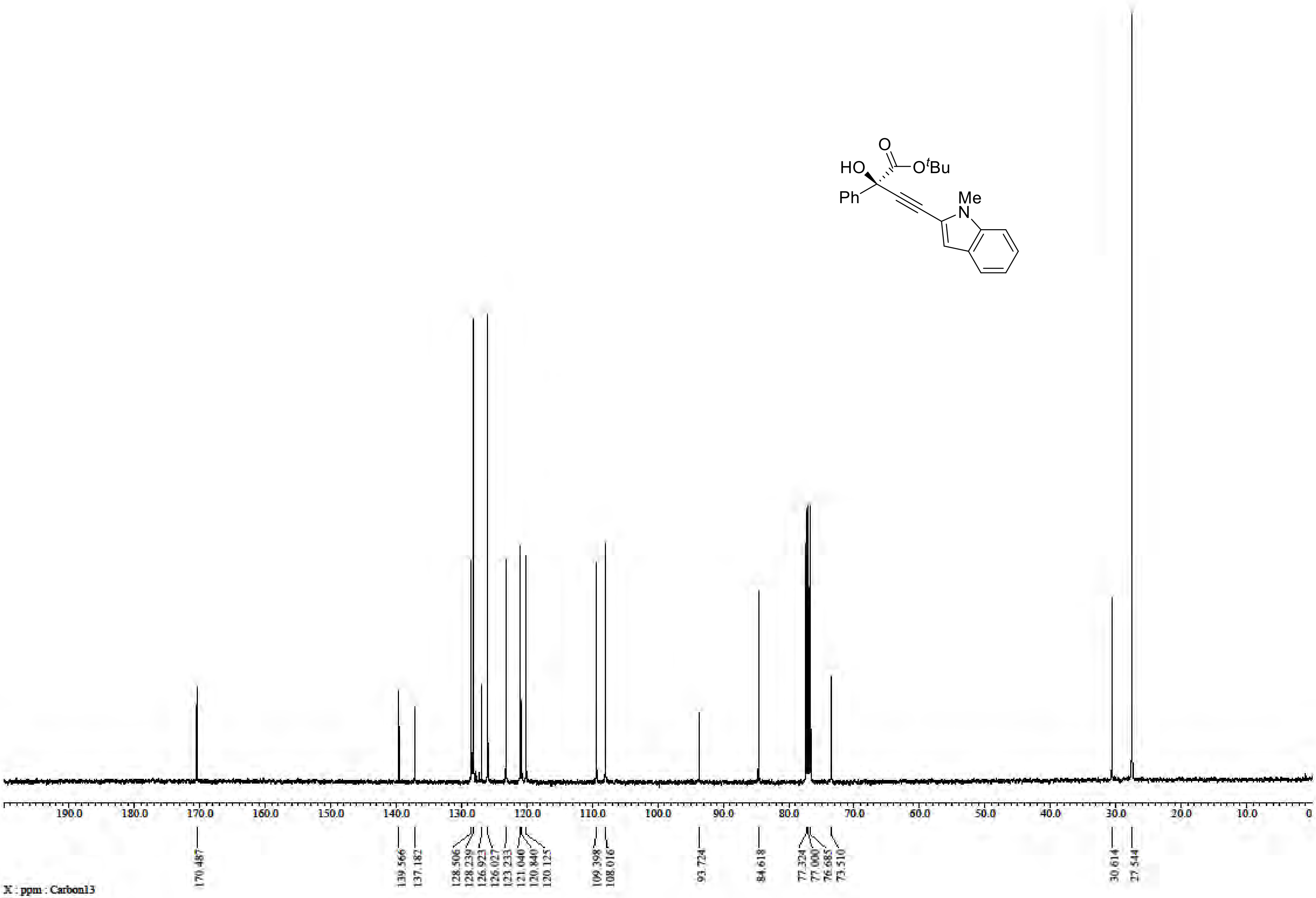

^{13}C NMR spectrum of **3cf** in $CDCl_3$

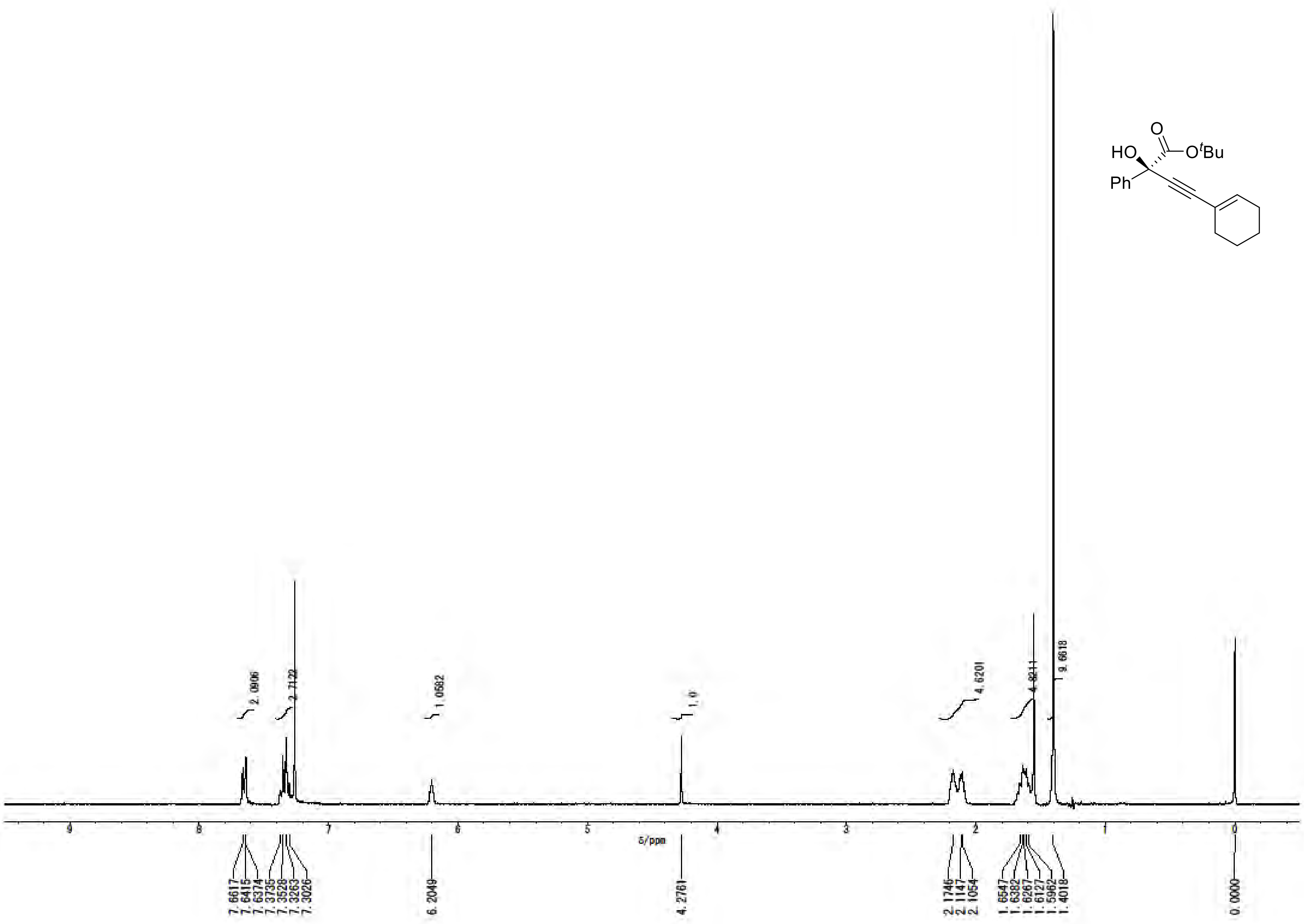

^{1}H NMR spectrum of **3cg** in $CDCl_3$

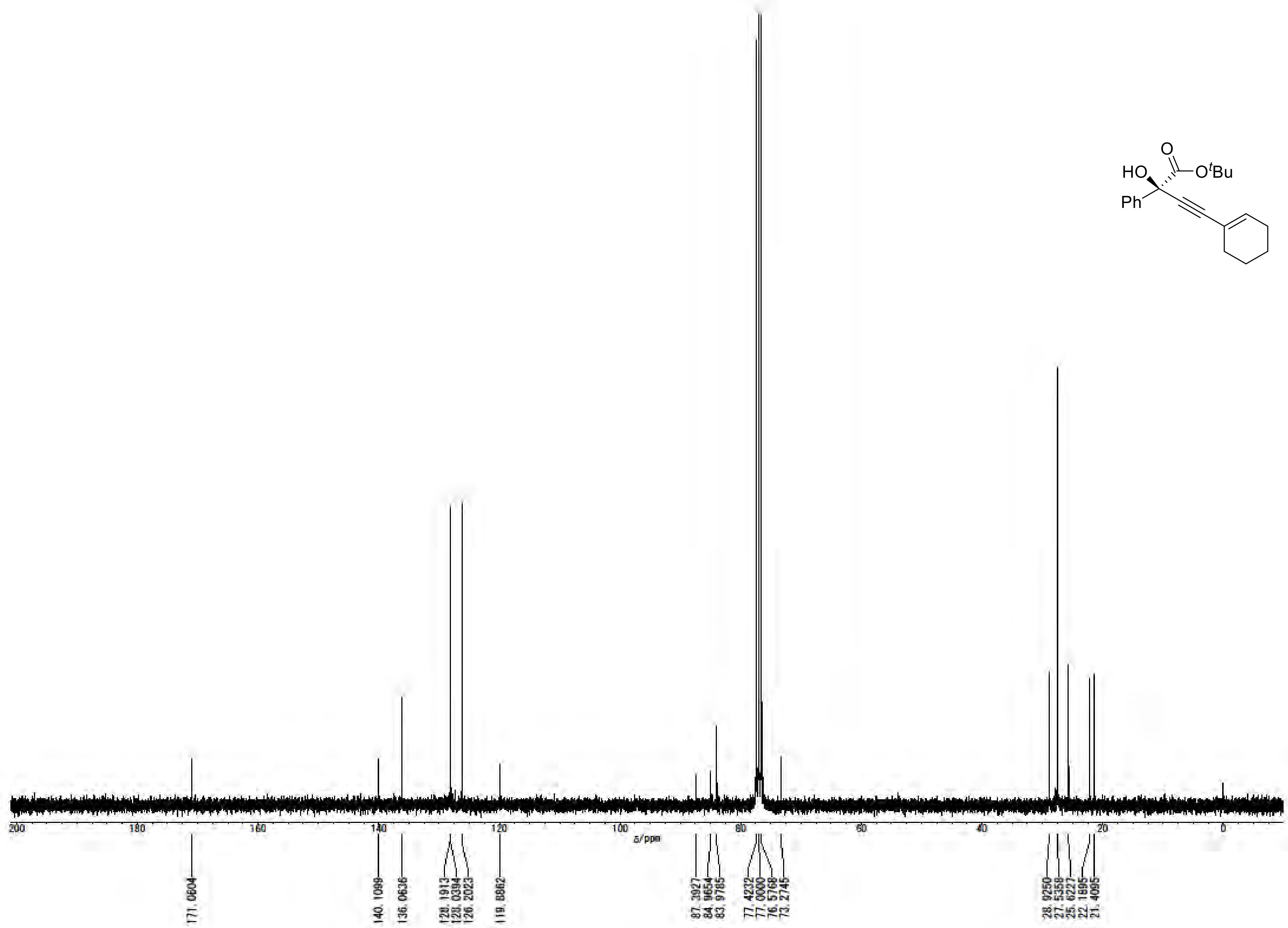

^{13}C NMR spectrum of **3cg** in $CDCl_3$

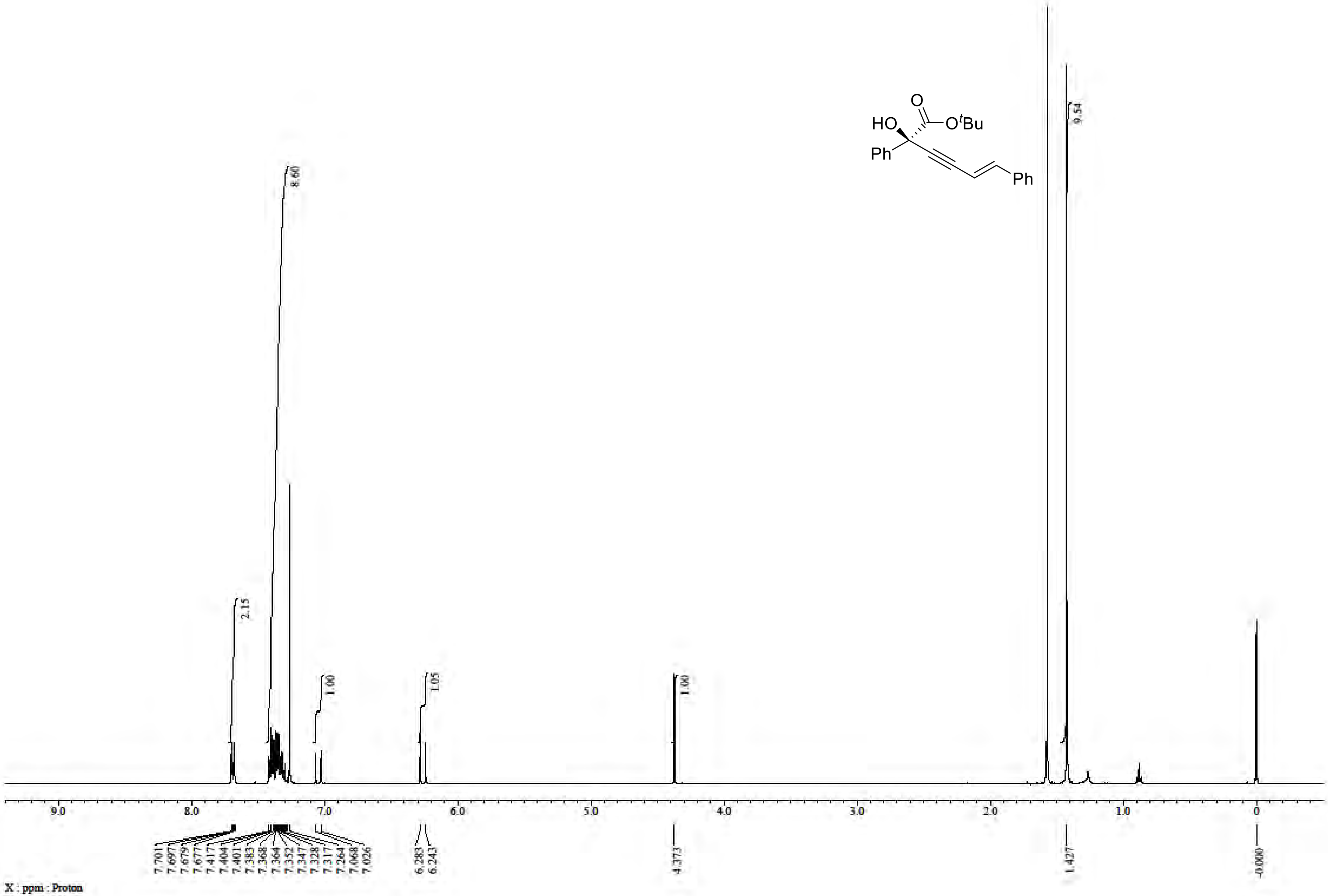

^{1}H NMR spectrum of **3ch** in $CDCl_3$

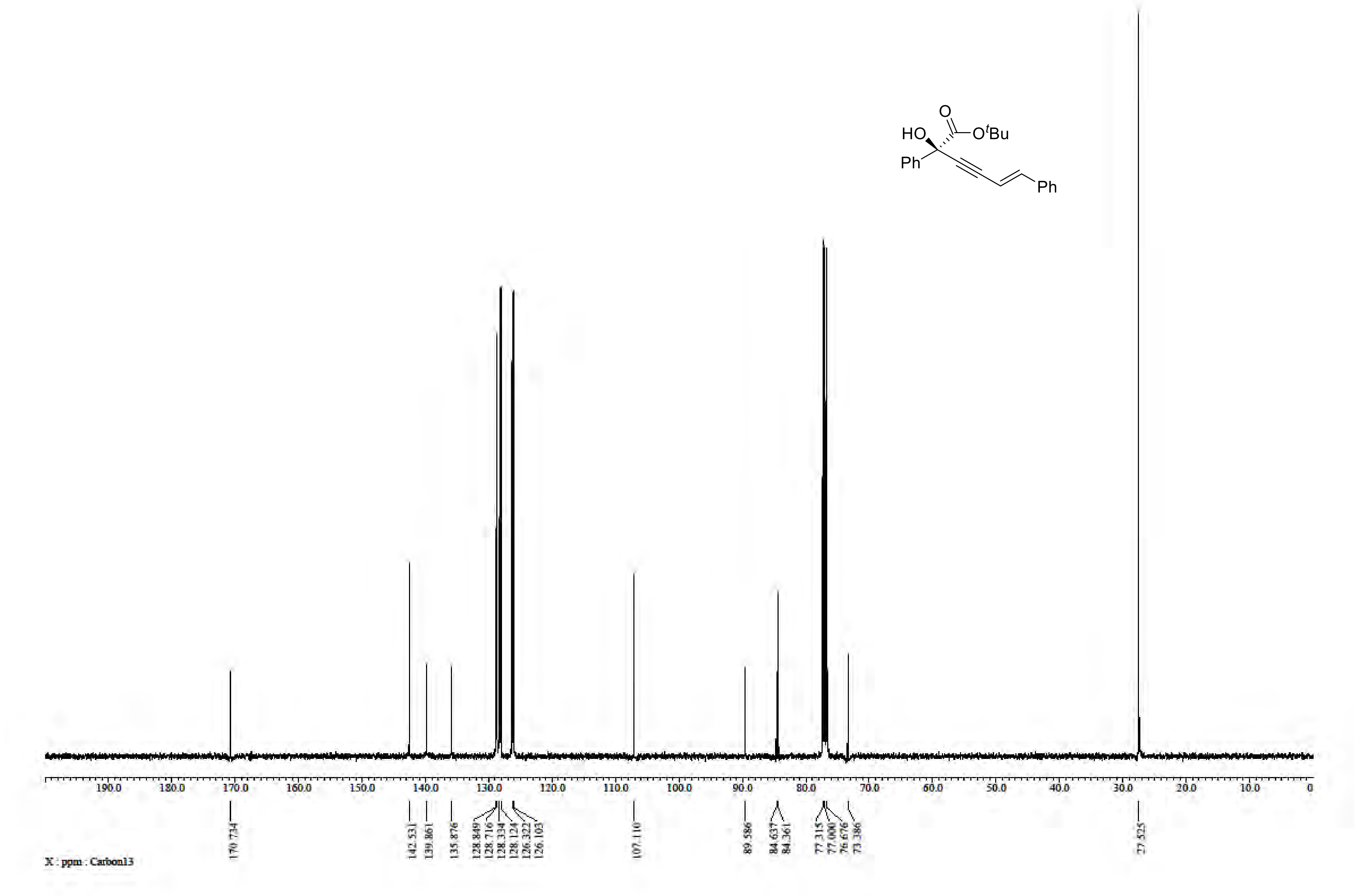

^{13}C NMR spectrum of **3ch** in $CDCl_3$

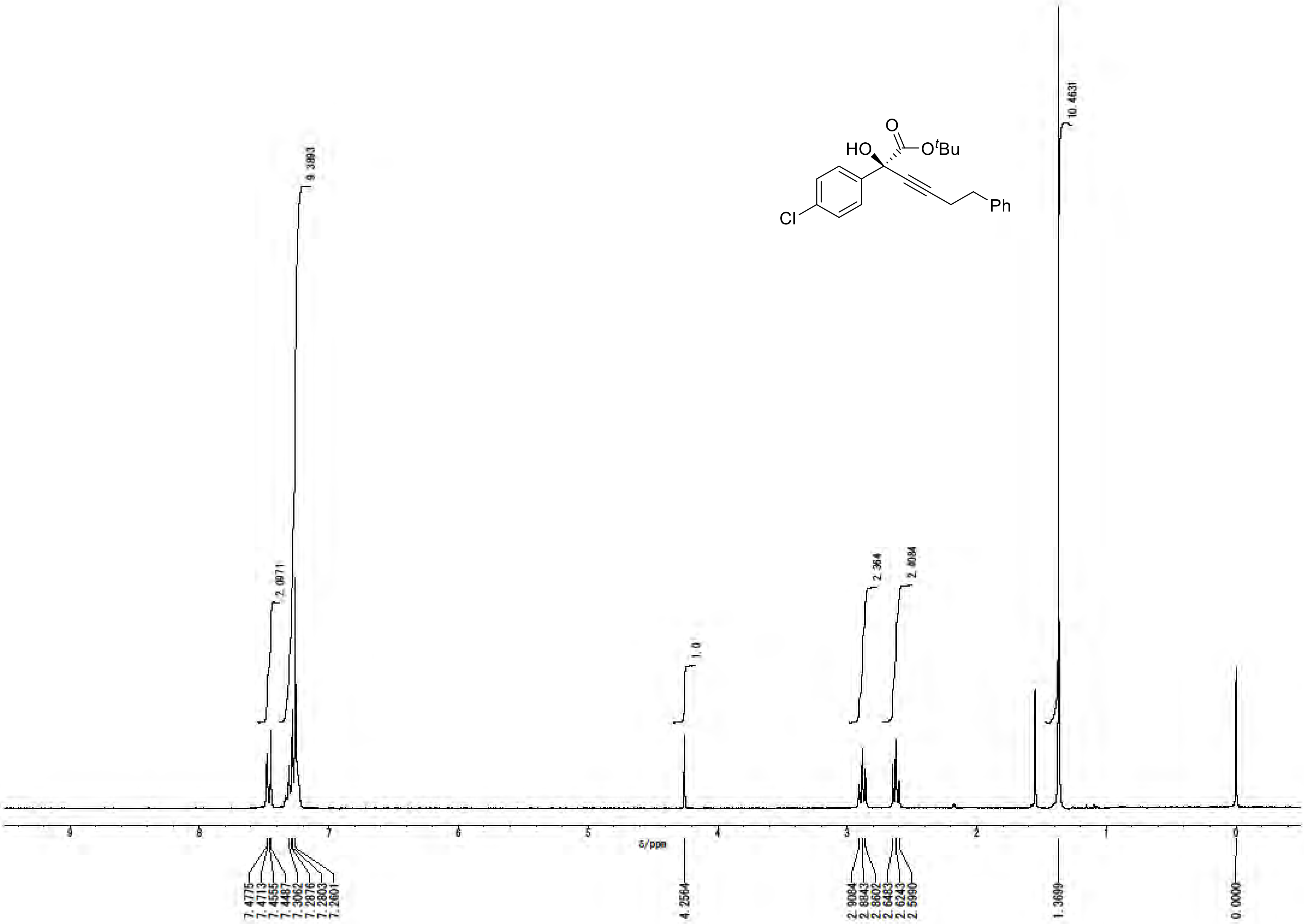

^{1}H NMR spectrum of **3fi** in $CDCl_3$

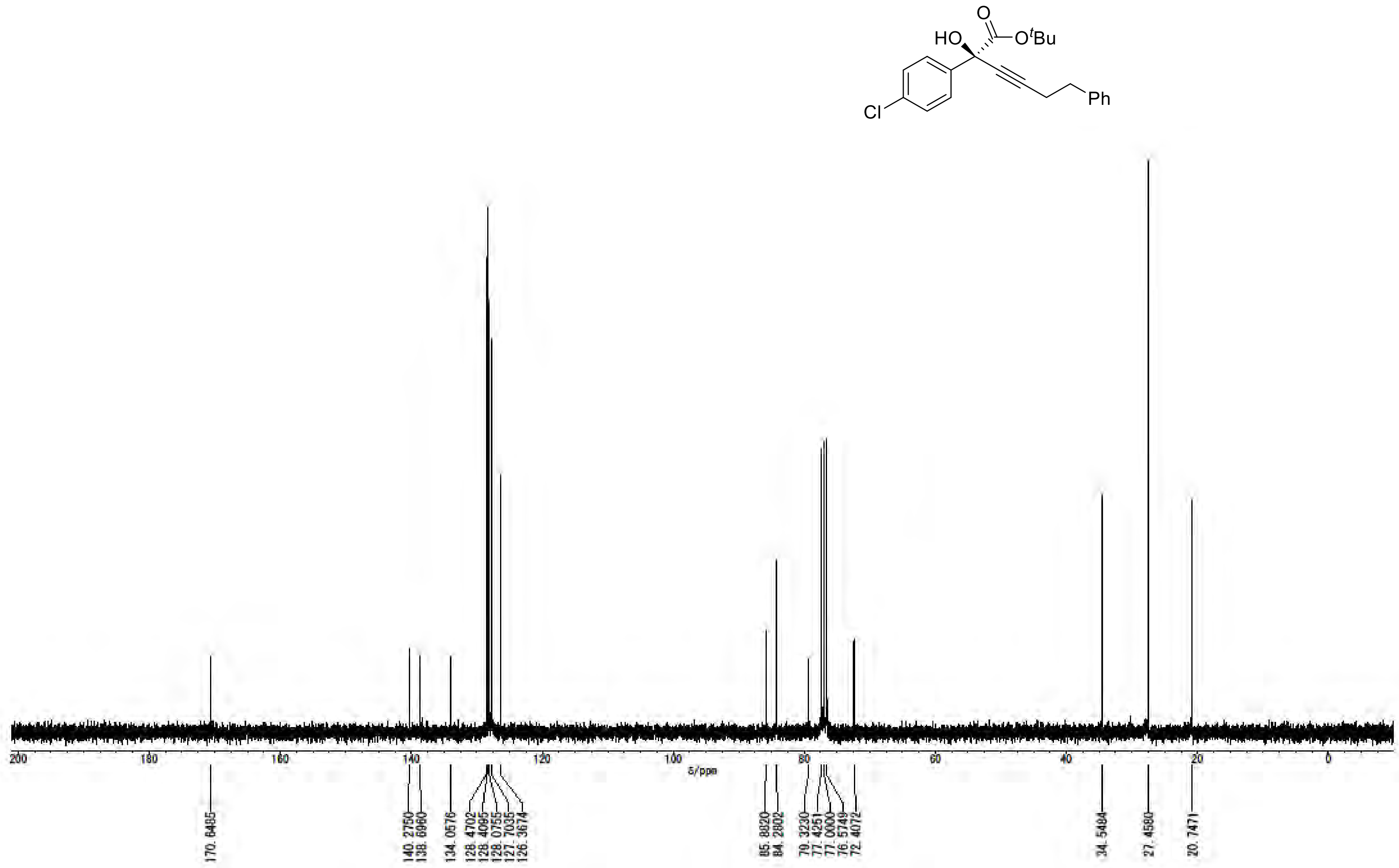

^{13}C NMR spectrum of **3fi** in $CDCl_3$

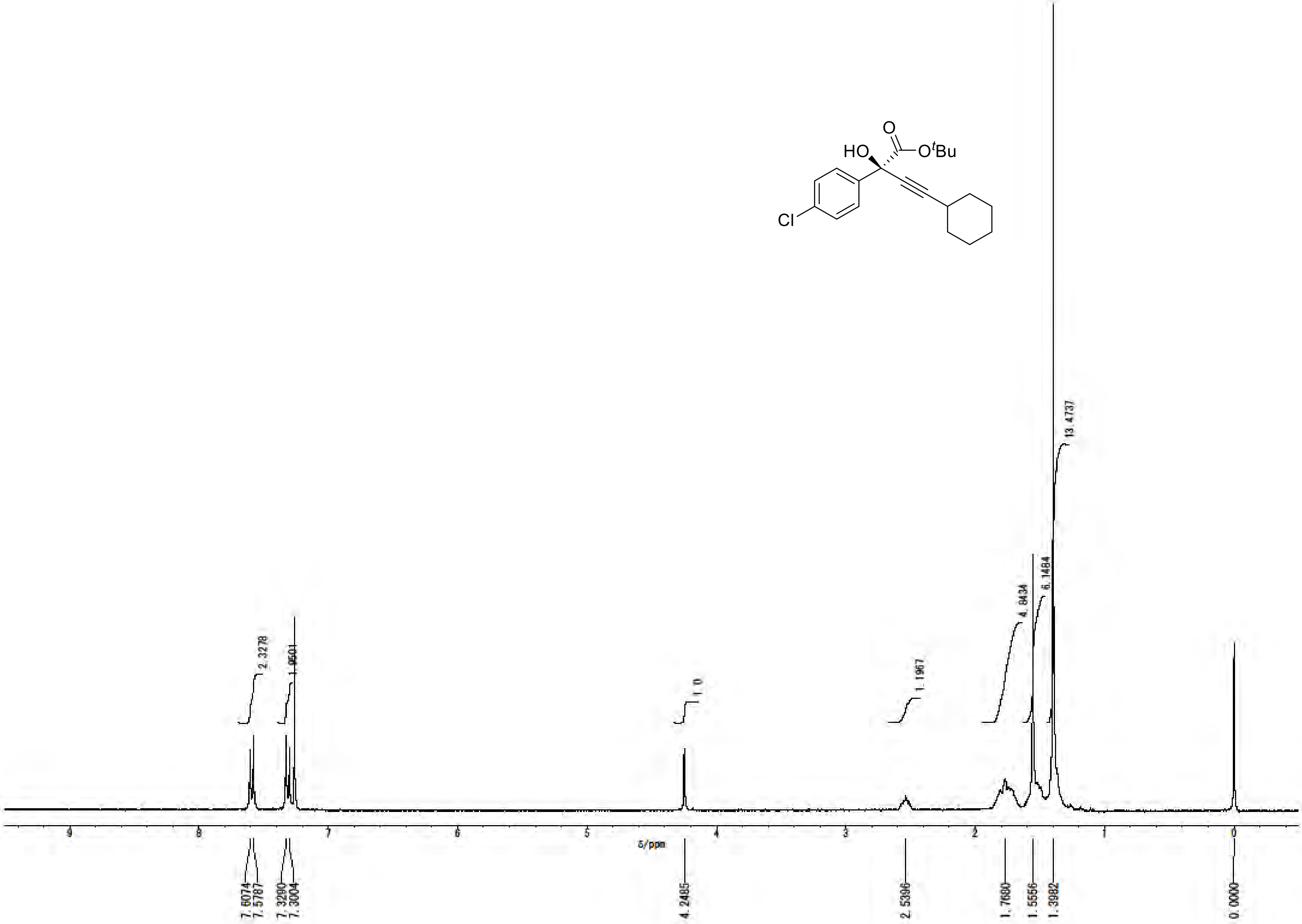

^{1}H NMR spectrum of **3fj** in $CDCl_3$

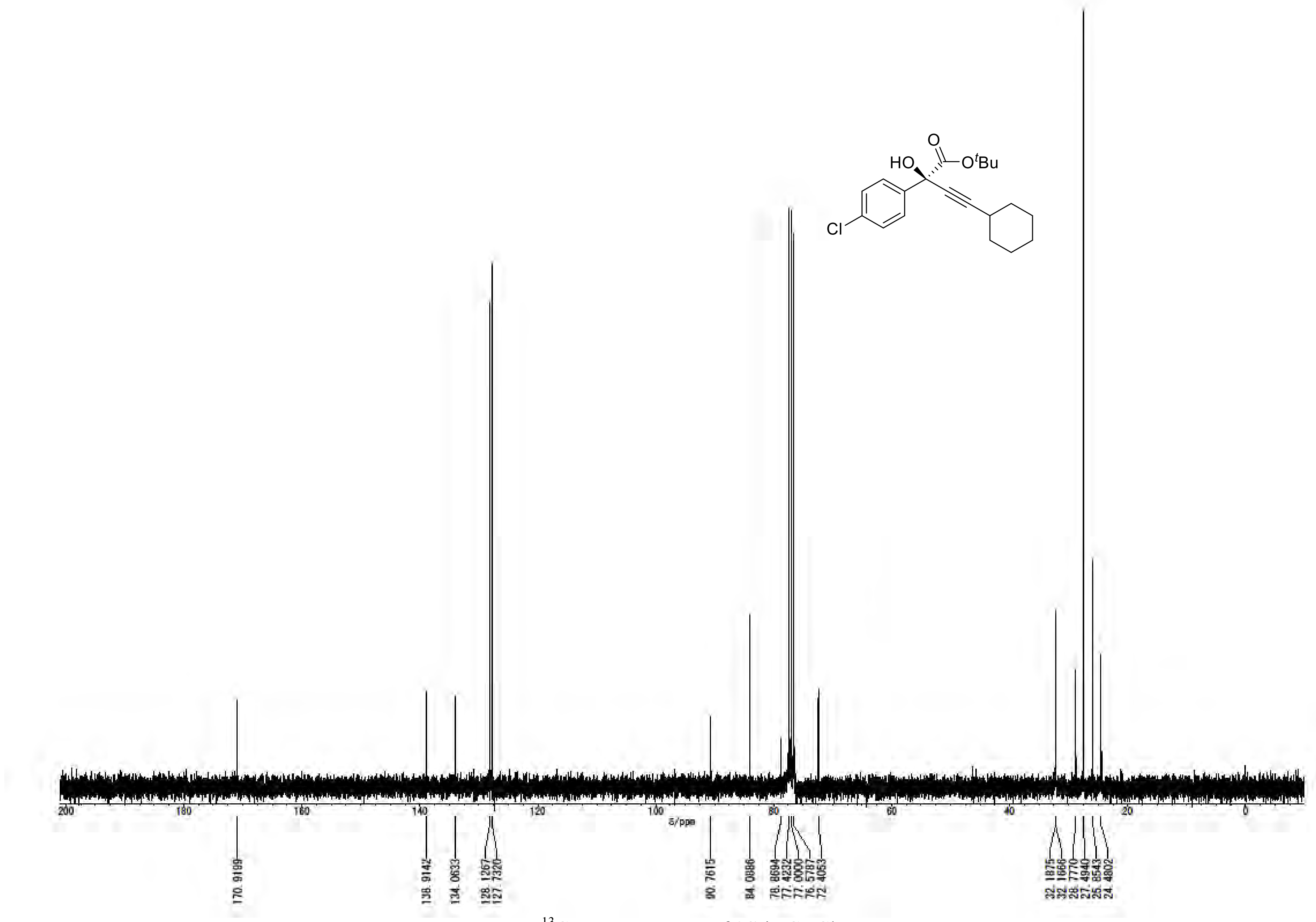

^{13}C NMR spectrum of **3fj** in $CDCl_3$

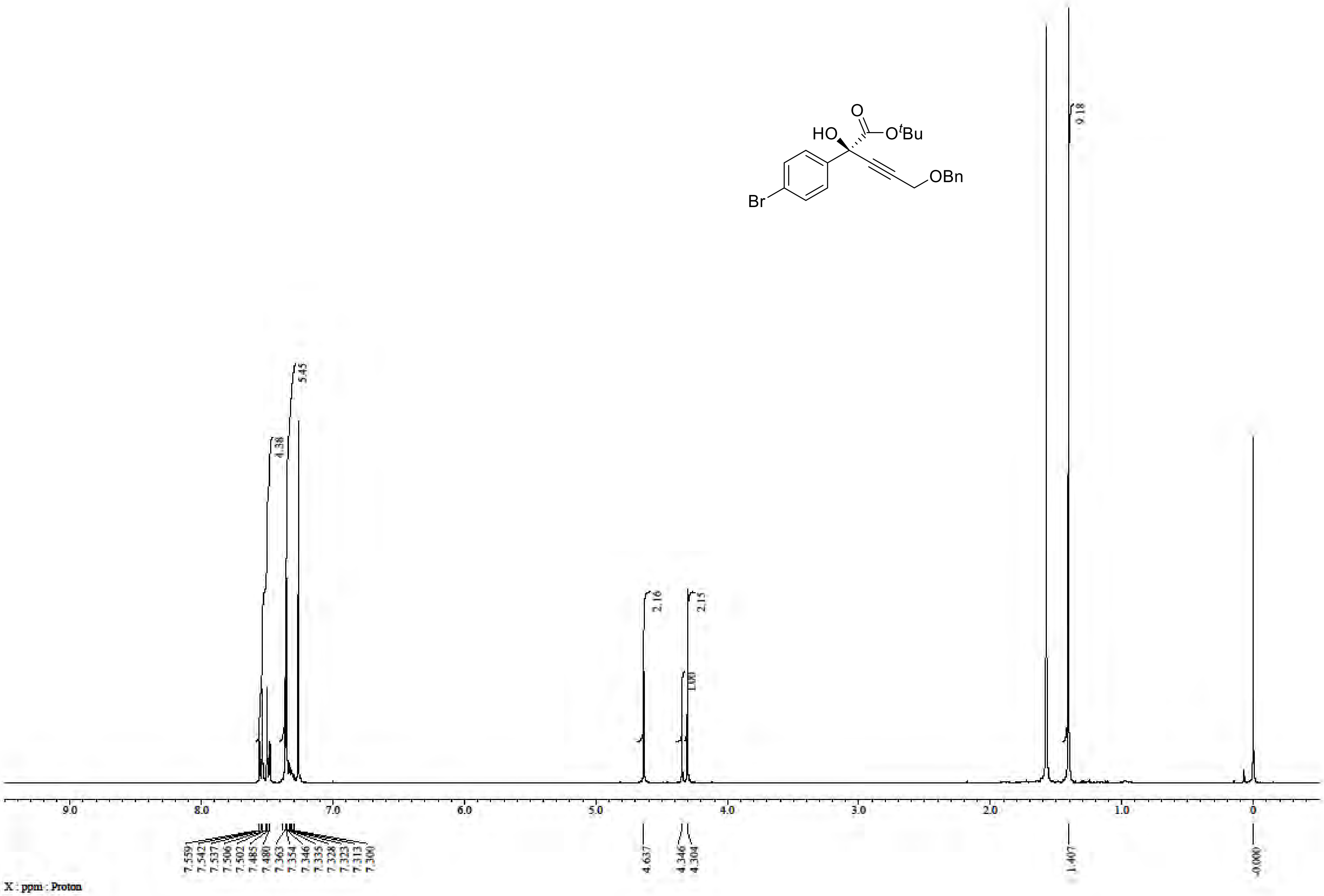

^{1}H NMR spectrum of **3gk** in $CDCl_3$

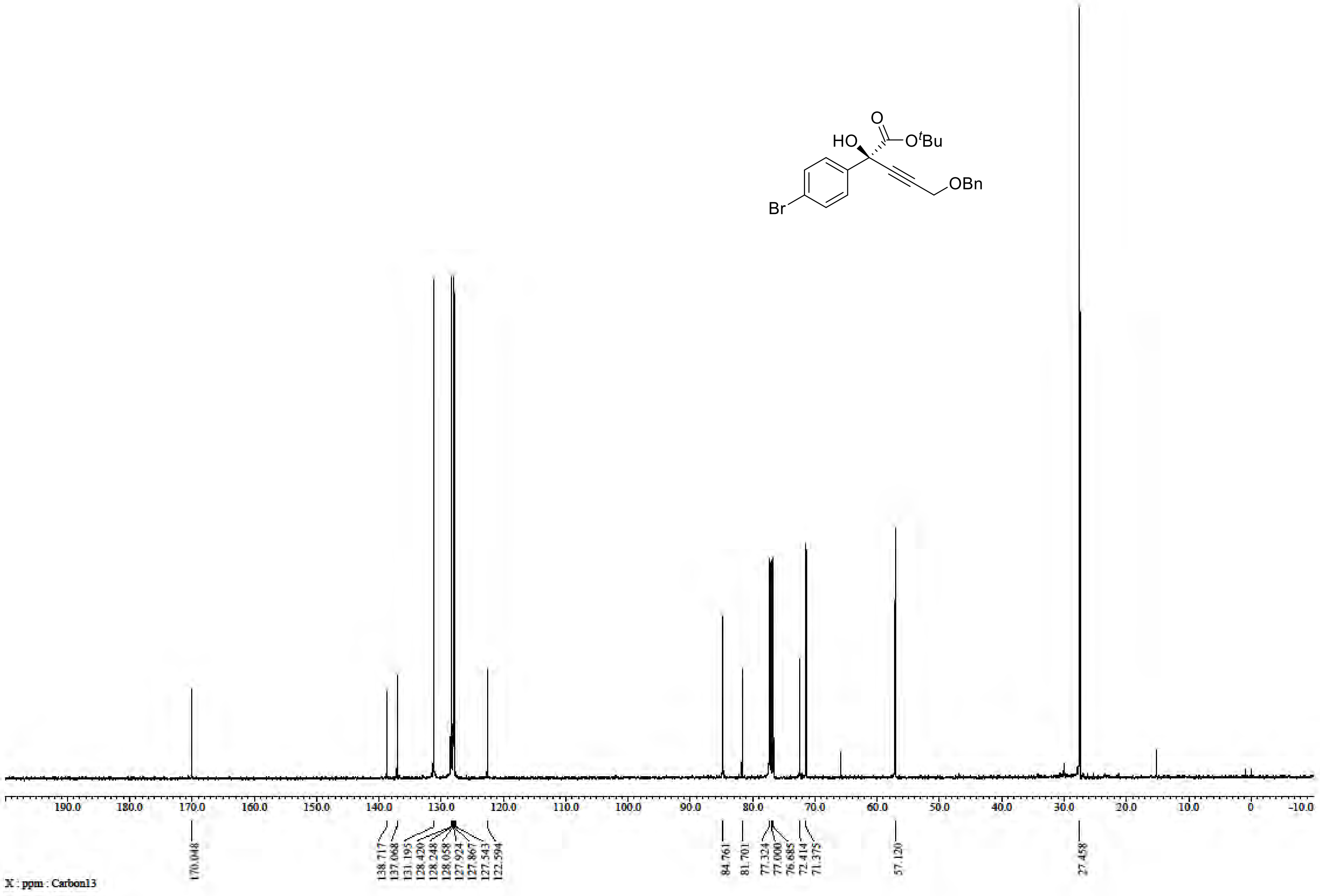

^{13}C NMR spectrum of **3gk** in $CDCl_3$

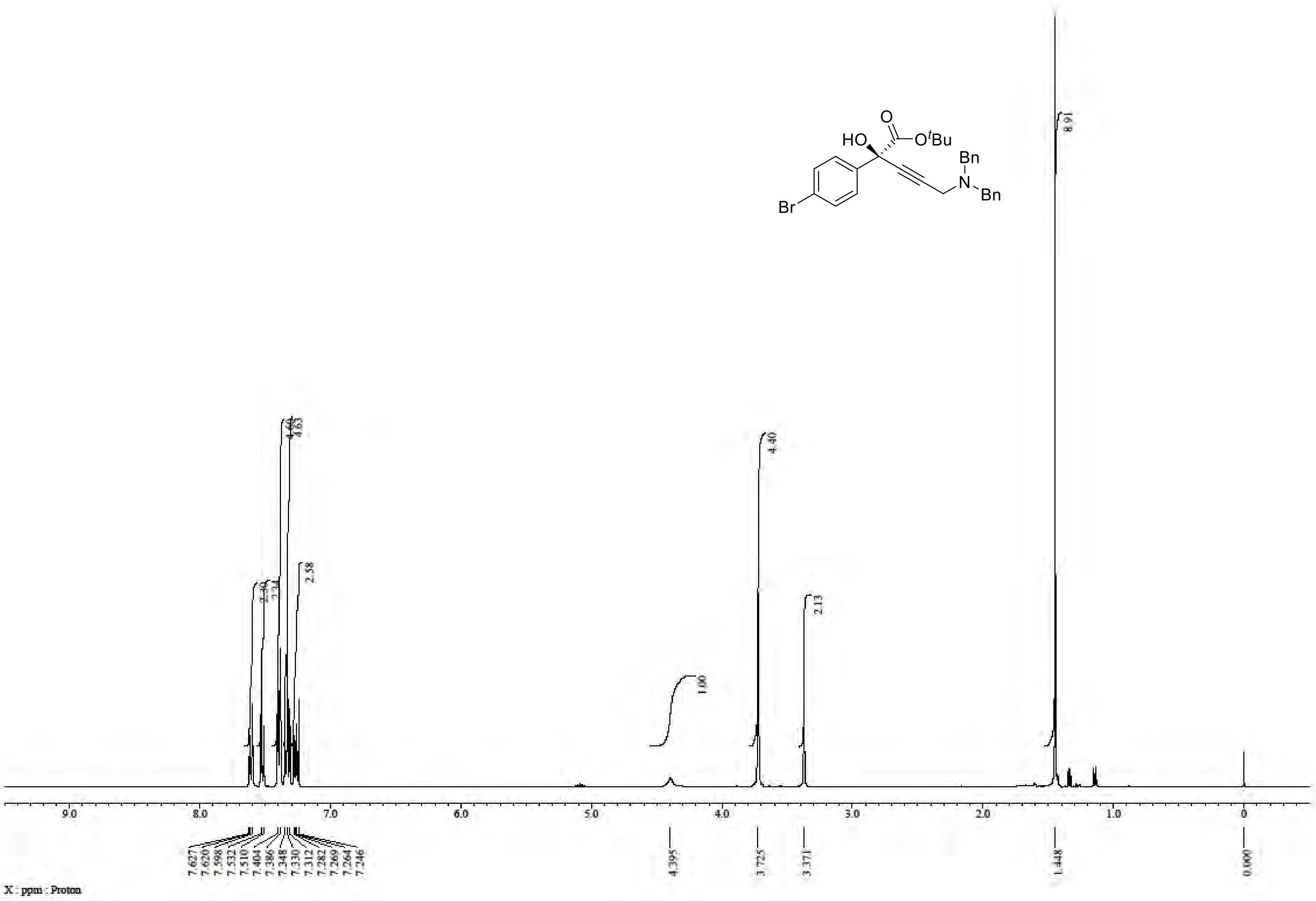

^{1}H NMR spectrum of **3gl** in $CDCl_3$

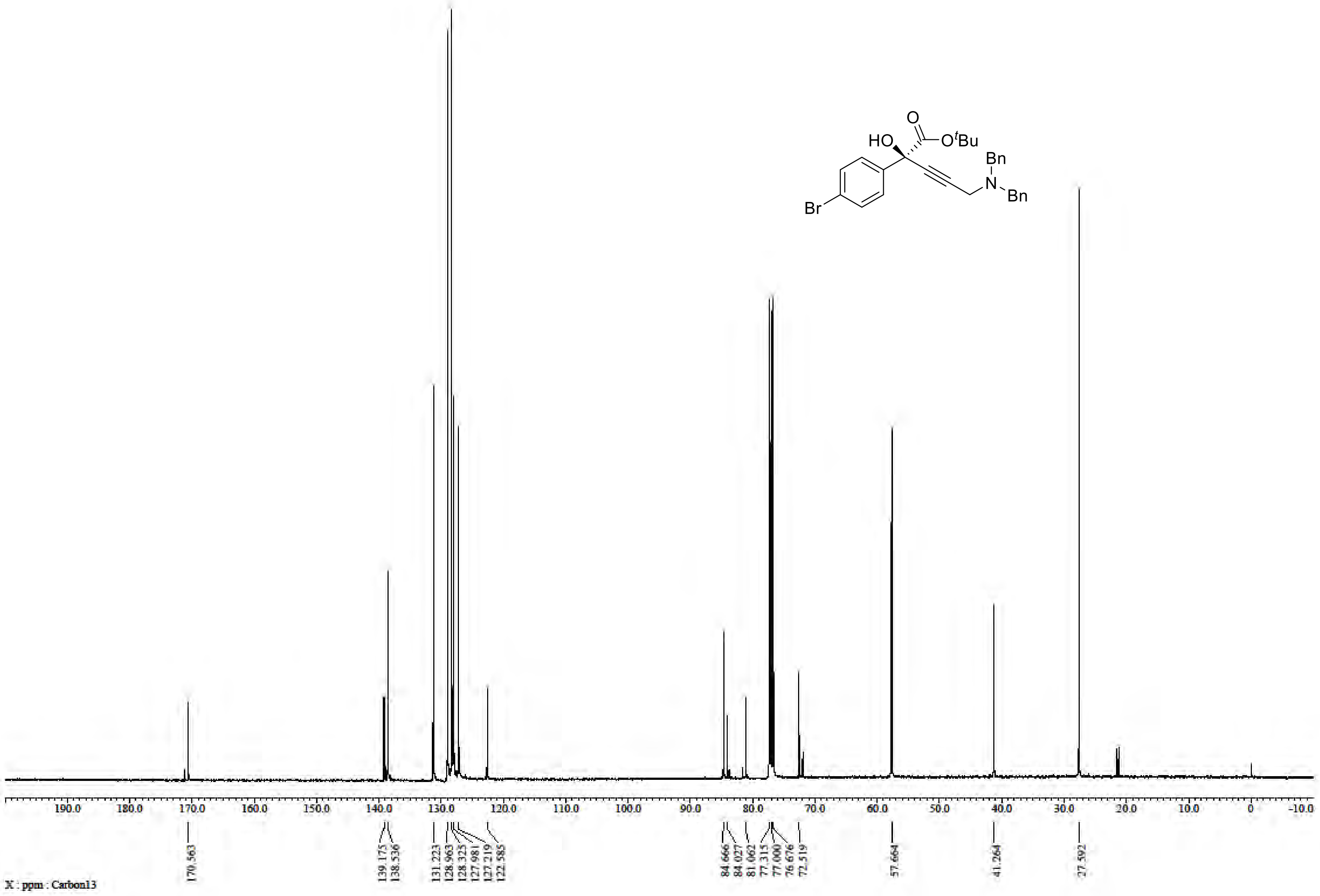

^{13}C NMR spectrum of **3gl** in $CDCl_3$

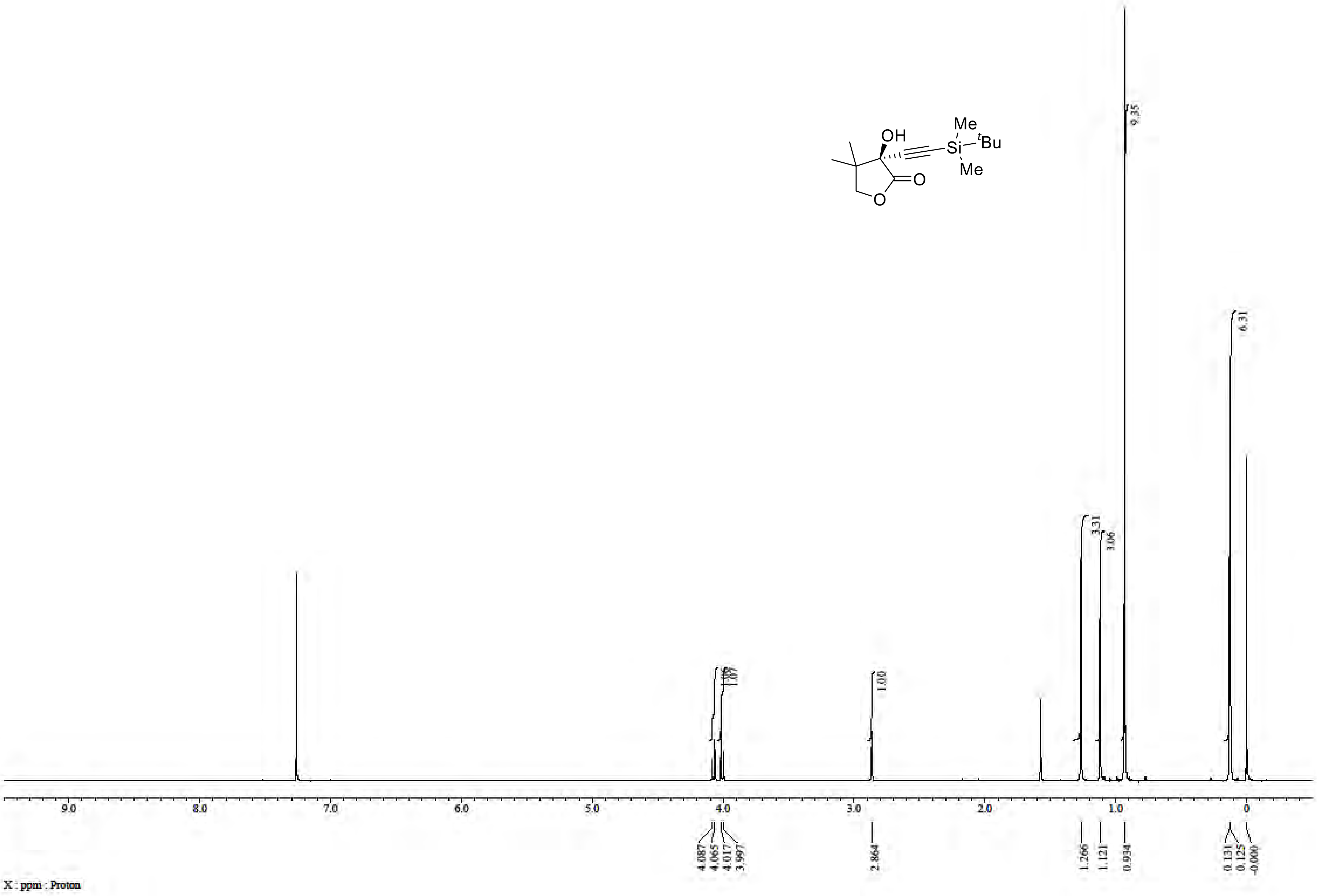

^{1}H NMR spectrum of **3rm** in $CDCl_3$

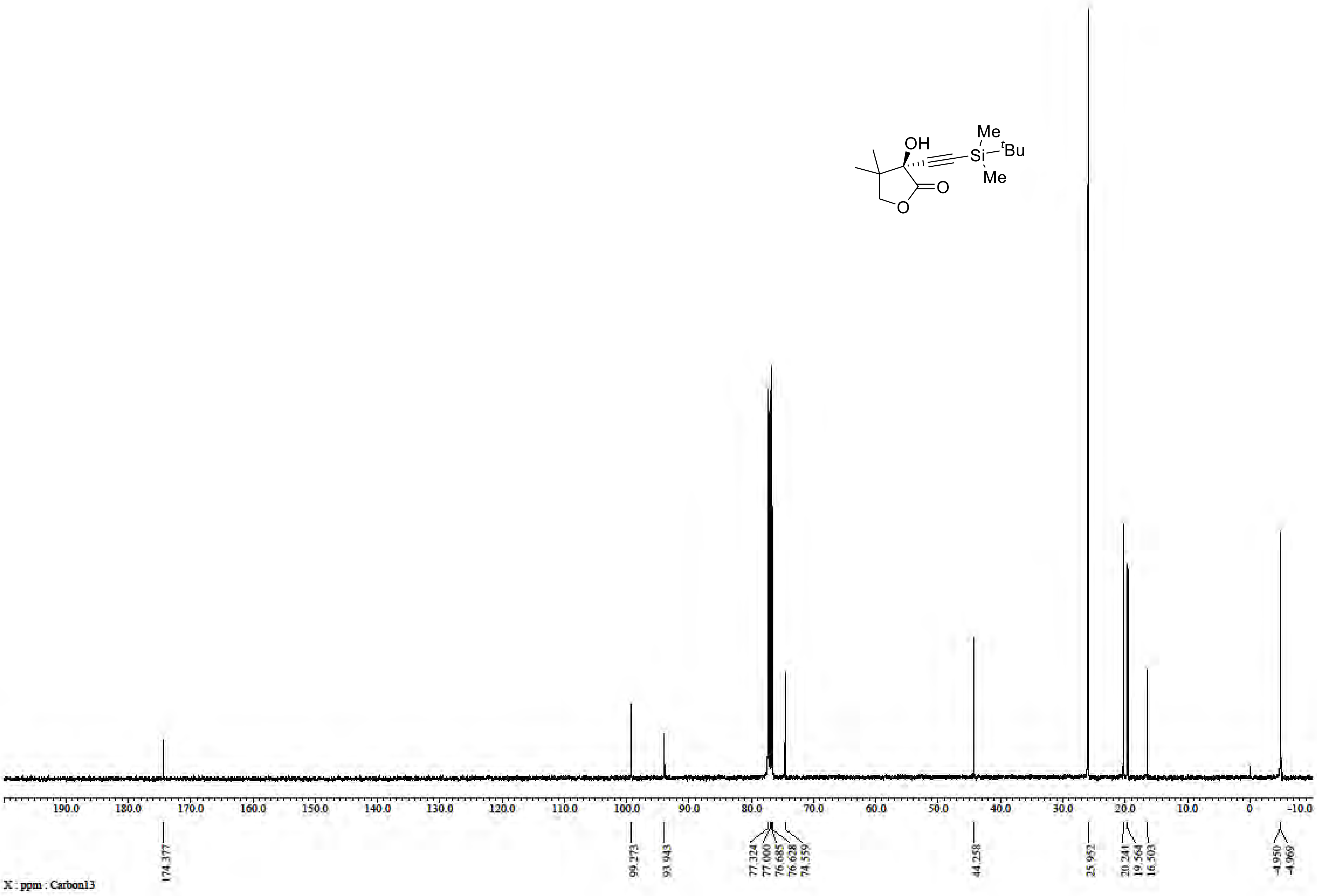

^{13}C NMR spectrum of **3rm** in $CDCl_3$
